# Assessments of different batches and dose levels of a two-dose Ad26.ZEBOV and MVA-BN-Filo vaccine regimen

**DOI:** 10.1038/s41541-021-00402-8

**Published:** 2021-12-20

**Authors:** Viki Bockstal, Auguste Gaddah, Neil Goldstein, Georgi Shukarev, Stephan Bart, Kerstin Luhn, Cynthia Robinson, Dickson Anumendem, Maarten Leyssen, Macaya Douoguih

**Affiliations:** 1 Janssen Vaccines & Prevention B.V., Leiden, The Netherlands; 2 Janssen Research & Development, Beerse, Belgium; 3 Optimal Research LLC, Rockville, MD USA

**Keywords:** Infectious diseases, Vaccines

## Abstract

Two phase 3 clinical studies were conducted in the USA to bridge across different Ad26.ZEBOV manufacturing processes and sites, and to evaluate the immunogenicity of different dose levels of Ad26.ZEBOV and MVA-BN-Filo. Study 1 evaluated the immunological equivalence of three batches of Ad26.ZEBOV administered as dose 1, followed by one batch of MVA-BN-Filo as dose 2. In Study 2, immunogenic non-inferiority of intermediate (Ad26.ZEBOV: 2 × 10^10^ viral particles [vp], MVA-BN-Filo: 5 × 10^7^ infectious units [Inf.U]) and low (8 × 10^9^ vp, 5 × 10^7^ Inf.U) doses of Ad26.ZEBOV and MVA-BN-Filo were evaluated against the full clinical dose (5 × 10^10^ vp, 1 × 10^8^ Inf.U). In Study 1, equivalence was demonstrated for two of three batch comparisons post-dose 1 and all three batches after the full regimen. Study 2 demonstrated a dose-dependent response; however, non-inferiority against the full clinical dose was not met. All regimens were well tolerated and immune responses were observed in all participants, regardless of manufacturing process or dose. Consistency of immunogenicity of different Ad26.ZEBOV batches was demonstrated and a dose-dependent response was observed after Ad26.ZEBOV, MVA-BN-Filo vaccination. ClinicalTrials.gov identifiers: NCT02543268; NCT02543567.

## Introduction

Reports of outbreaks of Ebola virus disease (EVD) across the African continent have increased since the first description of the hemorrhagic fever caused by Ebola virus (EBOV) in 1976^1^. With the occurrence of several outbreaks in the last decade, EVD has become a permanent public health threat. The two largest EVD outbreaks occurred in 2014–2016 in Guinea, Liberia, and Sierra Leone, with a case-fatality rate (CFR) of 40% (28,616 cases and 11,310 deaths)^2,3^, and in 2018–2020 in the Democratic Republic of Congo (DRC), with a CFR of 66% (3470 cases and 2287 deaths)^3,4^.

These major outbreaks triggered accelerated development of several vaccine candidates targeting the EBOV surface glycoprotein (GP)^5^. rVSV-ZEBOV-GP (Ervebo, Merck Sharp and Dohme) is a single-dose recombinant, replication-competent vesicular stomatitis viral vectored vaccine expressing the GP of the Kikwit variant of the Zaire EBOV species. This vaccine demonstrated high efficacy when used in a reactive manner in a ring-vaccination strategy^6,7^ and received approval by the U.S. Food and Drug Administration (FDA)^8^, in addition to a conditional approval from the European Medicines Agency^9^ and World Health Organization (WHO) prequalification^10^, for use in adults over 18 years of age. The WHO Strategic Advisory Group of Experts on Immunization (SAGE) recommends this vaccine for outbreak control of those at high risk of Ebola exposure^11^.

WHO SAGE also recommended vaccination of lower-risk populations with the two-dose Ad26.ZEBOV, MVA-BN-Filo vaccine regimen in the 2018–2020 outbreak in the DRC^11^. The urgent public health need for an additional effective vaccine for the prevention of EVD led to the approval of this regimen under exceptional circumstances by the European Commission for prophylactic use in those aged 1 year or older in July 2020^12–14^. In parallel, approval under an exceptional emergency situation was also granted by the Rwanda FDA in September 2019. Following the approval, a large vaccination campaign was implemented in Rwanda, aimed at protecting against the import of EBOV across the border with the DRC^15^. The first vaccine in this two-dose regimen is Ad26.ZEBOV (Zabdeno^®^, Janssen Vaccines), a recombinant, replication-incompetent adenovirus type 26 viral vector which encodes the Mayinga Ebola GP. The second vaccine is MVA-BN-Filo (Mvabea^®^, Bavarian Nordic), a recombinant, non-replicating modified vaccinia Ankara viral vector encoding GPs from Ebola Zaire (Mayinga), Sudan, and Marburg viruses and nucleoprotein from Taï Forest virus. Ad26.ZEBOV followed by MVA-BN-Filo in an approximate 8-week interval has been shown to be well tolerated and immunogenic in phase 1, 2, and 3 studies^16–21^.

The 2014–2016 West African outbreak highlighted the need to increase manufacturing capacity, which drove changes to the manufacturing process of Ad26.ZEBOV. Most notably, the virus seed strategy was changed (from a 1-tiered virus seed, based on a master virus seed (MVS) in Leiden, the Netherlands) to a 2-tiered seed approach. This 2-tiered approach involved manufacturing at two sites, in Leiden and Bern, Switzerland, using a working virus seed (WVS). Accordingly, two phase 3 clinical trials were performed in healthy adults in the United States of America (USA), using vaccine batches produced according to the intended 2-tiered virus seed commercial scale manufacturing processes.

The first study was designed to bridge across the two different manufacturing sites using different virus seeds of Ad26.ZEBOV. The second study was designed to evaluate the impact of the potency of the Ad26.ZEBOV and MVA-BN-Filo batches on the immunogenicity of the vaccination regimen. Furthermore, both studies would provide additional safety and immunogenicity data for the use of the vaccine regimen in people at risk of exposure to EBOV, such as healthcare workers, frontline workers, military and laboratory personnel, or other travelers to regions where there is potential exposure to Ebola.

## Results

### Baseline characteristics

**Fig. 1:**
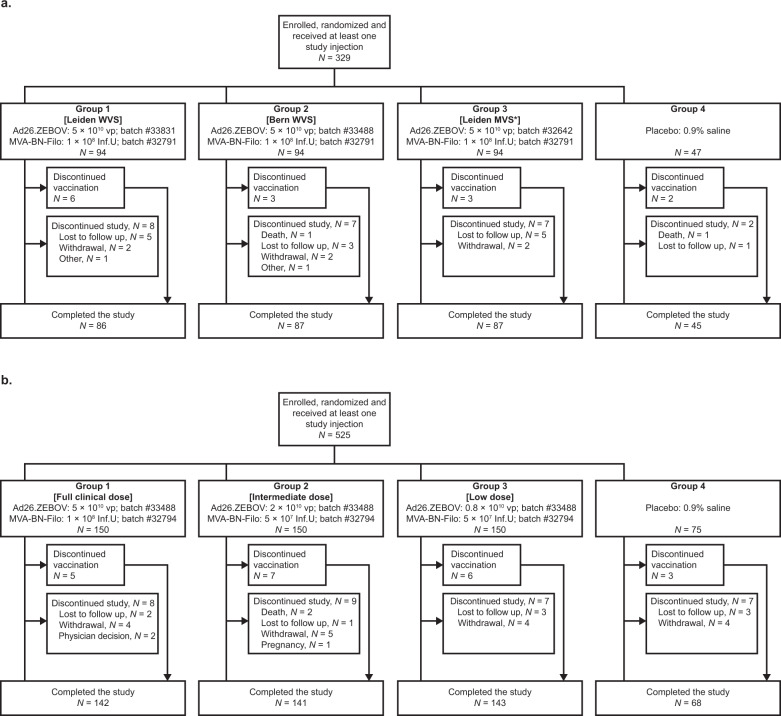
Study flow. Panel **a** shows the study flow for Study 1. Panel **b** shows the study flow for Study 2. Inf.U: infectious units; MVS: master virus seed; vp: viral particles; WVS: working virus seed. *Same Leiden MVS batch as used in phase 1/2 studies.

A total of 329 participants were enrolled and randomized in Study 1, and 525 in Study 2, according to the study designs detailed in Fig. 1. In Study 1 and Study 2, respectively, the first participant was enrolled on 21 September 2015 and 30 July 2015, and the date of the last participant last visit was 20 July 2016 and 29 November 2016. In general, the demographics were similar across groups within each study (Table 1). While there was a higher proportion of Hispanic or Latino participants (19.5%) in Study 1 than in Study 2 (6.7%), Study 2 had a higher proportion of White participants (79.6%) than Study 1 (57.4%). Attrition was similar in both studies: 305 of 329 (92.7%) participants completed Study 1, and 494 of 525 (94.1%) completed Study 2. Reasons for not completing were mainly withdrawal by the participants or loss to follow up (Fig. 1). Three pregnancies were reported in Study 2; as a result, two participants did not receive MVA-BN-Filo, and the third pregnancy was reported three weeks after the second vaccination.

**Table 1:** Demographics of the two study populations.

	Study 1: Group 1 [Leiden WVS]	Study 1: Group 2 [Bern WVS]	Study 1: Group 3 [Leiden MVS^a^]	Study 1: Group 4 [Placebo]	Study 2: Group 1 [Full clinical dose]	Study 2: Group 2 [Intermediate dose]	Study 2: Group 3 [Low dose]	Study 2: Group 4 [Placebo]
Ad26.ZEBOV (vp)	5 × 10^10^	5 × 10^10^	5 × 10^10^	Placebo	5 × 10^10^	2 × 10^10^	8 × 10^9^	Placebo
Batch	#33831	#33488	#32642	—	#33488	#33488	#33488	—
MVA-BN-Filo (Inf.U)	1 × 10^8^	1 × 10^8^	1 × 10^8^	Placebo	1 × 10^8^	5 × 10^7^	5 × 10^7^	Placebo
Batch	#32791	#32791	#32791	—	#32794	#32794	#32794	—
*N* =	94	94	94	47	150	150	150	75
Age (years), mean ± SD	31.8 ± 9.8	33.5 ± 9.6	30.7 ± 9.2	32.0 ± 9.4	34.0 ± 9.0	34.8 ± 9.4	34.6 ± 10.1	33.0 ± 8.8
Male, *n* (%)	56 (59.6)	48 (51.1)	52 (55.3)	21 (44.7)	85 (56.7)	67 (44.7)	67 (44.7)	37 (49.3)
BMI (kg/m^2^), mean ± SD	28.07 ± 4.91	28.61 ± 4.97	27.18 ± 4.94	28.82 ± 5.73	27.96 ± 4.84	27.21 ± 5.11	27.42 ± 4.72	28.15 ± 4.99
Race, *n* (%)								
American Indian/Alaskan native	0	0	0	1 (2.1)	0	1 (0.7)	0	0
Asian	2 (2.1)	3 (3.2)	2 (2.1)	1 (2.1)	1 (0.7)	2 (1.3)	1 (0.7)	1 (1.3)
Black/African American	35 (37.2)	34 (36.2)	33 (35.1)	11 (23.4)	21 (14.0)	26 (17.3)	29 (19.3)	18 (24.0)
Native Hawaiian or Pacific Islander	0	3 (3.2)	0	0	1 (0.7)	0	0	0
White	52 (55.3)	51 (54.3)	55 (58.5)	31 (66.0)	124 (82.7)	120 (80.0)	118 (78.7)	56 (74.7)
Multiple	4 (4.3)	3 (3.2)	4 (4.3)	3 (6.4)	3 (2.0)	1 (0.7)	2 (1.3)	0
Ethnicity, *n* (%)								
Hispanic or Latino	20 (21.3)	15 (16.0)	17 (18.1)	12 (25.5)	13 (8.7)	11 (7.3)	10 (6.7)	1 (1.3)
Not Hispanic or Latino	74 (78.7)	79 (84.0)	77 (81.9)	35 (74.5)	136 (90.7)	139 (92.7)	140 (93.3)	74 (98.7)
Unknown	0	0	0	0	1 (0.7)	0	0	0

^a^Same Leiden MVS batch as used in phase 1/2 studies.

BMI: body mass index; Inf.U: infectious units; MVS: master virus seed; SD: standard deviation; vp: viral particles; WVS: working virus seed.

In the following sections, we describe the EBOV GP-specific humoral immune responses assessed in Study 1 and in Study 2 separately (in both studies, EBOV GP-specific antibody levels were low or not quantifiable at all assessed time points in placebo recipients). In a subsequent section we describe the combined safety and tolerability results from both studies.

### Immunogenicity: Study 1 (assessment of Ad26.ZEBOV manufacturing consistency)

Immunological equivalence was evaluated for three different batches of Ad26.ZEBOV, manufactured from either a WVS at the manufacturing facility in Leiden (Group 1), the same WVS at the manufacturing facility in Bern (Group 2), or the MVS at the manufacturing facility in Leiden (Group 3).

**Fig. 2:**
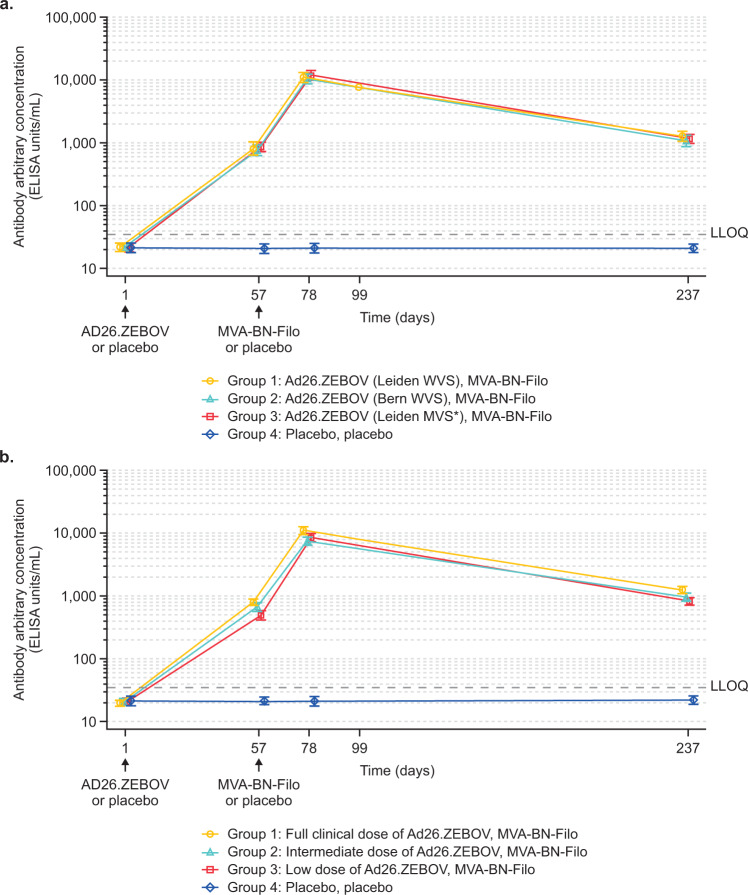
Geometric mean concentrations of EBOV-specific binding antibodies in the two studies. Panel **a** (Study 1) shows the geometric mean profile of the three different batches of Ad26.ZEBOV (5 × 10^10^ vp) (Group 1: Leiden WVS, batch #33831; Group 2: Bern WVS, batch #33488; Group 3: Leiden MVS*, batch #32642), MVA-BN-Filo (1 × 10^8^ Inf.U), or placebo, placebo (Group 4) administered 56 days apart. Panel **b** (Study 2) shows the geometric mean profile of the different dose levels - Group 1 (full clinical dose: Ad26.ZEBOV [5 × 10^10^ vp], MVA-BN-Filo [1 × 10^8^ Inf.U]), Group 2 (intermediate dose: Ad26.ZEBOV [2 × 10^10^ vp], MVA-BN-Filo [5 × 10^7^ Inf.U]), Group 3 (low dose: Ad26.ZEBOV [8 × 10^9^ vp], MVA-BN-Filo [5 × 10^7^ Inf.U]), or Group 4 (placebo, placebo), administered 56 days apart. Both panels show the change in geometric mean concentrations over time (for actual values, see Table 2). Error bars in both panels represent the 95% confidence intervals. Inf.U: infectious units; LLOQ: lower limit of quantification; MVS: master virus seed; vp: viral particles; WVS: working virus seed. *Same Leiden MVS batch as used in phase 1/2 studies.

For EBOV GP-specific binding antibody responses, at day 57, following Ad26.ZEBOV injection on day 1 (but prior to administration of MVA-BN-Filo), responder rates were 96.5–100% for the three batches of Ad26.ZEBOV vaccine with geometric mean concentrations (GMCs) of 813 ELISA units/mL (EU/mL) (95% confidence interval [CI]: 632–1046), 745 EU/mL (95% CI: 603–921), and 851 EU/mL (95% CI: 720–1006) for Groups 1, 2, and 3 respectively (Fig. 2; Table 2). At day 78, 21 days post-MVA-BN-Filo administration, the responder rate was 100% in all three groups, with similar GMCs (11,089 EU/mL (95% CI: 9323–13,189), 10,337 EU/mL (95% CI: 8660–12,339), and 11,790 EU/mL (95% CI: 9701–14,328) in the three groups. At day 237, 6 months post-MVA-BN-Filo administration, binding antibody responses persisted in almost all (97.6–98.7%) participants, with GMCs of 1262 EU/mL (95% CI: 1029–1549), 1053 EU/mL (95% CI: 846–1310), and 1147 EU/mL (95% CI: 948–1387) in Groups 1, 2, and 3, respectively.

**Table 2:** EBOV-specific binding antibodies (ELISA U/mL) GMCs and responder rates (%) in both studies^a^.

	Study 1: Group 1 [Leiden WVS]	Study 1: Group 2 [Bern WVS]	Study 1: Group 3 [Leiden MVS^b^]	Study 2: Group 1 [Full clinical dose]	Study 2: Group 2 [Intermediate dose]	Study 2: Group 3 [Low dose]
Day 1 (Baseline), *N* =	85	86	87	140	131	136
GMC, EU/mL (95% CI)	<LLOQ (<LLOQ–LLOQ)	<LLOQ (<LLOQ–<LLOQ)	<LLOQ (<LLOQ–<LLOQ)	<LLOQ (<LLOQ–<LLOQ)	<LLOQ (<LLOQ–<LLOQ)	<LLOQ (<LLOQ–<LLOQ)
Day 57 (56 days post-dose 1), *N* =	85	88	88	140	131	136
GMC, EU/mL (95% CI)	813 (632–1046)	745 (603–921)	851 (720–1006)	793 (698–902)	669 (571–784)	496 (422–582)
Responders, *n/N*^c^ (%)	82/85 (96.5)	83/86 (96.5)	87/87 (100)	135/140 (96.4)	127/131 (96.9)	131/136 (96.3)
Day 78 (21 days post-dose 2), *N* =	81	87	86	135	123	130
GMC, EU/mL (95% CI)	11,089 (9323–13,189)	10,337 (8660–12,339)	11,790 (9701–14,328)	11,054 (9673–12,633)	7524 (6472–8746)	8538 (7338–9934)
Responders, *n/N*^c^ (%)	81/81 (100)	85/85 (100)	85/85 (100)	135/135 (100)	123/123 (100)	130/130 (100)
Day 237 (180 days post-dose 2), *N* =	82	82	80	131	121	129
GMC, EU/mL (95% CI)	1262 (1029–1549)	1053 (846–1310)	1147 (948–1387)	1263 (1100–1450)	962 (822–1125)	831 (716–965)
Responders, *n/N*^c^ (%)	80/82 (97.6)	78/80 (97.5)	78/79 (98.7)	129/131 (98.5)	119/121 (98.3)	127/129 (98.4)

^a^Placebo groups (Group 4 in both studies) were lower than the LLOQ throughout.

^b^Same Leiden MVS batch as used in phase 1/2 studies.

^c^Number of participants with data at both baseline and at that time point.

CI: confidence interval; EBOV: Ebola virus; EU: ELISA units; GMC: geometric mean concentration; Inf.U: infectious units; LLOQ: lower limit of quantification; MVS: master virus seed; vp: viral particles; WVS: working virus seed.

The primary objective of Study 1 was to demonstrate immunological equivalence between the Ad26.ZEBOV batch manufactured in Bern from WVS (Group 2) and the batch manufactured in Leiden from MVS (roup 3) 56 days after Ad26.ZEBOV vaccination, using the pre-specified equivalence margin of ^2^/_3_ (0.67) at the lower bound to 1^1^/_2_ (1.5) at the upper bound. At day 57, the GMC ratio of the Bern batch (Group 2) versus the Leiden batch prepared from MVS (Group 3) was 0.9 (95% CI: 0.65–1.17). Equivalence could not be demonstrated as the lower limit of the 95% CI of the GMC ratio was below the lower limit of the equivalence criterion set at 0.67 (Table 3). The post-hoc observation that this study was underpowered (42%) to conclude on the primary objective is addressed in the Discussion section.

**Table 3:** Primary, secondary, and exploratory equivalence (Study 1) and non-inferiority (Study 2) assessments; per protocol analysis set.

Assessment type (Criterion)	Comparison	GMC ratio (95% CI)	Equivalent: Y/N
Study 1: Primary (^2^/_3_ [0.67] <95% CI ratio <1^1^/_2_ [1.5])			
Day 57	Group 2 [Bern WVS] vs Group 3 [Leiden MVS^a^]	0.9 (0.65–1.17)	N
Study 1: Secondary (^2^/_3_ [0.67]) <95% CI ratio <1^1^/_2_ [1.5])			
Day 57	Group 1 [Leiden WVS] vs Group 2 [Bern WVS]	1.1 (0.81–1.47)	Y
Day 57	Group 1 [Leiden WVS] vs Group 3 [Leiden MVS^a^]	1.0 (0.71–1.29)	Y
Day 78	Group 1 [Leiden WVS] vs Group 2 [Bern WVS]	1.1 (0.83–1.38)	Y
Day 78	Group 1 [Leiden WVS] vs Group 3 [Leiden MVS^a^]	0.9 (0.73–1.21)	Y
Day 78	Group 2 [Bern WVS] vs Group 3 [Leiden MVS^a^]	0.9 (0.68–1.13)	Y
Study 2: Primary (^2^/_3_ [0.67] <95% CI ratio)			
Day 78	Group 2 [intermediate dose] vs Group 1 [full clinical dose]	0.7 (0.56–0.83)	N
Day 78	Group 3 [low dose] vs Group 1 [full clinical dose]^b^	0.8 (0.63–0.94)	—
Study 2: Exploratory (^1^/_2_ [0.5] <95% CI ratio)			
Day 78	Group 2 [intermediate dose] vs Group 1 [full clinical dose]	0.7 (0.56–0.83)	Y
Day 78	Group 3 [low dose] vs Group 1 [full clinical dose]	0.8 (0.63–0.94)	Y
Study 2: Exploratory^c^ (^2^/_3_ [0.67] <95% CI ratio)			
Day 57	Group 2 [intermediate dose] vs Group 1 [full clinical dose]	0.8 (0.68–1.04)	Y
Day 57	Group 3 [low dose] vs Group 1 [full clinical dose]	0.6 (0.51–0.77)	N

^a^Same Leiden MVS batch as used in phase 1/2 studies.

^b^Hierarchical testing (i.e., to be tested only if non-inferiority is established for the primary comparison of Group 2 vs Group 1).

^c^Post-hoc exploratory analysis.

CI: confidence interval; GMC: geometric mean concentration; MVS: master virus seed; N: no; WVS: working virus seed; Y: yes.

A secondary objective of Study 1 was to demonstrate equivalence of the Ad26.ZEBOV batch manufactured in Leiden from WVS (Group 1) versus the batch manufactured in Leiden from MVS (Group 3), and the Ad26.ZEBOV batch manufactured in Leiden from WVS (Group 1) versus the batch manufactured in Bern from WVS (Group 2), using the same pre-specified equivalence margin specified above. At day 57, following Ad26.ZEBOV injection on day 1 but prior to administration of MVA-BN-Filo, the GMC ratio of the Leiden WVS batch (Group 1) versus the Leiden MVS batch (Group 3) was 1.0 (95% CI: 0.71–1.29) and the GMC ratio of the Leiden WVS batch (Group 1) versus the Bern WVS batch (Group 2) was 1.1 (95% CI: 0.81–1.47). The equivalence criterion was met for both secondary comparisons as the 95% CI of the respective GMC ratios fell within the range of 0.67–1.5.

A further secondary objective of Study 1 was to demonstrate equivalence of the three different Ad26.ZEBOV batches upon completion of the two-dose vaccination regimen, using the same pre-specified equivalence margin. At day 78, 21 days post-MVA-BN-Filo, the GMC ratio of Group 1 versus Group 2 was 1.1 (95% CI: 0.83–1.38), of Group 1 versus Group 3 was 0.9 (95% CI: 0.73–1.21), and of Group 2 versus Group 3 was 0.9 (95% CI: 0.68–1.13). The criteria for the pre-specified equivalence assessments between the three groups were all met as the 95% CI for all respective GMC ratios fell within the range of 0.67–1.5 (Table 3).

For EBOV GP-specific neutralizing antibody responses, at day 78, 21 days post-MVA-BN-Filo administration, responses were observed in 98.8–100% of participants with geometric mean titers (GMTs) of 4751 50% inhibitory concentration (IC_50_) (95% CI: 3874–5826), 5498 IC_50_ (95% CI: 4386–6893), and 5051 IC_50_ (95% CI: 4005–6372) observed in Groups 1, 2, and 3, respectively (Table 4). At day 237, six months post-MVA-BN-Filo administration, neutralizing antibodies persisted in 70.7–79.3% of participants with GMTs of 448 IC_50_ (95% CI: 367–546), 425 IC_50_ (95% CI: 344–526), and 401 IC_50_ (95% CI: 329–488) in Groups 1, 2, and 3, respectively (Table 4). There were strong correlations between the neutralizing and binding antibody responses post-MVA-BN-Filo administration (Spearman correlation coefficient = 0.765 at 21 days post-MVA-BN-Filo; Spearman correlation coefficient = 0.805 at six months post-MVA-BN-Filo) (Supplementary Fig. 1).

**Table 4:** EBOV GP-specific neutralizing antibody responses (psVNA, IC_50_ titer): geometric means and responder rates; per protocol analysis set.

	Study 1: Group 1 [Leiden WVS]	Study 1: Group 2 [Bern WVS]	Study 1: Group 3 [Leiden MVS^a^]	Study 1: Group 4 [Placebo]	Study 2: Group 1 [Full clinical dose]	Study 2: Group 2 [Intermediate dose]	Study 2: Group 3 [Low dose]	Study 2: Group 4 [Placebo]
Day 1 (Baseline), *N* =	85	88	88	43	140	131	136	67
GMT, IC_50_ (95% CI)	<LLOQ (<LLOQ–<LLOQ)	<LLOQ (<LLOQ–<LLOQ)	<LLOQ (<LLOQ–<LLOQ)	<LLOQ (<LLOQ–<LLOQ)	<LLOQ (<LLOQ–<LLOQ)	<LLOQ (<LLOQ–<LLOQ)	<LLOQ (<LLOQ–<LLOQ)	<LLOQ (<LLOQ–<LLOQ)
Day 57 (56 days post-dose 1), *N* =	85	88	88	43	140	131	136	66
GMT, IC_50_ (95% CI)	197 (158–245)	202 (169–241)	195 (162–235)	<LLOQ	213 (188–242)	166 (146–188)	144 (128–162)	<LLOQ (<LLOQ–<LLOQ)
Responder, *n*/N^*^ (%)	33/85 (38.8)	32/88 (36.4)	35/88 (39.8)	0/43 (0.0)	60/140 (42.9)	38/131 (29.0)	33/136 (24.3)	0/66 (0.0)
(95% CI)	(28.4%–50.0%)	(26.4%–47.3%)	(29.5%–50.8%)	(0.0%–8.2%)	(34.5%–51.5%)	(21.4%–37.6%)	(17.3%–32.4%)	(0.0%–5.4%)
Day 78 (21 days post-dose 2), *N* =	81	87	86	41	135	123	130	66
GMT, IC_50_ (95% CI)	4751 (3874–5826)	5498 (4386–6893)	5051 (4005–6372)	<LLOQ	4906 (4217–5708)	3049 (2588–3592)	3842 (3237–4560)	<LLOQ (<LLOQ–<LLOQ)
Responder, *n*/N^*^ (%)	80/81 (98.8)	86/87 (98.9)	86/86 (100.0)	0/41 (0.0)	135/135 (100.0)	122/123 (99.2)	129/130 (99.2)	0/66 (0.0)
(95% CI)	(93.3%–>99.9%)	(93.8%–>99.9%)	(95.8%–100.0%)	(0.0%–8.6%)	(97.3%–100.0%)	(95.6%–>99.9%)	(95.8%–>99.9%)	(0.0%–5.4%)
Day 237 (180 days post-dose 2), *N* =	82	82	80	43	131	121	129	59
GMT, IC_50_ (95% CI)	448 (367–546)	425 (344–526)	401 (329–488)	<LLOQ	508 (441–586)	406 (344–480)	346 (297–403)	<LLOQ (<LLOQ–<LLOQ)
Responder, *n*/N^*^ (%)	65/82 (79.3)	58/82 (70.7)	62/80 (77.5)	0/43 (0.0)	111/131 (84.7)	91/121 (75.2)	88/129 (68.2)	0/59 (0.0)
(95% CI)	(68.9%–87.4%)	(59.6%–80.3%)	(66.8%–86.1%)	(0.0%–8.2%)	(77.4%–90.4%)	(66.5%–82.6%)	(59.4%–76.1%)	(0.0%–6.1%)

^a^Same Leiden MVS batch as used in phase 1/2 studies.

CI: confidence interval; EBOV: Ebola virus; GMT: geometric mean titer; GP: glycoprotein; IC_50_, 50% inhibitory concentration; LLOQ: lower limit of quantification; MVS: master virus seed; N: number of participants with data at that time point; N*: number of participants with data at baseline and at that time point; psVNA: pseudovirion neutralization assay; WVS: working virus seed.

### Immunogenicity: Study 2 (non-inferiority assessment of different vaccine regimen dose levels)

From this point onwards we refer to Ad26.ZEBOV 5 × 10^10^ viral particles (vp), MVA-BN-Filo 1 × 10^8^ infectious units (Inf.U) as ‘full clinical dose group’ (Group 1); to Ad26.ZEBOV 2 × 10^10^ vp, MVA-BN-Filo 5 × 10^7^ Inf.U as ‘intermediate-dose group’ (Group 2); and to Ad26.ZEBOV 8 × 10^9^ vp, MVA-BN-Filo 5 × 10^7^ Inf.U as ‘low-dose group’ (Group 3).

For EBOV GP-specific binding antibody responses in Study 2, the immunogenicity profiles appeared similar to those observed in Study 1 (Fig. 2; Table 2). At day 57, following Ad26.ZEBOV injection on day 1 (but prior to MVA-BN-Filo administration), GMCs of 793 EU/mL (95% CI: 698–902) were observed in the full clinical dose group, 669 EU/mL (95% CI: 571–784) in the intermediate-dose group, and 496 EU/mL (95% CI: 422–582) in the low-dose group (Fig. 2). At this time point, responder rates were similar between the full clinical dose (96.4%), intermediate-dose (96.9%), and low-dose groups (96.3%) (Table 2). At day 78, 21 days post-MVA-BN-Filo administration, 100% responders were observed in all groups, with a GMC of 11,054 EU/mL (95% CI: 9673–12,633) in the full clinical dose group, 7524 EU/mL (95% CI: 6472–8746) in the intermediate-dose group, and 8538 EU/mL (95% CI: 7338–9934) in the low-dose group. Geometric mean-fold increases of 14.2 (full clinical dose group), 10.9 (intermediate-dose group), and 16.5 (low-dose group) were observed when compared to pre-MVA-BN-Filo (day 57) concentrations. At day 237, six months post-MVA-BN-Filo administration, binding antibodies persisted in 98.3–98.5% of all participants with GMCs of 1263 EU/mL (95% CI: 1100–1450) in the full clinical dose group, 962 EU/mL (95% CI: 822–1125) in the intermediate-dose group, and 831 EU/mL (95% CI: 716–965) in the low-dose group (Table 2).

The primary objective of Study 2 was to demonstrate non-inferiority of the intermediate dose level versus the full clinical dose level of the regimen, based on the GMC ratio 21 days post-MVA-BN-Filo, using the pre-specified non-inferiority margin of ^2^/_3_ (0.67). At day 78, the GMC ratio of the intermediate-dose group versus the full clinical dose group was 0.7 (95% CI: 0.56–0.83) (Table 3). The pre-specified non-inferiority criterion of ^2^/_3_ (0.67) for the lower limit of the 95% CI was not met, hence, non-inferiority could not be demonstrated.

A pre-planned exploratory non-inferiority analysis was also performed 21 days post-MVA-BN-Filo administration, using a margin of ^1^/_2_ (0.5). In this analysis, the exploratory non-inferiority of ^1^/_2_ (0.5) was met for both the intermediate-dose and low-dose groups, albeit with >99% power based on the observed pooled standard deviation and sample size.

Post-hoc, an exploratory analysis assessing non-inferiority 56 days after Ad26.ZEBOV vaccination was added, using the criterion of ^2^/_3_ (0.67). At day 57, the GMC ratio of intermediate versus full clinical dose group was 0.8 (95% CI: 0.68–1.04), meeting the non-inferiority criterion. The GMC ratio of the low versus full clinical dose group was 0.6 (95% CI: 0.51–0.77), hence non-inferiority was not demonstrated for the low-dose group.

For EBOV GP-specific neutralizing antibody responses, at day 78, 21 days post-MVA-BN-Filo administration, responses were observed in 99–100% of participants with GMTs of 4906 IC_50_ (95% CI: 4217–5708) in the full clinical dose group, 3049 IC_50_ (95% CI: 2588–3592) in the intermediate-dose group, and 3842 IC_50_ (95% CI: 3237–4560) in the low-dose group (Table 4). At day 237, six months post-MVA-BN-Filo administration, neutralizing antibodies persisted in 84.7%, 75.2%, and 68.2% of participants in the full-, intermediate-, and low-dose groups, with GMTs of 508 IC_50_ (95% CI: 441–586), 406 IC_50_ (95% CI: 344–480), and 346 IC_50_ (95% CI: 297–403), respectively. There were strong correlations between the neutralizing and binding antibody responses post-MVA-BN-Filo administration (Spearman correlation coefficient = 0.829 at 21 days post-MVA-BN-Filo; Spearman correlation coefficient = 0.751 at six months post-MVA-BN-Filo) (Supplementary Fig. 1).

### Safety

In both studies, the vaccinations were generally well tolerated; 18 serious adverse events (SAEs) were reported in 11 vaccinees, including four deaths across both studies, none of which were considered to be related to the study vaccine administrations (Table 5). In Study 1, one vaccinee died on day 216 due to chronic prescription drug abuse, and a placebo recipient died at day 54 due to the toxic effects of benzodiazepines, cocaine, and opiates. In Study 2, two vaccinees died – one due to accidental fentanyl intoxication on day 12, and a second due to fatal gunshot wounds on day 17. Of the other 14 SAEs, 10 SAEs in Study 1 consisted of eight separate SAEs in one vaccinee between 18 and 103 days after MVA-BN-Filo due to treatment for peripheral arterial occlusive disease and a spontaneous abortion at day 62 (MVA-BN-Filo was not administered following the positive pregnancy test). Two placebo recipients had SAEs – a case of Bell’s palsy at day 16, and a pulmonary embolism, 97 days after the second injection. In Study 2, two SAEs were hospitalizations, one for a respiratory disorder 14 days after MVA-BN-Filo, and the second for a fractured humerus at day 170.

**Table 5:** Rates of unsolicited adverse events after each dose and serious adverse events over the whole study duration in the respective groups of the two studies, *n* (%).

	Study 1: Group 1 [Leiden WVS]	Study 1: Group 2 [Bern WVS]	Study 1: Group 3 [Leiden MVS^a^]	Study 1: Group 4 [Placebo]	Study 2: Group 1 [Full clinical dose]	Study 2: Group 2 [Intermediate dose]	Study 2: Group 3 [Low dose]	Study 2: Group 4 [Placebo]
Post-dose 1 (Ad26.ZEBOV or placebo), *N* =	94	94	94	47	150	150	150	75
Any unsolicited AE, *n* (%)	7 (7.4)	13 (13.8)	16 (17.0)	10 (21.3)	10 (6.7)	12 (8.0)	10 (6.7)	11 (14.7)
Severity grade 3	0	0	0	1 (2.1)	0	2 (1.3)	0	0
Post-dose 2 (MVA-BN-Filo or placebo), *N* =	88	91	91	45	145	143	144	72
Any unsolicited AE, *n* (%)	7 (8.0)	13 (14.3)	16 (17.6)	4 (8.9)	8 (5.5)	8 (5.6)	8 (5.6)	3 (4.2)
Severity grade 3	0	0	1 (1.1)	0	1 (0.7)	0	0	0
Whole study duration, *N* =	94	94	94	47	150	150	150	75
SAE, *n* (%)	1 (1.1)	2 (2.1)^b^	1 (1.1)	3 (6.4)^b^	1 (0.7)	2 (1.3)^c^	1 (0.7)	0
Related	0	0	0	0	0	0	0	0

^a^Same Leiden MVS batch as used in phase 1/2 studies.

^b^Includes one death due to drug overdose.

^c^Includes two deaths (gunshot wound and narcotic drug toxicity).

Unsolicited AEs were based on investigator assessment and were reported from signing of the informed consent form until 42 days post-dose 2; SAEs were reported from signing of the informed consent form until the end of the study (day 237).

AE: adverse event; MVS: master virus seed; *N*: number of participants with data at that time point; *n* (%): number (percentage) of participants with one or more events, where the denominator is the number of participants with available reactogenicity data after the given dose; SAE: serious adverse event; WVS: working virus seed.

In Study 1, solicited local reactions (Table 6) were reported following 52.1–62.8% of Ad26.ZEBOV doses and 46.2–54.9% of MVA-BN-Filo doses. The local reactions were reported following 25.5% of the first placebo injections and 4.4% of the second (Table 6). In Study 2, local reactions were reported after 24.7–52.0% of Ad26.ZEBOV doses and 6.7% of the first placebo injections, with the highest frequency after the highest dose of Ad26.ZEBOV (Group 1). After MVA-BN-Filo injection, local reactions were reported by 31.9–42.1% of vaccinees and 6.9% of placebo recipients. Almost all local reactions in both studies were reported as mild/moderate (Grades 1 or 2), transient, and consisted mainly of local injection pain (Table 6).

**Table 6:** Rates of solicited local adverse events after each dose in the respective groups of the two studies, *n* (%).

	Study 1: Group 1 [Leiden WVS]	Study 1: Group 2 [Bern WVS]	Study 1: Group 3 [Leiden MVS^a^]	Study 1: Group 4 [Placebo]	Study 2: Group 1 [Full clinical dose]	Study 2: Group 2 [Intermediate dose]	Study 2: Group 3 [Low dose]	Study 2: Group 4 [Placebo]
Post-dose 1 (Ad26.ZEBOV or placebo), *N* =	94	94	94	47	150	150	150	75
Any local AE, *n* (%)	59 (62.8)	49 (52.1)	59 (62.8)	12 (25.5)	78 (52.0)	61 (40.7)	37 (24.7)	5 (6.7)
Severity grade 1	45 (47.9)	40 (42.6)	47 (50.0)	9 (19.1)	65 (43.3)	57 (38.0)	32 (21.3)	4 (5.3)
Severity grade 2	14 (14.9)	8 (8.5)	11 (11.7)	3 (6.4)	12 (8.0)	4 (2.7)	5 (3.3)	1 (1.3)
Severity grade 3	0	1 (1.1)	1 (1.1)	0	1 (0.7)	0	0	0
Erythema	0	1 (1.1)	2 (2.1)	2 (4.3)	0	0	1 (0.7)	0
Pain	57 (60.6)	49 (52.1)	58 (61.7)	10 (21.3)	73 (48.7)	58 (38.7)	28 (18.7)	3 (4.0)
Pruritus	5 (5.3)	8 (8.5)	8 (8.5)	1 (2.1)	10 (6.7)	5 (3.3)	6 (4.0)	3 (4.0)
Swelling	16 (17.0)	8 (8.5)	5 (5.3)	2 (4.3)	13 (8.7)	4 (2.7)	10 (6.7)	1 (1.3)
Post-dose 2 (MVA-BN-Filo or placebo), *N* =	88	91	91	45	145	143	144	72
Any local AE, *n* (%)	45 (51.1)	42 (46.2)	50 (54.9)	2 (4.4)	61 (42.1)	49 (34.3)	46 (31.9)	5 (6.9)
Severity grade 1	35 (39.8)	33 (36.3)	40 (44.0)	2 (4.4)	52 (35.9)	45 (31.5)	40 (27.8)	5 (6.9)
Severity grade 2	8 (9.1)	8 (8.8)	10 (11.0)	0	9 (6.2)	2 (1.4)	6 (4.2)	0
Severity grade 3	2 (2.3)	1 (1.1)	0	0	0	2 (1.4)	0	0
Erythema	0	0	0	0	0	0	0	0
Pain	45 (51.1)	40 (44.0)	48 (52.7)	2 (4.4)	60 (41.4)	47 (32.9)	42 (29.2)	5 (6.9)
Pruritus	3 (3.4)	4 (4.4)	9 (9.9)	1 (2.2)	2 (1.4)	3 (2.1)	8 (5.6)	0
Swelling	7 (8.0)	5 (5.5)	9 (9.9)	1 (2.2)	6 (4.1)	6 (4.2)	5 (3.5)	1 (1.4)

Solicited AEs were based on participant-completed diary cards and were reported on the day of injection (post-injection) and for the following seven days.

^a^Same Leiden MVS batch as used in phase 1/2 studies.

AE: adverse event; MVS: master virus seed; *N*: number of participants with data at that time point; *n* (%): number (percentage) of participants with one or more events, where the denominator is the number of participants with available reactogenicity data after the given dose; WVS: working virus seed.

Solicited systemic AEs were reported by the majority of participants after the first vaccinations with Ad26.ZEBOV in Study 1 (70.2–77.7%), while the frequency of reports was lower in Study 2 (35.3–55.3%) after Ad26.ZEBOV vaccinations (Table 7). Rates in placebo groups (42.6% and 33.3% in placebo recipients in Studies 1 and 2, respectively) were lower than in Ad26.ZEBOV vaccine groups. These systemic AEs were mainly mild to moderate, with fatigue, headache, and myalgia being the most frequent. There was a similar profile of systemic AEs in both studies, except that arthralgia and chills were reported more frequently in Study 1 than Study 2. There was a trend for the lower dosages of Ad26.ZEBOV to be associated with lower systemic AE rates in Study 2, but rates in Study 1 were consistent across the three batches.

**Table 7:** Rates of solicited systemic adverse events after each dose in the respective groups of the two studies, *n* (%).

	Study 1: Group 1 [Leiden WVS]	Study 1: Group 2 [Bern WVS]	Study 1: Group 3 [Leiden MVS^a^]	Study 1: Group 4 [Placebo]	Study 2: Group 1 [Full clinical dose]	Study 2: Group 2 [Intermediate dose]	Study 2: Group 3 [Low dose]	Study 2: Group 4 [Placebo]
Post-dose 1 (Ad26.ZEBOV or placebo), *N* =	94	94	94	47	150	150	150	75
Any systemic AE, *n* (%)	73 (77.7)	66 (70.2)	73 (77.7)	20 (42.6)	83 (55.3)	74 (49.3)	53 (35.3)	25 (33.3)
Severity grade 1	40 (42.6)	32 (34.0)	43 (45.7)	18 (38.3)	52 (34.7)	53 (35.3)	45 (30.0)	20 (26.7)
Severity grade 2	29 (30.9)	29 (30.9)	26 (27.7)	2 (4.3)	26 (17.3)	19 (12.7)	6 (4.0)	4 (5.3)
Severity grade 3	4 (4.3)	5 (5.3)	4 (4.3)	0	5 (3.3)	2 (1.3)	2 (1.3)	1 (1.3)
Arthralgia	33 (35.1)	23 (24.5)	29 (30.9)	1 (2.1)	30 (20.0)	17 (11.3)	9 (6.0)	2 (2.7)
Chills	39 (41.5)	27 (28.7)	32 (34.0)	1 (2.1)	31 (20.7)	12 (8.0)	6 (4.0)	2 (2.7)
Fatigue	50 (53.2)	47 (50.0)	55 (58.5)	14 (29.8)	60 (40.0)	51 (34.0)	25 (16.7)	15 (20.0)
Headache	48 (51.1)	39 (41.5)	56 (59.6)	10 (21.3)	49 (32.7)	39 (26.0)	26 (17.3)	9 (12.0)
Myalgia	50 (53.2)	48 (51.1)	47 (50.0)	7 (14.9)	45 (30.0)	31 (20.7)	25 (16.7)	5 (6.7)
Nausea	18 (19.1)	16 (17.0)	18 (19.1)	5 (10.6)	8 (5.3)	13 (8.7)	5 (3.3)	5 (6.7)
Pyrexia	12 (12.8)	14 (14.9)	13 (13.8)	0	8 (5.3)	4 (2.7)	4 (2.7)	3 (4.0)
Post-dose 2 (MVA-BN-Filo or placebo), *N* =	88	91	91	45	145	143	144	72
Any systemic AE, *n* (%)	40 (45.5)	35 (38.5)	45 (49.5)	11 (24.4)	43 (29.7)	46 (32.2)	56 (38.9)	16 (22.2)
Severity grade 1	27 (30.7)	25 (27.5)	30 (33.0)	10 (22.2)	33 (22.8)	36 (25.2)	44 (30.6)	10 (13.9)
Severity grade 2	11 (12.5)	9 (9.9)	12 (13.2)	1 (2.2)	9 (6.2)	7 (4.9)	9 (6.3)	5 (6.9)
Severity grade 3	2 (2.3)	1 (1.1)	3 (3.3)	0	1 (0.7)	3 (2.1)	3 (2.1)	1 (1.4)
Arthralgia	12 (13.6)	7 (7.7)	11 (12.1)	0	4 (2.8)	9 (6.3)	11 (7.6)	1 (1.4)
Chills	7 (8.0)	5 (5.5)	8 (8.8)	0	3 (2.1)	7 (4.9)	3 (2.1)	4 (5.6)
Fatigue	18 (20.5)	20 (22.0)	27 (29.7)	3 (6.7)	18 (12.4)	24 (16.8)	21 (14.6)	11 (15.3)
Headache	18 (20.5)	16 (17.6)	20 (22.0)	5 (11.1)	13 (9.0)	19 (13.3)	16 (11.1)	7 (9.7)
Myalgia	29 (33.0)	25 (27.5)	30 (33.0)	0	23 (15.9)	23 (16.1)	31 (21.5)	5 (6.9)
Nausea	4 (4.5)	3 (3.3)	4 (4.4)	3 (6.7)	5 (3.4)	8 (5.6)	2 (1.4)	4 (5.6)
Pyrexia	3 (3.4)	3 (3.3)	7 (7.7)	4 (8.9)	5 (3.4)	5 (3.5)	5 (3.5)	5 (6.9)

Solicited AEs were based on participant-completed diary cards and were reported on the day of injection (post-injection) and for the following seven days.

^a^Same Leiden MVS batch as used in phase 1/2 studies.

AE: adverse event; MVS: master virus seed; *N*: number of participants with data at that time point; *n* (%): number (percentage) of participants with one or more events, where the denominator is the number of participants with available reactogenicity data after the given dose; WVS: working virus seed.

Rates of solicited systemic AEs after the second vaccination with MVA-BN-Filo were similar in both Study 1 (38.5–49.5%) and Study 2 (29.7–38.9%), and were higher than placebo in both studies (24.4% and 22.2%, respectively). Fatigue, headache, and myalgia were the most frequently reported systemic AEs and were mainly mild to moderate in intensity with relatively few severe AEs (Table 7).

In both studies, unsolicited AEs were reported at low rates and most were mild and unrelated to the study procedures as judged by the investigator (Table 5). Unsolicited AEs mainly consisted of infections, or respiratory or nervous system disorders unrelated to vaccination, with no trends associated with different dosages or batches of vaccines. Rates in placebo recipients were higher than in vaccine groups after the first injections, but similar after the second injections.

## Discussion

Clinical studies that initially evaluated the heterologous two-dose Ad26.ZEBOV, MVA-BN-Filo vaccination regimen were all designed at the time of the 2014–2016 West African outbreak as part of an accelerated development plan intended to address the urgent medical need^16–21^. In that context, all phases of clinical development (including four phase 1^22–25^, two phase 2^26,27^, and three phase 3^28–30^ studies) were conducted simultaneously, and in parallel, with increasing the production capacity. Two phase 3 clinical studies were conducted in the USA to support the manufacturing specifications, both with USA populations. As the 2014–2016 outbreak highlighted the need to increase the manufacturing capacity, changes were made to the manufacturing process of Ad26.ZEBOV.

Hence, Study 1 was designed to bridge Ad26.ZEBOV produced from a 1-tiered virus seed, based on MVS in Leiden, the Netherlands, to a 2-tiered seed approach, using WVS, as well as the change in manufacturing site to Bern, Switzerland. The latter manufacturing process will be used for the commercial drug substance. Since the evaluation of immunological equivalence focused on Ad26.ZEBOV, the primary comparison was performed on the EBOV GP binding antibody responses after the first vaccination. The equivalence limits on the 95% CI for the GMC ratios were set narrowly as ^2^/_3_ (0.67) to 1^1^/_2_ (1.5). When this study was designed at the time of the 2014–2016 outbreak, very little was known about the intrinsic variation in binding antibody concentrations between study populations as measured by ELISA. The assumed variation in binding antibody concentrations within the study population was based on a single phase 1 clinical study^16^. In retrospect, higher standard deviations were observed in two subsequent phase 1 studies^17,18^ and also in Study 1. It appeared that Study 1 was substantially underpowered to conclude on the primary objective, i.e. 42% as compared to the planned 83%. Although 56 days after Ad26.ZEBOV administration the GMCs were similar between the Ad26.ZEBOV batch manufactured in Bern from WVS (745 EU/mL; 95% CI: 603–921) and the batch manufactured in Leiden from MVS (851 EU/mL; 95% CI: 720–1006), the primary objective was not met. The GMC ratio was 0.9 (95% CI: 0.65–1.17) and the lower limit of the 95% CI was just outside the lower limit of the equivalence criterion of 0.67. While a formal conclusion cannot be drawn about the other comparisons evaluated as a secondary objective, the equivalence criteria were met for WVS batch Bern and WVS batch Leiden, and for WVS batch Leiden and MVS batch Leiden. Since the clinically relevant time point to assess the function of the priming vaccine in a two-dose heterologous regimen is after the second vaccination, an additional pre-planned equivalence assessment was performed at 21 days post-MVA-BN-Filo using the same criteria. After completion of the full regimen, the equivalence criteria for all three comparisons were met. Extensive physicochemical comparability assessments indicated that the final Ad26.ZEBOV products from the three manufacturing processes are comparable and Study 1 confirms these physicochemical comparability results, providing assurance that the vaccine and manufacturing process are consistent.

In addition to supporting the change in both manufacturing process and facility to increase the manufacturing capacity, information was needed regarding how the dose level of the Ad26.ZEBOV and MVA-BN-Filo batches impacts the immunogenicity of the two-dose vaccination regimen. Hence, Study 2 was designed to compare the immunogenicity of two different dose levels, an intermediate dose level (Ad26.ZEBOV: 2 × 10^10^ vp, MVA-BN-Filo: 5 × 10^7^ Inf.U) and low dose level (8 × 10^9^ vp, 5 × 10^7^ Inf.U) against the full clinical dose (5 × 10^10^ vp, 1 × 10^8^ Inf.U). After completion of the two-dose vaccination regimen, the responder rate was 100% for all three dose levels. However, a dose level-dependent antibody response was observed: the highest GMC was observed in the full clinical dose group (11,054 EU/mL; 95% CI: 9673–12,633) versus intermediate (7524 EU/mL; 95% CI: 6472–8746) and low (8538 EU/mL; 95% CI: 7338–9934) dose level groups. While the pre-specified non-inferiority criterion of ^2^/_3_ (0.67) for the lower limit of the 95% CI around the GMC ratio was not met and non-inferiority could not be concluded for the intermediate dose level (Group 2) compared to the full clinical dose level (Group 1), non-inferiority was met for both the intermediate and low dose levels with the additional pre-planned exploratory non-inferiority limit of ^1^/_2_ (0.5). This criterion was also selected and FDA endorsed for the currently ongoing Ad26.ZEBOV, MVA-BN-Filo phase 3 lot-to-lot study (USA; NCT0422878)^30^, primarily based on the population variability as measured by Filovirus Animal Non-Clinical Group (FANG) ELISA in the completed phase 1 studies. In addition, a margin of ^1^/_2_ (0.5) is expected to be adequate to detect potentially clinically meaningful differences in GMCs between groups. However, using the ^1^/_2_ (0.5) margin, the power of Study 2 would have been more than 99% with the current sample size. Hence, while there are some statistical limitations to this evaluation, an important conclusion is that the potencies of the Ad26.ZEBOV and MVA-BN-Filo vaccine batches appear to have an impact on the immunogenicity of the two-dose vaccination regimen, yet the observed differences in GMC between the groups after completion of the two-dose regimen are relatively small – it remains to be determined whether these differences would be clinically meaningful.

Overall, the safety and the humoral immunogenicity data observed in Study 1 and Study 2 were similar for the same vaccine dose levels, which was to be expected considering that both study populations were from the same areas in the USA and the same immunological assays were used. These data are in line with previously reported studies that evaluated the Ad26.ZEBOV, MVA-BN-Filo vaccine regimen in Europe and Africa^16–21^ and contributed to the indication of the two-dose heterologous Ad26.ZEBOV, MVA-BN-Filo vaccine regimen, authorized under exceptional circumstances by the European Union^12–14^.

## Methods

We report on two randomized, double-blind, placebo-controlled phase 3 studies of the heterologous two-dose vaccine regimen where Ad26.ZEBOV is followed by MVA-BN-Filo 56 days later. Both studies were performed under the supervision of the same coordinating investigator in multiple sites in the USA; Study 1 (Mishawaka, Indiana; Rockville, Maryland; San Diego, California) and Study 2 (Huntsville, Alabama; Melbourne, Florida; Peoria, Illinois; Rockville, Maryland). The protocol for each study was approved by a central institutional review board (MaGil IRB, Rockville, Maryland, USA), registered with ClinicalTrials.gov (Study 1, NCT02543268; Study 2, NCT02543567), and performed according to Declaration of Helsinki and Good Clinical Practice guidelines, as well as local regulations. The protocols for each study have been uploaded to the Nature Research Protocol Exchange. All participants provided written informed consent prior to enrollment.

### Participants

Eligible participants were adults of either sex, aged from 18 to 50 years, who were in good health in the opinion of the investigator at the screening visit based on a medical examination, medical history, and clinical laboratory assessments, and were free of any acute infection or fever on the day of vaccination. Main exclusion criteria included any known exposure to Ebola disease, prior receipt of any Ebola vaccine or Ad26- or MVA-based vaccine, or any other investigational vaccine within 3 months of screening, any known allergy to vaccine components, seropositivity for hepatitis B, hepatitis C, or HIV, recent receipt of blood products, or any chronic medical condition that could influence the protocol-specified assessments. Women of childbearing potential were required to have a negative pregnancy test at screening and before each vaccination, and to practice an approved method of birth control from 28 days before vaccination until the end of the study.

### Study designs

In both studies, participants were randomized (2:2:2:1) at enrollment to one of four groups using a computer-generated schedule (via an Interactive Web Response System) provided by the sponsor, balanced using randomly permuted blocks, and stratified by site. All participants received an intramuscular injection of vaccine or placebo in the deltoid muscle according to their group allocation on days 1 and 57 (Table 8). The second injection was given in the opposite arm to the first. Compositions of the different vaccines are shown in Table 8, the first injection being Ad26.ZEBOV and the second MVA-BN-Filo; placebo was 0.5 mL 0.9% saline. All vaccinations were administered by study personnel blinded to vaccine or placebo, or batch being used; masking tape was used to cover the dispensing syringes containing the treatment allocated.

**Table 8:** Vaccines administered in the different study groups.

	Study 1: Group 1 [Leiden WVS]	Study 1: Group 2 [Bern WVS]	Study 1: Group 3 [Leiden MVS^a^]	Study 1: Group 4 [Placebo]	Study 2: Group 1 [Full clinical dose]	Study 2: Group 2 [Intermediate dose]	Study 2: Group 3 [Low dose]	Study 2: Group 4 [Placebo]
*N* =	94	94	94	47	150	150	150	75
Ad26.ZEBOV								
Dose (vp)	5 × 10^10^	5 × 10^10^	5 × 10^10^	—	5 × 10^10^	2 × 10^10^	8 × 10^9^	—
Batch	#33831	#33488	#32642	—	#33488	#33488	#33488	—
MVA-BN-Filo								
Dose (Inf.U)	1 × 10^8^	1 × 10^8^	1 × 10^8^	—	1 × 10^8^	5 × 10^7^	5 × 10^7^	—
Batch	#32791	#32791	#32791		#32794	#32794	#32794	—

^a^Same Leiden MVS batch as used in phase 1/2 studies.

Inf.U: infectious units; MVS: master virus seed; vp: viral particles; WVS: working virus seed.

In Study 1 the primary objective was to demonstrate equivalence of EBOV GP binding antibody responses measured by FANG ELISA at 56 days post-Ad26.ZEBOV in groups whose participants were administered with the vaccine batch produced with the WVS in the commercial process (Group 2) and the vaccine batch from the MVS used in phase 2 studies (Group 3). Equivalence was considered to have been met if the 95% CI of the estimated GMC ratio was entirely within the predefined range of ^2^/_3_ (0.67) to 1^1^/_2_ (1.5). The GMC ratio and its 95% CI was determined by computing the difference between the log_10_-transformed ELISA concentrations (EU/mL) between groups, and back-transforming the estimated difference and its 95% CI. Secondary objectives were to demonstrate: (1) equivalence of Ad26.ZEBOV batches manufactured in Leiden from WVS (Group 1) and MVS (Group 3) at 56 days post-Ad26.ZEBOV; (2) equivalence of the Ad26.ZEBOV batch manufactured in Leiden from WVS (Group 1) and the batch manufactured in Bern from WVS (Group 2) at 56 days post-Ad26.ZEBOV; (3) equivalence of 3 different Ad26.ZEBOV batches administered as dose 1 followed by a single dose of MVA-BN-Filo 56 days later, at the 21 days post-dose 2 time point, using the same equivalence margin.

The primary objective of Study 2 was to demonstrate non-inferiority of the intermediate-dose level to the full clinical dose, based on the GMCs of EBOV GP binding antibodies measured by FANG ELISA at 21 days post- MVA-BN-Filo (day 78), using a predefined non-inferiority margin of ^2^/_3_ (0.67). If the primary objective would be met, non-inferiority of the low-dose level to the full clinical dose would be evaluated in the same way (hierarchical testing). Additionally, a pre-planned exploratory non-inferiority analysis was performed at 21 days post-MVA-BN-Filo, using a margin of ½ (0.5). This non-inferiority criterion was used in an ongoing phase 3 lot-to-lot study assessing consistency of Ad26.ZEBOV and MVA-BN-Filo manufacturing and was applied here for consistency^31^. A post-hoc exploratory analysis was performed at 56 days post-Ad26.ZEBOV, using a non-inferiority margin of ^2^/_3_ (0.67), as per regulatory authority request.

For each pair-wise comparison, estimated differences were expressed as ratios of GMCs with 95% CI, determined from comparing the log_10_-transformed ELISA concentrations (EU/mL) between groups and back-transformation of the estimated difference and corresponding 95% CI. Non-inferiority was to be demonstrated if the 95% CI of the estimated GMC ratio was entirely above the non-inferiority margin.

### Vaccines

Ad26.ZEBOV is a monovalent, recombinant, replication-incompetent Ad26-based vector that encodes the full-length EBOV Mayinga GP. MVA-BN-Filo (Bavarian Nordic) is a recombinant, non-replicating, modified vaccinia Ankara-vectored vaccine encoding EBOV Mayinga, Sudan virus Gulu, and Marburg virus Musoke variant GPs, as well as Taï Forest virus nucleoprotein.

In Study 1, three groups received three different batches of Ad26.ZEBOV (Table 8). Group 1 received batch #33831, manufactured in Leiden, the Netherlands, from WVS; Group 2 received batch #33488 (also used in Study 2), manufactured using WVS in the commercial manufacturing facility in Bern, Switzerland; Group 3 received Ad26.ZEBOV batch #32642, manufactured in the facility in Leiden using the MVS and used in all previously published phase 1 and 2 studies^16–19,32^.

The manufacturing processes of the MVS and WVS, ranging from bioreactor infection to seed harvesting, are identical. The difference in MVS runs versus WVS runs is that MVS runs are infected using a pre-MVS seed, which is a non-Good Manufacturing Practice (GMP) development seed. For WVS runs, the MVS is used to infect the bioreactor after release. Therefore, the manufacturing process for batches of the Ad26.ZEBOV vaccine substance produced by MVS or by WVS differs only in the origin of the seed.

Each Ad26.ZEBOV batch was supplied as a frozen liquid suspension to be thawed before use at a concentration of 1 × 10^11^ vp/mL in 2 mL single-use glass vials, with an extractable volume of 0.5 mL per vial (Table 8). Each 0.5 mL dose of MVA-BN-Filo was supplied as a frozen liquid suspension to be thawed before use at a concentration of 2 × 10^8^ Inf.U/mL.

In Study 2, Ad26.ZEBOV (batch #33488) was supplied as a frozen liquid suspension to be thawed before use in single doses at a measured concentration of 8 × 10^10^ vp/mL in 2 mL single-use glass vials. Unblinded qualified pharmacy personnel prepared three dilutions to give 5 × 10^10^ vp/mL (full clinical dose), 2 × 10^10^ vp/mL (intermediate dose), and 8 × 10^9^ vp/mL (low dose) per 0.5 mL dose (Table 8). MVA-BN-Filo was manufactured in a manufacturing facility in Kvistgård, Denmark and supplied as a frozen liquid suspension to be thawed before use at a concentration of 2 × 10^8^ Inf.U per mL in 2-mL single-use glass vials. The pharmacy personnel prepared two dilutions of MVA-BN-Filo to give 1 × 10^8^ Inf.U/mL (full clinical dose) or 5 × 10^7^ Inf.U/mL (intermediate and low dose level) per dose (Table 8).

### Immunogenicity assessments

Four serum samples for assessment of immune responses were obtained: immediately before the first vaccination on day 1; before the second vaccination on day 57; 21 days after the second vaccination on day 78; and 6 months after the second vaccination on day 237.

EBOV GP-specific total immunoglobulin G (IgG) binding antibody concentrations were measured by FANG ELISA at Q^2^ Solutions (San Juan Capistrano, CA, USA)^19,33^ and were summarized as group GMCs of EU/mL with 95% CIs. In brief, serially diluted serum samples were added to EBOV GP coated on 96-well microtiter plates at a standard starting dilution of 1:50 up to a dilution of 1:1600 (2-fold dilutions). EBOV GP-specific antibodies were detected with a goat anti-human IgG antibody conjugated with horseradish peroxidase (HRP) followed by a colorimetric reaction (tetramethylbenzidine [TMB] substrate). Each plate contained a negative control sample and serial dilutions (as described above) of a low and a high control sample to guarantee assay validity. The GMC of the 6 sample dilution points was calculated using a reference curve generated from high titer human sera to obtain the reportable value in EU/mL. A FANG ELISA result (EU/mL) was considered positive if the value was above the assay lower limit of quantification (LLOQ; 36.11 EU/mL). Values below the LLOQ were imputed with LLOQ/2. Responder rates were determined as the percentage of participants in each group with post-vaccination concentrations >2.5-fold the LLOQ, i.e. 36.11 EU/mL, in baseline seronegative individuals, or >2.5-fold the baseline value in pre-vaccination seropositive participants.

EBOV GP-specific neutralizing antibody titers were measured with a pseudovirion neutralization assay (psVNA) at Monogram (San Francisco, CA, USA) and summarized as group GMTs of IC_50_ with 95% CIs^19^. A psVNA result (IC_50_ titer) was considered positive if the IC_50_ titer was more than three times amphotropic murine leukemia virus (aMLV) and above the assay-specific LLOQ of 120 IC_50_ titer. Values that were less than three times aMLV or below the LLOQ were imputed with LLOQ/2. For psVNA, a participant was classed as a responder at a considered time point if the sample interpretation was negative at baseline and positive post-baseline and the post-baseline value was greater than twice the LLOQ, or if sample interpretation was positive both at baseline and post-baseline and there was a greater than two-fold increase from baseline. Spearman correlation coefficients were calculated for EBOV GP-specific binding antibody concentrations (FANG ELISA) and psVNA titers at 21 days and six months post-MVA-BN-Filo.

### Safety assessments

Participants were assessed at 30 and 60 min after each vaccination for any immediate AEs. Participants were supplied with diary cards to report solicited local and systemic AEs and daily body temperature for 7 days after each vaccination, which were graded as mild (Grade 1), moderate (Grade 2), or severe (Grade 3) by the participant. After seven days, participants continued to record any other AEs as “unsolicited AEs” until 42 days after the second vaccination. SAEs were to be reported to the investigators at any time up to six months after dose 2 (day 237). A data review committee was commissioned to assess safety data during the study; this committee would convene to review the available safety data in the case that a pausing rule was met and for any single events that were considered to put at risk the safety of the participants.

### Statistics

In Study 1, the sample size was calculated assuming a 5% type I error rate, a standard deviation of 0.323 for log_10_-transformed binding antibodies 56 days after Ad26.ZEBOV and a 10% difference in GMCs between batches. With 94 participants, the power was 83% to conclude equivalence between batches with margins of ^2^/_3_ (0.67) and 1^1^/_2_ (1.5).

The sample size for Study 2 was based on the assumption that the standard deviation for log_10_-transformed binding antibodies was 0.303 at 21 days post-MVA-BN-Filo, based on data from a phase 1 study^16^, and GMCs for intermediate and low dose levels would be at least 90% of the full clinical dose. For 90% power, including a 10% drop-out rate, a total of 150 participants were needed per group.

All immunogenicity analyses were based on the per protocol analysis set, which included all randomized and vaccinated participants who received both Ad26.ZEBOV (dose 1) and MVA-BN-Filo (dose 2) vaccinations within the protocol-defined window, who had at least one post-vaccination evaluable immunogenicity blood sample, and who had no major protocol deviations that could influence the immune response.

No formal statistical testing of the safety data was planned or performed in either study. Analysis of solicited and unsolicited AEs was based on all participants included in the Full Analysis Set (i.e. all participants who were randomized and received at least one dose of vaccine or placebo), with descriptive summaries.

### Reporting summary

Further information on research design is available in the Nature Research Reporting Summary linked to this article.

## Supplementary information

Supplementary Information

Reporting Summary

## Data Availability

Janssen has an agreement with the Yale Open Data Access (YODA) Project to serve as the independent review panel for evaluation of requests for CSRs and participant level data from investigators and physicians for scientific research that will advance medical knowledge and public health. Data will be made available following publication and approval by YODA of any formal requests with a defined analysis plan. For more information on this process or to make a request, please visit The Yoda Project site at http://yoda.yale.edu. The data sharing policy of Janssen Pharmaceutical Companies of Johnson & Johnson is available at https://www.janssen.com/clinical-trials/transparency.
