# Supplementary material for: Assessments of different batches and dose levels of a two-dose Ad26.ZEBOV and MVA-BN-Filo vaccine regimen
Source: NPJ Vaccines. 2021 Dec 20;6:157. doi: 10.1038/s41541-021-00402-8 (PMC8688528; doi:10.1038/s41541-021-00402-8)
Supplement: Supplementary file 1 — Supplementary Information [file 41541_2021_402_MOESM1_ESM.pdf]

Supplementary Information

Supplementary Fig. 1. Correlations between the neutralizing and binding antibody responses post-dose 2 (MVA-BN-Filo) administration for Study 1 (**a**) and Study 2 (**b**)

a)

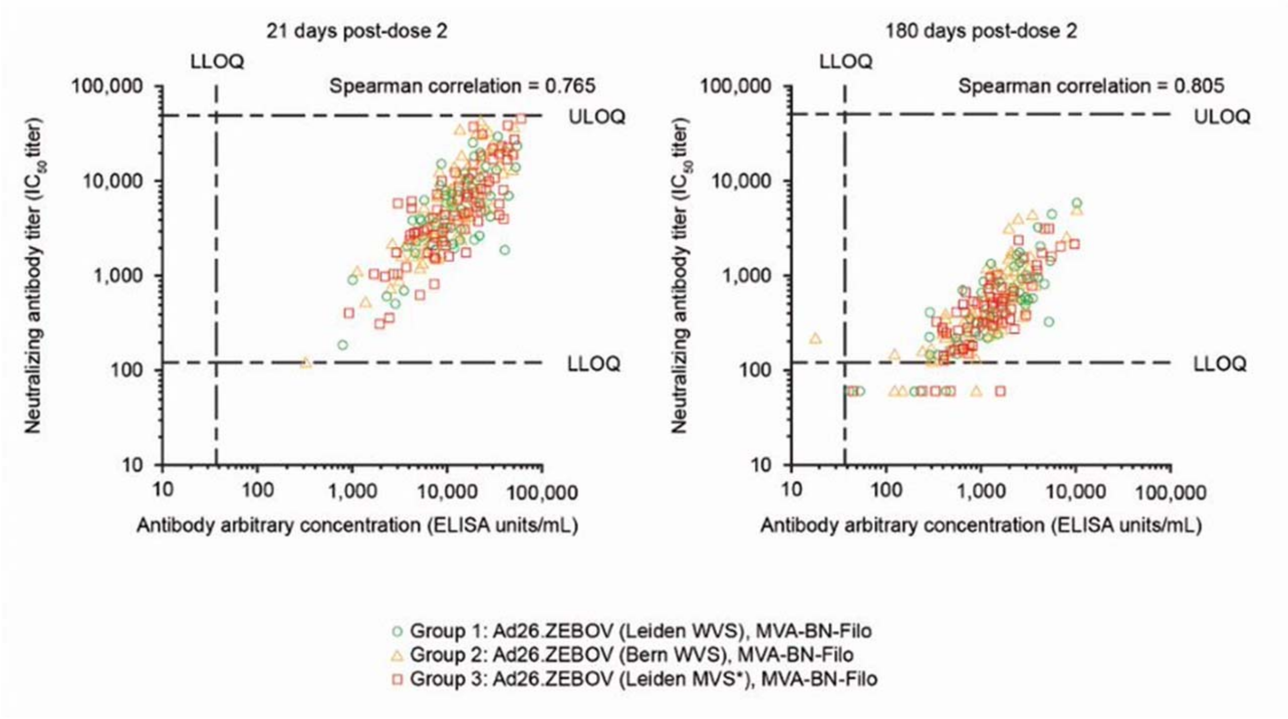

b)

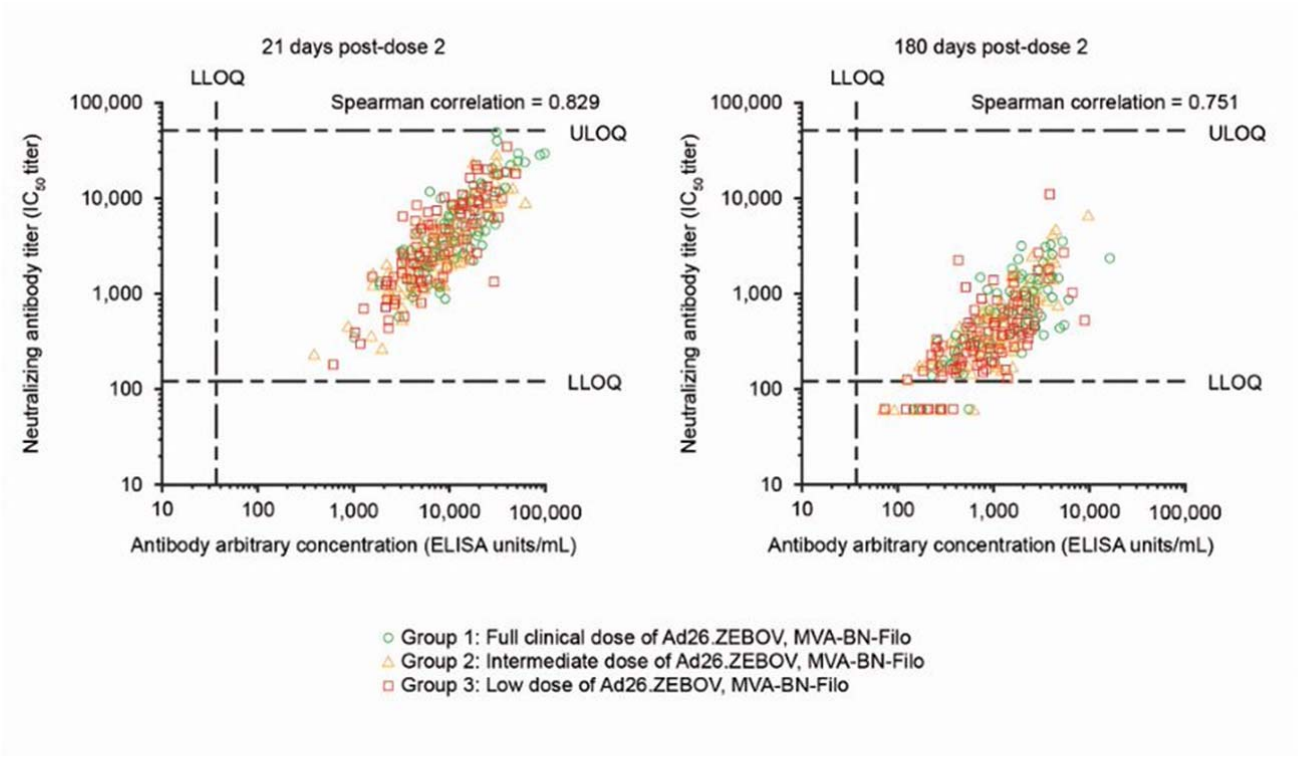

LLOQ: lower limit of quantification; MVS: master virus seed; ULOQ: upper limit of quantification; WVS: working virus seed.

Janssen Vaccines & Prevention B.V.*

Clinical Protocol

A Phase 3, Randomized, Double-blind, Placebo-controlled Study to Evaluate A Range of Dose Levels of a Heterologous Prime-boost Regimen of Ad26.ZEBOV and MVA-BN®-Filo in Healthy Adult Subjects

Protocol VAC52150EBL3002; Phase 3
AMENDMENT 2

IND Number: 16280

VAC52150 (Ad26.ZEBOV/MVA-BN-Filo [MVA-mBN226B])

*Janssen Vaccines & Prevention B.V. (formerly known as Crucell Holland B.V.) is a Janssen pharmaceutical company of Johnson & Johnson and is hereafter referred to as the sponsor of the study. The sponsor is identified on the Contact Information page that accompanies the protocol.

Status: Approved
Date: 1 September 2016
EDMS number: EDMS-ERI-106271717, 6.0

GCP Compliance: This study will be conducted in compliance with Good Clinical Practice, and applicable regulatory requirements.

Confidentiality Statement
The information in this document contains trade secrets and commercial information that are privileged or confidential and may not be disclosed unless such disclosure is required by applicable law or regulations. In any event, persons to whom the information is disclosed must be informed that the information is privileged or confidential and may not be further disclosed by them. These restrictions on disclosure will apply equally to all future information supplied to you that is indicated as privileged or confidential.

**Protocol History VAC52150EBL3002_Protocol**		
Document Type and *File Name*	Issued Date	Comments
Initial Clinical Protocol *VAC52150EBL3002_Protocol*	30 July 2015	-
Protocol Amendment 1 *VAC52150EBL3002_Protocol_Amend_1*	17 November 2015	For details, see Section Amendment_1
Protocol Amendment 2 *VAC52150EBL3002_Protocol_Amend_2*	This document	For details, see Section Amendment_2

TABLE OF CONTENTS

TABLE OF CONTENTS 3

LIST OF ATTACHMENTS 5

LIST OF IN-TEXT TABLES 5

PROTOCOL AMENDMENT 6

SYNOPSIS 11

TIME AND EVENTS SCHEDULE 18

ABBREVIATIONS 20

DEFINITIONS OF TERMS 21

1. INTRODUCTION 22
1.1. Background 22
1.1.1. Nonclinical Studies 22
1.1.2. Clinical Studies 23
1.1.2.1. Safety Profile of Ad26-based Vaccines 23
1.1.2.2. Safety Profile of MVA-BN-based Vaccines 26
1.1.2.3. Relevant Safety Information From Ongoing VAC52150 Studies 27
1.2. Benefit/Risk Section 27
1.2.1. Known Benefits 27
1.2.2. Potential Benefits 27
1.2.3. Known Risks 27
1.2.4. Potential Risks 28
1.2.5. Overall Benefit/Risk Assessment 29
1.3. Overall Rationale for the Study 30

2. OBJECTIVES, ENDPOINTS AND HYPOTHESIS 32
2.1. Objectives 32
2.2. Endpoints 33
2.3. Hypothesis 33

3. STUDY DESIGN AND RATIONALE 34
3.1. Overview of Study Design 34
3.2. Study Design Rationale 37

4. SUBJECT POPULATION 38
4.1. Inclusion Criteria 38
4.2. Exclusion Criteria 40
4.3. Prohibitions and Restrictions 42

5. STUDY VACCINE ALLOCATION AND BLINDING 43

6. DOSAGE AND ADMINISTRATION 44
6.1. General Instructions and Procedures 44
6.2. Criteria for Postponement of Vaccination 45
6.3. Contraindications to Boost Vaccination 46

7. STUDY VACCINE COMPLIANCE 46

8. PRESTUDY AND CONCOMITANT THERAPY 46

9. STUDY EVALUATIONS 47

9.1. Study Procedures 47
9.1.1. Overview 47
9.1.2. Screening Phase 48
9.1.3. Study Visits 49
9.1.4. VAC52150 Vaccine Development Roll-over Study 52
9.2. Immunogenicity Evaluations 52
9.3. Safety Evaluations 53
9.3.1. Pausing Rules 56
9.4. Vaccine-induced Seropositivity 57
9.5. Sample Collection and Handling 58

10. SUBJECT COMPLETION/DISCONTINUATION OF STUDY VACCINATION/WITHDRAWAL FROM THE STUDY 58
10.1. Completion 58
10.2. Discontinuation of Study Vaccination/Withdrawal From the Study 58
10.3. Withdrawal From the Use of Research Samples 59

11. STATISTICAL METHODS 60
11.1. Analysis Sets 60
11.2. Sample Size Determination 61
11.3. Subject Information 61
11.4. Immunogenicity Analyses 62
11.5. Safety Analyses 62
11.6. Interim Analysis 63
11.7. Data Review Committee 63

12. ADVERSE EVENT REPORTING 63
12.1. Definitions 64
12.1.1. Adverse Event Definitions and Classifications 64
12.1.2. Attribution Definitions 66
12.1.3. Severity Criteria 66
12.2. Special Reporting Situations 67
12.3. Procedures 67
12.3.1. All Adverse Events 67
12.3.2. Serious Adverse Events 68
12.3.3. Immediate Reportable Events 69
12.3.4. Pregnancy 69
12.4. Contacting Sponsor Regarding Safety 70

13. PRODUCT QUALITY COMPLAINT HANDLING 70
13.1. Procedures 70
13.2. Contacting Sponsor Regarding Product Quality 70

14. STUDY VACCINE INFORMATION 71
14.1. Description of Study Vaccines 71
14.2. Packaging and Labeling 71
14.3. Preparation, Handling, and Storage 71
14.4. Study Vaccine Accountability 72

15. STUDY-SPECIFIC MATERIALS 73

16. ETHICAL ASPECTS 73
16.1. Study-specific Design Considerations 73
16.2. Regulatory Ethics Compliance 73
16.2.1. Investigator Responsibilities 73
16.2.2. Independent Ethics Committee or Institutional Review Board 74
16.2.3. Informed Consent 75
16.2.4. Privacy of Personal Data 76
16.2.5. Long-term Retention of Samples for Additional Future Research 76

16.2.6. Country Selection 77

17. ADMINISTRATIVE REQUIREMENTS 77
17.1. Protocol Amendments 77
17.2. Regulatory Documentation 77
17.2.1. Regulatory Approval/Notification 77
17.2.2. Required Prestudy Documentation 78
17.3. Subject Identification, Enrollment, and Screening Logs 78
17.4. Source Documentation 79
17.5. Case Report Form Completion 79
17.6. Data Quality Assurance/Quality Control 80
17.7. Record Retention 80
17.8. Monitoring 81
17.9. Study Completion/Termination 81
17.9.1. Study Completion/End of Study 81
17.9.2. Study Termination 81
17.10. On-site Audits 82
17.11. Use of Information and Publication 82

REFERENCES 84

ATTACHMENTS 86

INVESTIGATOR AGREEMENT 92

LAST PAGE 92

LIST OF ATTACHMENTS

Attachment 1: Toxicity Tables for Use in Trials Enrolling Healthy Adults 86

LIST OF IN-TEXT TABLES

TABLES

Table 1: Schematic Overview of Study Design and Groups 35
Table 2: Visit Windows 48
Table 3: Overview of Immunogenicity Assessments 53

PROTOCOL AMENDMENT

Amendment_2 (this document)

The overall reason for the amendment: This amendment was issued to add wording on "Immediate Reportable Events" after observation of a neurological event (Miller Fisher syndrome) in a study participant after receipt of MVA-BN-Filo or placebo in study VAC52150EBL2001. Based on the request of the Agence Nationale de Sécurité du Médicament et des produits de santé (ANSM) for study VAC52150EBL2001, the sponsor has decided to implement the collection of neurologic and autoimmune events ("Immediate Reportable Events") throughout the entire clinical development plan.

Further changes were made to adapt the enrollment criteria for the VAC52150 Vaccine Development Roll-over study, to change the sponsor name and to align the protocol with the new protocol template (dated 6 June 2016).

The changes made to the clinical protocol VAC52150EBL3002 Amendment 1, dated 17-Nov-2015, are listed below, including the rationale of each change and a list of all applicable sections.

Rationale: As requested by the ANSM, wording on the collection of "Immediate Reportable Events" was added after one subject in study VAC52150EBL2001 experienced a serious and very rare condition called "Miller Fisher syndrome" about a month after boost vaccination wih either MVA-BN-Filo or placebo.

SYNOPSIS
Time and Events Schedule
1.1.2.3 Relevant Safety Information From Ongoing VAC52150 Studies
1.2.5 Overall Benefit/Risk Assessment
2.2 Endpoints
3.1 Overview of Study Design
8 PRESTUDY AND CONCOMITANT THERAPY
9.1.2 Screening Phase
9.1.3 Study Visits
9.3 Safety Evaluations
11.5 Safety Analyses
12.1.1 Adverse Event Definitions and Classifications
12.2 Special Reporting Situations
12.3.1 All Adverse Events
12.3.3 Immediate Reportable Events

Rationale: Adverse events of special interest (cardiovascular events) will no longer be collected as no cardiovascular events have been associated with the current MVA-BN-Filo vaccine. Information was added on the procedure that needs to be followed in case any cardiac sign or symptom develops after the boost vaccination.

1.2.5 Overall Benefit/Risk Assessment
9.3 Safety Evaluations
11.5 Safety Analyses
12.2 Special Reporting Situations

Rationale: Further details regarding enrollment into the VAC52150 roll-over study have been added, such as the inclusion of placebo subjects before unblinding of the current study.

SYNOPSIS
3.1 Overview of Study Design
9.1.4 VAC52150 Vaccine Development Roll-over Study
12.3.4 Pregnancy

Rationale: One case of chest pain, that might be indicative of pericarditis and that was not finally confirmed, was observed in the MVA-BN clinical trial program and has been added to the Potential Risks section.

1.2.4 Potential Risks

Rationale: The name of the sponsor changed from Crucell Holland B.V. to Janssen Vaccines & Prevention B.V. (formerly known as Crucell Holland B.V.).

TITLE PAGE
1 INTRODUCTION
INVESTIGATOR AGREEMENT

Rationale: Minor textual changes were made, in addition to updates to be in line with other current protocols.

Time and Events Schedule
ABBREVIATIONS
1 INTRODUCTION
4.1 Inclusion Criteria
9.1.2 Screening Phase
9.1.3 Study Visits
9.3 Safety Evaluations
11.1 Analysis Sets
11.5 Safety Analyses
12.1.1 Adverse Event Definitions and Classifications
16.2.5 Long-term Retention of Samples for Additional Future Research
REFERENCES
Attachment 1

Rationale: Changes were made to align the protocol with the new protocol template (dated 6 June 2016).

SYNOPSIS
2 OBJECTIVES, ENDPOINTS AND HYPOTHESIS
9.2 Immunogenicity Evaluations
9.3 Safety Evaluations
10.2 Discontinuation of Study Vaccination/Withdrawal From the Study
10.3 Withdrawal From the Use of Research Samples
12.1.1 Adverse Event Definitions and Classifications
12.3.4 Pregnancy
17.5 Case Report Form Completion

Amendment_1 (17 November 2015)

The overall reason for the amendment: This amendment includes the request of the Center for Biologics Evaluation and Research (CBER, a division of US Food and Drug Administration [FDA]) to extend the long-term safety follow-up to 6 months post-boost.

The changes made to the clinical protocol VAC52150EBL3002, dated 30-Jul-2015, are listed below, including the rationale of each change and a list of all applicable sections.

Rationale: As requested by the CBER (US FDA), the 6-month visit has been changed to 6-month post-boost visit.

SYNOPSIS
Time and Events Schedule
1.2.5 Overall Benefit/Risk Assessment
3.1 Overview of Study Design
4.3 Prohibitions and Restrictions
5 STUDY VACCINE ALLOCATION AND BLINDING
9.1.1 Overview
9.1.3 Study Visits
10.1 Completion

Rationale: As requested by the CBER (US FDA), the safety laboratory assessments at screening are to be performed within 28 days prior to the prime vaccination and may be repeated if they fall outside this time window.

Time and Events Schedule
4.1 Inclusion Criteria
9.1.2 Screening Phase

Rationale: The time of unblinding and database lock for primary analysis has been changed to when all subjects have completed the 6-month post-boost visit or discontinued earlier.

SYNOPSIS
Time and Events Schedule
1.2.5 Overall Benefit/Risk Assessment
3.1 Overview of Study Design
5 STUDY VACCINE ALLOCATION AND BLINDING
9.1.3 Study Visits
10.1 Completion
11 STATISTICAL METHODS

Rationale: As requested by the CBER (US FDA), the Per Protocol Analysis set will be used for assessment of the primary objective. The Immunogenicity Analysis set will be used to evaluate the robustness of the analysis results, and for all other analyses of immune response.

11.1 Analysis Sets

Rationale: Information regarding the marketing authorization of MVA-BN and the Phase 3 clinical study POX-MVA-013 has been updated.

1.1 Background
REFERENCES

Rationale: The VAC52150 Vaccine Development Registry was replaced by a roll-over study. Further details regarding enrollment have been added.

SYNOPSIS
3.1 Overview of Study Design
9.1.4 VAC52150 Vaccine Development Roll-over Study
12.3.4 Pregnancy

Rationale: Exploratory immunogenicity endpoints and assessments have been revised and corrected.

2.2 Endpoints
9.2 Immunogenicity Evaluations **Error! Reference source not found. Error! Reference source not found.**

Rationale: The protocol has been updated to be in line with the current protocol template (version 14 October 2015).

4 SUBJECT POPULATION
9.3 Safety Evaluations **Error! Reference source not found.**
10 SUBJECT COMPLETION/DISCONTINUATION OF STUDY VACCINATION/WITHDRAWAL FROM THE STUDY
12.2 Special Reporting Situations
12.4 Contacting Sponsor Regarding Safety
13.2 Contacting Sponsor Regarding Product Quality
16.2.2 Independent Ethics Committee or Institutional Review Board
16.2.6 Country Selection
17.1 Protocol Amendments
17.4 Source Documentation
17.5 Case Report Form Completion
17.6 Data Quality Assurance/Quality Control
17.7 Record Retention
17.8 Monitoring
17.9.1 Study Completion/End of Study
17.10 On-site Audits
17.11 Use of Information and Publication

Rationale: Minor textual changes have been made.

SYNOPSIS
ABBREVIATIONS
1 INTRODUCTION
2 OBJECTIVES, ENDPOINTS AND HYPOTHESIS
3.1 Overview of Study Design
9.1.1 Overview
9.2 Immunogenicity Evaluations **Error! Reference source not found.**
9.3 Safety Evaluations
10.2 Discontinuation of Study Vaccination/Withdrawal From the Study
11.1 Analysis Sets
11.2 Sample Size Determination
11.4 Immunogenicity Analyses
REFERENCES

SYNOPSIS

A Phase 3, Randomized, Double-blind, Placebo-controlled Study to Evaluate A Range of Dose Levels of a Heterologous Prime-boost Regimen of Ad26.ZEBOV and MVA-BN®-Filo in Healthy Adult Subjects

The sponsor, in collaboration with Bavarian Nordic (BN), is investigating the potential of a prophylactic Ebola vaccine regimen comprised of the following 2 candidate Ebola vaccines:

Ad26.ZEBOV is a monovalent vaccine expressing the full length Ebola virus (EBOV, formerly known as *Zaire ebolavirus*) Mayinga glycoprotein (GP), and is produced in the human cell line PER.C6®.

MVA-mBN226B, further referred to as Modified Vaccinia Ankara (MVA)-BN®-Filo, is a multivalent vaccine expressing the Sudan virus (SUDV) GP, the EBOV GP, the Marburg virus (MARV) Musoke GP, and the Tai Forest virus (TAFV, formerly known as *Côte d'Ivoire ebolavirus*) nucleoprotein (NP), and is produced in chicken embryo fibroblast cells. The EBOV GP expressed by MVA-BN-Filo has 100% homology to the one expressed by Ad26.ZEBOV.

In this Phase 3 study, the sponsor's adenovirus serotype 26 (Ad26) vaccine expressing the EBOV Mayinga GP (Ad26.ZEBOV) and the MVA-BN vaccine with EBOV, SUDV and MARV GP inserts and TAFV NP insert (MVA-BN-Filo) will be evaluated as a heterologous prime-boost regimen, in which one study vaccine (Ad26.ZEBOV) is used to prime a filovirus-specific immune response and the other study vaccine (MVA-BN-Filo) is used to boost the immune response. The EBOV GP that is currently circulating in West Africa has 97% homology to the EBOV GP used in this vaccine regimen.

OBJECTIVES, ENDPOINTS AND HYPOTHESIS

OBJECTIVES

Primary Objective

The primary objective is to demonstrate the non-inferiority of a heterologous prime-boost regimen using Ad26.ZEBOV $2x10^{10}$ viral particles (vp) as prime and MVA-BN-Filo $5x10^{7}$ infectious units (Inf.U) as boost at a 56-day interval (intermediate dose level) versus the same regimen with recently released batches of Ad26.ZEBOV $5x10^{10}$ vp and MVA-BN-Filo $1x10^{8}$ Inf.U (release titer), in terms of humoral immune response expressed by the ratio of geometric mean concentrations (GMC) against the EBOV GP as measured by enzyme-linked immunosorbent assay (ELISA, ELISA U/mL [EU/mL]) 21 days post-boost using a non-inferiority margin of 2/3. If non-inferiority of the intermediate dose level is demonstrated, non-inferiority of a low dose level, ie, Ad26.ZEBOV $0.8x10^{10}$ vp and MVA-BN-Filo $5x10^{7}$ Inf.U, versus the release titer will be investigated in the same way. The aim of these evaluations is to aid in establishment of specifications for end-expiry titers.

Secondary Objectives

The secondary objectives are:

- To assess humoral immune responses to the EBOV GP of a heterologous prime-boost regimen of Ad26.ZEBOV and MVA-BN-Filo administered intramuscularly (IM) at a 56-day interval using 3 different combinations of dose levels of Ad26.ZEBOV and MVA-BN-Filo as measured by ELISA (EU/mL) at all other time points.
- To assess the safety and tolerability of a heterologous prime-boost regimen of Ad26.ZEBOV and MVA-BN-Filo administered IM at a 56-day interval using 3 different combinations of dose levels of Ad26.ZEBOV and MVA-BN-Filo.

Exploratory Objectives

The exploratory objectives are:

- To further explore humoral immune responses to different EBOV GPs and the adenovirus and MVA backbones.
- To explore cellular immune responses to different EBOV GPs using enzyme-linked immunospot (ELISpot) and intracellular cytokine staining (ICS).

ENDPOINTS

Primary Endpoint:

- Binding antibody levels against the EBOV GP using ELISA (EU/mL) at 21 days after the boost vaccination.

Secondary Endpoints:

- Binding antibody levels against the EBOV GP using ELISA (EU/mL) at all other time points.
- Solicited local and systemic adverse events (reactogenicity) until 7 days post vaccination.
- Adverse events until the 42-day post-boost visit (Day 99).
- Serious adverse events and immediate reportable events (IREs) until the end of the study.

Exploratory Endpoints:

Exploratory endpoints may include, but might not be limited to, the following assays:

- Neutralizing antibody responses against the EBOV GP defined as the serum titer that is able to inhibit viral infection by a certain percentage (50%/80% and/or 90% inhibitory concentration [IC50/IC80 and/or IC90]) in the virus neutralization assay.
- Binding and/or neutralizing antibody responses against Ad26 and/or MVA vector in ELISA or neutralization assays.
- Humoral immune responses to different EBOV and/or Filovirus GPs, if assays are available.
- Molecular and functional characterization of study vaccine-elicited antibodies, which may include, but will not be limited to, repertoire analysis, Fc characterization, isotype analysis and epitope mapping.
- The presence and functional capacity of T cells will be determined after pathogen-specific stimulation of PBMC with EBOV GP-specific peptides in the IFN-γ ELISpot assay. Cytokine-producing T cells can be quantified using ELISpot technology.
- Activation of CD4+ and CD8+ T cell subsets and their cytokine expression patterns may be determined by flow cytometry (ICS) after EBOV GP-specific stimulation of PBMC (including, but not limited to, IFN-γ, interleukin [IL]-2, and tumor necrosis factor [TNF]-α).

HYPOTHESIS

Null Hypothesis:

The EBOV GP-specific antibody GMC 21 days post-boost (following administration of Ad26.ZEBOV 2×10^{10} vp and MVA-BN-Filo 5×10^{7} Inf.U at a 56-day interval) is inferior by at least 2/3 to the GMC

following administration of recently released batches of Ad26.ZEBOV 5x10^{10} vp and MVA-BN-Filo 1x10^{8} Inf.U for the ratio of $GMC_{intermediate\ dose\ level}/GMC_{release\ titer}$.

The EBOV GP-specific antibody GMC 21 days post-boost (following administration of Ad26.ZEBOV 0.8x10^{10} vp and MVA-BN-Filo 5x10^{7} Inf.U at a 56-day interval) is inferior by at least 2/3 to the GMC following administration of recently released batches of Ad26.ZEBOV 5x10^{10} vp and MVA-BN-Filo 1x10^{8} Inf.U for the ratio of $GMC_{low\ dose\ level}/GMC_{release\ titer}$.

Alternative Hypothesis:

The EBOV GP-specific antibody GMC 21 days post-boost (following administration of Ad26.ZEBOV 2x10^{10} vp and MVA-BN-Filo 5x10^{7} Inf.U at a 56-day interval) is non-inferior to the GMC following administration of recently released batches of Ad26.ZEBOV 5x10^{10} vp and MVA-BN-Filo 1x10^{8} Inf.U using a 2/3 non-inferiority margin for the ratio of $GMC_{intermediate\ dose\ level}/GMC_{release\ titer}$.

The EBOV GP-specific antibody GMC 21 days post-boost (following administration of Ad26.ZEBOV 0.8x10^{10} vp and MVA-BN-Filo 5x10^{7} Inf.U at a 56-day interval) is non-inferior to the GMC following administration of recently released batches of Ad26.ZEBOV 5x10^{10} vp and MVA-BN-Filo 1x10^{8} Inf.U using a 2/3 non-inferiority margin for the ratio of $GMC_{low\ dose\ level}/GMC_{release\ titer}$.

To assess non-inferiority, hierarchical testing is applied: non-inferiority of the intermediate dose level will be shown if the 95% confidence interval (CI) of the ratio of the anti-EBOV GP ELISA GMC of Ad26.ZEBOV and MVA-BN-Filo at the intermediate dose level over Ad26.ZEBOV and MVA-BN-Filo at the release titer is entirely above the non-inferiority margin of 2/3. If non-inferiority is shown, then also for the low dose level, non-inferiority towards the release titer will be tested in the same way.

OVERVIEW OF STUDY DESIGN

This is a randomized, double-blind, placebo-controlled, parallel-group, multicenter, Phase 3 study to evaluate a range of dose levels of Ad26.ZEBOV and MVA-BN-Filo to aid in establishment of end-expiry specifications using Ad26.ZEBOV at a dose of 5x10^{10}, 2x10^{10} or 0.8x10^{10} vp as prime and MVA-BN-Filo at a dose of 1x10^{8} or 5x10^{7} Inf.U as boost at a 56-day interval in healthy adult subjects in the United States (US). The prime-boost regimen will only differ in the dose level of Ad26.ZEBOV (ie, 5x10^{10}, 2x10^{10} or 0.8x10^{10} vp, respectively referred to as Groups 1, 2 and 3) or MVA-BN-Filo (ie, 1x10^{8} Inf.U [Group 1] or 5x10^{7} Inf.U [Groups 2 and 3]), while the timing of the boost vaccination (ie, 56 days after prime) and the sequence of vaccination will be identical.

Approximately 525 subjects will be enrolled and randomly assigned to one of the 4 groups: to one of 3 groups receiving Ad26.ZEBOV and MVA-BN-Filo (Groups 1 to 3) with approximately 150 subjects per group, or to a placebo group (Group 4) with approximately 75 subjects.

The subject population will consist of healthy men and women aged between 18 and 50 years (inclusive), who have never received a candidate Ebola vaccine and have not had prior exposure to Ebola virus (including travel to West Africa less than 1 month prior to screening) or a diagnosis of Ebola virus disease.

The subjects will be randomized at baseline (on Day 1) in a 2:2:2:1 ratio to Groups 1, 2, 3 and 4. Subjects will be randomized to receive the prime-boost regimen with either Ad26.ZEBOV and MVA-BN-Filo or placebo. Randomization will be stratified by site.

Group	N	Prime	Boost
		Day 1	Day 57
1	150	Ad26.ZEBOV $5x10^{10}$ vp	MVA-BN-Filo $1x10^{8}$ Inf.U
2	150	Ad26.ZEBOV $2x10^{10}$ vp	MVA-BN-Filo $5x10^{7}$ Inf.U
3	150	Ad26.ZEBOV $0.8x10^{10}$ vp	MVA-BN-Filo $5x10^{7}$ Inf.U
4	75	Placebo (0.9% saline)	Placebo (0.9% saline)

N: planned number of subjects to receive study vaccine

Study-site personnel (except for those with primary responsibility for study vaccine preparation and dispensing), sponsor personnel and subjects will be blinded to the study vaccine allocation until the time of database lock for the primary analysis, when all subjects have completed the 6-month post-boost visit or discontinued earlier. See below for details on blinding in case of interim analyses.

All subjects will receive the study vaccine (Ad26.ZEBOV and MVA-BN-Filo or placebo) IM in the deltoid muscle, either Ad26.ZEBOV ($5x10^{10}$, $2x10^{10}$ or $0.8x10^{10}$ vp) on Day 1, followed by a boost vaccination of MVA-BN-Filo ($1x10^{8}$ or $5x10^{7}$ Inf.U) on Day 57; or placebo (0.9% saline) on Day 1, followed by a boost vaccination of placebo (0.9% saline) on Day 57.

The study consists of a screening phase of up to 6 weeks (starting from the moment the subject signs the informed consent form [ICF]). Subjects will be vaccinated at baseline (Day 1) and at Day 57, and will have follow-up visits at 7 and 28 days post-prime (Days 8 and 29), at 7, 21 and 42 days post-boost (Days 64, 78 and 99) and at 6 months post-boost (Day 237). All subjects will complete a 6-month post-boost visit (Day 237) to further assess safety and immunogenicity.

Subjects who reach the Day 237 visit prior to unblinding will be approached to consent for enrollment into the VAC52150 Vaccine Development Roll-over study for long-term surveillance (for a total of up to 60 months after the prime vaccination). After unblinding, only subjects who received Ad26.ZEBOV and/or MVA-BN-Filo will remain in the VAC52150 Vaccine Development Roll-over study for long-term safety surveillance. After unblinding, subjects who received placebo and have already been enrolled into the VAC52150 Vaccine Development Roll-over study will be discontinued from further participation in the roll-over study.

SUBJECT POPULATION

Screening of subjects for eligibility will be performed within 6 weeks before administration of the study vaccine on Day 1. The subject population will consist of healthy (on the basis of medical history, physical examination, electrocardiogram (ECG), vital signs, clinical laboratory testing, and clinical judgment) men and women aged between 18 and 50 years (inclusive), who have not had prior exposure to Ebola virus (including travel to West Africa less than 1 month prior to screening) or a diagnosis of Ebola virus disease. Subjects who have received a candidate Ebola vaccine or an experimental candidate Ad26- or MVA-based vaccine in the past or with known allergy or history of anaphylaxis or other serious adverse reactions to vaccines or vaccine products, including known allergy to egg, egg products and aminoglycosides will be excluded.

DOSAGE AND ADMINISTRATION

All subjects will receive a vaccination, according to randomization, on Day 1 and on Day 57 at the following dose levels:

- Ad26.ZEBOV: $5x10^{10}$ vp supplied in a single use vial (0.5 mL extractable) (Group 1), $2x10^{10}$ vp (Group 2) or $0.8x10^{10}$ vp (Group 3);
- MVA-BN-Filo: $1x10^{8}$ Inf.U (nominal titer) supplied in a single use vial (0.5 mL extractable) (Group 1), $5x10^{7}$ Inf.U (nominal titer) (Groups 2 and 3);
- Placebo: 0.9% saline, 0.5 mL (Group 4).

The Ad26.ZEBOV and MVA-BN-Filo dose levels used in Groups 2 and 3 will be prepared by dilution of the release titer by unblinded qualified study-site personnel with primary responsibility for study vaccine preparation and dispensing, and not involved in any other study-related procedure.

Study vaccines (Ad26.ZEBOV and MVA-BN-Filo or placebo) will be administered as 0.5-mL IM injections into the deltoid muscle by a blinded study vaccine administrator. The boost vaccination should be administered in the opposite arm from the prime vaccination.

After each vaccination, subjects will remain at the site for a total of 60 (±15) minutes post-vaccination to monitor for the development of any acute reactions, or longer if deemed necessary by the investigator.

Criteria for postponement of vaccination at the scheduled time for vaccine administration and contraindications to boost vaccination have been defined and will be applied by the investigator.

IMMUNOGENICITY EVALUATIONS

The investigator will collect samples for immunogenicity assessments as specified in the Time and Events Schedule, for evaluation of primary, secondary and exploratory endpoints. Samples to assess humoral immune responses will be taken from all subjects; samples to assess cellular immune responses will be taken from subjects at selected site(s) with the capabilities to process peripheral blood mononuclear cells (PBMC, targeted at 10% of all subjects). Subjects giving informed consent for the study will be informed that their leftover blood samples will be stored for potential future research. Subjects participating at selected site(s) where PBMC samples are collected will be asked explicitly to consent for potential future genetic research to be performed on PBMC samples. Subjects can withdraw consent for their samples to be used for future research at any time.

SAFETY EVALUATIONS

Safety evaluations will be performed as specified in the Time and Events Schedule.

Safety will be assessed by collection of solicited local and systemic adverse events (reactogenicity), unsolicited adverse events, IREs and serious adverse events. The subjects will be closely observed by study-site personnel for the first 30 (±10) minutes after each vaccination and again at 60 (±15) minutes post-vaccination, and any unsolicited, solicited local or systemic adverse events will be documented during this period. Upon discharge from the site, subjects will receive a diary, a thermometer and a ruler to measure and record body temperature and solicited local reactions. Subjects will be instructed to record solicited local and systemic adverse events in the diary in the evening after each vaccination and then daily for the next 7 days at approximately the same time each day. Diaries should be completed at home by the subject. The investigator will document unsolicited adverse events from signing of the ICF onwards until 42 days post-boost, and serious adverse events and IREs from signing of the ICF onwards until the end of the study. The secondary safety endpoints are adverse events, serious adverse events, IREs, and solicited local and systemic adverse events. Adverse events that are ongoing at 42 days post-boost vaccination will be followed by the investigator until resolution or stabilization.

Other safety assessments include vital signs (blood pressure, pulse/heart rate, and body temperature), physical examination and pregnancy testing.

The investigators, together with the sponsor's medical monitor, will be responsible for the safety monitoring of the study, and will halt vaccination of further subjects in case any of the pre-specified pausing rules have been met.

STATISTICAL METHODS

Sample size calculations were performed under the following assumptions:

- Immune response is measured by binding antibody levels against the EBOV GP (using ELISA) at 21 days post-boost (following an Ad26.ZEBOV prime and MVA-BN-Filo boost after 56 days).
- A standard deviation of 0.303 at the $\log_{10}$ scale (21 days post-boost, following an Ad26.ZEBOV prime and MVA-BN-Filo boost after 56 days) in Groups 1 to 3.
- GMC of intermediate dose level or low dose level = 0.9xGMC of release titer.
- Hierarchical testing will be applied, such that non-inferiority of the intermediate dose level towards the release titer is tested first. If non-inferiority is shown, then also for the low dose level, non-inferiority towards the release titer will be tested.

A total of 150 subjects per group receiving Ad26.ZEBOV as prime and MVA-BN-Filo as boost are needed to have an overall power of at least 90% to show:

(1) non-inferiority in immune response after vaccination with Ad26.ZEBOV $2x10^{10}$ vp as prime and MVA-BN-Filo $5x10^{7}$ Inf.U as boost 56 days later, compared to vaccination with recently released batches of Ad26.ZEBOV $5x10^{10}$ vp as prime and MVA-BN-Filo $1x10^{8}$ Inf.U as boost 56 days later; and

(2) subsequently, if non-inferiority is shown for (1), to show non-inferiority in immune response after vaccination with Ad26.ZEBOV $0.8x10^{10}$ vp as prime and MVA-BN-Filo $5x10^{7}$ Inf.U as boost 56 days later, compared to vaccination with recently released batches of Ad26.ZEBOV $5x10^{10}$ vp as prime and MVA BN Filo $1x10^{8}$ Inf.U as boost 56 days later.

The sample size calculation takes a 10% overall dropout rate into account. Adding a control group of approximately 75 subjects receiving placebo, the total sample size will be approximately 525 subjects.

Non-inferiority of the intermediate dose level will be shown if the 95% CI of the ratio of the anti-EBOV GP ELISA GMC of Ad26.ZEBOV and MVA-BN-Filo at the intermediate dose level over Ad26.ZEBOV and MVA-BN-Filo at the release titer is entirely above the non-inferiority margin of 2/3. This non-inferiority margin has been used in the development of other vaccines for which no correlate of protection has been established. If non-inferiority is shown, then also for the low dose level, non-inferiority towards the release titer will be tested in the same way.

The primary analysis will be performed when all subjects have completed the last study-related visit (ie, Day 237) or discontinued earlier.

Interim analyses may be performed during the study for the purpose of informing future vaccine-related decisions in a timely manner. The results will not influence the conduct of the study in terms of early termination or later safety or immunogenicity endpoint assessments. Study-site personnel (except for those with primary responsibility for study vaccine preparation and dispensing), the sponsor (except for programming, statistics, clinical and clinical immunology personnel involved in the analysis, and the sponsor committee involved in making future decisions for the program) and subjects will remain blinded to study vaccine allocation until the time of database lock for the primary analysis.

Specific details will be provided in the Statistical Analysis Plan (SAP).

A Data Review Committee (DRC) will be established by the sponsor before the start of the study and will convene to review the available safety data, as outlined in the charter, in case a pausing rule is met. Ad hoc DRC meetings may be requested via the sponsor for any single event or combination of multiple events which are considered to jeopardize the safety of the subjects.

Immunogenicity Analyses

Descriptive statistics (actual values and changes from baseline, including 95% CIs, if applicable) will be calculated for continuous immunologic parameters by time point. Graphical representations of changes in immunologic parameters will be prepared, as applicable. Frequency tabulations will be calculated for discrete (qualitative) immunologic parameters by time point.

To assess the primary objective, only the Ad26.ZEBOV and MVA-BN-Filo vaccinated groups are considered. For each pairwise comparison of the ELISA actual values (in EU/mL) at 21 days post-boost of the intermediate dose level versus the release titer, estimated differences will be expressed as ratios of GMC with corresponding 95% CI. This 95% CI is determined from comparing log_{10}-transformed ELISA concentrations between groups and back-transformation of the estimated difference and corresponding 95% CI. Non-inferiority of a dose level compared to the release titer will be shown if the 95% CI of the estimated GMC ratio ($GMC_{intermediate\ (or\ low)\ dose\ level}/GMC_{release\ titer}$) entirely lies above 2/3. Hierarchical testing will be applied: in case non-inferiority of the intermediate dose level versus the release titer is shown, non-inferiority of the low dose level versus the release titer will be tested in the same way.

The primary comparison will be repeated adjusted for sex, age and body weight as a sensitivity analysis.

As an exploratory analysis, response patterns over time for the immunologic parameters will be analyzed, taking into account within-subject correlations.

Safety Analyses

No formal statistical testing of safety data is planned. Adverse events and categorical safety parameters will be tabulated, and continuous safety parameters will be descriptively analyzed.

TIME AND EVENTS SCHEDULE

Groups 1, 2, 3 and 4	Screening Phase[a] (≤6 weeks)	Study Visits							
		Day 1	Day 8	Day 29	Day 57	Day 64	Day 78	Day 99[b]	Day 237[b]
**Study Procedures**		**Prime**	+7d pp	+28d pp	**Boost**	+7d pb	+21d pb	+42d pb	+6m pb
Screening/Administrative									
Informed consent[c]	X								
Inclusion/exclusion criteria	X[d]								
Medical history and demographics	X								
Prestudy therapies[e]	X								
Serum pregnancy test[f]	X								
Serology (HIV-1/2, hepatitis B/C)	X								
Follicle-stimulating hormone (FSH)[g]	X								
Check clinical status + available data		X[h]							
Randomization		X							
Study vaccine administration[i]		▲			▼				
Safety Assessments									
Urine pregnancy test[f]		X[j]			X[j]				
Physical examination[k]	X	X[j]	X	X	X[j]	X	X	X	X
Electrocardiogram[l]	X								
Vital signs[m]	X	X[j]			X[j]				
Distribution of subject diary[n]		X			X				
Review of subject diary by site staff			X			X			
Adverse events[o]	Continuous								
Serious adverse events and immediate reportable events[p]	Continuous								
Concomitant therapies	X	X	X	X	X	X	X	X	X[q]
Clinical Laboratory Assessments[q]									
Hematology, chemistry	X								
Urinalysis	X								
Immunogenicity Assessments[s]									
Blood sampling for humoral assays (# mL)		X[j] (40 mL)		X (20 mL)	X[j] (20 mL)		X (100 mL)	X (50 mL)	X (50 mL)
Blood sampling for cellular assays (# mL)		X[j] (60 mL)		X (40 mL)	X[j] (40 mL)		X (60 mL)	X (60 mL)	X (60 mL)

d pp: days post-prime; d pb: days post-boost; m pb: months post-boost;
▲ *Ad26.ZEBOV $5x10^{10}$ viral particles (vp), $2x10^{10}$ vp, $0.8x10^{10}$ vp or placebo* ▼ *MVA-BN-Filo $1x10^{8}$ infectious units (Inf.U), $5x10^{7}$ Inf.U or placebo.*

NOTE: *If a subject withdraws early from the study, early withdrawal assessments should be obtained per the assessments for the 42-day post-boost visit, with the exception of the immunogenicity assessments. A subject who wishes to withdraw consent from participation in the study will be offered an optional visit for safety follow-up (before formal withdrawal of consent), but the subject has the right to refuse.*

[a] Screening may be split into multiple days or visits. Retesting of values (eg, safety laboratory) that lead to exclusion is allowed once using an unscheduled visit during the screening phase. The safety laboratory assessments at screening are to be performed within 28 days prior to the prime vaccination and may be repeated if they fall outside this time window.

[b] In addition to the assessments scheduled for the 42-day post-boost visit, subjects will be instructed to contact the investigator before the next visit if they experience any adverse event or intercurrent illness that they perceive as relevant and/or can be possibly related to study vaccine in their opinion. All subjects will complete a 6-month post-boost visit (Day 237) to further assess safety and immunogenicity.

[c] Signing of the informed consent form (ICF) needs to be done before the first study-related activity. Minimum criteria for the availability of documentation supporting the eligibility criteria are described in Section 17.4.

[d] The investigators should ensure that all study enrollment criteria have been met at the end of the screening phase.

[e] Prestudy therapies up to 30 days prior to the start of screening and previous vaccinia/smallpox vaccination at any time prior to study entry must be recorded in the case report form (CRF).

[f] For women of childbearing potential.

[g] For women >45 years of age with amenorrhea for less than 2 years or at any age with amenorrhea for more than 6 months.

[h] If a subject's clinical status changes (including available laboratory results or receipt of additional medical records) after screening so the subject no longer meets eligibility criteria, the subject should be excluded from further participation in the study.

[i] After each vaccination, subjects will remain at the site for a total of 60 (±15) minutes post-vaccination to monitor for the development of any acute reactions, or longer if deemed necessary by the investigator. Solicited and unsolicited adverse events emerging during the observation period at the site will be recorded in the CRF.

[j] Prior to study vaccine administration.

[k] A full physical examination, including height and body weight, will be carried out at screening. At other visits, an abbreviated, symptom-directed examination will be performed as indicated by the investigator.

[l] A single, 12-lead electrocardiogram (ECG) (supine) after at least 5 minutes rest will be performed and interpreted locally. Additional ECG monitoring may be done at other time points during the study if clinically indicated based on signs and symptoms. If blood sampling or vital sign measurement is scheduled for the same time point as ECG recording, the procedures should be performed in the following order: vital signs, ECG(s), blood draw.

[m] Includes blood pressure, pulse/heart rate (at least 5 minutes of rest in supine position) and body temperature to be assessed prior to study vaccine administration.

[n] Subjects will use the subject diary to document solicited local and systemic adverse events (reactogenicity) in the evening after each vaccination and then daily for the next 7 days. If a solicited local or systemic adverse event is not resolved on Day 8, the follow-up will be captured on the diary.

[o] Pregnancies will be reported from signing of the ICF until the end of the study.

[p] For reporting of immediate reportable events, refer to Section 12.3.3.

[q] After the 42-day post-boost visit, concomitant therapies should only be recorded if given in conjunction with serious adverse events and immediate reportable events.

[r] Blood samples (~15 mL in total) will be collected for serum chemistry and hematology, serology (HIV-1/2, hepatitis B/C) and pregnancy testing.

[s] Blood samples will be collected for humoral assays from all subjects and for cellular assays from subjects at selected site(s) with the capabilities to process peripheral blood mononuclear cells (PBMC) (targeted at 10% of all subjects).

ABBREVIATIONS

Ad26	adenovirus serotype 26 (vector)
Ad26.ZEBOV	adenovirus serotype 26 expressing the Ebola virus Mayinga glycoprotein
ALT	alanine aminotransferase
AST	aspartate aminotransferase
β-hCG	β-human chorionic gonadotropin
BN	Bavarian Nordic
CI	confidence interval
CRF	case report form(s)
DRC	Data Review Committee
EBOV	Ebola virus
ECG	electrocardiogram
eDC	electronic data capture
EDTA	ethylenediaminetetraacetic acid
ELISA	enzyme-linked immunosorbent assay
ELISpot	enzyme-linked immunospot
EU	ELISA units
FACS	fluorescence-activated cell sorting
FSH	follicle stimulating hormone
GCP	Good Clinical Practice
GMC	geometric mean concentration
GP	glycoprotein
HBsAg	hepatitis B surface antigen
HCV	hepatitis C virus
HIV	human immunodeficiency virus
IC	inhibitory concentration
ICF	informed consent form
ICH	International Council for Harmonisation
ICS	intracellular cytokine staining
IEC	Independent Ethics Committee
IFN-γ	interferon gamma
IL	interleukin
IM	intramuscular(ly)
Inf.U	infectious units
IRB	Institutional Review Board
IRE	Immediate Reportable Event
IWRS	interactive web response system
MARV	Marburg virus
MedDRA	Medical Dictionary for Regulatory Activities
MVA	Modified Vaccinia Ankara
MVA-BN-Filo	Modified Vaccinia Ankara Bavarian Nordic vector expressing multiple filovirus proteins
MVS	master virus seed
NP	nucleoprotein
PBMC	peripheral blood mononuclear cells
PCR	polymerase chain reaction
PQC	Product Quality Complaint
QPA	quantitative PCR potency assay
RBC	red blood cell
SAP	Statistical Analysis Plan
SUDV	Sudan virus
SUSAR	suspected unexpected serious adverse reaction
TAFV	Tai Forest virus
$TCID_{50}$	50% tissue culture infective dose
THAM	tris (hydroxymethyl)-amino methane
TNF	tumor necrosis factor
US	United States
VISP	vaccine-induced seropositivity

vp	viral particles
WBC	white blood cell
WVS	working virus seed

DEFINITIONS OF TERMS

Study vaccine	Ad26.ZEBOV, MVA-BN-Filo or placebo.
Blinded study vaccine administrator	A blinded trained study nurse, medical doctor/investigator, or otherwise qualified health care provider.
Independent study vaccine monitor	An unblinded study vaccine monitor assigned to the study who is responsible for the unblinded interface between the sponsor and the investigational site pharmacy.

1. INTRODUCTION

Janssen Vaccines & Prevention B.V. (formerly known as Crucell Holland B.V.) (hereafter referred to as the sponsor), in collaboration with Bavarian Nordic (BN), is investigating the potential of a prophylactic Ebola vaccine regimen comprised of the following 2 candidate Ebola vaccines:

Ad26.ZEBOV is a monovalent vaccine expressing the full length Ebola virus (EBOV, formerly known as *Zaire ebolavirus*) Mayinga glycoprotein (GP), and is produced in the human cell line PER.C6®.

MVA-mBN226B, further referred to as Modified Vaccinia Ankara (MVA)-BN®-Filo, is a multivalent vaccine expressing the Sudan virus (SUDV) GP, the EBOV GP, the Marburg virus (MARV) Musoke GP, and the Tai Forest virus (TAFV, formerly known as *Côte d'Ivoire ebolavirus*) nucleoprotein (NP), and is produced in chicken embryo fibroblast cells. The EBOV GP expressed by MVA-BN-Filo has 100% homology to the one expressed by Ad26.ZEBOV.

For the most up-to-date nonclinical and clinical information regarding Ad26.ZEBOV and MVA-BN-Filo, refer to the latest versions of the Investigator's Brochures and Addenda (if applicable).[14,15] A brief summary of the nonclinical and clinical information is provided below.

The term "sponsor" used throughout this document refers to the entities listed in the Contact Information page(s), which will be provided as a separate document.

1.1. Background

Ebola viruses belong to the Filoviridae family and cause Ebola virus disease, which can induce severe hemorrhagic fever in humans and nonhuman primates. Case fatality rates in Ebola disease range from 25% to 90% (average: 50%), according to the World Health Organization.[23] These viruses are highly prioritized by the United States (US) Government, who has defined them as 'Category A' agents, due to the high mortality rate of infected individuals. Currently, no licensed vaccine, treatment or cure exists for this disease.

Filoviruses are named for their long, filamentous shape. Within this filamentous virus, a single 19-kilobase negative-sense ribonucleic acid (RNA) genome encodes 7 proteins: the GP, the polymerase, the NP, the secondary matrix protein, the transcriptional activator, the polymerase cofactor, and the matrix protein. The virion surface is covered by homotrimers of the viral GP, which is believed to be the sole host attachment factor for filoviruses. Following cell entry, the viruses replicate their genomes and viral proteins in the cytoplasm using an RNA-dependent RNA polymerase, which is carried into the cell together with the virus.[9]

1.1.1. Nonclinical Studies

Immunogenicity and Efficacy

Immunogenicity and efficacy of the vaccine combination Ad26.ZEBOV and MVA-BN-Filo was evaluated in a nonhuman primate model (ie, Cynomolgus macaques, *Macaca fascicularis*). The combination was assessed in a multivalent filovirus setting in a small number (2 per regimen) of

animals and the study included heterologous prime-boost regimens of adenovirus serotype 26 (Ad26), Ad35 and MVA-BN-Filo vectors expressing different Ebola and Marburg proteins. Full protection from Ebola virus disease and death after wild-type EBOV Kikwit 1995 challenge was obtained with all heterologous regimens, including the Ad26 and MVA vaccine regimen. All heterologous prime-boost regimens induced comparable immune responses against the EBOV Mayinga GP. Independently of the vaccine regimen, a strong boost effect was seen after heterologous prime-boost immunization. Two additional studies involving more animals are ongoing, to strengthen the robustness of the nonclinical efficacy data, and also to optimize the prime-boost schedule so as to obtain induction of protective immunity as quickly as possible, to specifically respond to the Ebola virus disease outbreak in West Africa.

Toxicology

A repeated-dose toxicity study in rabbits was performed with prime-boost combinations of Ad26.ZEBOV and MVA-BN-Filo. The different dose regimens were well tolerated when administered twice by intramuscular (IM) injection to New Zealand White rabbits with a 14-day interval period. Additionally, the objective was to assess the persistence, reversibility or delayed onset of any effects after a 14-day treatment-free period. In the heterologous prime-boost regimen, either vector or both were used to prime a filovirus-specific immune response and the other/same vector or both were used to boost the immune response 2 weeks later. All vaccine dosing regimens resulted in detectable EBOV GP-specific antibody titers. No significant toxicological effects (no adverse effects) were observed. The immune response was associated with transient increases in fibrinogen, C-reactive protein, globulin, decreases in hematocrit and hemoglobin, and microscopic findings in draining iliac lymph nodes, spleen and at the injection sites. The findings were noted to be recovering over a 2-week treatment-free period and were considered to reflect a physiological response associated with vaccination. There were no effects noted that were considered to be adverse.

Biodistribution

Single-dose biodistribution studies in rabbits were performed using the MVA-BN vector or the Ad26 vector in combination with another insert (Ad26.ENVA.01: an experimental, prophylactic Ad26 vector expressing the human immunodeficiency virus [HIV] type 1, Clade A envelope protein). MVA-BN distributed to the skin, muscle, blood, spleen, lung, liver, and pooled lymph nodes and was rapidly cleared (within 48 hours following vaccination). Ad26.ENVA.01 was primarily localized in the injection site muscle, the regional lymph nodes and the spleen. Three months after the single IM injection of Ad26.ENVA.01, the vaccine was cleared from most of the examined tissues. As biodistribution is dependent on the vector platform (MVA or Ad26) and not on the insert, it can be assumed that recombinant MVA-BN-Filo or Ad26.ZEBOV is distributed in the same way as the MVA-BN vector or Ad26.ENVA.01 vector, respectively.

1.1.2. Clinical Studies

1.1.2.1. Safety Profile of Ad26-based Vaccines

To date, no human clinical studies have been completed with Ad26.ZEBOV or MVA-BN-Filo. The safety/tolerability and immunogenicity of the Ad26.ZEBOV and MVA-BN-Filo vaccines

are being assessed in the ongoing Phase 1 studies (VAC52150EBL1001, VAC52150EBL1002, VAC52150EBL1003 and VAC52150EBL1004), where monovalent Ad26.ZEBOV and multivalent MVA-BN-Filo are combined in homologous or heterologous prime-boost regimens in which each vector is used to prime a filovirus-specific immune response followed by a boost immunization with the same or the other vector 2 to 8 weeks later. Two additional Phase 1 studies investigating MVA-BN-Filo are also ongoing (EBL01 and CVD-Mali Ebola Vaccine #1000). Refer to the latest versions of the Ad26.ZEBOV and MVA-BN-Filo Investigator's Brochures and Addenda (if applicable) for more details.[14,15]

Limited data from the ongoing Phase 1 studies with Ad26.ZEBOV and MVA-BN-Filo are available.

VAC52150EBL1001, a first-in-human study, enrolled 87 subjects. Based on the 7-day post-prime safety data (blinded on a treatment group level) on 72 subjects (36 per treatment group), the following information was obtained. Most of the adverse events reported were grade 1 or grade 2 in severity. Local injection site reactions were reported in 18 (50%) MVA/placebo subjects (all grade 1) and 28 (78%) Ad26/placebo subjects (grade 1 [22], grade 2 [5], grade 3 [1]). The most frequent local reaction was injection site pain, in 17 (47%) MVA/placebo subjects and 28 (78%) Ad26/placebo subjects, with one grade 3 case occurring in the Ad26/placebo group. Solicited systemic reactions were reported in 25 (69%) MVA/placebo subjects (grade 1 [24] and grade 2 [1]) and 31 (86%) Ad26/placebo subjects (grade 1 [22], grade 2 [7], grade 3 [1], unknown [1]). The most frequent systemic reactions were fatigue (50% overall), followed by headache (46%) and myalgia (35%). One Ad26/placebo subject experienced 3 grade 3 solicited systemic reactions (headache, myalgia and nausea). None of the subjects reported fever; however, 2 subjects had one temperature measurement missing. The most frequent unsolicited adverse events were decreased neutrophils, in 3 (8%) MVA/placebo subjects and 6 (17%) Ad26/placebo subjects, followed by activated partial thromboplastin time prolongation and hypokalemia, in 3 (8%) MVA/placebo subjects and 5 (14%) Ad26/placebo subjects each. All of these events were transient in nature and resolved without intervention. No deaths or serious adverse events and no adverse events leading to discontinuation of the study vaccination were reported.[14]

Study VAC52150EBL1002 completed enrollment of 128 subjects; the blinded phase of the study is ongoing. No serious adverse events related to study vaccine have been reported and no safety issues have been identified to date.

Safety data generated with the 2 backbones containing different inserts are provided below.

Safety Data From Other Ad26-based Vaccine Programs

Ad26.ZEBOV is a monovalent recombinant, replication-incompetent Ad26-based vaccine. Only limited clinical data are available for Ad26.ZEBOV. However, adenovirus vaccines containing other gene inserts revealed no significant safety issues. The data described below are based on the evaluation of the prototype vaccine Ad26.ENVA.01, which expresses the HIV envelope gene.[11]

Three randomized, placebo-controlled, Phase 1 studies (IPCAVD-001, IPCAVD-003, IPCAVD-004) have evaluated the safety and immunogenicity of the prototype vaccine Ad26.ENVA.01. This prototype vaccine has been administered to more than 200 healthy, HIV-negative subjects between the ages of 18 and 50 years in the US and Africa.[11,12,13]

- In the dose-escalation study IPCAVD-001 (n=60), 2 or 3 IM doses of Ad26.ENVA.01 ($1x10^9$, $1x10^{10}$, $5x10^{10}$, $1x10^{11}$ viral particles [vp]) were given to Ad26 seronegative subjects. There were no deaths or vaccine-related serious adverse events. Ad26.ENVA.01 was generally well tolerated at all 4 dose levels with minimal reactogenicity observed in the $1x10^9$ and $1x10^{10}$ vp dose groups. Moderate to severe malaise, myalgia, fatigue and chills occurred in the majority of subjects 12 to 18 hours after the first dose of $1x10^{11}$ vp, but were resolved within 24 to 36 hours and were not seen after the second injection at this dose level. Two subjects in the $1x10^{11}$ vp dose group chose not to have the second injection, however, 1 of them decided to have the 6-month injection. Envelope-specific humoral and cell-mediated immune responses were induced at all 4 dose levels of vaccine.[2,4]
- In the single-dose study IPCAVD-003 (n=24), an IM dose of Ad26.ENVA.01 ($5x10^{10}$ vp) or placebo was given to subjects, who were stratified according to baseline Ad26 immune status, to evaluate the safety, mucosal immunogenicity and innate immune responses. Local reactogenicity comprised moderate injection site pain/tenderness and/or moderate to severe erythema which resolved within 3 days of vaccination. Transient systemic reactogenicity comprised headache, chills, joint pain, myalgia, malaise/fatigue, and fever. No deaths or vaccine-related serious adverse events were observed. Vaccination elicited both systemic and mucosal envelope-specific humoral and cellular immune responses. No increased activated total or vector-specific mucosal CD4+ T-lymphocytes following vaccination were detected in the colorectal mucosa, indicating that vaccination with Ad26 did not increase mucosal inflammation.[1]
- In study IPCAVD-004 (n=217), the safety and immunogenicity of IM doses of Ad26.ENVA.01 and Ad35.ENV (an Ad35 vector expressing an HIV envelope GP used in that study at a dose of $5x10^{10}$ vp), given in heterologous and homologous prime-boost regimens at 3- versus 6-month intervals, was evaluated. There were 452 adverse events reported by 84 of 176 Ad26-vaccine recipients (47.7%), the majority being mild (75.5%) in severity. The proportion of subjects with moderate or severe symptoms was not statistically significantly different between vaccine and placebo. There were 3 serious adverse events: 2 serious adverse events in placebo recipients (grade 3 peritonsillar abscess and grade 4 migraine headache, both resolved with no residual effects) and 1 serious adverse event in an Ad35/Ad26 vaccine recipient (grade 4 acute myelogenous leukemia, resolved with sequelae). No deaths or vaccine-related serious adverse events were reported. Overall, 97% to 100% of subjects developed anti-envelope binding antibodies (enzyme-linked immunosorbent assay [ELISA]) after a second dose, with heterologous and homologous regimens being comparable. Immune responses in groups who received 3- and 6-month schedules were comparable. Four weeks post-vaccination, interferon (IFN)-γ enzyme-linked immunospot (ELISpot) assay showed response rates between 44% and 100%. The heterologous and homologous regimens were comparable. There was induction of Ad26-neutralizing antibodies in the majority of vaccine recipients after 2 immunizations with Ad26.ENVA.01.[16]

In addition, the sponsor performed a Phase 1/2a double-blind, randomized, placebo-controlled, dose-escalation study (MAL-V-A001) to evaluate the safety, tolerability and immunogenicity of 2 dose levels ($1x10^{10}$ and $5x10^{10}$ vp) of Ad35.CS.01/Ad26.CS.01 (both expressing the malaria *Plasmodium falciparum* circumsporozoite antigen) prime-boost regimens in healthy subjects. The dose-escalation phase was followed by an evaluation of efficacy of the higher dose level in an experimental malaria challenge. A total of 42 subjects were enrolled and were vaccinated. The analysis of adverse events did not show any consistent pattern suggestive of an association of Ad35.CS.01 or Ad26.CS.01 with specific adverse events. There were no serious adverse events reported during the study. No subject discontinued during a study phase (vaccination or challenge) due to adverse events. One subject in the high-dose group completed the vaccination phase and the final safety follow-up visit but did not take part in any challenge phase activities because of ongoing dyspnea. The most common related adverse events after each vaccination were injection site pain, malaise, headache, myalgia and chills. The incidence of vaccine-related adverse events was generally higher in the high-dose group than in the low-dose group. In general, incidence of malaise, headache, and myalgia were higher after the third dose (Ad26) than after the first or second doses (Ad35). Injection site pain was more commonly reported in the low and high-dose groups than by placebo subjects. There were no clinically significant changes in laboratory test parameters or vital signs data.[5]

Recent data indicate that administration of a deoxyribonucleic acid (DNA) vaccine expressing EBOV Mayinga GP, the same GP as in the Ad26.ZEBOV component, was safe, well tolerated and immunogenic in a Phase 1 clinical study. During this study, 9 subjects received three 4-weekly IM doses of vaccine (4 mg/dose), followed by a boost at ≥32 weeks in 8 subjects.[19]

1.1.2.2. Safety Profile of MVA-BN-based Vaccines

MVA-BN is a further attenuated version of the MVA virus, which in itself is a highly attenuated strain of the poxvirus Chorioallantois Vaccinia Virus Ankara. MVA-BN induces strong cellular activity as well as a humoral (antibody) immune response and has demonstrated an ability to stimulate a response even in individuals with pre-existing immunity against Vaccinia. One of the advantages of MVA-BN is the virus' inability to replicate in a vaccinated individual. The replication cycle is blocked at a very late stage, which ensures that new viruses are not generated and released. This means that the virus cannot spread in the vaccinated person and none of the serious side effects normally associated with replicating Vaccinia viruses have been seen with MVA-BN.

MVA-BN (MVA-BN®, trade name IMVAMUNE® outside the European Union, invented name IMVANEX® in the European Union) has received marketing authorization in the European Union for active immunization against smallpox in adults, and in Canada for persons 18 years of age and older who have a contraindication to the first or second generation smallpox vaccines including individuals with immune deficiencies and skin disorders.[10] A Phase 3 clinical study (POX-MVA-013) has been completed (ClinicalTrials.gov Identifier: NCT01144637).[6] Results of completed and ongoing clinical studies of MVA-BN-based vaccines in more than 8,100 individuals, including elderly, children and immunocompromised subjects in whom replicating vaccines are contraindicated, have shown that the platform displays high

immunogenicity and a favorable safety profile.[17] Across all clinical studies, no trends for unexpected or serious adverse reactions due to the product were detected.

Extensive nonclinical studies support the safety profile of the MVA-BN strain.[20,21]

1.1.2.3. Relevant Safety Information From Ongoing VAC52150 Studies

One subject in the study VAC52150EBL2001 experienced a serious and very rare condition called “Miller Fisher syndrome”. This condition consists of double vision, pain on moving the eye, and difficulty with balance while walking. Miller Fisher syndrome most commonly occurs following a recent infection. The subject experienced these symptoms about a week after suffering from a common cold and fever. The event happened about a month after boost vaccination with either MVA-BN-Filo or placebo. This subject had to go to the hospital for treatment and has recovered. After an extensive investigation, the event has been considered to be doubtfully related to vaccine and most likely related to the previous common cold.

In the ongoing clinical studies with more than 2,000 participants, there have been a few reports of mild to moderate tingling especially in the hands and feet or a sensation of mild to moderate muscle weakness in subjects vaccinated with Ad26.ZEBOV or placebo. These symptoms have been observed to last no more than 24 to 48 hours in the majority of cases but can last for several weeks before going away on their own. These types of symptoms have also been reported following administration of other licensed vaccines and following acute viral infections of various types. One serious case of probable peripheral sensory neuropathy of moderate severity has occurred and has been ongoing for several months, interfering with some of the subject’s daily activities.

1.2. Benefit/Risk Section

1.2.1. Known Benefits

The clinical benefit of prime-boost combinations of Ad26.ZEBOV and MVA-BN-Filo is to be established.

1.2.2. Potential Benefits

Subjects may benefit from clinical testing and physical examination; others may benefit from the knowledge that they may aid in the development of an Ebola vaccine. There is no direct individual benefit from vaccination for the subjects at the current development stage.

1.2.3. Known Risks

To date, there are only limited data from the Phase 1 studies with Ad26.ZEBOV and MVA-BN-Filo available. However, Ad26- and Ad35-based vaccines with other gene inserts have been administered to a limited number of human volunteers in clinical studies. These other vaccines mainly elicited some solicited local and systemic reactions, as expected with injectable vaccines, and no serious safety concerns in study participants. MVA-BN-based vaccines have been administered to more than 8,100 individuals without unexpected or serious adverse reactions reported. For details, see the safety data presented in Section 1.1.

1.2.4. Potential Risks

The following potential risks for Ad26.ZEBOV and MVA-BN-Filo will be monitored during the study and are specified in the protocol:

Risks Related to Vaccines

Subjects may exhibit general signs and symptoms associated with administration of a vaccine, or a placebo vaccination, including nausea/vomiting, headache, myalgia, arthralgia, fever, fatigue/malaise and chills. In addition, subjects may experience local (injection site) reactions such as pain/tenderness, erythema, induration/swelling and itching at the injection site. These events will be monitored, but are generally short-term and do not require treatment.

Subjects may have an allergic reaction to the vaccination. An allergic reaction may cause a rash, hives or even difficulty breathing (anaphylaxis). Severe reactions are rare. Medications must be available in the clinic to treat serious allergic reactions.

The risks related to vaccine-induced seropositivity (VISP) are discussed in Section 9.4.

Risk of Myo/Pericarditis

While replicating smallpox vaccines have been associated with an increased risk to develop myo/pericarditis,[18] this has not been observed with MVA-BN and is not expected with this highly attenuated, non-replicating vaccine. Based on observations with first- and second-generation replication-competent smallpox vaccines, particular attention has been placed on the monitoring for cardiac signs and symptoms in all clinical studies using MVA-BN. Despite the close cardiac monitoring, no event indicating a case of pericarditis has been observed in any completed MVA-BN study. There has been 1 case of chest pain that might be indicative of pericarditis (consisting of chest pain only with no other cardiac findings suggestive of pericarditis) with previous MVA use although this diagnosis was not finally confirmed and the subject fully recovered. In a review of prospective surveillances for cardiac adverse events in 6 different clinical studies in 382 subjects receiving MVA vaccines, only 1 subject (0.3%) met the criteria for vaccine-induced myocarditis and eventually the subject was found to suffer from exercise-induced palpitations. Self-limited mild elevations in troponin I were recorded in 3 subjects (0.8%) without evidence of myo/pericarditis.[7] Based on the current exposure data in more than 8,100 subjects vaccinated with MVA-BN and other MVA-BN recombinant products, the safety profile of MVA-BN has shown to be comparable with other licensed, live attenuated vaccines.

Pregnancy and Birth Control

The effect of the study vaccines on a fetus or nursing baby is unknown, as well as the effect on semen, so women of childbearing potential, and men having sexual intercourse with women, are required to agree to practice adequate birth control measures for sexual intercourse from at least 28 days before the prime vaccination (or prior to enrollment for men) until at least 3 months after the prime vaccination or up to 28 days after the boost vaccination (whichever takes longer) (see Section 4.3). Women who are pregnant or breast-feeding, or are planning to become pregnant

while enrolled in the study until 3 months after the prime vaccination or up to 28 days after the boost vaccination (whichever takes longer), will be excluded from enrollment into the study.

Risks from Blood Draws

Blood draws may cause pain/tenderness, bruising, bleeding, and, rarely, infection at the site where the blood is taken.

Unknown Risks

There are no clinical data on the use of either vaccine (Ad26.ZEBOV or MVA-BN-Filo) in:

- Children (<18 years);
- Pregnant or nursing women;
- Adults >50 years;
- Immunocompromised individuals (including those with HIV infection).

There may be other serious risks that are not known.

1.2.5. Overall Benefit/Risk Assessment

Based on the available data and proposed safety measures, the overall benefit/risk assessment for this clinical study is considered acceptable for the following reasons:

Preliminary safety data from the ongoing Phase 1 studies and safety data generated with the 2 vaccines with different inserts revealed no significant safety issues (see Sections 1.1 and 1.2.3). Further experience from Ad26.ZEBOV or MVA-BN-Filo will be gained from currently ongoing clinical studies.

- Only subjects who meet all inclusion criteria and none of the exclusion criteria (specified in Section 4) will be allowed to participate in this study. The selection criteria include adequate provisions to minimize the risk and protect the well-being of subjects in the study.
- Safety will be closely monitored throughout the study:
  - After each vaccination, subjects will remain at the site for a total of 60 (±15) minutes post-vaccination to monitor the development of any acute reactions, or longer if deemed necessary by the investigator. Refer to Section 6.1 for more information on emergency care. The subjects will be closely observed by study-site personnel for the first 30 (±10) minutes after each vaccination and again at 60 (±15) minutes post-vaccination, and any unsolicited, solicited local or systemic adverse events will be documented during this period. Subjects will use a diary to document solicited local and systemic adverse events in the evening after each vaccination and then daily for the next 7 days at approximately the same time each day. Diaries should be completed at home by the subject.

- The investigator or the designee will document unsolicited adverse events from signing of the informed consent form (ICF) onwards until 42 days post-boost, and serious adverse events and immediate reportable events (IREs) from signing of the ICF onwards until the end of the study.
- Safety evaluations, including an electrocardiogram (ECG; performed at screening and at other time points during the study if clinically indicated based on signs and symptoms), physical examinations, vital sign measurements, clinical laboratory testing (performed at screening) and pregnancy testing, will be performed at scheduled visits during the study, which lasts up to 6 months after the boost vaccination.
- Any clinically significant abnormalities (including those persisting at the end of the study/early withdrawal) will be followed by the investigator until resolution or until a clinically stable endpoint is reached.

- Several safety measures are included in this protocol to minimize the potential risk to subjects, including the following:
  - The neuroinflammatory disorders listed in Section 12.1.1 should be categorized as IREs and should be reported to the sponsor as described in Section 12.1.1.
  - There are pre-specified pausing rules that would result in pausing of further vaccination if predefined conditions occur, preventing exposure of new subjects to study vaccine until a Data Review Committee (DRC) reviews all safety data (see Section 9.3.1).
  - Subjects will discontinue study vaccine for the reasons included in Section 10.2.
  - If acute illness (excluding minor illnesses such as diarrhea or mild upper respiratory tract infection) or fever (body temperature ≥38.0°C) occur at the scheduled time for vaccination, the subject may be vaccinated up to 10 days beyond the window allowed for the scheduled vaccination, or be withdrawn from that vaccination at the discretion of the investigator and after consultation with the sponsor (see Section 6.2).
  - Contraindications to boost vaccinations are included in Section 6.3.
  - If a subject withdraws from the study (withdrawal of consent), he/she maintains the option to participate in the safety follow-up (see Section 10.2).

1.3. Overall Rationale for the Study

In nonclinical studies in the Cynomolgus macaque model, heterologous prime-boost regimens of a multivalent mixture of Ad26 vectors (each expressing EBOV Mayinga, SUDV or MARV GP) and MVA-BN-Filo provided complete protection against the highly pathogenic wild-type EBOV Kikwit 1995 variant (report pending). Further nonclinical studies are ongoing to evaluate the protection of the multivalent vaccine regimen in additional animals and to assess the protective efficacy of a combination regimen of Ad26.ZEBOV and MVA-BN-Filo (either a simultaneous administration or as prime-boost regimen).

In humans, both Ad26- and MVA-based vaccines containing various antigenic inserts have been shown to be safe and immunogenic (see Section 1.1). To date, more than 230 subjects have

received the sponsor's Ad26-based vaccines in completed clinical studies (based on the adenoviral vaccine safety database report [dated 20 March 2015]). Up to 28 October 2015, 227 subjects received Ad26.ZEBOV in ongoing studies. The MVA-BN platform is the basis of the non-replicating smallpox vaccine registered in Canada and Europe, and has been safely used in more than 7,600 humans.[17] Although routinely used by the subcutaneous route, MVA-BN at a dose of $1x10^8$ 50% Tissue Culture Infective Dose ($TCID_{50}$) has been demonstrated to be as safe and immunogenic when used by the IM route.[8,22] The IM route has been chosen for the present study.

This study is one of a series of studies to evaluate the heterologous combination of Ad26.ZEBOV and MVA-BN-Filo as a possible vaccine regimen to prevent Ebola virus disease. The concept of a prime-boost regimen that will be evaluated with the candidate prophylactic Ebola vaccines Ad26.ZEBOV and MVA-BN-Filo is supported by the results of clinical studies with candidate malaria vaccines which have demonstrated that Ad-based prime immunization followed by MVA-vector boost induced high levels of immunity. The sponsor's Ad26 vaccine expressing the EBOV Mayinga GP (Ad26.ZEBOV) and the MVA-BN vaccine with EBOV, SUDV and MARV GP inserts and TAFV NP insert (MVA-BN-Filo) will be evaluated as a heterologous prime-boost regimen using Ad26.ZEBOV to prime a filovirus-specific immune response and MVA-BN-Filo to boost the immune response 56 days later. The EBOV GP that is currently circulating in West Africa has 97% homology to the EBOV GP used in this vaccine regimen.[3]

This study will support the lower specification for potency over the expected shelf life for both Ad26.ZEBOV and MVA-BN-Filo. It is the intention to aid in establishment of end-expiry specifications using a prime-boost regimen consisting of Ad26.ZEBOV and MVA-BN-Filo vaccines through the evaluation of a range of dose levels: Ad26.ZEBOV at either $5x10^{10}$ vp (release titer), $2x10^{10}$ vp (intermediate dose level), or $0.8x10^{10}$ vp (low dose level) and MVA-BN-Filo at either $1x10^8$ infectious units (Inf.U) (release titer) or $5x10^7$ Inf.U (lower dose).

The Ad26.ZEBOV intermediate dose level ($2x10^{10}$ vp) and low dose level ($0.8x10^{10}$ vp) are based on the infectivity as monitored using the quantitative polymerase chain reaction (PCR) potency assay (QPA) method during storage. The reason is that the VP-qPCR method, which measures viral particles based on viral DNA amplification using PCR, is not stability indicating. The QPA method, which provides a measure of infectivity by measuring newly synthesized viral DNA after infection of a cell monolayer by Ad26.ZEBOV, is stability indicating and can therefore detect changes in the product. For consistency reasons, all clinical doses are calculated to and expressed in vp.

The MVA-BN-Filo lower dose ($5x10^7$ Inf.U) is based on the potency as monitored using fluorescence-activated cell sorting (FACS) method during storage. The FACS method has stability-indicating potential (as has been extensively shown for IMVAMUNE®) and can therefore detect changes in the product. The lower dose of $5x10^7$ Inf.U is defined as the lower limit that is still able to trigger the immune reaction and is slightly lower than the current actual stability specification of $1.55x10^8$ Inf.U/mL (or $7.5x10^7$ Inf.U per 0.5 mL dose) as a safety approach.

This Phase 3 study will be conducted to identify the immunogenicity of 3 different combinations of dose levels, using a prime-boost regimen consisting of Ad26.ZEBOV and MVA-BN-Filo administered at a 56-day interval at the following doses: recently released batches of Ad26.ZEBOV $5x10^{10}$ vp and MVA-BN-Filo $1x10^{8}$ Inf.U (release titer), Ad26.ZEBOV $2x10^{10}$ vp and MVA-BN-Filo $5x10^{7}$ Inf.U (intermediate dose level), or Ad26.ZEBOV $0.8x10^{10}$ vp and MVA-BN-Filo $5x10^{7}$ Inf.U (low dose level), through the measurement of geometric mean concentrations (GMC) by ELISA. This study will also allow for expansion of the safety experience of the Ad26.ZEBOV/MVA-BN-Filo prime-boost regimen.

2. OBJECTIVES, ENDPOINTS AND HYPOTHESIS

2.1. Objectives

Primary Objective

The primary objective is to demonstrate the non-inferiority of a heterologous prime-boost regimen using Ad26.ZEBOV $2x10^{10}$ vp as prime and MVA-BN-Filo $5x10^{7}$ Inf.U as boost at a 56-day interval (intermediate dose level) versus the same regimen with recently released batches of Ad26.ZEBOV $5x10^{10}$ vp and MVA-BN-Filo $1x10^{8}$ Inf.U, in terms of humoral immune response expressed by the ratio of GMC against the EBOV GP as measured by ELISA (ELISA U/mL [EU/mL]) 21 days post-boost using a non-inferiority margin of 2/3. If non-inferiority of the intermediate dose level is demonstrated, non-inferiority of a low dose level, ie, Ad26.ZEBOV $0.8x10^{10}$ vp and MVA-BN-Filo $5x10^{7}$ Inf.U, versus the release titer will be investigated in the same way. The aim of these evaluations is to aid in establishment of specifications for end-expiry titers.

Secondary Objectives

The secondary objectives are:

- To assess humoral immune responses to the EBOV GP of a heterologous prime-boost regimen of Ad26.ZEBOV and MVA-BN-Filo administered intramuscularly (IM) at a 56-day interval using 3 different combinations of dose levels of Ad26.ZEBOV and MVA-BN-Filo as measured by ELISA (EU/mL) at all other time points.
- To assess the safety and tolerability of a heterologous prime-boost regimen of Ad26.ZEBOV and MVA-BN-Filo administered IM at a 56-day interval using 3 different combinations of dose levels of Ad26.ZEBOV and MVA-BN-Filo.

Exploratory Objectives

The exploratory objectives are:

- To further explore humoral immune responses to different EBOV GPs and the adenovirus and MVA backbones.
- To explore cellular immune responses to different EBOV GPs using ELISpot and intracellular cytokine staining (ICS).

2.2. Endpoints

Primary Endpoint:

- Binding antibody levels against the EBOV GP using ELISA (EU/mL) at 21 days after the boost vaccination.

Secondary Endpoints:

- Binding antibody levels against the EBOV GP using ELISA (EU/mL) at all other time points.
- Solicited local and systemic adverse events (reactogenicity) until 7 days post vaccination.
- Adverse events until the 42-day post-boost visit (Day 99).
- Serious adverse events and IREs until the end of the study.

Exploratory Endpoints:

Exploratory endpoints may include, but might not be limited to, the following assays:

- Neutralizing antibody responses against the EBOV GP defined as the serum titer that is able to inhibit viral infection by a certain percentage (50%/80% and/or 90% inhibitory concentration [IC_{50}/IC_{80} and/or IC_{90}]) in the virus neutralization assay.
- Binding and/or neutralizing antibody responses against Ad26 and/or MVA vector in ELISA or neutralization assays.
- Humoral immune responses to different EBOV and/or Filovirus GPs, if assays are available.
- Molecular and functional characterization of study vaccine-elicited antibodies, which may include, but will not be limited to, repertoire analysis, Fc characterization, isotype analysis and epitope mapping.
- The presence and functional capacity of T cells will be determined after pathogen-specific stimulation of PBMC with EBOV GP-specific peptides in the IFN-γ ELISpot assay. Cytokine-producing T cells can be quantified using ELISpot technology.
- Activation of CD4+ and CD8+ T cell subsets and their cytokine expression patterns may be determined by flow cytometry (ICS) after EBOV GP-specific stimulation of PBMC (including, but not limited to, IFN-γ, interleukin [IL]-2, and tumor necrosis factor [TNF]-α).

2.3. Hypothesis

Null Hypothesis:

The EBOV GP-specific antibody GMC 21 days post-boost (following administration of Ad26.ZEBOV $2x10^{10}$ vp and MVA-BN-Filo $5x10^{7}$ Inf.U at a 56-day interval) is inferior by at least 2/3 to the GMC following administration of recently released batches of Ad26.ZEBOV $5x10^{10}$ vp and MVA-BN-Filo $1x10^{8}$ Inf.U for the ratio of $GMC_{\text{intermediate dose level}}/GMC_{\text{release titer}}$.

The EBOV GP-specific antibody GMC 21 days post-boost (following administration of Ad26.ZEBOV at $0.8x10^{10}$ vp and MVA-BN-Filo $5x10^{7}$ Inf.U at a 56-day interval) is inferior by

at least 2/3 to the GMC following administration of recently released batches of Ad26.ZEBOV $5x10^{10}$ vp and MVA-BN-Filo $1x10^{8}$ Inf.U for the ratio of $GMC_{low\ dose\ level}/GMC_{release\ titer}$.

Alternative Hypothesis:

The EBOV GP-specific antibody GMC 21 days post-boost (following administration of Ad26.ZEBOV $2x10^{10}$ vp and MVA-BN-Filo $5x10^{7}$ Inf.U at a 56-day interval) is non-inferior to the GMC following administration of recently released batches of Ad26.ZEBOV $5x10^{10}$ vp and MVA-BN-Filo $1x10^{8}$ Inf.U using a 2/3 non-inferiority margin for the ratio of $GMC_{intermediate\ dose\ level}/GMC_{release\ titer}$.

The EBOV GP-specific antibody GMC 21 days post-boost (following administration of Ad26.ZEBOV $0.8x10^{10}$ vp and MVA-BN-Filo $5x10^{7}$ Inf.U at a 56-day interval) is non-inferior to the GMC following administration of recently released batches of Ad26.ZEBOV $5x10^{10}$ vp and MVA-BN-Filo $1x10^{8}$ Inf.U using a 2/3 non-inferiority margin for the ratio of $GMC_{low\ dose\ level}/GMC_{release\ titer}$.

To assess non-inferiority, hierarchical testing is applied: non-inferiority of the intermediate dose level will be shown if the 95% confidence interval (CI) of the ratio of the anti-EBOV GP ELISA GMC of Ad26.ZEBOV and MVA-BN-Filo at the intermediate dose level over Ad26.ZEBOV and MVA-BN-Filo at the release titer is entirely above the non-inferiority margin of 2/3. If non-inferiority is shown, then also for the low dose level, non-inferiority towards the release titer will be tested in the same way.

3. STUDY DESIGN AND RATIONALE

3.1. Overview of Study Design

This is a randomized, double-blind, placebo-controlled, parallel-group, multicenter, Phase 3 study to evaluate for a range of dose levels of Ad26.ZEBOV and MVA-BN-Filo to aid in establishment of end-expiry specifications using Ad26.ZEBOV at a dose of $5x10^{10}$, $2x10^{10}$ or $0.8x10^{10}$ vp as prime and MVA-BN-Filo at a dose of $1x10^{8}$ Inf.U or $5x10^{7}$ Inf.U as boost at a 56-day interval in healthy adult subjects in the US. The prime-boost regimen will only differ in the dose level of Ad26.ZEBOV (ie, $5x10^{10}$, $2x10^{10}$ or $0.8x10^{10}$ vp, respectively referred to as Groups 1, 2 and 3) or MVA-BN-Filo (ie, $1x10^{8}$ Inf.U [Group 1] or $5x10^{7}$ Inf.U [Groups 2 and 3]), while the timing of the boost vaccination (ie, 56 days after prime) and the sequence of vaccination will be identical.

Approximately 525 subjects will be enrolled and randomly assigned to one of the 4 groups: to one of 3 groups receiving Ad26.ZEBOV and MVA-BN-Filo (Groups 1 to 3) with approximately 150 subjects per group, or to a placebo group (Group 4) with approximately 75 subjects.

The subject population will consist of healthy men and women aged between 18 and 50 years (inclusive), who have not had prior exposure to Ebola virus (including travel to West Africa less than 1 month prior to screening) or a diagnosis of Ebola virus disease. Subjects who have received a candidate Ebola vaccine or an experimental candidate Ad26- or MVA-based vaccine in the past or with known allergy or history of anaphylaxis or other serious adverse reactions to

vaccines or vaccine products, including known allergy to egg, egg products and aminoglycosides will be excluded.

The subjects will be randomized at baseline (on Day 1) in a 2:2:2:1 ratio to Groups 1, 2, 3 and 4. Subjects will be randomized to receive the prime-boost regimen with either Ad26.ZEBOV and MVA-BN-Filo or placebo. Randomization will be stratified by site. A schematic overview of the study design and groups is provided in Table 1.

Table 1: Schematic Overview of Study Design and Groups

Group	N	Prime<br>Day 1	Boost<br>Day 57
1	150	Ad26.ZEBOV $5x10^{10}$ vp	MVA-BN-Filo $1x10^{8}$ Inf.U
2	150	Ad26.ZEBOV $2x10^{10}$ vp	MVA-BN-Filo $5x10^{7}$ Inf.U
3	150	Ad26.ZEBOV $0.8x10^{10}$ vp	MVA-BN-Filo $5x10^{7}$ Inf.U
4	75	Placebo (0.9% saline)	Placebo (0.9% saline)

N: planned number of subjects to receive study vaccine

Study-site personnel (except for those with primary responsibility for study vaccine preparation and dispensing), sponsor personnel and subjects will be blinded to the study vaccine allocation until the time of database lock for the primary analysis, when all subjects have completed the 6-month post-boost visit or discontinued earlier. Refer to Section 5 for details on blinding in case of interim analyses.

The study consists of a screening phase of up to 6 weeks (starting from the moment the subject signs the ICF). Subjects will be vaccinated at baseline (Day 1) and at Day 57, and will have follow-up visits at 7 and 28 days post-prime (Days 8 and 29), at 7, 21 and 42 days post-boost (Days 64, 78 and 99) and at 6 months post-boost (Day 237). All subjects will complete a 6-month post-boost visit (Day 237) to further assess safety and immunogenicity.

The baseline visit may be scheduled as soon as the results of all screening assessments are known (but should occur within 6 weeks from screening, see Section 9.1.2) and show that the subject is eligible for inclusion. The prime vaccination will occur on Day 1 (baseline), after the completion of all baseline assessments.

All subjects will receive the study vaccine (Ad26.ZEBOV and MVA-BN-Filo or placebo) IM in the deltoid muscle:

- Ad26.ZEBOV $5x10^{10}$ vp on Day 1, followed by a boost vaccination of MVA-BN-Filo $1x10^{8}$ Inf.U on Day 57 (release titer); *OR*
- Ad26.ZEBOV $2x10^{10}$ vp on Day 1, followed by a boost vaccination of MVA-BN-Filo $5x10^{7}$ Inf.U on Day 57 (intermediate dose level); *OR*
- Ad26.ZEBOV $0.8x10^{10}$ vp on Day 1, followed by a boost vaccination of MVA-BN-Filo $5x10^{7}$ Inf.U on Day 57 (low dose level); *OR*

- Placebo (0.9% saline) on Day 1, followed by a boost vaccination of placebo (0.9% saline) on Day 57.

Refer to Section 6 for further details on dosage and administration. After each vaccination, subjects will remain at the site for a total of 60 (±15) minutes post-vaccination to monitor for the development of any acute reactions, or longer if deemed necessary by the investigator.

The investigators, together with the sponsor's medical monitor, will be responsible for the safety monitoring of the study, and will halt vaccination of further subjects in case any of the pre-specified pausing rules described in Section 9.3.1 have been met. In addition, discontinuation of study vaccine should occur in any subject meeting the criteria outlined in Section 10.2. Criteria for postponement of vaccination at the scheduled time for vaccine administration and contraindications to boost vaccination have been defined and will be applied by the investigator (see Sections 6.2 and 6.3, respectively).

The investigator will collect samples for immunogenicity assessments (humoral and cellular assays) for the evaluation of primary, secondary and exploratory endpoints as planned by the sponsor (see Table 3 in Section 9.2) at the time points indicated in the Time and Events Schedule. Samples to assess humoral immune responses will be taken from all subjects; samples to assess cellular immune responses will be taken from subjects at selected site(s) with the capabilities to process peripheral blood mononuclear cells (PBMC) (targeted at 10% of all subjects). Subjects giving informed consent for the study will be informed that their leftover blood samples will be stored for potential future research. Subjects participating at selected site(s) where PBMC samples are collected will be asked explicitly to consent for potential future genetic research to be performed on PBMC samples. Subjects can withdraw consent for their samples to be used for future research at any time (see Section 16.2.5).

Safety will be assessed by collection of solicited local and systemic adverse events (reactogenicity), unsolicited adverse events, IREs and serious adverse events. The subjects will be closely observed by study-site personnel for the first 30 (±10) minutes after each vaccination and again at 60 (±15) minutes post-vaccination, and any unsolicited, solicited local or systemic adverse events will be documented during this period. Upon discharge from the site, subjects will receive a diary, a thermometer and a ruler to measure and record body temperature and solicited local reactions. Subjects will be instructed to record solicited local and systemic adverse events in the diary in the evening after each vaccination and then daily for the next 7 days at approximately the same time each day. Diaries should be completed at home by the subject. The investigator will document unsolicited adverse events from signing of the ICF onwards until 42 days post-boost, and serious adverse events and IREs from signing of the ICF onwards until the end of the study. The secondary safety endpoints are adverse events, serious adverse events, IREs, and solicited local and systemic adverse events, see Section 9.3.

Other safety assessments include vital signs (blood pressure, pulse/heart rate, and body temperature), physical examination and pregnancy testing at the time points indicated in the Time and Events Schedule.

The primary analysis will be performed when all subjects have completed the last study-related visit (ie, Day 237) or discontinued earlier.

Interim analyses may be performed (as detailed in Section 11.6) during the study for the purpose of informing future vaccine-related decisions in a timely manner. The results will not influence the conduct of the study in terms of early termination or later safety or immunogenicity endpoint assessments.

A DRC will be commissioned for this study. Refer to Section 11.7, Data Review Committee, for details.

Subjects who reach the Day 237 visit prior to unblinding will be approached to consent for enrollment into the VAC52150 Vaccine Development Roll-over study for long-term surveillance (for a total of up to 60 months after the prime vaccination) (see Section 9.1.4 for details). After unblinding, only subjects who received Ad26.ZEBOV and/or MVA-BN-Filo will remain in the VAC52150 Vaccine Development Roll-over study (VAC52150EBL4001) for long-term safety surveillance. After unblinding, subjects who received placebo and have already been enrolled into the VAC52150 Vaccine Development Roll-over study will be discontinued from further participation in the roll-over study.

3.2. Study Design Rationale

Control and Blinding

Randomization will be used to minimize bias in the assignment of subjects to groups, to increase the likelihood that known and unknown subject attributes (eg, demographic and baseline characteristics) are evenly balanced across groups, and to enhance the validity of possible statistical comparisons across groups.

Placebo recipients are included for blinding purposes and the assessment of safety, and will provide control specimens for immunogenicity assays. A placebo control will be used to establish the frequency and magnitude of changes in clinical safety endpoints that may occur in the absence of Ad26.ZEBOV and MVA-BN-Filo. The study vaccines (Ad26.ZEBOV and MVA-BN-Filo versus placebo) will be blinded to reduce potential bias during data collection and evaluation of clinical safety endpoints. Blinding will be guaranteed by preparation of study vaccine by unblinded qualified study-site personnel not involved in any other study-related procedure, and by the administration of vaccine in a masked syringe by a blinded study vaccine administrator (see Definitions of Terms).

Study Groups

The prime-boost regimen (Groups 1, 2 and 3) will only differ in the dose of Ad26.ZEBOV (ie, $5x10^{10}$, $2x10^{10}$ or $0.8x10^{10}$ vp, respectively referred to as Groups 1, 2 and 3) or MVA-BN-Filo (ie, $1x10^8$ Inf.U [Group 1] or $5x10^7$ Inf.U [Groups 2 and 3]), while the timing of the boost vaccination (ie, 56 days after prime) and the sequence of vaccination will be identical. The MVA-BN-Filo $1x10^8$ Inf.U dose corresponds to the dose of $1x10^8$ $TCID_{50}$ that is used in the current Phase 1 studies. A control group (Group 4) will be receiving placebo (prime and boost).

The safety, tolerability and immunogenicity of the regimen and different dose levels will be evaluated in the study.

Future Research

Subjects giving informed consent for the study will be informed that their leftover blood samples (serum and/or PBMC) will be stored for potential future research (see Section 16.2.5). Future scientific research may be conducted to further investigate Ebola vaccine- and disease-related questions. This may include the development of new or the improvement of existing techniques to characterize EBOV-directed immune responses or diagnostic tests. Subjects participating at selected site(s) where PBMC samples are collected will be asked explicitly to consent for potential future genetic research to be performed on PBMC samples. Subjects can withdraw consent for their samples to be used for future research at any time.

4. SUBJECT POPULATION

Screening for eligible subjects will be performed within 6 weeks before administration of the study vaccine on Day 1. Signing of the ICF needs to be done before the first study-related activity.

The inclusion and exclusion criteria for enrolling subjects in this study are described in the following 2 subsections. If there is a question about the inclusion or exclusion criteria below, the investigator must consult with the appropriate sponsor representative and resolve any issues before enrolling a subject in the study. Waivers are not allowed.

For a discussion of the statistical considerations of subject selection, refer to Section 11.2, Sample Size Determination.

4.1. Inclusion Criteria

Each potential subject must satisfy all of the following criteria to be enrolled in the study.

1. Signed an ICF indicating that he/she understands the purpose of, and procedures required for, the study and is willing to participate in the study.
2. Man or woman, between 18 and 50 years of age, inclusive, at randomization.
3. Healthy in the investigator’s clinical judgment on the basis of medical history, physical examination, ECG and vital signs performed at screening. If the results of these screening tests are outside the normal reference ranges, the subject may be included only if the investigator judges the abnormalities or deviations from normal to be not clinically significant. This determination must be recorded in the subject's source documents and initialed by the investigator.

4. Healthy on the basis of clinical laboratory tests performed at screening. If the results of the laboratory screening tests are outside the normal reference ranges, the subject may be included only if the investigator judges the abnormalities or deviations from normal to be not clinically significant. This determination must be recorded in the subject's source documents and initialed by the investigator.

   Note: The safety laboratory assessments at screening are to be performed within 28 days prior to the prime vaccination on Day 1 and may be repeated if they fall outside this time window.

   Note: In case of menstruation, urinalysis must be postponed but a result should be available before the prime vaccination.

   Note: If laboratory screening tests are out of range and deemed clinically significant, repeat of screening tests is permitted once using an unscheduled visit during the screening period to assess eligibility.

5. Before randomization, a woman must be either:

   - Of childbearing potential and practicing (or intending to practice) a highly effective method of birth control consistent with local regulations regarding the use of birth control methods for subjects participating in clinical studies, beginning at least 28 days prior to vaccination. The sponsor considers the following methods of birth control to be highly effective: established use of oral, injected or implanted hormonal methods of contraception; placement of an intrauterine device or intrauterine system; barrier methods: condom or occlusive cap (diaphragm or cervical/vault caps) with spermicidal foam/gel/film/cream/suppository; male partner sterilization (the vasectomized partner should be the sole partner for that subject); true abstinence (when this is in line with the preferred and usual lifestyle of the subject); *OR*
   - Not of childbearing potential: postmenopausal (>45 years of age with amenorrhea for at least 2 years or any age with amenorrhea for at least 6 months, and a serum follicle stimulating hormone (FSH) level >40 IU/L or mIU/mL); permanently sterilized (eg, bilateral tubal occlusion [which includes tubal ligation procedures as consistent with local regulations], hysterectomy, bilateral salpingectomy, bilateral oophorectomy); or otherwise be incapable of pregnancy.

     Note: If the social situation of a woman of childbearing potential changes (eg, woman who is not heterosexually active becomes active), she must begin a highly effective method of birth control, as described above.

6. Woman of childbearing potential must have a negative serum (β-human chorionic gonadotropin [β-hCG]) at screening and a negative urine β-hCG pregnancy test immediately prior to each study vaccine administration.

7. Man who is sexually active with a woman of childbearing potential and has not had a vasectomy performed more than 1 year prior to screening must be willing to use condoms for sexual intercourse beginning prior to enrollment, in addition to the birth control method used by the female partner.
8. Available and willing to participate for the duration of the study and follow-up visits.
9. Willing and able to comply with the protocol requirements, including the prohibitions and restrictions specified in Section 4.3.
10. Willing to provide verifiable identification.
11. Having a means to be contacted.

4.2. Exclusion Criteria

Any potential subject who meets any of the following criteria will be excluded from participating in the study.

1. Having received a candidate Ebola vaccine.
2. Diagnosed with Ebola virus disease, or prior exposure to Ebola virus, including travel to West Africa less than 1 month prior to screening. West Africa includes but is not limited to the countries of Guinea, Liberia, Mali, and Sierra Leone.
3. Having received an experimental candidate Ad26- or MVA-based vaccine in the past.

   <u>Note</u>: Receipt of any approved vaccinia/smallpox vaccine or experimental Ad-vector vaccine other than Ad26 at any time prior to study entry is allowed.
4. Known allergy or history of anaphylaxis or other serious adverse reactions to vaccines or vaccine products (including any of the constituents of the study vaccines [eg, polysorbate 80, ethylenediaminetetraacetic acid (EDTA) or L-histidine for Ad26.ZEBOV vaccine; and tris (hydroxymethyl)-amino methane (THAM) for MVA-BN-Filo vaccine]), including known allergy to egg, egg products and aminoglycosides.
5. Presence of acute illness (this does not include minor illnesses such as diarrhea or mild upper respiratory tract infection) or body temperature ≥38.0ºC on Day 1. Subjects with such symptoms will be excluded from enrollment at that time, but may be rescheduled for enrollment at a later date.
6. Positive hepatitis B surface antigen (HBsAg) or hepatitis C virus (HCV) antibody at screening.
7. HIV type 1 or type 2 infection.
8. Pregnant, breast-feeding, or planning to become pregnant while enrolled in this study until at least 3 months after the prime vaccination or up to 28 days after the boost vaccination (whichever takes longer).

9. Presence of significant conditions or clinically significant findings during screening of medical history, physical examination, ECG, vital signs or laboratory testing for which, in the opinion of the investigator, participation would not be in the best interest of the subject (eg, compromise the safety or well-being) or that could prevent, limit, or confound the protocol-specified assessments.

10. History of or underlying liver or renal insufficiency or significant cardiac, vascular, pulmonary (eg, persistent asthma), gastrointestinal, endocrine, neurologic, hematologic, rheumatologic, psychiatric, or metabolic disturbances.

11. History of malignancy other than squamous cell or basal cell skin cancer. However, subjects who underwent surgical excision, that is considered cured, can be enrolled.

12. Major surgery (per the investigator's judgment) within the 6 weeks prior to screening, or planned major surgery during the study (from the start of screening onwards).

13. Post-organ and/or stem cell transplant whether or not with chronic immunosuppressive therapy.

14. Received any disallowed therapies as noted in Section 8 before the planned first administration of the prime vaccine on Day 1.

15. Received an investigational drug or investigational vaccines or used an invasive investigational medical device within 3 months prior to screening, or current or planned participation in another clinical study during the study.

    Note: Participation in an observational clinical study is allowed.

16. Donation of a unit of blood within 12 weeks before Day 1 or plans to donate blood during participation in the study (from the start of screening onwards).

17. Receipt of blood products or immunoglobulin within 3 months prior to screening and during participation in the study.

18. Current or past abuse of alcohol, recreational or narcotic drugs, which in the investigator's opinion would compromise the subject's safety and/or compliance with the study procedures.

19. History of chronic urticaria (recurrent hives).

20. Unable to communicate reliably with the investigator.

21. Unlikely to adhere to the requirements of the study in the opinion of the investigator.

22. Employee of the investigator or study site, with direct involvement in the proposed study or other studies under the direction of that investigator or study site, as well as family members of the employees or the investigator.

23. Under legal guardianship or incapacitation.

NOTE: Investigators should ensure that all study enrollment criteria have been met at screening. If a subject's clinical status changes (including available laboratory results or receipt of additional medical records) after screening but before the prime vaccination on Day 1 such that

he/she no longer meets all eligibility criteria, then the subject should be excluded from further participation in the study. Section 17.4, Source Documentation, describes the required documentation to support meeting the enrollment criteria.

4.3. Prohibitions and Restrictions

Potential subjects must be willing and able to adhere to the following prohibitions and restrictions during the course of the study to be eligible for participation:

1. Woman of childbearing potential must remain on a highly effective method of birth control consistent with local regulations regarding the use of birth control methods for subjects participating in clinical studies (see inclusion criteria) until at least 3 months after the prime vaccination or up to 28 days after the boost vaccination (whichever takes longer). If the social situation of a woman of childbearing potential changes (eg, woman who is not heterosexually active becomes active), she must begin a highly effective method of birth control, as described above in Section 4.1, until at least 3 months after the prime vaccination or up to 28 days after the boost vaccination (whichever takes longer).

   Note: A period of 3 months after vaccination with Ad26.ZEBOV and 28 days after vaccination with MVA-BN-Filo should be respected.

   Note: Prior to each study vaccine administration, a urine pregnancy test should be performed for women of childbearing potential.

2. Woman must agree not to donate eggs (ova, oocytes) for the purposes of assisted reproduction from the start of screening onwards until at least 3 months after the prime vaccination or up to 28 days after the boost vaccination (whichever takes longer).

3. Man who has not had a vasectomy performed more than 1 year prior to screening and is sexually active with a woman of childbearing potential must use condoms for sexual intercourse until at least 3 months after the prime vaccination or up to 28 days after the boost vaccination (whichever takes longer), in addition to the birth control method used by the female partner, and must also not donate sperm from the start of the study onwards until at least 3 months after the prime vaccination or up to 28 days after the boost vaccination (whichever takes longer).

4. Woman should not breast-feed while enrolled in the study until at least 3 months after the prime vaccination or up to 28 days after the boost vaccination (whichever takes longer).

5. Not travel to epidemic Ebola areas while enrolled in the study from the start of screening onwards until the 42-day post-boost visit. Subjects who traveled after the 42-day post-boost visit to these areas should have returned at least 1 month before the Day 237 visit. Any traveling to epidemic Ebola areas should be documented in the case report form (CRF).

   Note: Subjects travelling to epidemic Ebola areas will be excluded from follow-up collection of blood for immunogenicity assessments if they contract Ebola virus disease (see also exclusion criterion #2 in Section 4.2.)

6. Not use any disallowed concomitant therapies as described in Section 8.

5. STUDY VACCINE ALLOCATION AND BLINDING

Study Vaccine Allocation

Central randomization will be implemented in this study. Subjects will be randomly assigned in a 2:2:2:1 ratio to Groups 1, 2, 3 and 4 (placebo), respectively, based on a computer-generated randomization schedule prepared before the study by or under the supervision of the sponsor. The randomization will be balanced by using randomly permuted blocks and will be stratified by site. The interactive web response system (IWRS) will assign a unique code, which will dictate the group assignment for the subject. The requestor must use his or her own user identification and personal identification number when contacting the IWRS, and will then give the relevant subject details to uniquely identify the subject.

Blinding

Study-site personnel, sponsor personnel and subjects will be blinded to the study vaccine allocation until the time of database lock for the primary analysis, when all subjects have completed the 6-month post-boost visit or discontinued earlier, except for unblinded qualified study-site personnel with primary responsibility for study vaccine preparation and dispensing, and not involved in any other study-related procedure. The study vaccines will be administered by a blinded study vaccine administrator (see Definitions of Terms).

In case interim analyses will be performed before the primary analysis, study-site personnel (except for those with primary responsibility for study vaccine preparation and dispensing), the sponsor (except programming, statistics, clinical and clinical immunology personnel and the sponsor committee involved in making future decisions for the program) and subjects will remain blinded to study vaccine allocation (see below).

The investigator will not be provided with randomization codes until the time of database lock for the primary analysis. The codes will be maintained within the IWRS, which has the functionality to allow the investigator to break the blind for an individual subject.

The blind should only be broken if specific emergency treatment/course of action would be dictated by knowing the group assignment of the subject. In such cases, the investigator may in an emergency determine the identity of the study vaccine by contacting the IWRS. It is

recommended that the investigator contact the sponsor or its designee if possible to discuss the particular situation, before breaking the blind. Telephone contact with the sponsor or its designee will be available 24 hours per day, 7 days per week. In the event the blind is broken, the sponsor must be informed as soon as possible. The date and reason for the unblinding must be documented by the IWRS, in the appropriate section of the CRF, and in the source document. The documentation received from the IWRS indicating the code break must be retained with the subject's source documents in a secure manner.

If the randomization code is broken by the investigator or the study-site personnel, the subject must discontinue further study vaccine administration and must be followed as appropriate (see Section 10.2 for details). If the randomization code is broken by the sponsor for safety reporting purposes, the subject should not discontinue further study vaccine administration and may remain in the study (if the randomization code is still blinded to the study-site personnel and the subject).

Data that may potentially unblind the study vaccine assignment (ie, study vaccine preparation/accountability data, or other specific laboratory data) will be handled with special care to ensure that the integrity of the blind is maintained and the potential for bias is minimized. This can include making special provisions, such as segregating the data in question from view by the investigators, clinical team, or others as appropriate until the time of database lock for the primary analysis and unblinding. The pharmacy and preparation of study vaccines will be monitored by an independent study vaccine monitor (see Section 17.8).

6. DOSAGE AND ADMINISTRATION

An overview of the study vaccines and dose levels in each group is provided in Table 1.

6.1. General Instructions and Procedures

All subjects will receive a vaccination, according to randomization, on Day 1 and on Day 57 at the following dose levels:

- Ad26.ZEBOV: $5x10^{10}$ vp supplied in a single use vial (0.5 mL extractable) (Group 1), $2x10^{10}$ vp (Group 2) or $0.8x10^{10}$ vp (Group 3);
- MVA-BN-Filo: $1x10^{8}$ Inf.U (nominal titer) supplied in a single use vial (0.5 mL extractable) (Group 1) or $5x10^{7}$ Inf.U (nominal titer) (Groups 2 and 3);
- Placebo: 0.9% saline, 0.5 mL (Group 4).

The Ad26.ZEBOV and MVA-BN-Filo dose levels used in Groups 2 and 3 will be prepared by dilution of the release titer (as detailed in Site Investigational Product Procedures Manual) by unblinded qualified study-site personnel with primary responsibility for study vaccine preparation and dispensing, and not involved in any other study-related procedure.

Ad26.ZEBOV and MVA-BN-Filo, or placebo, will be administered as 0.5-mL IM injections by a blinded study vaccine administrator. The injection site should be free from any injury, local skin conditions, or other issue that might interfere with the evaluation of local reactions

(eg, significant tattoo). In each subject, the boost vaccination should be administered in the opposite deltoid from the prime vaccination (unless the opposite arm has a condition that prevents evaluating the arm after injection) and it should be recorded in the CRF in which arm the vaccination has been administered. No local or topical anesthetic will be used prior to the injection.

Discontinuation of study vaccine administration should occur in any subject meeting the criteria outlined in Section 10.2. Criteria for postponement of vaccination at the scheduled time for vaccine administration and contraindications to boost vaccination have been defined in Sections 6.2 and 6.3, respectively. Refer to Section 9.3.1 for details on the pre-specified pausing rules to halt vaccination of further subjects.

After each vaccination, subjects will remain at the site for a total of 60 (±15) minutes post-vaccination to monitor for the development of any acute reactions, or longer if deemed necessary by the investigator. As with any vaccine, allergic reactions following vaccination with the study vaccine are possible. Therefore, appropriate drugs and medical equipment to treat acute anaphylactic reactions must be immediately available and a medically qualified member of study-site personnel trained to recognize and treat anaphylaxis must be present in the clinic during the entire vaccination procedure and post-vaccination monitoring period.

The investigator must provide emergency care as needed for any subject who experiences a life-threatening event. All sites will have facilities, equipment and the ability to manage an anaphylactic reaction. If additional therapy is required, the investigator will arrange for transport to the closest appropriate facility for continuing care.

The Site Investigational Product Procedures Manual specifies the maximum time that will be allowed between preparation and administration of the study vaccine. For storage conditions, please refer to Section 14.3.

6.2. Criteria for Postponement of Vaccination

A subject will not be vaccinated if he/she experiences any of the following events at the scheduled time of vaccination:

- Acute illness (this does not include minor illnesses such as diarrhea or mild upper respiratory tract infection);
- Fever (body temperature ≥38.0°C).

Subjects experiencing any of the events described above may be vaccinated up to 10 days beyond the window allowed for the scheduled vaccination, or be withdrawn from that vaccination at the discretion of the investigator and after consultation with the sponsor (see Section 10.2).

<u>Note</u>: In case the boost vaccination is postponed, the timing of the post-boost visits will be planned relative to the actual vaccination day (see Section 9.1.1).

6.3. Contraindications to Boost Vaccination

A subject will not be given the boost vaccination if he/she experiences any of the following events at any time after the prime vaccination and the sponsor's medical monitor will be notified immediately:

1. Anaphylaxis clearly attributable to vaccination with study vaccine; *OR*
2. Generalized urticaria within 72 hours of vaccination considered to be at least possibly related to study vaccine; *OR*
3. A serious adverse event considered to be at least possibly related to study vaccine; *OR*
4. Injection site ulceration, abscess or necrosis considered to be at least possibly related to study vaccine; *OR*
5. Any other safety concern threatening the subject's safety.

Subjects experiencing any of the events described above must not receive any further study vaccine, but should be monitored for safety and for immunogenicity according to the protocol as described in Section 10.2.

An ad hoc DRC meeting may be requested via the sponsor for any single event or combination of multiple events which are considered to jeopardize the safety of the subjects.

7. STUDY VACCINE COMPLIANCE

All study vaccines will be administered on site by a blinded study vaccine administrator (see Definitions of Terms). The date and time of each study vaccine administration will be recorded in the CRF.

8. PRESTUDY AND CONCOMITANT THERAPY

Prestudy therapies administered up to 30 days prior to the start of screening and previous vaccinia/smallpox vaccination at any time prior to study entry must be recorded in the CRF.

Concomitant therapies must be recorded from screening onwards until the 42-day post-boost visit. Concomitant therapies should also be recorded after the 42-day post-boost visit but only if given in conjunction with serious adverse events and IREs that meet the criteria outlined in Sections 12.3.2 and 12.3.3, respectively.

All therapies (prescription or over-the-counter medications, including vaccines, vitamins, herbal supplements; non-pharmacologic therapies such as electrical stimulation, acupuncture, special diets, exercise regimens) must be recorded in the CRF. Recorded information will include a description of the type of the drug, treatment period, dosing regimen, route of administration, and its indication.

Subjects must use adequate birth control measures prior to randomization as described in Section 4.

Subjects are allowed to receive all routine immunizations according to local schedules, taking into consideration the following restrictions:

- Inactivated vaccines should be administered at least 15 days before or after administration of any study vaccine to avoid any potential interference in efficacy of the routine immunizations or the interpretation of immune responses to study vaccines, as well as to avoid potential confusion with regard to attribution of adverse events.
- Live attenuated vaccines are prohibited in the period from 30 days before baseline (Day 1) to 30 days after the boost vaccination.

However, if a vaccine is indicated in a post-exposure setting (eg, rabies or tetanus), it must take priority over the study vaccine.

Analgesic/antipyretic medications and non-steroidal anti-inflammatory drugs may be used post-vaccination only in case of medical need (eg, body temperature ≥38.5°C or pain) and their use must be documented. Use of these medications as routine prophylaxis prior to study vaccine administration is prohibited.

Chronic or recurrent use of medications that modify the host immune response (eg, cancer chemotherapeutic agents, systemic corticosteroids, immunomodulators) are prohibited.

The sponsor must be notified in advance (or as soon as possible thereafter) of any instances in which prohibited therapies are administered. Prohibited therapies will be captured as protocol deviations.

9. STUDY EVALUATIONS

9.1. Study Procedures

9.1.1. Overview

The Time and Events Schedule summarizes the frequency and timing of safety, tolerability and immunogenicity measurements and evaluations applicable to this study. Details for all study procedures are provided in the following sections. Additional unscheduled study visits may be required if, in the investigator's opinion, further clinical or laboratory evaluation is needed.

Visit Windows

The screening visit has to be performed within 6 weeks prior to the baseline visit (ie, the day of the subject's prime vaccination, Day 1). If a subject did not receive study vaccine on the planned day of vaccination, the timings of the next visits post-vaccination (see Time and Events Schedule) will be determined relative to the actual day of vaccination. Visit windows that will be allowed are summarized in Table 2. The subject should be encouraged to come in the exact day planned and use the visit window only if absolutely necessary.

Table 2: Visit Windows

Visit Description	Day	Window
Seven Days Post-prime Vaccination	Day 8	±2 days
Twenty-eight Days Post-prime Vaccination	Day 29	±3 days
Boost Vaccination	Day 57	±3 days
Seven Days Post-boost Vaccination	Day 64	±2 days
Twenty-one Days Post-boost Vaccination	Day 78	±3 days
Forty-two Days Post-boost Vaccination	Day 99	±3 days
Six Months Post-boost Vaccination	Day 237	±15 days

Blood Sampling Volumes

Approximately 280 mL of blood (or 600 mL including PBMC samples), excluding the blood volume for clinical laboratory testing at screening, will be drawn over a period of around 8 months, and remains well below the limits of standard blood donation.

Additional serum or urine pregnancy tests may be performed, as determined necessary by the investigator or required by local regulation, to establish the absence of pregnancy at any time during the subject's participation in the study.

Repeat or unscheduled samples may be taken for safety reasons or for technical issues with the samples.

9.1.2. Screening Phase

Up to 6 weeks before baseline (the day of the subject’s prime vaccination, Day 1) and after signing and dating the ICF (see Section 16.2.3), screening assessments will be performed as indicated in the Time and Events Schedule. Screening may be split into multiple days or visits. In exceptional cases, the screening phase can be extended if discussed with and approved (documented) by the sponsor, eg, if not all the test results become available during the allocated 6 weeks; this will be evaluated on a case-by-case basis. Screening results from study VAC52150EBL3003 (eg, laboratory and ECG data) can be used if obtained within 6 weeks before the prime vaccination in the current study.

For men and women of non-childbearing potential, there will be no minimum duration of the screening period and it will last only for the time required to verify eligibility criteria. For women of childbearing potential, it should be confirmed that adequate birth control measures were used from at least 28 days before the prime vaccination with a negative serum β-hCG pregnancy test at screening and a negative urine test immediately prior to each study vaccination (see Section 4). All men and women, except for women of non-childbearing potential, will be asked to use adequate birth control for sexual intercourse until at least 3 months after the prime vaccination or up to 28 days after the boost vaccination (whichever takes longer) (see Section 4.3).

Only subjects complying with the criteria specified in Section 4 will be included in the study. The investigator will provide detailed information on the study to the subject and will obtain written informed consent prior to each subject’s study participation. The procedures indicated in

the Time and Events Schedule will only be performed after the subject's written informed consent has been obtained.

The following is performed to determine eligibility requirements as specified in the inclusion and exclusion criteria:

- Review of all inclusion and exclusion criteria;
- Review of medical history (including concomitant diseases) and demographics;
- Review of prestudy therapies (up to 30 days prior to the start of screening), previous vaccinia/smallpox vaccination if known (at any time prior to study entry) and concomitant therapies;
- Serum pregnancy test (for women of childbearing potential);
- Blood sampling for hematology and chemistry (fasting or non-fasting);
- Urine sampling for urinalysis (dipstick);
- Serology testing (HIV type 1 or type 2, hepatitis B, hepatitis C);
- FSH assessment (for women >45 years of age with amenorrhea for less than 2 years or at any age with amenorrhea for more than 6 months);
- ECG recording;
- Full physical examination (including height and body weight; a genitourinary examination is not required);
- Measurement of vital signs (blood pressure, pulse/heart rate, body temperature).

All adverse events, serious adverse events, IREs and pregnancies will be collected from the time a signed and dated ICF is obtained.

The overall eligibility of the subject to participate in the study will be assessed once all screening values and results of any other required evaluations are available. Retesting of values (eg, safety laboratory) that lead to exclusion is allowed once using an unscheduled visit during screening to assess eligibility. The safety laboratory assessments are to be performed within 28 days prior to the prime vaccination and may be repeated if they fall outside this time window. Study subjects who qualify for inclusion will be contacted and scheduled for enrollment and prime vaccination within 6 weeks.

9.1.3. Study Visits

Day 1 Visit (Prime Vaccination)

If eligible, the subject will come for the baseline visit (Day 1). The investigator should ensure that all enrollment criteria have been met during screening. If a subject's clinical status changes (including available laboratory results or receipt of additional medical records) after screening but before the prime vaccination (Day 1) such that the subject no longer meets all enrollment criteria, then the subject should be excluded from further participation in the study. If the initial

laboratory sampling occurred more than 6 weeks before baseline (Day 1), sampling will need to be repeated.

A urine pregnancy test (for women of childbearing potential), a symptom-directed physical examination (defined in Section 9.3) as indicated by the investigator, and measurements of vital signs will be performed. The eligible subjects will be allocated (by central randomization) to a study group as described in Section 5 and will receive the prime vaccination via IM injection as described in Section 6, unless any of the pre-specified criteria not to proceed with vaccination are met (refer to Sections 6.2 and 10.2 for details) or if a pause for vaccination of further subjects has been installed (see Section 9.3.1).

Study vaccine will be prepared on-site by unblinded qualified study-site personnel not involved in any other study-related procedure who will place a blinding tape on the syringe to mask its content and send the study vaccine to a blinded study vaccine administrator (see Definitions of Terms) for administration to the subject (see Section 14.3 for details). Refer to Section 6 for further details on dosage and administration and post-vaccination monitoring.

Subjects will have blood drawn for immunogenicity assessments as specified in the Time and Events Schedule. Refer to Section 9.2 for further details on the immunogenicity evaluations.

All adverse events, serious adverse events and pregnancies will be collected and documented on the CRF, together with the information on any concomitant therapies. For reporting of IREs, refer to Section 12.3.3.

Upon discharge from the site, subjects will be provided with a diary, a thermometer and a ruler to measure and record body temperature and local solicited adverse events. Subjects will also record solicited local and systemic adverse events (reactogenicity) in the diary in the evening after vaccination and then daily for the next 7 days at approximately the same time each day. Diaries should be completed at home by the subject. Subjects will be instructed to contact the investigator immediately in case they experience an unsolicited adverse event (not listed on the diary card) or for any severe (grade 3) solicited adverse event (listed on the diary card).

Days 8 and 29 Visits

Subjects will come to the site at 7 and 28 days after the prime vaccination as indicated in the Time and Events Schedule. The investigator will examine the injection site for occurrences of erythema, induration/swelling, pain/tenderness or itching at this visit in order to complete the relevant parts of the CRF. The subject's diary will be reviewed by study-site personnel at the 7-day post-prime visit (Day 8). A symptom-directed physical examination (defined in Section 9.3) will be performed as indicated by the investigator.

Subjects will have blood drawn for immunogenicity assessments as specified in the Time and Events Schedule. Refer to Section 9.2 for further details on the immunogenicity evaluations.

All adverse events, serious adverse events and pregnancies will be collected and documented on the CRF, together with the information on any concomitant therapies. For reporting of IREs, refer to Section 12.3.3.

Day 57 Visit (Boost Vaccination)

The subjects will receive the boost vaccination via IM injection as described in Section 6, unless any of the pre-specified criteria not to proceed with vaccination are met (refer to Sections 6.2, 6.3 and 10.2 for details) or if a pause for vaccination of further subjects has been installed (see Section 9.3.1).

Before the boost vaccination, a urine pregnancy test (for women of childbearing potential), a symptom-directed physical examination (defined in Section 9.3) as indicated by the investigator, and measurements of vital signs will be performed.

Subjects will have blood drawn for immunogenicity assessments as specified in the Time and Events Schedule. Refer to Section 9.2 for further details on the immunogenicity evaluations.

The same procedures are applicable as for the prime vaccination regarding vaccine preparation; collection of adverse events, serious adverse events, IREs and pregnancies, together with the information on concomitant therapies; and procedures for subjects after discharging.

Days 64, 78 and 99 Visits

Subjects will come to the site at 7, 21 and 42 days after the boost vaccination as indicated in the Time and Events Schedule. The investigator will examine the injection site for occurrences of erythema, induration/swelling, pain/tenderness or itching at these visits in order to complete the relevant parts of the CRF. The subject's diary will be reviewed by study-site personnel at the 7-day post-boost visit (Day 64). A symptom-directed physical examination (defined in Section 9.3) will be performed as indicated by the investigator.

Subjects will have blood drawn for immunogenicity assessments at the specified visits in the Time and Events Schedule. Refer to Section 9.2 for further details on the immunogenicity evaluations.

All adverse events, serious adverse events and pregnancies will be collected and documented on the CRF, together with the information on any concomitant therapies. For reporting of IREs, refer to Section 12.3.3.

Subjects will be instructed to contact the investigator before the next visit (ie, on Day 237) if they experience any adverse event or intercurrent illness that they perceive as relevant and/or can be possibly related to study vaccine in their opinion.

Day 237 Visit

All subjects will complete a 6-month post-boost visit (Day 237). A symptom-directed physical examination (defined in Section 9.3) will be performed as indicated by the investigator. When all subjects have had their 6-month post-boost visit (Day 237) or discontinued earlier and the database has been locked for the primary analysis, the study will be unblinded.

Subjects will have blood drawn for immunogenicity assessments on Day 237. Refer to Section 9.2 for further details on the immunogenicity evaluations.

Serious adverse event and IRE information will be collected until the end of the study, and concomitant therapies should only be recorded if given in conjunction with serious adverse events and IREs. Pregnancies will be reported until the end of the study.

9.1.4. VAC52150 Vaccine Development Roll-over Study

Subjects who reach the Day 237 visit prior to unblinding will be approached to consent for enrollment into the VAC52150 Vaccine Development Roll-over study for long-term surveillance (for a total of up to 60 months after the prime vaccination). After unblinding, only subjects enrolled in this study who received Ad26.ZEBOV or MVA-BN-Filo will remain in the VAC52150 Vaccine Development Roll-over study for long-term safety surveillance. After unblinding, subjects who received placebo and have already been enrolled into the VAC52150 Vaccine Development Roll-over study will be discontinued from further participation in the roll-over study. The parent(s)/legal guardian of children born to vaccinated female subjects who became pregnant with estimated conception within 28 days after vaccination with MVA-BN-Filo or within 3 months after vaccination with Ad26.ZEBOV, will also be approached to consent for enrollment of their offspring into the roll-over study, according to the same rules that apply for the other subjects.

9.2. Immunogenicity Evaluations

Blood samples for immunogenicity assessments will be collected at the time points and in the volumes indicated in the Time and Events Schedule. Samples for assessment of humoral immune responses will be obtained from all subjects. Samples for assessment of cellular immune responses will be obtained from subjects at selected site(s) with the capabilities to process PBMC (targeted at 10% of all subjects). Table 3 provides an overview of the immunogenicity assessments (humoral and cellular assays).

Table 3: Overview of Immunogenicity Assessments

Sample	Immunogenicity Assessments (non-exhaustive)	Assays (non-exhaustive)
Serum, all subjects	Analysis of antibodies binding to EBOV GP<br>Analysis of neutralizing antibodies to EBOV GP<br>Analysis of binding and/or neutralizing antibodies to adenovirus and/or MVA<br>Analysis of anti-EBOV GP antibody characteristics, including IgG subtyping	ELISA (EU/mL)<br>Virus neutralization assay<br>Adenovirus and/or MVA ELISA and/or neutralization assay<br>Molecular antibody characterization
PBMC, subjects at selected site(s)[a]	T-cell IFN-γ responses to EBOV GP<br>Analysis of T-cell responses to EBOV GP (including CD4/8, IL-2, IFN-γ, TNF-α and/or activation markers)	ELISpot<br>ICS

ELISA: enzyme-linked immunosorbent assay; ELISpot: enzyme-linked immunospot; ICS: intracellular cytokine staining; IFN: interferon; IgG: immunoglobulin G; IL: interleukin; TNF: tumor necrosis factor

[a] with the capabilities to process PBMC (targeted at 10% of all subjects).

9.3. Safety Evaluations

The study will include the following evaluations of safety and tolerability as described below and according to the time points provided in the Time and Events Schedule. Any clinically significant abnormalities in any of the safety assessments occurring from signing of the ICF onwards until 42 days after the boost vaccination must be recorded on the Adverse Event section of the CRF. Thereafter, reporting will be limited to all serious adverse events and IREs. Any clinically significant abnormalities (including those persisting at the end of the study/early withdrawal) will be followed by the investigator until resolution or until a clinically stable endpoint is reached (see Section 12).

The investigators, together with the sponsor's medical monitor, will be responsible for the safety monitoring of the study, and will halt vaccination of further subjects in case any of the pre-specified pausing rules described in Section 9.3.1 have been met. Further safety measures with regards to vaccination are described in Sections 6.2 and 6.3.

A DRC will be established by the sponsor before the start of the study and will convene to review the available safety data in case a pausing rule is met (as described in Section 9.3.1). Details regarding the DRC are provided in Section 11.7.

Adverse Events

All adverse events, whether serious or non-serious, will be collected at all visits from signing of the ICF onwards until 42 days post-boost. Thereafter, reporting will be limited to all serious adverse events and IREs up to the subject's last study-related procedure. Solicited local and systemic adverse events (reactogenicity, see below) will be reported by the subject until 7 days after each administration of study vaccine. Adverse events will be followed by the investigator as specified in Section 12, Adverse Event Reporting.

Solicited Adverse Events

Solicited adverse events are precisely defined events that subjects are specifically asked about and which are noted by subjects in the diary. The subjects will be closely observed by study-site

personnel for the first 30 (±10) minutes after each administration of study vaccine and again at 60 (±15) minutes post-vaccination, and any unsolicited, solicited local or systemic adverse events will be documented during this period. Upon discharge from the site, subjects will receive a diary, a thermometer and a ruler to measure and record body temperature and solicited local reactions. Subjects will be instructed to record solicited local and systemic adverse events in the diary in the evening after each administration of study vaccine and then daily for the next 7 days (until Day 8) at approximately the same time each day to serve as a reminder to the subject for the next visit. On Day 8, the diary needs to be completed on site before the subject leaves the site. The investigator should discuss the information from the diary with the subject, document the relevant information in the clinic chart, and complete the relevant parts of the CRF as described in the CRF Completion Guidelines.

On-site and diary reported solicited adverse events will be captured on a separate CRF page as described in the CRF Completion Guidelines, in contrast to the unsolicited adverse events which will be reported on the Adverse Event page of the CRF. The investigator must record in the CRF his/her opinion concerning the relationship of the adverse event to study vaccine.

Solicited Local (Injection Site) Adverse Events

Subjects will also be instructed on how to note occurrences of erythema, induration/swelling (measured using the ruler supplied), pain/tenderness and itching at the injection site in the evening after each administration of study vaccine and then daily for the next 7 days in the diary at approximately the same time each day.

Solicited Systemic Adverse Events

Subjects will be instructed on how to record daily oral body temperature using a thermometer provided for home use. Subjects should record the body temperature in the evening after each vaccination and then daily for the next 7 days in the diary. Body temperature should be measured at approximately the same time each day. If more than one measurement is made on any given day, the highest value will be recorded in the CRF.

Subjects will also be instructed on how to note the following symptoms in the evening after each administration of study vaccine and then daily for the next 7 days in the diary at approximately the same time each day:

- Nausea/vomiting
- Headache
- Myalgia
- Arthralgia
- Fatigue/malaise
- Chills

If a ***solicited local or systemic adverse event*** is not resolved on Day 8, the follow-up will be captured on the diary. The subject will be instructed to record the date of last symptoms and maximum severity in the diary after resolution.

Cardiac Events

In case any cardiac sign or symptom develops after the boost vaccination, an ECG and troponin I test should be obtained and the subject should be referred to a local cardiologist.

Clinical Laboratory Tests

Blood samples (~15 mL in total) for serum chemistry and hematology, serology (HIV-1/2, hepatitis B/C) and pregnancy testing and a urine sample for urinalysis (dipstick) will be collected at screening. Samples can be taken in fasting or non-fasting conditions but should be documented in the CRF. The investigator must review the laboratory results, document this review and record any clinically relevant changes occurring during the study in the Adverse Event section of the CRF. The laboratory reports must be filed with the source documents. For reporting of IREs, refer to Section 12.3.3.

The following tests will be performed by the central laboratory and will only be measured at screening:

- Hematology Panel

  - hemoglobin
  - white blood cell (WBC) count with differential
  - platelet count

  Blood smear: A WBC evaluation may include any abnormal cells, which will then be reported by the laboratory. A hematology evaluation may include abnormalities in the red blood cell (RBC) count and/or RBC parameters and/or RBC morphology, which will then be reported by the laboratory. In addition, any other abnormal cells in a blood smear will also be reported. Only clinically significant abnormal WBC, abnormal RBC, or any other abnormal cells in a blood smear will be reported as adverse events.

- Serum Chemistry Panel

  - sodium
  - potassium
  - blood urea nitrogen
  - aspartate aminotransferase (AST)
  - alanine aminotransferase (ALT)
  - total bilirubin
  - creatinine
  - FSH (only in women >45 years of age with amenorrhea <2 years or at any age with amenorrhea >6 months)

- Urinalysis – Dipstick for:

  - glucose
  - ketones
  - protein
  - blood

  In case of positive urinalysis dipstick results, the sediment will be examined microscopically (only RBC will be documented).

Additional clinical laboratory assessments to be performed are as follows:

- Serum pregnancy test for women of childbearing potential at screening;
- Urine pregnancy test for women of childbearing potential before each study vaccination;

- Serology (HIV type 1 and type 2 antibody, HBsAg, and HCV antibody) at screening.

Any abnormal laboratory values will be graded according to the toxicity grading tables in Attachment 1 if applicable for laboratory tests.

Electrocardiogram (ECG)

A single, 12-lead ECG will be performed at screening and interpreted locally. Additional ECG monitoring may be done at other time points during the study if clinically indicated based on signs and symptoms.

During the collection of ECGs, subjects should be in a quiet setting without distractions (eg, television, cell phones). Subjects should rest in a supine position for at least 5 minutes before ECG collection and should refrain from talking or moving arms or legs. If blood sampling or vital sign measurement is scheduled for the same time point as ECG recording, the procedures should be performed in the following order: vital signs, ECG(s), blood draw.

Vital Signs (blood pressure, pulse/heart rate, body temperature)

Vital sign measurements will be performed at the time points indicated in the Time and Events Schedule. Blood pressure and pulse/heart rate measurements will be assessed (at rest in supine position) with a completely automated device. Manual techniques will be used only if an automated device is not available. Confirmatory measurements can be performed if inconsistent with a prior measurement. Body temperature is preferably measured orally. If the body temperature was measured at another site this needs to be captured in the CRF.

Physical Examination

A full physical examination, including height and body weight, will be performed by the investigator at screening. A full physical examination includes the following: general appearance, eyes, ears, nose, throat, cardiovascular system, respiratory system, gastrointestinal system, and skin and mucous membranes. A neurological and musculoskeletal examination as well as an examination of the lymph nodes will also be performed. The height should be measured barefooted at the screening visit. To obtain the actual body weight, subjects must be weighed lightly clothed.

After screening, an abbreviated, symptom-directed physical examination will be performed as indicated by the investigator based on any clinically relevant issues, clinically relevant symptoms and medical history. The symptom-directed physical examination may be repeated at other visits if deemed necessary by the investigator. An abbreviated, symptom-directed physical examination may include inspection of the vaccination site(s).

9.3.1. Pausing Rules

The investigators and the sponsor's medical monitor will review the blinded safety data of enrolled subjects on an ongoing basis. The sponsor's medical monitor will be involved in all discussions and decisions.

If any of the following events occur in any subject who received at least one dose of study vaccine in the study (at any site), that site investigator will halt the vaccination of further subjects and the sponsor's medical monitor will be notified immediately. The sponsor's medical monitor will then also inform all the other investigators to halt further vaccination as well.

1. Death in any subject considered at least possibly related to study vaccine; *OR*
2. An anaphylactic reaction within 24 hours of vaccination or the presence of generalized urticaria within 72 hours of vaccination in any subject considered at least possibly related to study vaccine; *OR*
3. A life-threatening or other serious adverse event in any subject considered at least possibly related to study vaccine.

For the events described above, the sponsor's medical monitor notifies the DRC immediately and dosing will be halted. Within 3 business days, the DRC will convene to review the available safety data as outlined in the charter and to advise whether vaccination may resume, or additional safety data are needed, eventually ask for a protocol amendment, or discontinue further vaccination or suspend the study. The sites will be allowed to resume activities upon receipt of a written notification from the sponsor. The communications from the DRC will be forwarded by the investigator to the Independent Ethics Committee (IEC)/Institutional Review Board (IRB) according to local standards and regulations and health authorities will be notified of any pause and the DRC recommendation.

Any event that meets the criteria of a serious adverse event should be recorded on the Serious Adverse Event page of the CRF (see Section 12.3.2).

9.4. Vaccine-induced Seropositivity

In general, uninfected subjects who participate in Ebola vaccine studies may develop Ebola-specific antibodies as a result of an immune response to the candidate Ebola vaccine, referred to as VISP. These antibodies may be detected in Ebola serologic tests, causing the test to appear positive even in the absence of actual Ebola infection. VISP may become evident during the study, or after the study has been completed. The potential of a study participant becoming PCR-positive after vaccination is being assessed in a Phase 1 study (VAC52150EBL1002).

Subjects should not donate blood during participation in the study (from the start of screening onwards; see Section 4.2).

Consent will be obtained to contact the doctors that the subject sees regularly, to let them know that the subject is taking part in this study. It is important for all of the subject's doctors to know that the subject may be administered experimental vaccines. Subjects participating in the study will be provided with a "wallet (study) card" and instructed to carry this card with them for the duration of the study (see Section 12.3.1).

9.5. Sample Collection and Handling

The actual dates and times of sample collection must be recorded in the CRF or laboratory requisition form. Refer to the Time and Events Schedule for the timing and frequency of all sample collections.

Sample collection and processing will be performed by the study-site personnel according to current versions of approved standard operating procedures.

Instructions for the collection, handling, storage, and shipment of samples will be provided in the Laboratory Manual. Collection, handling, storage, and shipment of samples must be under the specified, and where applicable, controlled temperature conditions as indicated in the Laboratory Manual.

10. SUBJECT COMPLETION/DISCONTINUATION OF STUDY VACCINATION/WITHDRAWAL FROM THE STUDY

10.1. Completion

A subject will be considered to have completed the study if he or she has completed all assessments at the 6-month post-boost visit.

10.2. Discontinuation of Study Vaccination/Withdrawal From the Study

Discontinuation of Study Vaccination

If a subject's study vaccine must be discontinued before the end of the vaccination schedule, this will not result in automatic withdrawal of the subject from the study.

A subject's study vaccine (prime or boost) may be discontinued at the discretion of the investigator and after consultation with the sponsor for any of the events in Section 6.2.

A subject's study vaccine should be **permanently** discontinued if:

- The investigator believes that for safety reasons (eg, adverse event) it is in the best interest of the subject to withhold from further administration of study vaccines;
- The subject or the partner of a male subject becomes pregnant;
- The subject has confirmed Ebola virus disease through natural exposure to the virus (eg, by travel to an affected country);
- The subject experiences any of the events described in Section 6.3;
- The randomization code is broken by the investigator or the study-site personnel.

Subjects meeting any of the reasons listed above must not receive any further study vaccine, but should continue to be monitored for safety and for immunogenicity according to the protocol if this does not result in safety risks for the subject. In case of early discontinuation of study vaccine due to an adverse event, the investigator will collect all information relevant to the

adverse event and safety of the subject, and will follow the subject to resolution, or until reaching a clinically stable endpoint.

Withdrawal From the Study

Each subject has the right to withdraw from the study at any time for whatever reason. The investigator should make an attempt to contact subjects who did not return for scheduled visits or follow-up. Although the subject is not obliged to give reason(s) for withdrawing early, the investigator should make a reasonable effort to ascertain the reason(s) while fully respecting the subject's rights.

A subject will be withdrawn from the study for any of the following reasons:

- Decision by the investigator to withdraw a subject for repeated failure to comply with protocol requirements;
- Decision by the sponsor to stop or cancel the study;
- Decision by local regulatory authorities and IEC/IRB to stop or cancel the study;
- Lost to follow-up;
- Withdrawal of consent;
- Death.

If a subject withdraws early from the study for any of the reasons listed above (except in case of death), early withdrawal assessments should be obtained per the assessments for the 42-day post-boost visit, with the exception of the immunogenicity assessments. A subject who wishes to withdraw consent from participation in the study will be offered an optional visit for safety follow-up (before formal withdrawal of consent), but the subject has the right to refuse.

If a subject is lost to follow-up, every reasonable effort must be made by the study-site personnel to contact the subject and determine the reason for discontinuation/withdrawal. The measures taken to follow up must be documented.

When a subject withdraws before completing the study, the reason for withdrawal is to be documented in the CRF and in the source document. Study vaccine assigned to the withdrawn subject may not be assigned to another subject. For subjects who withdraw from the study after randomization but before the prime vaccination, an additional subject will be enrolled. Subjects who withdraw from the study after receiving the prime vaccination will not be replaced.

10.3. Withdrawal From the Use of Research Samples

A subject who withdraws from the study will have the following options for storage of samples for potential future use:

- The collected samples will be retained and used in accordance with the subject's original informed consent for storage of samples for future use.

- The subject may withdraw consent for storage of samples for potential future use (see Section 16.2.5), in which case the samples will be destroyed and no further testing will take place. To initiate the sample destruction process, the investigator must notify the sponsor study site contact of withdrawal of consent for the storage of leftover samples for future research and request sample destruction. The sponsor study site contact will, in turn, contact the biomarker representative to execute sample destruction. If requested, the investigator will receive written confirmation from the sponsor that the samples have been destroyed. Details of the sample retention for research are presented in the ICF.

Withdrawal From Storage of Samples for Future Use While Remaining in the Study

The subject may withdraw consent for storage of samples for future use (refer to Section 16.2.5) while remaining in the study. In such a case, the samples will be destroyed after they are no longer needed for the clinical study as described above. Details of the sample retention for research are presented in the ICF.

11. STATISTICAL METHODS

Statistical analysis will be done by the sponsor or under the authority of the sponsor. A general description of the statistical methods to be used to analyze the safety and immunogenicity data is outlined below. Specific details will be provided in the Statistical Analysis Plan (SAP).

The primary analysis will be conducted when all subjects have completed the 6-month post-boost visit or discontinued earlier. Refer to Section 5 for details on blinding.

Interim analyses may be performed as described in Section 11.6.

11.1. Analysis Sets

The Full Analysis set includes all subjects who were randomized and received at least 1 dose of study vaccine, regardless of the occurrence of protocol deviations. Safety data will be analyzed based on the Full Analysis set.

The Immunogenicity Analysis set includes all randomized and vaccinated subjects, who have data from baseline and at least 1 post-vaccination immunogenicity blood draw.

The Per Protocol Analysis set includes all randomized and vaccinated subjects, who received both the prime and boost vaccinations (administered not more than 10 days outside the visit window), have immunogenicity data from baseline and at least 1 post-vaccination evaluable immunogenicity sample, and have no major protocol violations influencing the immune response.

For assessment of the primary objective, the analysis population is the Per Protocol Analysis set. The analysis of the primary endpoint will also be based on the Immunogenicity Analysis set to evaluate the robustness of the analysis results. For all other analyses of immune response, the analysis population is the Immunogenicity Analysis set.

11.2. Sample Size Determination

Sample size calculations are performed under the following assumptions:

- Immune response is measured by binding antibody levels against the EBOV GP (using ELISA) at 21 days post-boost (following an Ad26.ZEBOV prime and MVA-BN-Filo boost after 56 days).
- A standard deviation of 0.303 at the log_{10} scale (21 days post-boost, following an Ad26.ZEBOV prime and MVA-BN-Filo boost after 56 days) in Groups 1 to 3.
- GMC of intermediate dose level or low dose level = 0.9xGMC of release titer.
- Hierarchical testing will be applied, such that non-inferiority of the intermediate dose level towards the release titer is tested first. If non-inferiority is shown, then also for the low dose level, non-inferiority towards the release titer will be tested.

A total of 150 subjects per group receiving Ad26.ZEBOV as prime and MVA-BN-Filo as boost are needed to have an overall power of at least 90% to show:

(1) non-inferiority in immune response after vaccination with Ad26.ZEBOV $2x10^{10}$ vp as prime and MVA-BN-Filo $5x10^{7}$ Inf.U as boost 56 days later, compared to vaccination with recently released batches of Ad26.ZEBOV $5x10^{10}$ vp as prime and MVA-BN-Filo $1x10^{8}$ Inf.U as boost 56 days later; and

(2) subsequently, if non-inferiority is shown for (1), to show non-inferiority in immune response after vaccination with Ad26.ZEBOV $0.8x10^{10}$ vp as prime and MVA-BN-Filo $5x10^{7}$ Inf.U as boost 56 days later, compared to vaccination with recently released batches of Ad26.ZEBOV $5x10^{10}$ vp as prime and MVA-BN-Filo $1x10^{8}$ Inf.U as boost 56 days later.

The sample size calculation takes a 10% overall dropout rate into account. Adding a control group of approximately 75 subjects receiving placebo, the total sample size will be approximately 525 subjects.

Non-inferiority of the intermediate dose level will be shown if the 95% CI of the ratio of the anti-EBOV GP ELISA GMC of Ad26.ZEBOV and MVA-BN-Filo at the intermediate dose level over Ad26.ZEBOV and MVA-BN-Filo at the release titer is entirely above the non-inferiority margin of 2/3. This non-inferiority margin has been used in the development of other vaccines for which no correlate of protection has been established. If non-inferiority is shown, then also for the low dose level, non-inferiority towards the release titer will be tested in the same way.

11.3. Subject Information

For all subjects, demographic characteristics (eg, age, height, weight, body mass index, race and sex) and screening/baseline characteristics (eg, physical examination, medical history) will be summarized using descriptive statistics and/or listed.

11.4. Immunogenicity Analyses

Descriptive statistics (actual values and changes from baseline, including 95% CIs, if applicable) will be calculated for continuous immunologic parameters by time point. Graphical representations of changes in immunologic parameters will be prepared, as applicable. Frequency tabulations will be calculated for discrete (qualitative) immunologic parameters by time point.

To assess the primary objective, only the Ad26.ZEBOV and MVA-BN-Filo vaccinated groups are considered. For each pairwise comparison of the ELISA actual values (in EU/mL) at 21 days post-boost of the intermediate dose level versus the release titer, estimated differences will be expressed as ratios of GMC with corresponding 95% CI. This 95% CI is determined from comparing $\log_{10}$-transformed ELISA concentrations between groups and back-transformation of the estimated difference and corresponding 95% CI. Non-inferiority of a dose level compared to the release titer will be shown if the 95% CI of the estimated GMC ratio ($GMC_{\text{intermediate (or low) dose level}}/GMC_{\text{release titer}}$) entirely lies above 2/3. Hierarchical testing will be applied: in case non-inferiority of the intermediate dose level versus the release titer is shown, non-inferiority of the low dose level versus the release titer will be tested in the same way.

The primary comparison will be repeated adjusted for sex, age and body weight as a sensitivity analysis.

As an exploratory analysis, response patterns over time for the immunologic parameters will be analyzed, taking into account within-subject correlations.

11.5. Safety Analyses

No formal statistical testing of safety data is planned. Adverse events and categorical safety parameters will be tabulated, and continuous safety parameters will be descriptively analyzed.

Baseline for all safety parameters will be defined as the last value before the prime vaccination.

Adverse Events (Including Reactogenicity)

The verbatim terms used in the CRF by investigators to report adverse events will be coded using the Medical Dictionary for Regulatory Activities (MedDRA). All reported adverse events (solicited local, solicited systemic, and unsolicited) will be included in the analysis. For each adverse event, the number and percentage of subjects who experience at least 1 occurrence of the given event will be summarized by group. Summaries, listings, datasets, or subject narratives may be provided, as appropriate, for those subjects who die, who discontinue study vaccine due to an adverse event, or who experience a severe or a serious adverse event.

The analysis for solicited adverse events will be done on those subjects in the Full Analysis set for whom reactogenicity assessments are available in the database. The analysis of unsolicited adverse events, including (but not limited to) serious adverse events, IREs, adverse events leading to discontinuation and fatal adverse events, will be done on the Full Analysis set.

Clinical Laboratory Tests (Pregnancy Tests)

The results of the serum and urine pregnancy tests will be listed.

Vital Signs

Descriptive statistics of blood pressure (systolic and diastolic), pulse/heart rate and body temperature values and changes from baseline will be summarized at each scheduled time point. The percentage of subjects with values beyond clinically important limits will be summarized.

Physical Examination

Physical examination findings will be listed.

11.6. Interim Analysis

Interim analyses may be performed during the study for the purpose of informing future vaccine-related decisions in a timely manner. The results will not influence the conduct of the study in terms of early termination or later safety or immunogenicity endpoint assessments. Study-site personnel (except for those with primary responsibility for study vaccine preparation and dispensing), the sponsor (except for programming, statistics, clinical and clinical immunology personnel involved in the analysis, and the sponsor committee involved in making future decisions for the program) and subjects will remain blinded to study vaccine allocation until the time of database lock for the primary analysis. Refer to Section 5 for further details on blinding.

A separate interim SAP will be prepared before the conduct of an interim analysis.

11.7. Data Review Committee

A DRC will be established by the sponsor before the start of the study and will convene to review the available safety data in case a pausing rule is met (as described Section 9.3.1). Ad hoc DRC meetings may be requested via the sponsor for any single event or combination of multiple events which are considered to jeopardize the safety of the subjects. After the review, the DRC will make recommendations regarding the continuation of the study. The details will be provided in a separate DRC charter.

12. ADVERSE EVENT REPORTING

Timely, accurate, and complete reporting and analysis of safety information from clinical studies are crucial for the protection of subjects, investigators, and the sponsor, and are mandated by regulatory agencies worldwide. The sponsor has established Standard Operating Procedures in conformity with regulatory requirements worldwide to ensure appropriate reporting of safety information; all clinical studies conducted by the sponsor or its affiliates will be conducted in accordance with those procedures.

12.1. Definitions

12.1.1. Adverse Event Definitions and Classifications

Adverse Event

An adverse event is any untoward medical occurrence in a clinical study subject administered a medicinal (investigational or non-investigational) product. An adverse event does not necessarily have a causal relationship with the treatment. An adverse event can therefore be any unfavorable and unintended sign (including an abnormal finding), symptom, or disease temporally associated with the use of a medicinal (investigational or non-investigational) product, whether or not related to that medicinal (investigational or non-investigational) product. (Definition per International Council for Harmonisation [ICH])

This includes any occurrence that is new in onset or aggravated in severity or frequency from the baseline condition, or abnormal results of diagnostic procedures, including laboratory test abnormalities.

Note: The sponsor collects adverse events starting with the signing of the ICF (refer to Section 12.3.1 for time of last adverse event recording).

Serious Adverse Event

A serious adverse event based on ICH and European Union Guidelines on Pharmacovigilance for Medicinal Products for Human Use is any untoward medical occurrence that at any dose:

- Results in death
- Is life-threatening
  (The subject was at risk of death at the time of the event. It does not refer to an event that hypothetically might have caused death if it were more severe.)
- Requires inpatient hospitalization or prolongation of existing hospitalization
- Results in persistent or significant disability/incapacity
- Is a congenital anomaly/birth defect
- Is a suspected transmission of any infectious agent via a medicinal product
- Is Medically Important*

*Medical and scientific judgment should be exercised in deciding whether expedited reporting is also appropriate in other situations, such as important medical events that may not be immediately life-threatening or result in death or hospitalization but may jeopardize the subject or may require intervention to prevent one of the other outcomes listed in the definition above. These should usually be considered serious.

If a serious and unexpected adverse event occurs for which there is evidence suggesting a causal relationship between the study vaccine and the event (eg, death from anaphylaxis), the event must be reported as a suspected unexpected serious adverse reaction (SUSAR) (even after the

study is over, if the sponsor, DRC or investigator becomes aware of them) by the sponsor to the Health Authorities and by the investigator to the IEC/IRB according to regulatory and local requirements.

Unlisted (Unexpected) Adverse Event/Reference Safety Information

An adverse event is considered unlisted if the nature or severity is not consistent with the applicable product reference safety information. For Ad26.ZEBOV and MVA-BN-Filo, the expectedness of an adverse event will be determined by whether or not it is listed in the Investigator's Brochures and Addenda, if applicable.

Adverse Event Associated With the Use of the Study Vaccine

An adverse event is considered associated with the use of the study vaccine if the attribution is **possibly, probably**, or **very likely** by the definitions listed in Section 12.1.2, Attribution Definitions.

Immediate Reportable Events

The following list of neuroinflammatory disorders are categorized as IREs, and should be reported to the sponsor within 24 hours of becoming aware of the event using the IRE Form. Relevant data from the IRE Form will be captured in the clinical database.

- Cranial nerve disorders, including paralyses/paresis (eg, Bell’s palsy)
- Optic neuritis
- Multiple sclerosis
- Transverse myelitis
- Guillain-Barré syndrome, including Miller Fisher syndrome, Bickerstaff’s encephalitis and other variants
- Acute disseminated encephalomyelitis, including site specific variants: eg, non-infectious encephalitis, encephalomyelitis, myelitis, myeloradiculomyelitis
- Myasthenia gravis and Lambert-Eaton myasthenic syndrome
- Immune-mediated peripheral neuropathies and plexopathies (including chronic inflammatory demyelinating polyneuropathy, multifocal motor neuropathy and polyneuropathies associated with monoclonal gammopathy)
- Narcolepsy
- Isolated paresthesia of >7 days duration

Symptoms, signs or conditions which might (or might not) represent the above diagnoses, should be recorded and reported as IREs even if the final or definitive diagnosis has not yet been determined, and alternative diagnoses have not yet been eliminated or shown to be less likely. Follow-up information and final diagnoses, if applicable, should be submitted as soon as they become available.

If the IRE is also serious (serious adverse event), it will also be reported using the same process as for other serious adverse events.

12.1.2. Attribution Definitions

Every effort should be made by the investigator to explain any adverse event and to assess its potential causal relationship, ie, to administration of the study vaccine or to alternative causes, eg, natural history of underlying disease(s), concomitant drug(s). This applies to all adverse events, whether serious or non-serious. Assessment of causality must be done by a licensed study physician (the investigator or designee).

The investigator will use the following guidelines to assess the causal relationship of an adverse event to study vaccine:

Not Related

An adverse event that is not related to the use of the vaccine.

Doubtful

An adverse event for which an alternative explanation is more likely, eg, concomitant drug(s), concomitant disease(s), or the relationship in time suggests that a causal relationship is unlikely.

Possible

An adverse event that might be due to the use of the vaccine. An alternative explanation, eg, concomitant drug(s), concomitant disease(s), is inconclusive. The relationship in time is reasonable; therefore, the causal relationship cannot be excluded.

Probable

An adverse event that might be due to the use of the vaccine. The relationship in time is suggestive. An alternative explanation is less likely, eg, concomitant drug(s), concomitant disease(s).

Very Likely

An adverse event that is listed as a possible adverse reaction and cannot be reasonably explained by an alternative explanation, eg, concomitant drug(s), concomitant disease(s). The relationship in time is very suggestive.

12.1.3. Severity Criteria

Adverse events will be coded for severity using the toxicity grading tables in Attachment 1. For adverse events not identified in the table, the following guidelines will apply:

**Mild**	Grade 1	Symptoms causing no or minimal interference with usual social and functional activities
**Moderate**	Grade 2	Symptoms causing greater than minimal interference with usual social and functional activities
**Severe**	Grade 3	Symptoms causing inability to perform usual social and functional activities

12.2. Special Reporting Situations

Safety events of interest on a sponsor study vaccine that may require expedited reporting or safety evaluation include, but are not limited to:

- Administration of an overdose of study vaccine
- Accidental or occupational exposure to a study vaccine
- Administration error involving a study vaccine (with or without subject/patient exposure to the study vaccine, eg, name confusion)
- IREs

Special reporting situations should be recorded in the CRF. For reporting of IREs, refer to Section 12.3.3. Any special reporting situation that meets the criteria of a serious adverse event should be recorded on the Serious Adverse Event page of the CRF.

12.3. Procedures

12.3.1. All Adverse Events

All adverse events and special reporting situations, whether serious or non-serious, will be reported from the time a signed and dated ICF is obtained until the 42-day post-boost visit. Serious adverse events and IREs will be collected from signing of the ICF onwards until the end of the study. Subjects will record symptoms of solicited local or systemic adverse events (reactogenicity) in the diary in the evening after each vaccination and then daily for the next 7 days. Serious adverse events must be reported by the investigator using the Serious Adverse Event Form. SUSARs will be reported even after the study is over, if the sponsor, the DRC or the investigator becomes aware of them. The sponsor will evaluate any safety information that is spontaneously reported by an investigator beyond the time frame specified in the protocol.

All adverse events, regardless of seriousness, severity, or presumed relationship to study vaccine, must be recorded using medical terminology in the source document and the CRF. Whenever possible, diagnoses should be given when signs and symptoms are due to a common etiology (eg, cough, runny nose, sneezing, sore throat, and head congestion should be reported as "upper respiratory infection"). Investigators must record in the CRF their opinion concerning the relationship of the adverse event to study vaccine. All measures required for adverse event management must be recorded in the source document and reported according to sponsor instructions. Solicited adverse events will be captured on a separate CRF page as described in the CRF Completion Guidelines. Unsolicited adverse events which will be reported on the Adverse Event page of the CRF. For reporting of IREs, refer to Section 12.3.3.

The sponsor assumes responsibility for appropriate reporting of adverse events to the regulatory authorities. The sponsor will also report to the investigator (and the head of the investigational institute where required) all SUSARs. The investigator (or sponsor where required) must report SUSARs to the appropriate IEC/IRB that approved the protocol unless otherwise required and documented by the IEC/IRB. A SUSAR will be reported to regulatory authorities unblinded.

Participating investigators and IEC/IRB will receive a blinded SUSAR summary, unless otherwise specified.

For all studies with an outpatient phase, including open-label studies, the subject must be provided with a "wallet (study) card" and instructed to carry this card with them for the duration of the study indicating the following:

- Study number
- Statement, in the local language(s), that the subject is participating in a clinical study
- Investigator's name and 24-hour contact telephone number
- Local sponsor's name and 24-hour contact telephone number (for medical staff only)
- Site number
- Subject number
- Any other information that is required to do an emergency breaking of the blind

12.3.2. Serious Adverse Events

All serious adverse events occurring during the study must be reported to the appropriate sponsor contact person by study-site personnel within 24 hours of their knowledge of the event.

Information regarding serious adverse events will be transmitted to the sponsor using the Serious Adverse Event Form, which must be completed and signed by a physician from the study site, and transmitted to the sponsor within 24 hours. The initial and follow-up reports of a serious adverse event should be made by facsimile (fax).

All serious adverse events that have not resolved by the end of the study, or that have not resolved upon discontinuation of the subject's participation in the study, must be followed until any of the following occurs:

- The event resolves
- The event stabilizes
- The event returns to baseline, if a baseline value/status is available
- The event can be attributed to agents other than the study vaccine or to factors unrelated to study conduct
- It becomes unlikely that any additional information can be obtained (subject or health care practitioner refusal to provide additional information, lost to follow-up after demonstration of due diligence with follow-up efforts)

Suspected transmission of an infectious agent by a medicinal product will be reported as a serious adverse event. Any event requiring hospitalization (or prolongation of hospitalization) that occurs during the course of a subject's participation in a study must be reported as a serious adverse event, except hospitalizations for the following:

- Hospitalizations not intended to treat an acute illness or adverse event (eg, social reasons such as pending placement in long-term care facility)
- Surgery or procedure planned before entry into the study (must be documented in the CRF). Note: Hospitalizations that were planned before the signing of the ICF, and where the underlying condition for which the hospitalization was planned has not worsened, will not be considered serious adverse events. Any adverse event that results in a prolongation of the originally planned hospitalization is to be reported as a new serious adverse event.

The cause of death of a subject in a study, whether or not the event is expected or associated with the study vaccine, is considered a serious adverse event.

12.3.3. Immediate Reportable Events

One subject in the study VAC52150EBL2001 experienced a serious and very rare condition called “Miller Fisher syndrome”. This condition consists of double vision, pain on moving the eye, and difficulty with balance while walking. Miller Fisher syndrome most commonly occurs following a recent infection. The subject experienced these symptoms about a week after suffering from a common cold and fever. The event happened about a month after boost vaccination with either MVA-BN-Filo or placebo. This subject had to go to the hospital for treatment and has recovered. After an extensive investigation, the event has been considered to be doubtfully related to vaccine and most likely related to the previous common cold.

Any events of neuroimmunologic significance (listed in Section 12.1.1) should be categorized as IREs and should be reported throughout the study using the IRE Form provided **within 24 hours to the sponsor**. Events suggestive of the disorders considered IREs should be reported even if the final diagnosis has not been yet determined, and follow-up information and final diagnosis should be submitted to the sponsor as soon as they become available.

If an event meets serious adverse event criteria (see above), it should be documented as such using the Serious Adverse Event Form, as well as the relevant CRF Adverse Event page and the complete IRE Form page 3 to be included as part of the Serious Adverse Event report.

12.3.4. Pregnancy

Pregnancies will be reported from signing of the ICF until the end of the study.

All initial reports of pregnancy in female subjects or partners of male subjects must be reported to the sponsor by the study-site personnel within 24 hours of their knowledge of the event using the appropriate pregnancy notification form. Abnormal pregnancy outcomes (eg, spontaneous abortion, fetal death, stillbirth, congenital anomalies, ectopic pregnancy) are considered serious adverse events and must be reported using the Serious Adverse Event Form. Any subject or partner of a male subject who becomes pregnant during the study must be promptly withdrawn from further study vaccination but should continue participation in the study for safety follow-up.

Because the effect of the study vaccine on sperm is unknown, pregnancies in partners of male subjects included in the study will be reported by the study-site personnel as noted above.

Follow-up information regarding the outcome of the pregnancy and any postnatal sequelae in the infant will be required.

The parent(s)/legal guardian of children born to vaccinated female subjects who become pregnant with estimated conception within 28 days after vaccination with MVA-BN-Filo or within 3 months after vaccination with Ad26.ZEBOV, will be approached to consent for enrollment of their children into the VAC52150 Vaccine Development Roll-over study, according to the same rules that apply for the other subjects (see Section 9.1.4).

12.4. Contacting Sponsor Regarding Safety

The names (and corresponding telephone numbers) of the individuals who should be contacted regarding safety issues or questions regarding the study are listed in the Contact Information page(s), which will be provided as a separate document.

13. PRODUCT QUALITY COMPLAINT HANDLING

A product quality complaint (PQC) is defined as any suspicion of a product defect related to manufacturing, labeling, or packaging, ie, any dissatisfaction relative to the identity, quality, durability, or reliability of a product, including its labeling or package integrity. A PQC may have an impact on the safety and efficacy of the product. Timely, accurate, and complete reporting and analysis of PQC information from studies are crucial for the protection of subjects, investigators, and the sponsor, and are mandated by regulatory agencies worldwide. The sponsor has established procedures in conformity with regulatory requirements worldwide to ensure appropriate reporting of PQC information; all studies conducted by the sponsor or its affiliates will be conducted in accordance with those procedures.

13.1. Procedures

All initial PQC reports must be reported to the sponsor by the study-site personnel within 24 hours after being made aware of the event.

If the defect is combined with a serious adverse event, the study-site personnel must report the PQC to the sponsor according to the serious adverse event reporting timelines (refer to Section 12.3.2). A sample of the suspected product should be maintained for further investigation if requested by the sponsor.

13.2. Contacting Sponsor Regarding Product Quality

The names (and corresponding telephone numbers) of the individuals who should be contacted regarding product quality issues are listed in the Contact Information page(s), which will be provided as a separate document.

14. STUDY VACCINE INFORMATION

14.1. Description of Study Vaccines

Ad26.ZEBOV

Ad26.ZEBOV is a monovalent recombinant, replication-incompetent Ad26-based vector that expresses the full length EBOV Mayinga GP and is produced in the human cell line PER.C6®.

The drug substance used in Ad26.ZEBOV drug product is manufactured, according to the 2x10 L scale process, in Bern, Switzerland manufacturing facility.

The Ad26.ZEBOV vaccine will be supplied at a concentration of 1×10^{11} vp/mL in 2-mL single use glass vials as a frozen liquid suspension to be thawed before use. Each vial contains an extractable volume of 0.5 mL. Refer to the Investigator's Brochure for a list of excipients.[14]

MVA-BN-Filo

MVA-BN-Filo is a recombinant multivalent vaccine intended for active immunization against Ebola and Marburg virus infection. MVA-BN-Filo is strongly attenuated; the vaccine is propagated in primary chicken embryo fibroblast cells and does not replicate in human cells.

The MVA-BN-Filo drug substance, starting from working virus seed (WVS) is manufactured in Kvistgård, Denmark manufacturing facility.

The MVA-BN-Filo vaccine will be supplied at a concentration of 2×10^{8} Inf.U/mL (nominal titer) in 2-mL glass vials as a frozen liquid suspension to be thawed before use. Each vial contains an extractable volume of 0.5 mL. Refer to the Investigator's Brochure for a list of excipients.[15]

Placebo

The placebo supplied for this study will be formulated as a sterile 0.9% saline for injection (as commercially available).

14.2. Packaging and Labeling

All study vaccines will be manufactured and packaged in accordance with Good Manufacturing Practice. All study vaccines will be packaged and labeled under the responsibility of the sponsor. No study vaccine can be repacked or relabeled on site without prior approval from the sponsor.

Further details for study vaccine packaging and labeling can be found in the Site Investigational Product Procedures Manual.

14.3. Preparation, Handling, and Storage

Study vaccine must be stored at controlled temperatures: Ad26.ZEBOV vials must be stored at ≤ -60°C and MVA-BN-Filo vials must be stored at ≤ -20°C.

Vials must be stored in a secured location with no access for unauthorized personnel. All study product storage equipment (including refrigerators, freezers) must be equipped with a continuous temperature monitor and alarm, and with back-up power systems. In the event that study vaccine is exposed to temperatures outside the specified temperature ranges, all relevant data will be sent to the sponsor to determine if the affected study vaccine can be used or will be replaced. The affected study vaccine must be quarantined and not used until further instruction from the sponsor is received.

Blinding will be achieved by preparation of study vaccine by unblinded qualified study-site personnel not involved in any other study-related procedure, and by the administration of vaccine in a masked syringe by a blinded study vaccine administrator (see Definitions of Terms).

Details on the preparation, the holding time and storage conditions from the time of preparation to administration of Ad26.ZEBOV and MVA-BN-Filo are provided in the Site Investigational Product Procedures Manual.

14.4. Study Vaccine Accountability

The investigator is responsible for ensuring that all study vaccine received at the site is inventoried and accounted for throughout the study. The study vaccine administered to the subject must be documented on the study vaccine accountability form. All study vaccine will be stored and disposed of according to the sponsor's instructions. Study-site personnel must not combine contents of the study vaccine containers.

Study vaccine must be handled in strict accordance with the protocol and the container label, and must be stored at the study site in a limited-access area or in a locked cabinet under appropriate environmental conditions. Unused study vaccine must be available for verification by the sponsor's study-site monitor during on-site monitoring visits. The return to the sponsor of unused study vaccine will be documented on the study vaccine return form. When the study site is an authorized destruction unit and study vaccine supplies are destroyed on-site, this must also be documented on the study vaccine return form.

Potentially hazardous materials such as used ampules, needles, syringes and vials containing hazardous liquids should be disposed of immediately in a safe manner and therefore will not be retained for study vaccine accountability purposes.

Study vaccine should be dispensed under the supervision of the investigator or a qualified member of the study-site personnel, or by a qualified staff member. Study vaccine will be supplied only to subjects participating in the study. Returned study vaccine must not be dispensed again, even to the same subject. Study vaccine may not be relabeled or reassigned for use by other subjects. The investigator agrees neither to dispense the study vaccine from, nor store it at, any site other than the study sites agreed upon with the sponsor.

15. STUDY-SPECIFIC MATERIALS

The investigator will be provided with the following supplies:

- Investigator's Brochures and Addenda (if applicable) for Ad26.ZEBOV and MVA-BN-Filo
- Site Investigational Product Procedures Manual
- Laboratory Manual
- IWRS Manual
- Electronic Data Capture (eDC) Manual/electronic CRF Completion Guidelines and Randomization Instructions
- Sample ICF
- Subject diaries
- Rulers, thermometers
- Subject wallet cards
- Recruitment tools, as applicable

16. ETHICAL ASPECTS

16.1. Study-specific Design Considerations

Potential subjects will be fully informed of the risks and requirements of the study and, during the study, subjects will be given any new information that may affect their decision to continue participation. They will be told that their consent to participate in the study is voluntary and may be withdrawn at any time with no reason given and without penalty or loss of benefits to which they would otherwise be entitled. Only subjects who are fully able to understand the risks, benefits, and potential adverse events of the study, and provide their consent voluntarily will be enrolled.

The total blood volume to be collected is considered to be within the limits of standard blood donation.

16.2. Regulatory Ethics Compliance

16.2.1. Investigator Responsibilities

The investigator is responsible for ensuring that the study is performed in accordance with the protocol, current ICH guidelines on Good Clinical Practice (GCP), and applicable regulatory and country-specific requirements.

Good Clinical Practice is an international ethical and scientific quality standard for designing, conducting, recording, and reporting studies that involve the participation of human subjects. Compliance with this standard provides public assurance that the rights, safety, and well-being of study subjects are protected, consistent with the principles that originated in the Declaration of Helsinki, and that the study data are credible.

16.2.2. Independent Ethics Committee or Institutional Review Board

Before the start of the study, the investigator (or sponsor where required) will provide the IEC/IRB with current and complete copies of the following documents (as required by local regulations):

- Final protocol and, if applicable, amendments
- Sponsor-approved ICF (and any other written materials to be provided to the subjects)
- Investigator's Brochure (or equivalent information) and amendments/addenda
- Sponsor-approved subject recruiting materials
- Information on compensation for study-related injuries or payment to subjects for participation in the study, if applicable
- Investigator's curriculum vitae or equivalent information (unless not required, as documented by the IEC/IRB)
- Information regarding funding, name of the sponsor, institutional affiliations, other potential conflicts of interest, and incentives for subjects
- Any other documents that the IEC/IRB requests to fulfill its obligation

This study will be undertaken only after the IEC/IRB has given full approval of the final protocol, amendments (if any, excluding the ones that are purely administrative, with no consequences for subjects, data or study conduct, unless required locally), the ICF, applicable recruiting materials, and subject compensation programs, and the sponsor has received a copy of this approval. This approval letter must be dated and must clearly identify the IEC/IRB and the documents being approved.

During the study the investigator (or sponsor where required) will send the following documents and updates to the IEC/IRB for their review and approval, where appropriate:

- Protocol amendments (excluding the ones that are purely administrative, with no consequences for subjects, data or study conduct)
- Revision(s) to ICF and any other written materials to be provided to subjects
- If applicable, new or revised subject recruiting materials approved by the sponsor
- Revisions to compensation for study-related injuries or payment to subjects for participation in the study, if applicable
- New edition(s) of the Investigator's Brochure and amendments/addenda
- Summaries of the status of the study at intervals stipulated in guidelines of the IEC/IRB (at least annually)
- Reports of adverse events that are serious, unlisted/unexpected, and associated with the study vaccine
- New information that may adversely affect the safety of the subjects or the conduct of the study

- Deviations from or changes to the protocol to eliminate immediate hazards to the subjects
- Report of deaths of subjects under the investigator's care
- Notification if a new investigator is responsible for the study at the site
- Development Safety Update Report and Line Listings, where applicable
- Any other requirements of the IEC/IRB

For all protocol amendments (excluding the ones that are purely administrative, with no consequences for subjects, data or study conduct), the amendment and applicable ICF revisions must be submitted promptly to the IEC/IRB for review and approval before implementation of the change(s).

At least once a year, the IEC/IRB will be asked to review and reapprove this study, where required.

At the end of the study, the investigator (or sponsor where required) will notify the IEC/IRB about the study completion (if applicable, the notification will be submitted through the head of investigational institution).

16.2.3. Informed Consent

Each subject must give written consent according to local requirements after the nature of the study has been fully explained. The ICF(s) must be signed before performance of any study-related activity. The ICF(s) that is/are used must be approved by both the sponsor and by the reviewing IEC/IRB and be in a language that the subject can read and understand. The informed consent should be in accordance with principles that originated in the Declaration of Helsinki, current ICH and GCP guidelines, applicable regulatory requirements, and sponsor policy.

Before enrollment in the study, the investigator or an authorized member of the study-site personnel must explain to potential subjects the aims, methods, reasonably anticipated benefits, and potential hazards of the study, and any discomfort participation in the study may entail. Subjects will be informed that their participation is voluntary and that they may withdraw consent to participate at any time. They will be informed that choosing not to participate will not affect the care the subject will receive. Finally, they will be told that the investigator will maintain a subject identification register for the purposes of long-term follow up if needed and that their records may be accessed by health authorities and authorized sponsor personnel without violating the confidentiality of the subject, to the extent permitted by the applicable law(s) or regulations. By signing the ICF the subject is authorizing such access, and agrees to allow his or her study physician to recontact the subject for the purpose of obtaining consent for additional safety evaluations, if needed.

The subject will be given sufficient time to read the ICF and the opportunity to ask questions. After this explanation and before entry into the study, consent should be appropriately recorded by means of the subject's personally dated signature. After having obtained the consent, a copy of the ICF must be given to the subject.

If the subject is unable to read or write, an impartial witness should be present for the entire informed consent process (which includes reading and explaining all written information) and should personally date and sign the ICF after the oral consent of the subject is obtained.

16.2.4. Privacy of Personal Data

The collection and processing of personal data from subjects enrolled in this study will be limited to those data that are necessary to fulfill the objectives of the study.

These data must be collected and processed with adequate precautions to ensure confidentiality and compliance with applicable data privacy protection laws and regulations. Appropriate technical and organizational measures to protect the personal data against unauthorized disclosures or access, accidental or unlawful destruction, or accidental loss or alteration must be put in place. Sponsor personnel whose responsibilities require access to personal data agree to keep the identity of subjects confidential.

The informed consent obtained from the subject includes explicit consent for the processing of personal data and for the investigator/institution to allow direct access to his or her original medical records (source data/documents) for study-related monitoring, audit, IEC/IRB review, and regulatory inspection. This consent also addresses the transfer of the data to other entities and to other countries.

The subject has the right to request through the investigator access to his or her personal data and the right to request rectification of any data that are not correct or complete. Reasonable steps will be taken to respond to such a request, taking into consideration the nature of the request, the conditions of the study, and the applicable laws and regulations.

Exploratory immunogenicity research is not conducted under standards appropriate for the return of data to subjects. In addition, the sponsor cannot make decisions as to the significance of any findings resulting from exploratory research. Therefore, exploratory research data will not be returned to subjects or investigators, unless required by law or local regulations. Privacy and confidentiality of data generated in the future on stored samples will be protected by the same standards applicable to all other clinical data.

16.2.5. Long-term Retention of Samples for Additional Future Research

Samples collected in this study for which consent has been obtained and for which additional material is available after study-specified testing is complete, may be stored for up to 15 years (or according to local regulations) for possible additional future scientific/genetic research. Samples will only be used to understand Ebola vaccine- and disease-related questions, and to develop tests/assays related to the characterization of EBOV-directed immune responses or diagnostic tests. The research may begin at any time during the study or the post-study storage period. Applicable approvals will be sought before any such samples are used for analysis not specified in the protocol (amendment) approved by the IEC/IRB.

Stored samples will be coded throughout the sample storage and analysis process and will not be labeled with personal identifiers. Subjects may withdraw their consent for their samples to be

stored for future research at any time (refer to Section 10.3). In such case, their blood samples will be destroyed after all the tests specified for this study have been concluded.

The sponsor will be responsible for the overall management of the sample inventory, shipping plan, allocation and storage of samples.

16.2.6. Country Selection

This study will only be conducted in those countries where the intent is to launch or otherwise help ensure access to the developed product if the need for the product persists, unless explicitly addressed as a specific ethical consideration in Section 16.1.

17. ADMINISTRATIVE REQUIREMENTS

17.1. Protocol Amendments

Neither the investigator nor the sponsor will modify this protocol without a formal amendment by the sponsor. All protocol amendments must be issued by the sponsor, and signed and dated by the investigator. Protocol amendments must not be implemented without prior IEC/IRB approval, or when the relevant competent authority has raised any grounds for non-acceptance, except when necessary to eliminate immediate hazards to the subjects, in which case the amendment must be promptly submitted to the IEC/IRB and relevant competent authority. Documentation of amendment approval by the investigator and IEC/IRB must be provided to the sponsor. When the change(s) involve(s) only logistic or administrative aspects of the study, the IRB/IEC (where required) only needs to be notified.

During the course of the study, in situations where a departure from the protocol is unavoidable, the investigator or other physician in attendance will contact the appropriate sponsor representative (listed in the Contact Information page(s), which will be provided as a separate document). Except in emergency situations, this contact should be made <u>before</u> implementing any departure from the protocol. In all cases, contact with the sponsor must be made as soon as possible to discuss the situation and agree on an appropriate course of action. The data recorded in the CRF and source documents will reflect any departure from the protocol, and the source documents will describe this departure and the circumstances requiring it.

17.2. Regulatory Documentation

17.2.1. Regulatory Approval/Notification

This protocol and any amendment(s) must be submitted to the appropriate regulatory authorities in each respective country, if applicable. A study may not be initiated until all local regulatory requirements are met.

17.2.2. Required Prestudy Documentation

The following documents must be provided to the sponsor before shipment of study vaccine to the study site:

- Protocol and amendment(s), if any, signed and dated by the principal investigator
- A copy of the dated and signed (or sealed, where appropriate per local regulations), written IEC/IRB approval of the protocol, amendments, ICF, any recruiting materials, and if applicable, subject compensation programs. This approval must clearly identify the specific protocol by title and number and must be signed (or sealed, where appropriate per local regulations) by the chairman or authorized designee.
- Name and address of the IEC/IRB, including a current list of the IEC/IRB members and their function, with a statement that it is organized and operates according to GCP and the applicable laws and regulations. If accompanied by a letter of explanation, or equivalent, from the IEC/IRB, a general statement may be substituted for this list. If an investigator or a member of the study-site personnel is a member of the IEC/IRB, documentation must be obtained to state that this person did not participate in the deliberations or in the vote/opinion of the study.
- Regulatory authority approval or notification, if applicable
- Signed and dated statement of investigator (eg, Form FDA 1572), if applicable
- Documentation of investigator qualifications (eg, curriculum vitae)
- Completed investigator financial disclosure form from the principal investigator, where required
- Signed and dated clinical trial agreement, which includes the financial agreement
- Any other documentation required by local regulations

The following documents must be provided to the sponsor before enrollment of the first subject:

- Completed investigator financial disclosure forms from all subinvestigators
- Documentation of subinvestigator qualifications (eg, curriculum vitae)
- Name and address of any local laboratory conducting tests for the study, and a dated copy of current laboratory normal ranges for these tests, if applicable
- Local laboratory documentation demonstrating competence and test reliability (eg, accreditation/license), if applicable

17.3. Subject Identification, Enrollment, and Screening Logs

The investigator agrees to complete a subject identification and enrollment log to permit easy identification of each subject during and after the study. This document will be reviewed by the sponsor study-site contact for completeness.

The subject identification and enrollment log will be treated as confidential and will be filed by the investigator in the study file. To ensure subject confidentiality, no copy will be made. All reports and communications relating to the study will identify subjects by subject identification

and date of birth. In cases where the subject is not randomized into the study, the date seen and date of birth will be used.

The investigator must also complete a subject screening log, which reports on all subjects who were seen to determine eligibility for inclusion in the study.

17.4. Source Documentation

At a minimum, source documents consistent in the type and level of detail with that commonly recorded at the study site as a basis for standard medical care, must be available for the following: subject identification, eligibility, and study identification; study discussion and date of signed informed consent; dates of visits; results of safety and immunogenicity parameters as required by the protocol; record of all adverse events and follow-up of adverse events; concomitant medication; study vaccine receipt/dispensing/return records; study vaccine administration information; and date of study completion and reason for early discontinuation of study vaccine or withdrawal from the study, if applicable.

The author of an entry in the source documents should be identifiable.

Specific details required as source data for the study and source data collection methods will be reviewed with the investigator before the study and will be described in the monitoring guidelines (or other equivalent document).

The subject diary will be considered a source document. Information from the diary provided to subjects to record symptoms of solicited local and systemic adverse events until 7 days after each vaccination will be reviewed by the investigator to transcribe into the relevant parts of the CRF as described in the CRF Completion Guidelines.

17.5. Case Report Form Completion

Case report forms are prepared and provided by the sponsor for each subject in electronic format. All CRF entries, corrections, and alterations must be made by the investigator or authorized study-site personnel. The investigator must verify that all data entries in the CRF are accurate and correct.

The study data will be transcribed by study-site personnel from the source documents onto an electronic CRF, if applicable. Study-specific data will be transmitted in a secure manner to the sponsor.

Data must be entered into CRF in English. The CRF must be completed as soon as possible after a subject visit and the forms should be available for review at the next scheduled monitoring visit.

If necessary, queries will be generated in the electronic Data Capture (eDC) tool. If corrections to a CRF are needed after the initial entry into the CRF, this can be done in either of the following ways:

- Investigator and study-site personnel can make corrections in the eDC tool at their own initiative or as a response to an auto query (generated by the eDC tool).
- Sponsor or sponsor delegate can generate a query for resolution by the investigator and study-site personnel.

17.6. Data Quality Assurance/Quality Control

Steps to be taken to ensure the accuracy and reliability of data include the selection of qualified investigators and appropriate study sites, review of protocol procedures with the investigator and study-site personnel before the study, periodic monitoring visits by the sponsor and direct transmission of clinical laboratory data from a central laboratory into the sponsor's database. Written instructions will be provided for collection, handling, storage, and shipment of samples.

Guidelines for CRF completion will be provided and reviewed with study-site personnel before the start of the study. The sponsor will review CRF for accuracy and completeness during on-site monitoring visits and after transmission to the sponsor; any discrepancies will be resolved with the investigator or designee, as appropriate. After upload of the data into the study database they will be verified for accuracy and consistency with the data sources.

17.7. Record Retention

In compliance with the ICH/GCP guidelines, the investigator/institution will maintain all CRF and all source documents that support the data collected from each subject, as well as all study documents as specified in ICH/GCP Section 8, Essential Documents for the Conduct of a Clinical Trial, and all study documents as specified by the applicable regulatory requirement(s). The investigator/institution will take measures to prevent accidental or premature destruction of these documents.

Essential documents must be retained until at least 2 years after the last approval of a marketing application in an ICH region and until there are no pending or contemplated marketing applications in an ICH region or until at least 2 years have elapsed since the formal discontinuation of clinical development of the investigational product. These documents will be retained for a longer period if required by the applicable regulatory requirements or by an agreement with the sponsor. It is the responsibility of the sponsor to inform the investigator/institution as to when these documents no longer need to be retained.

If the responsible investigator retires, relocates, or for other reasons withdraws from the responsibility of keeping the study records, custody must be transferred to a person who will accept the responsibility. The sponsor must be notified in writing of the name and address of the new custodian. Under no circumstance shall the investigator relocate or dispose of any study documents before having obtained written approval from the sponsor.

If it becomes necessary for the sponsor or the appropriate regulatory authority to review any documentation relating to this study, the investigator/institution must permit access to such reports.

17.8. Monitoring

The sponsor will perform on-site monitoring visits as frequently as necessary. The monitor will record dates of the visits in a study site visit log that will be kept at the study site. The first post-initiation visit will be made as soon as possible after enrollment has begun. At these visits, the monitor will compare the data entered into the CRF with the source documents (eg, hospital/clinic/physician's office medical records). The nature and location of all source documents will be identified to ensure that all sources of original data required to complete the CRF are known to the sponsor and study-site personnel and are accessible for verification by the sponsor study-site contact. If electronic records are maintained at the study site, the method of verification must be discussed with the study-site personnel.

Direct access to source documents (medical records) must be allowed for the purpose of verifying that the recorded data are consistent with the original source data. Findings from this review will be discussed with the study-site personnel. The sponsor expects that, during monitoring visits, the relevant study-site personnel will be available, the source documents will be accessible, and a suitable environment will be provided for review of study-related documents. The monitor will meet with the investigator on a regular basis during the study to provide feedback on the study conduct.

There will be independent monitoring of the pharmacy and preparation of study vaccines by an unblinded monitor (independent study vaccine monitor, see Definitions of Terms); regular monitors will be blinded.

In addition to on-site monitoring visits, remote contacts can occur. It is expected that during these remote contacts, study-site personnel will be available to provide an update on the progress of the study at the site.

17.9. Study Completion/Termination

17.9.1. Study Completion/End of Study

The study is considered completed with the last visit for the last subject participating in the study. The final data from the study site will be sent to the sponsor (or designee) after completion of the final subject visit at that study site, in the time frame specified in the Clinical Trial Agreement.

17.9.2. Study Termination

The sponsor reserves the right to close the study site or terminate the study at any time for any reason at the sole discretion of the sponsor. Study sites will be closed upon study completion. A study site is considered closed when all required documents and study supplies have been collected and a study-site closure visit has been performed.

The investigator may initiate study-site closure at any time, provided there is reasonable cause and sufficient notice is given in advance of the intended termination.

Reasons for the early closure of a study site by the sponsor or investigator may include but are not limited to:

- Failure of the investigator to comply with the protocol, the requirements of the IEC/IRB or local health authorities, the sponsor's procedures, or GCP guidelines
- Inadequate recruitment of subjects by the investigator
- Discontinuation of further study vaccine development

17.10. On-site Audits

Representatives of the sponsor's clinical quality assurance department may visit the study site at any time during or after completion of the study to conduct an audit of the study in compliance with regulatory guidelines and company policy. These audits will require access to all study records, including source documents, for inspection. Subject privacy must, however, be respected. The investigator and study-site personnel are responsible for being present and available for consultation during routinely scheduled study-site audit visits conducted by the sponsor or its designees.

Similar auditing procedures may also be conducted by agents of any regulatory body, either as part of a national GCP compliance program or to review the results of this study in support of a regulatory submission. The investigator should immediately notify the sponsor if he/she has been contacted by a regulatory agency concerning an upcoming inspection.

17.11. Use of Information and Publication

All information, including but not limited to information regarding Ad26.ZEBOV and MVA-BN-Filo or the sponsor's operations (eg, patent application, formulas, manufacturing processes, basic scientific data, prior clinical data, formulation information) supplied by the sponsor to the investigator and not previously published, and any data, including exploratory research data, generated as a result of this study, are considered confidential and remain the sole property of the sponsor. The investigator agrees to maintain this information in confidence and use this information only to accomplish this study, and will not use it for other purposes without the sponsor's prior written consent.

The investigator understands that the information developed in the study will be used by the sponsor in connection with the continued development of Ad26.ZEBOV and MVA-BN-Filo, and thus may be disclosed as required to other clinical investigators or regulatory agencies. To permit the information derived from the clinical studies to be used, the investigator is obligated to provide the sponsor with all data obtained in the study.

The results of the study will be reported in a Clinical Study Report generated by the sponsor and will contain data from all study sites that participated in the study as per protocol. Recruitment performance or specific expertise related to the nature and the key assessment parameters of the study will be used to determine a coordinating investigator. Results of exploratory analyses performed after the Clinical Study Report has been issued will be reported in a separate report and will not require a revision of the Clinical Study Report. Study subject identifiers will not be

used in publication of results. Any work created in connection with performance of the study and contained in the data that can benefit from copyright protection (except any publication by the investigator as provided for below) shall be the property of the sponsor as author and owner of copyright in such work.

Consistent with Good Publication Practices and International Committee of Medical Journal Editors guidelines, the sponsor shall have the right to publish such primary (multicenter) data and information without approval from the investigator. The investigator has the right to publish study site-specific data after the primary data are published. If an investigator wishes to publish information from the study, a copy of the manuscript must be provided to the sponsor for review at least 60 days before submission for publication or presentation. Expedited reviews will be arranged for abstracts, poster presentations, or other materials. If requested by the sponsor in writing, the investigator will withhold such publication for up to an additional 60 days to allow for filing of a patent application. In the event that issues arise regarding scientific integrity or regulatory compliance, the sponsor will review these issues with the investigator. The sponsor will not mandate modifications to scientific content and does not have the right to suppress information. For multicenter study designs and substudy approaches, secondary results generally should not be published before the primary endpoints of a study have been published. Similarly, investigators will recognize the integrity of a multicenter study by not submitting for publication data derived from the individual study site until the combined results from the completed study have been submitted for publication, within 12 months of the availability of the final data (tables, listings, graphs), or the sponsor confirms there will be no multicenter study publication. Authorship of publications resulting from this study will be based on the guidelines on authorship, such as those described in the Uniform Requirements for Manuscripts Submitted to Biomedical Journals, which state that the named authors must have made a significant contribution to the design of the study or analysis and interpretation of the data, provided critical review of the paper, and given final approval of the final version.

Registration of Clinical Studies and Disclosure of Results

The sponsor will register and disclose the existence of and the results of clinical studies as required by law.

REFERENCES

1. Baden LR, Liu J, Li H, et al. Induction of HIV-1-specific mucosal immune responses following intramuscular recombinant adenovirus serotype 26 HIV-1 vaccination of humans. J Infect Dis. 2015;211(4):518-528.
2. Baden LR, Walsh SR, Seaman MS, et al. First-in-human evaluation of the safety and immunogenicity of a recombinant adenovirus serotype 26 HIV-1 Env vaccine (IPCAVD 001). J Infect Dis. 2013;207:240-247.
3. Baize S, Pannetier D, Oestereich L, et al. Emergence of Zaire Ebola virus disease in Guinea. N Engl J Med. 2014;371(15):1418-1425.
4. Barouch DH, Liu J, Peter L, et al. Characterization of humoral and cellular immune responses elicited by a recombinant adenovirus serotype 26 HIV-1 Env vaccine in healthy adults (IPCAVD 001). J Infect Dis. 2013;207:248-256.
5. Clinical Study Report MAL-V-A001. A Phase I/IIa, double-blind, randomized, placebo-controlled, dose-escalation clinical study evaluating safety, tolerability and immunogenicity of two dose levels of recombinant adenoviral serotype Ad35 and serotype Ad26 vectors expressing the malaria *Plasmodium falciparum* circumsporozoite antigen administered as heterologous prime-boost regimen, and assessing protective efficacy of the higher dose in a malaria challenge model in unblinded conditions. Crucell Holland B.V. (Aug 2014).
6. Clinical Study Report POX-MVA-013. A randomized, double-blind, placebo-controlled Phase III trial to evaluate immunogenicity and safety of three consecutive production lots of IMVAMUNE® (MVA-BN®) smallpox vaccine in healthy, Vaccinia-naïve subjects. Bavarian Nordic (Aug 2015).
7. Elizaga ML, Vasan S, Marovich MA, et al.; MVA Cardiac Safety Working Group. Prospective surveillance for cardiac adverse events in healthy adults receiving modified vaccinia Ankara vaccines: a systematic review. PLoS One. 2013;8(1):e54407.
8. Frey SE, Newman FK, Kennedy JS, et al. Clinical and immunologic responses to multiple doses of IMVAMUNE (Modified Vaccinia Ankara) followed by Dryvax challenge. Vaccine. 2007;25(51):8562-8573.
9. Friedrich B, Trefry J, Biggins J, et al. Potential vaccines and post-exposure treatments for filovirus infections. Viruses. 2012;4(9):1619-1650.
10. IMVANEX suspension for injection. UK Summary of Product Characteristics. Available at: http://www.ema.europa.eu/docs/en_GB/document_library/EPAR_-_Product_Information/human/002596/WC500147896.pdf. Accessed 26 March 2015.
11. Investigator Brochure: Ad26.ENVA.01 HIV-1 Vaccine. Edition 3. Crucell Holland B.V. (Jun 2013).
12. Investigator Brochure: JNJ-55471468-AAA, JNJ-55471494-AAA, JNJ-55471520-AAA (Ad26.Mos.HIV) HIV 1 vaccine. Edition 2. Crucell Holland B.V. (Nov 2014).
13. Investigator Brochure: JNJ-55471468-AAA, JNJ-55471494-AAA, JNJ-55471520-AAA (Ad26.Mos.HIV) HIV 1 vaccine. Addendum to Edition 2. Crucell Holland B.V. (Dec 2014).
14. Investigator's Brochure: JNJ-61210474 (Ad26.ZEBOV) Edition 2.1. Crucell Holland B.V. (5 June 2015). For updated information, refer to Edition 4 (23 June 2016).
15. Investigator's Brochure: MVA-BN-Filo (MVA-mBN226B) Edition 5. Bavarian Nordic A/S (18 March 2015). For updated information, refer to Edition 10 (11 July 2016).
16. Karita E, Mutua G, Bekker LG, et al. Safety in a Phase 1 randomized, double-blind, placebo-controlled trial evaluating two adenovirus hiv vaccines in three different geographic regions (IAVI-B003/IPCAVD-004/HVTN091 trial). Poster presented at AIDS Vaccine, 2013, Barcelona, Spain.
17. MVA-BN® (Modified Vaccinia Ankara - Bavarian Nordic). Available at: http://id.bavarian-nordic.com/pipeline/technology-platform/mva-bn.aspx Accessed 26 March 2015.

18. Neff J, Modlin J, Birkhead GS, et al. Monitoring the safety of a smallpox vaccination program in the United States: report of the joint Smallpox Vaccine Safety Working Group of the advisory committee on immunization practices and the Armed Forces Epidemiological Board. Clin Infect Dis. 2008;46 Suppl.3:S258-S270.

19. Sarwar UN, Costner P, Enama ME, et al. Safety and immunogenicity of DNA vaccines encoding ebolavirus and marburgvirus wild-type glycoproteins in a phase I clinical trial. J Infect Dis. 2015;211(4):549-557.

20. Stittelaar KJ, Kuiken T, de Swart RL et al. Safety of Modified Vaccinia Virus Ankara (MVA) in immune-suppressed macaques. Vaccine. 2001;19(27):3700-3709.

21. Verheust C, Goossens M, Pauwels K, Breyer D. Biosafety aspects of Modified Vaccinia Virus Ankara (MVA)-based vectors used for gene therapy or vaccination. Vaccine. 2012;30(16):2623-2632.

22. Vollmar J, Arndtz N, Eckl KM, et al. Safety and immunogenicity of IMVAMUNE, a promising candidate as a third generation smallpox vaccine. Vaccine. 2006;24(12):2065-2070.

23. WHO Fact Sheet N°103 Ebola Virus Disease 2014. Available at: http://www.who.int/mediacentre/factsheets/fs103/en/. Accessed 26 March 2015.

ATTACHMENTS

Attachment 1: Toxicity Tables for Use in Trials Enrolling Healthy Adults

The abbreviations used in the following tables are: ALT: alanine aminotransferase; aPTT: activated partial thromboplastin time; AST: aspartate aminotransferase; AV block: atrioventricular block; bpm: beats per minute; CK: creatine kinase; FEV_1: forced expiratory volume in 1 second; g: gram; HI: high; HPF: high power field; INR: international normalized ratio; IV: intravenous; LO: low; mEq: milliequivalent; mm Hg: millimeter of mercury; ms: millisecond; N: not graded; PT: prothrombin time; PTT: partial thromboplastin time; QTc: QT-interval corrected for heart rate; QTcB: Bazett's corrected QT interval; QTcF: Fridericia's corrected QT interval; RBC: red blood cell; Rx: therapy; s: second; U: unit; ULN: upper limit of normal

CLINICAL ADVERSE EVENTS

Grading scale used for clinical adverse events is adapted from the DMID Toxicity Tables (2014). For adverse events not included in the tables below, refer to the severity criteria guidelines in Section 12.1.3.

Cardiovascular	Grade 1	Grade 2	Grade 3
Arrhythmia		Asymptomatic, transient signs, no Rx required	Recurrent/persistent; symptomatic Rx required
Hemorrhage, blood loss	Estimated blood loss ≤100 mL	Estimated blood loss >100 mL, no transfusion required	Transfusion required
QTcF (Fridericia's correction) [a] or QTcB (Bazett's correction)	Asymptomatic, QTc interval 450-479 ms, *OR*<br>Increase in interval<30 ms above baseline	Asymptomatic, QTc interval 480-499 ms, *OR*<br>Increase in interval 30-60 ms above baseline[b]	Asymptomatic, QTc interval ≥500 ms, *OR*<br>Increase in interval ≥60 ms above baseline
PR interval (prolonged)	PR interval 0.21-0.25 s	PR interval >0.25 s	Type II 2nd degree AV block *OR* Ventricular pause >3.0 s
**Respiratory**	**Grade 1**	**Grade 2**	**Grade 3**
Cough	Transient-no treatment	Persistent cough	Interferes with daily activities
Bronchospasm, acute	Transient; no treatment; FEV_1 71%-80% of peak flow	Requires treatment; normalizes with bronchodilator; FEV_1 60%-70% (of peak flow)	No normalization with bronchodilator; FEV_1 <60% of peak flow
Dyspnea	Does not interfere with usual and social	Interferes with usual and social activities,	Prevents daily and usual social activity

[a] Inclusion dependent upon protocol requirements.

[b] The Grade 2 increase in interval is changed from 30-50 ms to 30-60 ms since the original DMID Toxicity Tables (2014) did not cover the increase in interval between 50 and 60 ms.

	activities	no treatment	or requires treatment
**Gastrointestinal**	**Grade 1**	**Grade 2**	**Grade 3**
Nausea/vomiting	Minimal symptoms; caused minimal or no interference with work, school or self-care activities	Notable symptoms; required modification in activity or use of medications; did not result in loss of work or cancellation of social activities	Incapacitating symptoms; required bed rest and/or resulted in loss of work or cancellation of social activities
Diarrhea	2-3 loose or watery stools or <400 g/24 hours	4-5 loose or watery stools or 400-800 g/24 hours	6 or more loose or watery stools or >800 g/24 hours or requires IV hydration
**Reactogenicity**	**Grade 1**	**Grade 2**	**Grade 3**
Local reactions			
Pain/tenderness at injection site	Aware of symptoms but easily tolerated; does not interfere with activity; discomfort only to touch	Notable symptoms; required modification in activity or use of medications; discomfort with movement	Incapacitating symptoms; inability to do work or usual activities; significant discomfort at rest
Erythema/redness[a]	2.5-5 cm	5.1-10 cm	>10 cm
Induration/swelling[b]	2.5-5 cm and does not interfere with activity	5.1-10 cm or interferes with activity	>10 cm or prevents daily activity
Itching at the injection site	Minimal symptoms; caused minimal or no interference with work, school or self-care activities	Notable symptoms; required modification in activity or use of medications; did not result in loss of work or cancellation of social activities	Incapacitating symptoms; required bed rest and/or resulted in loss of work or cancellation of social activities
Systemic reactions			
Allergic reaction	Pruritus without rash	Localized urticaria	Generalized urticaria; angioedema or anaphylaxis
Headache	Minimal symptoms; caused minimal or no interference with work, school or self-care activities	Notable symptoms; required modification in activity or use of medications; did not result in loss of work or cancellation of social activities	Incapacitating symptoms; required bed rest and/or resulted in loss of work or cancellation of social activities
Fatigue/malaise	Minimal symptoms; caused minimal or no interference with work, school or self-care activities	Notable symptoms; required modification in activity or use of medications; did not result in loss of work or cancellation of social activities	Incapacitating symptoms; required bed rest and/or resulted in loss of work or cancellation of social activities

[a] In addition to grading the measured local reaction at the greatest single diameter, the measurement should be recorded as a continuous variable.
[b] Induration/swelling should be evaluated and graded using the functional scale as well as the actual measurement.

Myalgia	Minimal symptoms; caused minimal or no interference with work, school or self-care activities	Notable symptoms; required modification in activity or use of medications; did not result in loss of work or cancellation of social activities	Incapacitating symptoms; required bed rest and/or resulted in loss of work or cancellation of social activities
Arthralgia	Minimal symptoms; caused minimal or no interference with work, school or self-care activities	Notable symptoms; required modification in activity or use of medications; did not result in loss of work or cancellation of social activities	Incapacitating symptoms; required bed rest and/or resulted in loss of work or cancellation of social activities
Chills	Minimal symptoms; caused minimal or no interference with work, school or self-care activities	Notable symptoms; required modification in activity or use of medications; did not result in loss of work or cancellation of social activities	Incapacitating symptoms; required bed rest and/or resulted in loss of work or cancellation of social activities

LABORATORY TOXICITY GRADING

Grading scale used for lab assessments is based on 'FDA's Toxicity Grading Scale for Healthy Adult and Adolescent Volunteers Enrolled in Preventive Vaccine Clinical Trials', but grade 3 and 4 are pooled below, consistent with the 3 scale toxicity grading used throughout the protocol. **If a laboratory value falls within the grading as specified below but also within the laboratory normal limits, the value is considered as normal.** For hemoglobin only the change from reference is used for the grading. The FDA table does not include toxicity grading for hematocrit, red blood cell counts or INR.

Blood, Serum, or Plasma Chemistries [a,b]	LO/HI/N [c]	Mild (Grade 1)	Moderate (Grade 2)	Severe (Grade 3)
Sodium (mEq/L or mmol/L)	LO	132-134	130-131	≤129
	HI	144-145	146-147	≥148
Potassium (mEq/L or mmol/L)	LO	3.5-3.6	3.3-3.4	≤3.2
	HI	5.1-5.2	5.3-5.4	≥5.5
Glucose (mg/dL)	LO	65-69	55-64	≤54
	HI [d]	100-110	111-125	>125
	HI [e]	110-125	126-200	>200
Blood urea nitrogen	HI	23-26 (mg/dL) or 8.3-9.4 (mmol/L)	27-31 (mg/dL) or 9.5- 11.2 (mmol/L)	>31 (mg/dL) or >11.2 (mmol/L)
Creatinine	N	1.5-1.7 (mg/dL) or 133-151 (μmol/L)	1.8-2.0 (mg/dL) or 152-177 (μmol/L)	>2.0 (mg/dL) or >177 (μmol/L)
Calcium (mg/dL)	LO	8.0-8.4	7.5-7.9	<7.5
	HI	10.5-11.0	11.1-11.5	>11.5
Magnesium (mg/dL)	LO	1.3-1.5	1.1-1.2	<1.1
Phosphorus (mg/dL)	LO	2.3-2.5	2.0-2.2	<2.0
Creatine kinase (CK) (mg/dL)	N	1.25-1.5xULN	1.6-3.0xULN	≥3.1xULN
Albumin (g/dL)	LO	2.8-3.1	2.5-2.7	<2.5
Total protein (g/dL)	LO	5.5-6.0	5.0-5.4	<5.0
Alkaline phosphatase (U/L)	N	1.1-2xULN	2.1-3xULN	>3xULN
AST (U/L)	HI	1.1-2.5xULN	2.6-5xULN	>5xULN
ALT (U/L)	HI	1.1-2.5xULN	2.6-5xULN	>5xULN
Bilirubin, serum total (mg/dL) – when accompanied by any increase in Liver Function Test		1.1-1.25xULN	1.26-1.5xULN	>1.5xULN
Bilirubin, serum total (mg/dL) – when Liver Function Test is normal		1.1-1.5xULN	1.6-2.0xULN	>2.0xULN
Amylase (U/L)	N	1.1x1.5ULN	1.6-2.0xULN	>2.0xULN
Lipase (U/L)	N	1.1x1.5ULN	1.6-2.0xULN	>2.0xULN

[a] Depending upon the laboratory used, references ranges, eligibility ranges and grading may be split out by sex and/or age.
[b] Cardiac troponin I increase by factor: >ULN-<2.0xULN; ≥2.0-<5.0xULN; ≥5.0xULN. (This footnote is added by the sponsor).
[c] Low, High, Not Graded.
[d] Fasting.
[e] Non-fasting.

**Hematology**	**LO/HI/N** [a]	**Mild (Grade 1)**	**Moderate (Grade 2)**	**Severe (Grade 3)**
Hemoglobin (women) change from baseline (g/dL)	LO	Any decrease-1.5	1.6-2.0	>2.0
Hemoglobin (men) change from baseline (g/dL)	LO	Any decrease-1.5	1.6-2.0	>2.0
White blood cell count (cell/mm^3)	HI	10,800-15,000	15,001-20,000	>20,000
	LO	2,500-3,500	1,500-2,499	<1,500
Lymphocytes (cell/mm^3)	LO	750-1,000	500-749	< 500
Neutrophils (cell/mm^3)	LO	1,500-2,000	1,000-1,499	< 1000
Eosinophils (cell/mm^3)	HI	650-1500	1501-5000	> 5000
Platelets (cell/mm^3)	LO	125,000-140,000	100,000-124,000	<100,000
**Coagulation**				
Prothrombin time (PT, seconds)	HI	1.0-1.10xULN	1.11-1.20xULN	>1.20xULN
International normalized ratio (INR)[b]	HI	1.1-1.5xULN	1.6-2.0xULN	>2.0xULN
Partial thromboplastin time (PTT or aPTT, seconds)	HI	1.0-1.2xULN	1.21-1.4xULN	>1.4xULN
Fibrinogen (mg/dL)	HI	400-500	501-600	>600
	LO	150-200	125-149	<125
**Urine**				
Protein (dipstick)	HI	Trace	1+	2+
Glucose (dipstick)	HI	Trace	1+	2+
Blood (microscopic) - red blood cells per high power field (RBC/HPF)	HI	1-10	11-50	>50 and/or gross blood

[a] Low, High, Not Graded.
[b] For INR, the values in the table are based on the Division of AIDS Table for Grading the Severity of Adult and Pediatric Adverse Events, 2009.

VITAL SIGNS TOXICITY GRADING

Grading scale used for vital signs is according to DMID Toxicity Tables (2014)

Vital Signs	LO/HI/N [a]	Mild (Grade 1) [b]	Moderate (Grade 2)	Severe (Grade 3)
Fever (°C)[c]	HI	38.0-38.4	38.5-38.9	>38.9
Fever (°F)	HI	100.4-101.1	101.2-102.0	>102.0
Tachycardia - beats per minute	HI	101-115	116-130	>130 or ventricular dysrhythmias
Bradycardia - beats per minute	LO	50-54 or 45-50 bpm if baseline <60 bpm	45-49 or 40-44 bpm if baseline <60 bpm	<45 or <40 bpm if baseline <60 bpm
Hypertension (systolic) - mmHg[d]	HI	141-150	151-160	>160
Hypertension (diastolic) - mmHg	HI	91-95	96-100	>100
Hypotension (systolic) - mmHg	LO	85-89	80-84	<80
Tachypnea - breaths per minute	HI	23-25	26-30	>30

[a] Low, High, Not Graded.
[b] If initial bound of grade 1 has gap from reference range or eligibility range, calculations based on the New England Journal of Medicine (NEJM) reference ranges.
[c] Oral temperature; no recent hot or cold beverages or smoking. A protocol should select either °C or °F for inclusion.
[d] Assuming subject is awake, resting, and supine; for adverse events, 3 measurements on the same arm with concordant results.

INVESTIGATOR AGREEMENT

I have read this protocol and agree that it contains all necessary details for carrying out this study. I will conduct the study as outlined herein and will complete the study within the time designated.

I will provide copies of the protocol and all pertinent information to all individuals responsible to me who assist in the conduct of this study. I will discuss this material with them to ensure that they are fully informed regarding the study drug, the conduct of the study, and the obligations of confidentiality.

Coordinating Investigator (where required):

Name (typed or printed): ______________________

Institution and Address: ______________________

____

____

____

Signature: ______________________ Date: ______________________

(Day Month Year)

Principal (Site) Investigator:

Name (typed or printed): ______________________

Institution and Address: ______________________

____

____

____

Telephone Number: ______________________

Signature: ______________________ Date: ______________________

(Day Month Year)

Sponsor's Responsible Medical Officer:

Name (typed or printed): [redacted]

Institution: [redacted]

Signature: [electronic signature appended at the end of the protocol] Date: ______________________

(Day Month Year)

Note: If the address or telephone number of the investigator changes during the course of the study, written notification will be provided by the investigator to the sponsor, and a protocol amendment will not be required.

LAST PAGE

SIGNATURES

Signed by	Date	Justification
[redacted]	01Sep2016, 09:59:05 AM, UTC	Document Approval

Crucell Holland B.V.*

Clinical Protocol

A Phase 3, Randomized, Double-Blind, Placebo-Controlled Study to Evaluate the Immunogenicity, Safety and Tolerability of a Heterologous Prime-Boost Regimen Using Three Different Batches of Ad26.ZEBOV and a Single Batch of MVA-BN®-Filo in Healthy Adult Subjects

Protocol VAC52150EBL3003; Phase 3
AMENDMENT 1

IND Number: 16280

VAC52150 (Ad26.ZEBOV/MVA-BN-Filo [MVA-mBN226B])

*Crucell Holland B.V. is a Janssen pharmaceutical company of Johnson & Johnson and is hereafter referred to as the sponsor of the study. The sponsor is identified on the Contact Information page that accompanies the protocol.

Status: Approved
Date: 18 November 2015
EDMS number: EDMS-ERI-106422179, 5.0

GCP Compliance: This study will be conducted in compliance with Good Clinical Practice, and applicable regulatory requirements.

Confidentiality Statement
The information in this document contains trade secrets and commercial information that are privileged or confidential and may not be disclosed unless such disclosure is required by applicable law or regulations. In any event, persons to whom the information is disclosed must be informed that the information is privileged or confidential and may not be further disclosed by them. These restrictions on disclosure will apply equally to all future information supplied to you that is indicated as privileged or confidential.

**Protocol History<br>VAC52150EBL3003_Protocol**		
Document Type and<br>*File Name*	Issued Date	Comments
Initial Clinical Protocol<br>*VAC52150EBL3003_Protocol*	30 July 2015	-
Protocol Amendment 1<br>*VAC52150EBL3003_Protocol_Amend_1*	This document	For details, see Section Amendment_1

TABLE OF CONTENTS

TABLE OF CONTENTS 3

LIST OF ATTACHMENTS 5

LIST OF IN-TEXT TABLES 5

PROTOCOL AMENDMENT 6

SYNOPSIS 9

TIME AND EVENTS SCHEDULE 14

ABBREVIATIONS 16

DEFINITIONS OF TERMS 17

1. INTRODUCTION 18
1.1. Background 18
1.2. Benefit/Risk Section 23
1.2.1. Known Benefits 23
1.2.2. Potential Benefits 23
1.2.3. Known Risks 23
1.2.4. Potential Risks 23
1.2.5. Overall Benefit/Risk Assessment 25
1.3. Overall Rationale for the Study 26

2. OBJECTIVES AND HYPOTHESIS 27
2.1. Objectives 27
2.2. Hypothesis 28

3. STUDY DESIGN AND RATIONALE 28
3.1. Overview of Study Design 28
3.2. Study Design Rationale 31

4. SUBJECT POPULATION 32
4.1. Inclusion Criteria 32
4.2. Exclusion Criteria 34
4.3. Prohibitions and Restrictions 36

5. STUDY VACCINE ALLOCATION AND BLINDING 37

6. DOSAGE AND ADMINISTRATION 38
6.1. General Instructions and Procedures 38
6.2. Criteria for Postponement of Vaccination 39
6.3. Contraindications to Boost Vaccination 39

7. STUDY VACCINE COMPLIANCE 40

8. PRESTUDY AND CONCOMITANT THERAPY 40

9. STUDY EVALUATIONS 41
9.1. Study Procedures 41
9.1.1. Overview 41
9.1.2. Screening Phase 42
9.1.3. Study Visits 43
9.1.4. VAC52150 Vaccine Development Roll-over Study 46
9.2. Immunogenicity Evaluations 46

9.2.1. Immunogenicity Endpoints ... 46
9.2.2. Immunogenicity Assessments ... 47
9.3. Safety Evaluations ... 47
9.3.1. Safety Endpoints ... 47
9.3.2. Safety Assessments ... 47
9.3.3. Pausing Rules ... 51
9.4. Vaccine-induced Seropositivity ... 52
9.5. Sample Collection and Handling ... 52

10. SUBJECT COMPLETION/DISCONTINUATION OF STUDY VACCINATION/WITHDRAWAL FROM THE STUDY ... 53
10.1. Completion ... 53
10.2. Discontinuation of Vaccinations ... 53
10.3. Withdrawal From the Study ... 53

11. STATISTICAL METHODS ... 55
11.1. Analysis Sets ... 55
11.2. Sample Size Determination ... 56
11.3. Subject Information ... 56
11.4. Immunogenicity Analyses ... 56
11.5. Safety Analyses ... 57
11.6. Interim Analysis ... 57
11.7. Data Review Committee ... 58

12. ADVERSE EVENT REPORTING ... 58
12.1. Definitions ... 58
12.1.1. Adverse Event Definitions and Classifications ... 58
12.1.2. Attribution Definitions ... 59
12.1.3. Severity Criteria ... 60
12.2. Special Reporting Situations ... 61
12.3. Procedures ... 61
12.3.1. All Adverse Events ... 61
12.3.2. Serious Adverse Events ... 62
12.3.3. Pregnancy ... 63
12.4. Contacting Sponsor Regarding Safety ... 63

13. PRODUCT QUALITY COMPLAINT HANDLING ... 63
13.1. Procedures ... 64
13.2. Contacting Sponsor Regarding Product Quality ... 64

14. STUDY VACCINE INFORMATION ... 64
14.1. Description of Study Vaccines ... 64
14.2. Packaging and Labeling ... 65
14.3. Preparation, Handling, and Storage ... 65
14.4. Study Vaccine Accountability ... 65

15. STUDY-SPECIFIC MATERIALS ... 66

16. ETHICAL ASPECTS ... 66
16.1. Study-specific Design Considerations ... 66
16.2. Regulatory Ethics Compliance ... 67
16.2.1. Investigator Responsibilities ... 67
16.2.2. Independent Ethics Committee or Institutional Review Board ... 67
16.2.3. Informed Consent ... 68
16.2.4. Privacy of Personal Data ... 69
16.2.5. Long-term Retention of Samples for Additional Future Research ... 70
16.2.6. Country Selection ... 70

17. ADMINISTRATIVE REQUIREMENTS ... 70

17.1. Protocol Amendments 70
17.2. Regulatory Documentation 71
17.2.1. Regulatory Approval/Notification 71
17.2.2. Required Prestudy Documentation 71
17.3. Subject Identification, Enrollment, and Screening Logs 72
17.4. Source Documentation 72
17.5. Case Report Form Completion 73
17.6. Data Quality Assurance/Quality Control 73
17.7. Record Retention 73
17.8. Monitoring 74
17.9. Study Completion/Termination 75
17.9.1. Study Completion/End of Study 75
17.9.2. Study Termination 75
17.10. On-site Audits 75
17.11. Use of Information and Publication 75

REFERENCES 78

ATTACHMENTS 80

INVESTIGATOR AGREEMENT 88

LAST PAGE 88

LIST OF ATTACHMENTS

Attachment 1: Toxicity Tables for Use in Trials Enrolling Healthy Adults 80
Attachment 2: Batch variability of the Manufacturing Process in Terms of Binding Concentrations Against the EBOV GP 86

LIST OF IN-TEXT TABLES

TABLES

Table 1: Schematic Overview of Study Design and Groups 29
Table 2: Visit Windows 41
Table 3: Overview of Immunogenicity Assessments 47

PROTOCOL AMENDMENT

Amendment_1 (this document)

The overall reason for the amendment: This amendment includes the request of the Center for Biologics Evaluation and Research (CBER, a division of US Food and Drug Administration [FDA]) to extend the long-term safety follow-up to 6 months post-boost.

The changes made to the clinical protocol VAC52150EBL3003, dd. 30-Jul-2015, are listed below, including the rationale of each change and a list of all applicable sections.

Rationale: As requested by the CBER (US FDA), the 6-month visit has been changed to 6-month post-boost visit.

SYNOPSIS
Time and Events Schedule
1.2.5 Overall Benefit/Risk Assessment
3.1 Overview of Study Design
4.3 Prohibitions and Restrictions
5 STUDY VACCINE ALLOCATION AND BLINDING
9.1.1 Overview
9.1.3 Study Visits
10.1 Completion

Rationale: As requested by the CBER (US FDA), the safety laboratory assessments at screening are to be performed within 28 days prior to the prime vaccination and may be repeated if they fall outside this time window.

Time and Events Schedule
4.1 Inclusion Criteria
9.1.2 Screening Phase

Rationale: The time of unblinding and database lock for primary analysis has been changed to when all subjects have completed the 6-month post-boost visit or discontinued earlier.

SYNOPSIS
Time and Events Schedule
1.2.5 Overall Benefit/Risk Assessment
3.1 Overview of Study Design
5 STUDY VACCINE ALLOCATION AND BLINDING
9.1.3 Study Visits
10.1 Completion
11 STATISTICAL METHODS

Rationale: As requested by the CBER (US FDA), the primary analysis will be conducted on immune responses (using ELISA) 56 days post-prime (ie, following the Ad26.ZEBOV vaccination and prior to the MVA-BN-Filo vaccination) as a primary endpoint, instead of 21 days post-boost (ie, after the MVA-BN-Filo vaccination). In addition (per Sponsor proposal), the primary objective has been changed to show equivalence between WVS batch Bern and MVS batch Leiden only (instead of showing equivalence of the 3 batches) since retrospective review of batch allocations for the clinical program showed that these 2 batches would be the only material used in the Phase 2 and 3 trials, making the objective to show equivalence of all 3 batches obsolete. Alternatively, as secondary objectives, immune responses (using ELISA) will be compared for 1) WVS batch Leiden and WVS batch Bern and for 2) WVS batch Leiden and MVS batch Leiden at 56 days post-prime. In addition, the 3 different batches of Ad26.ZEBOV as prime and a single batch of MVA-BN-Filo as boost at a 56-day interval will be compared based on immune responses (using ELISA) at 21 days post-boost.

SYNOPSIS
2 OBJECTIVES AND HYPOTHESIS
9.2.1 Immunogenicity Endpoints
11.2 Sample Size Determination
11.4 Immunogenicity Analyses

Rationale: As requested by the CBER (US FDA), the Per Protocol Analysis set will be used for assessment of the primary objective. The Immunogenicity Analysis set will be used to evaluate the robustness of the analysis results, and for all other analyses of immune response.

11.1 Analysis Sets

Rationale: As requested by the CBER (US FDA), the standard deviation assumed in the sample size calculation is adapted (obtained from clinical study VAC52150EBL1001) and the text on sample size is revised, including a justification of the non-zero difference between batches.

SYNOPSIS
11.2 Sample Size Determination
Attachment 2 Batch variability

Rationale: Information regarding the marketing authorization of MVA-BN and the Phase 3 clinical study POX-MVA-013 has been updated.

1.1 Background
REFERENCES

Rationale: The VAC52150 Vaccine Development Registry was replaced by a roll-over study. Further details regarding enrollment have been added.

SYNOPSIS
3.1 Overview of Study Design
9.1.4 VAC52150 Vaccine Development Roll-over Study
12.3.3 Pregnancy

Rationale: Exploratory immunogenicity endpoints and assessments have been revised and corrected.

9.2.1 Immunogenicity Endpoints
9.2.2 Immunogenicity Assessments

Rationale: The protocol has been updated to be in line with the current protocol template (version 14 October 2015).

4 SUBJECT POPULATION
9.3.2 Safety Assessments
10 SUBJECT COMPLETION/DISCONTINUATION OF STUDY VACCINATION/WITHDRAWAL FROM THE STUDY
12.2 Special Reporting Situations
12.4 Contacting Sponsor Regarding Safety
13.2 Contacting Sponsor Regarding Product Quality
16.2.2 Independent Ethics Committee or Institutional Review Board
16.2.6 Country Selection
17.1 Protocol Amendments
17.4 Source Documentation
17.5 Case Report Form Completion
17.6 Data Quality Assurance/Quality Control
17.7 Record Retention
17.8 Monitoring
17.9.1 Study Completion/End of Study
17.10 On-site Audits
17.11 Use of Information and Publication

Rationale: Minor textual changes have been made.

SYNOPSIS
ABBREVIATIONS
1 INTRODUCTION
2 OBJECTIVES AND HYPOTHESIS
3.1 Overview of Study Design
9.1.1 Overview
9.2.1 Immunogenicity Endpoints
9.3.2 Safety Assessments
10.3 Withdrawal From the Study
11.1 Analysis Sets
11.4 Immunogenicity Analyses
11.5 Safety Analyses
REFERENCES

SYNOPSIS

A Phase 3, Randomized, Double-Blind, Placebo-Controlled Study to Evaluate the Immunogenicity, Safety and Tolerability of a Heterologous Prime-Boost Regimen Using Three Different Batches of Ad26.ZEBOV and a Single Batch of MVA-BN®-Filo in Healthy Adult Subjects

The sponsor, in collaboration with Bavarian Nordic (BN), is investigating the potential of a prophylactic Ebola vaccine regimen comprised of the following 2 candidate Ebola vaccines:

Ad26.ZEBOV is a monovalent vaccine expressing the full length Ebola virus (EBOV, formerly known as *Zaire ebolavirus*) Mayinga glycoprotein (GP), and is produced in the human cell line PER.C6®.

MVA-mBN226B, further referred to as Modified Vaccinia Ankara (MVA)-BN®-Filo, is a multivalent vaccine expressing the Sudan virus (SUDV) GP, the EBOV GP, the Marburg virus (MARV) Musoke GP, and the Tai Forest virus (TAFV, formerly known as *Côte d'Ivoire ebolavirus*) nucleoprotein (NP), and is produced in chicken embryo fibroblast cells. The EBOV GP expressed by MVA-BN-Filo has 100% homology to the one expressed by Ad26.ZEBOV.

In this Phase 3 study, the sponsor's adenovirus serotype 26 (Ad26) vaccine expressing the EBOV Mayinga GP (Ad26.ZEBOV) and the MVA-BN vaccine with EBOV, SUDV and MARV GP inserts and TAFV NP insert (MVA-BN-Filo) will be evaluated as a heterologous prime-boost regimen, in which one study vaccine (Ad26.ZEBOV) is used to prime a filovirus-specific immune response and the other study vaccine (MVA-BN-Filo) is used to boost the immune response. The EBOV GP that is currently circulating in West Africa has 97% homology to the EBOV GP used in this vaccine regimen.

OBJECTIVES AND HYPOTHESIS

Primary Objective

The primary objective is to demonstrate that 2 different batches of Ad26.ZEBOV as prime (working virus seed [WVS] batch Bern and master virus seed [MVS] batch Leiden) induce an equivalent humoral immune response, in terms of geometric mean concentrations (GMC) against the EBOV GP as measured by enzyme-linked immunosorbent assay (ELISA, ELISA U/mL [EU/mL]) 56 days post-prime.

Secondary Objectives

The secondary objectives are:

- To compare the humoral immune response of 2 different batches of Ad26.ZEBOV as prime (WVS batch Leiden and WVS batch Bern; WVS batch Leiden and MVS batch Leiden), in terms of GMC against the EBOV GP as measured by ELISA (EU/mL) 56 days post-prime.
- To compare the humoral immune response of 3 different batches of Ad26.ZEBOV as prime and a single batch of MVA-BN-Filo as boost at a 56-day interval, in terms of GMC against the EBOV GP as measured by ELISA (EU/mL) 21 days post-boost.
- To assess humoral immune responses to the EBOV GP of a heterologous prime-boost regimen of Ad26.ZEBOV and MVA-BN-Filo administered intramuscularly (IM) at a 56-day interval using 3 different batches of Ad26.ZEBOV and a single batch of MVA-BN-Filo as measured by ELISA (EU/mL) at all other time points.
- To assess the safety and tolerability of a heterologous prime-boost regimen of Ad26.ZEBOV and MVA-BN-Filo administered IM at a 56-day interval using 3 different batches of Ad26.ZEBOV and a single batch of MVA-BN-Filo.

Exploratory Objectives

The exploratory objectives are:

- To further explore humoral immune responses to different EBOV GPs and the adenovirus and MVA backbones.
- To explore cellular immune responses to different EBOV GPs using enzyme-linked immunospot (ELISpot) and intracellular cytokine staining (ICS).

Hypothesis

Null Hypothesis:

The GMC of binding antibodies against the EBOV GP 56 days after Ad26.ZEBOV administration from the WVS Ad26.ZEBOV batch produced in Bern is not equivalent to the GMC 56 days after Ad26.ZEBOV administration from the MVS Ad26.ZEBOV batch produced in Leiden.

Alternative Hypothesis:

The GMC of binding antibodies against the EBOV GP 56 days after Ad26.ZEBOV administration from the WVS Ad26.ZEBOV batch produced in Bern is equivalent to the GMC 56 days after Ad26.ZEBOV administration from the MVS Ad26.ZEBOV batch produced in Leiden.

Immunogenic equivalence of the 2 Ad26.ZEBOV batches is shown if the 95% confidence interval (CI) of the ratio of GMC of binding antibodies against the EBOV GP (56 days after prime vaccination) of both batches is entirely within the range 2/3 to 3/2.

OVERVIEW OF STUDY DESIGN

This is a randomized, double-blind, placebo-controlled, parallel-group, multicenter, Phase 3 study to evaluate immunogenic equivalence of a heterologous prime-boost regimen using 3 different batches of Ad26.ZEBOV at a dose of $5x10^{10}$ viral particles (vp) as prime and a single batch of MVA-BN-Filo at a dose of $1x10^{8}$ infectious units (Inf.U) as boost at a 56-day interval in healthy adult subjects in the United States (US). The 3 drug substance batches used in Ad26.ZEBOV drug product have been manufactured, according to the 2x10 L scale process, from WVS in Leiden, The Netherlands manufacturing facility (Group 1), from WVS in Bern, Switzerland manufacturing facility (Group 2) and from MVS in Leiden, The Netherlands manufacturing facility (Group 3, identical to batch #32645 currently used for Phase 2 clinical studies).

Approximately 329 subjects will be enrolled and randomly assigned to one of the 4 groups: to one of 3 groups receiving Ad26.ZEBOV and MVA-BN-Filo (Groups 1 to 3) with approximately 94 subjects per group, or to a placebo group (Group 4) with approximately 47 subjects.

The subject population will consist of healthy men and women aged between 18 and 50 years (inclusive), who have never received a candidate Ebola vaccine and have not had prior exposure to Ebola virus (including travel to West Africa less than 1 month prior to screening) or a diagnosis of Ebola virus disease.

The subjects will be randomized at baseline (on Day 1) in a 2:2:2:1 ratio to Groups 1, 2, 3 and 4. Subjects will be randomized to receive the prime-boost regimen with either Ad26.ZEBOV and MVA-BN-Filo or placebo. Randomization will be stratified by site.

Group	N	Prime	Boost
		Day 1	Day 57
1	94	Ad26.ZEBOV – Batch #33831 (V)	MVA-BN-Filo – Batch #32791 (A)
2	94	Ad26.ZEBOV – Batch #33488 (B)	MVA-BN-Filo – Batch #32791 (A)
3	94	Ad26.ZEBOV – Batch #32642 (C)	MVA-BN-Filo – Batch #32791 (A)
4	47	Placebo (0.9% saline)	Placebo (0.9% saline)

A: batch Kvistgård; B: WVS batch Bern; C: MVS batch Leiden (identical to batch #32645 used for Phase 2 studies); V: WVS batch Leiden; N: planned number of subjects to receive study vaccine
Ad26.ZEBOV dose level is $5x10^{10}$ vp and MVA-BN-Filo dose level is $1x10^8$ Inf.U

Study-site personnel (except for those with primary responsibility for study vaccine preparation and dispensing), sponsor personnel and subjects will be blinded to the study vaccine allocation until the time of database lock for the primary analysis, when all subjects have completed the 6-month post-boost visit or discontinued earlier. See below for details on blinding in case of interim analyses.

All subjects will receive the study vaccine (Ad26.ZEBOV and MVA-BN-Filo or placebo) IM in the deltoid muscle, either Ad26.ZEBOV ($5x10^{10}$ vp, Batch #33831 [V], #33488 [B] or #32642 [C]) on Day 1, followed by a boost vaccination of MVA-BN-Filo ($1x10^8$ Inf.U, Batch #32791 [A]) on Day 57; or placebo (0.9% saline) on Day 1, followed by a boost vaccination of placebo (0.9% saline) on Day 57.

The study consists of a screening phase of up to 6 weeks (starting from the moment the subject signs the informed consent form [ICF]). Subjects will be vaccinated at baseline (Day 1) and at Day 57, and will have follow-up visits at 7 and 28 days post-prime (Days 8 and 29), at 7, 21 and 42 days post-boost (Days 64, 78 and 99) and at 6 months post-boost (Day 237). All subjects will complete a 6-month post-boost visit (Day 237) to further assess safety and immunogenicity.

After completing the present study, subjects who received Ad26.ZEBOV or MVA-BN-Filo will be approached to consent for enrollment into the VAC52150 Vaccine Development Roll-over study (under development) for long-term safety surveillance (at least 4 years after the prime vaccination).

SUBJECT POPULATION

Screening of subjects for eligibility will be performed within 6 weeks before administration of the study vaccine on Day 1. The subject population will consist of healthy (on the basis of medical history, physical examination, electrocardiogram (ECG), vital signs, clinical laboratory testing, and clinical judgment) men and women aged between 18 and 50 years (inclusive), who have not had prior exposure to Ebola virus (including travel to West Africa less than 1 month prior to screening) or a diagnosis of Ebola virus disease. Subjects who have received a candidate Ebola vaccine or an experimental candidate Ad26- or MVA-based vaccine in the past or with known allergy or history of anaphylaxis or other serious adverse reactions to vaccines or vaccine products, including known allergy to egg, egg products and aminoglycosides will be excluded.

DOSAGE AND ADMINISTRATION

All subjects will receive a vaccination, according to randomization, on Day 1 and on Day 57 at the following dose levels:

- Ad26.ZEBOV: $5x10^{10}$ vp, supplied in a single use vial (0.5 mL extractable) (Groups 1, 2, and 3);
- MVA-BN-Filo: $1x10^8$ Inf.U (nominal titer), supplied in a single use vial (0.5 mL extractable) (Groups 1, 2, and 3);
- Placebo: 0.9% saline, 0.5 mL (Group 4).

Study vaccines (Ad26.ZEBOV and MVA-BN-Filo or placebo) will be administered as 0.5-mL IM injections into the deltoid muscle by a blinded study vaccine administrator. The boost vaccination should be administered in the opposite arm from the prime vaccination.

After each vaccination, subjects will remain at the site for a total of 60 (±15) minutes post-vaccination to monitor for the development of any acute reactions, or longer if deemed necessary by the investigator.

Criteria for postponement of vaccination at the scheduled time for vaccine administration and contraindications to boost vaccination have been defined and will be applied by the investigator.

IMMUNOGENICITY EVALUATIONS

The investigator will collect samples for immunogenicity assessments as specified in the Time and Events Schedule, for evaluation of primary, secondary and exploratory endpoints. Samples to assess humoral immune responses will be taken from all subjects; samples to assess cellular immune responses will be taken from subjects at selected site(s) with the capabilities to process peripheral blood mononuclear cells (PBMC, targeted at 10% of all subjects). Subjects giving informed consent for the study will be informed that their leftover blood samples will be stored for potential future research. Subjects participating at selected site(s) where PBMC samples are collected will be asked explicitly to consent for potential future genetic research to be performed on PBMC samples. Subjects can withdraw consent for their samples to be used for future research at any time.

SAFETY EVALUATIONS

Safety evaluations will be performed as specified in the Time and Events Schedule.

Safety will be assessed by collection of solicited local and systemic adverse events (reactogenicity), unsolicited adverse events and serious adverse events. The subjects will be closely observed by study-site personnel for the first 30 (±10) minutes after each vaccination and again at 60 (±15) minutes post-vaccination, and any unsolicited, solicited local or systemic adverse events will be documented during this period. Upon discharge from the site, subjects will receive a diary, a thermometer and a ruler to measure and record body temperature and solicited local reactions. Subjects will be instructed to record solicited local and systemic adverse events in the diary in the evening after each vaccination and then daily for the next 7 days at approximately the same time each day. Diaries should be completed at home by the subject. The investigator will document unsolicited adverse events from signing of the ICF onwards until 42 days post-boost, and serious adverse events from signing of the ICF onwards until the end of the study. The secondary safety endpoints are adverse events, serious adverse events, and solicited local and systemic adverse events. Adverse events that are ongoing at 42 days post-boost vaccination will be followed by the investigator until resolution or stabilization.

Other safety assessments include vital signs (blood pressure, pulse/heart rate, and body temperature), physical examination and pregnancy testing.

The investigators, together with the sponsor's medical monitor, will be responsible for the safety monitoring of the study, and will halt vaccination of further subjects in case any of the pre-specified pausing rules have been met.

STATISTICAL METHODS

Sample size calculations were performed under the following assumptions:

- Immune response is measured by concentrations of binding antibodies against the EBOV GP (using ELISA) 56 days post-prime.
- A standard deviation of 0.323 at the $\log_{10}$ scale (56 days post-prime) in Group 2 (WVS batch Bern) and Group 3 (MVS batch Leiden). This standard deviation is obtained from clinical study VAC52150EBL1001.

- A non-zero difference of 10% in GMC of binding antibodies between batches, expressed as $GMC_{Group\ 2} = 0.9 \times GMC_{Group\ 3}$. This difference is based on the batch variability of the manufacturing process.

With a sample size of 94 subjects per group, and given assumptions, a power of 83% is achieved to conclude equivalence between the WVS Ad26.ZEBOV batch from Bern and the MVS Ad26.ZEBOV batch from Leiden. Equivalence will be shown if the 95% CI of the ratio of GMC of binding antibodies against the EBOV GP is entirely within 2/3 to 3/2. This range has been used in the development of other vaccines for which no correlate of protection has been established. The sample size calculation takes a 10% overall dropout rate into account. A total of 94 subjects per group receiving Ad26.ZEBOV as prime and MVA-BN-Filo as boost, and a control group of approximately 47 subjects receiving placebo results in an overall sample size of approximately 329 subjects.

The primary analysis will be performed when all subjects have completed the last study-related visit (ie, Day 237) or discontinued earlier.

Interim analyses may be performed during the study for the purpose of informing future vaccine-related decisions in a timely manner. The results will not influence the conduct of the study in terms of early termination or later safety or immunogenicity endpoint assessments. Study-site personnel (except for those with primary responsibility for study vaccine preparation and dispensing), the sponsor (except for programming, statistics, clinical and clinical immunology personnel involved in the analysis, and the sponsor committee involved in making future decisions for the program) and subjects will remain blinded to study vaccine allocation until the time of database lock for the primary analysis.

Specific details will be provided in the Statistical Analysis Plan (SAP).

A Data Review Committee (DRC) will be established by the sponsor before the start of the study and will convene to review the available safety data, as outlined in the charter, in case a pausing rule is met. Ad hoc DRC meetings may be requested via the sponsor for any single event or combination of multiple events which are considered to jeopardize the safety of the subjects.

Immunogenicity Analyses

Descriptive statistics (actual values and changes from baseline, including 95% CIs, if applicable) will be calculated for continuous immunologic parameters by time point. Graphical representations of changes in immunologic parameters will be prepared, as applicable. Frequency tabulations will be calculated for discrete (qualitative) immunologic parameters by time point.

To assess the primary objective, only Groups 2 (WVS Ad26.ZEBOV batch from Bern) and 3 (MVS Ad26.ZEBOV batch from Leiden) are considered. For pairwise comparisons of the ELISA concentrations (in EU/mL), estimated differences will be expressed as ratios of GMC with corresponding 95% CI. This 95% CI is determined from comparing $\log_{10}$-transformed ELISA concentrations between groups and back-transformation of the estimated difference and corresponding 95% CI. Equivalence of 2 groups will be shown if the 95% CI of the estimated GMC ratio is entirely within the range of 2/3 to 3/2. Bridging is accomplished if equivalence is shown for the pairwise comparison of Groups 2 and 3.

The primary comparison will be repeated adjusted for sex, age and body weight as a sensitivity analysis.

As an exploratory analysis, response patterns over time for the immunologic parameters will be analyzed, taking into account within-subject correlations.

Safety Analyses

No formal statistical testing of safety data is planned. Adverse events and categorical safety parameters will be tabulated, and continuous safety parameters will be descriptively analyzed.

TIME AND EVENTS SCHEDULE

**Groups 1, 2, 3 and 4**	**Screening Phase[a] (≤6 weeks)**	**Study Visits**							
		**Day 1**	**Day 8**	**Day 29**	**Day 57**	**Day 64**	**Day 78**	**Day 99[b]**	**Day 237[b]**
**Study Procedures**		**Prime**	+7d pp	+28d pp	**Boost**	+7d pb	+21d pb	+42d pb	+6m pb
Screening/Administrative									
Informed consent[c]	X								
Inclusion/exclusion criteria	X[d]								
Medical history and demographics	X								
Prestudy therapies[e]	X								
Serum pregnancy test[f]	X								
Serology (HIV-1/2, hepatitis B/C)	X								
Follicle-stimulating hormone (FSH)[g]	X								
Check clinical status + available data		X[h]							
Randomization		X							
Study vaccine administration[i]		▲			▼				
Safety Assessments									
Urine pregnancy test[f]		X[j]			X[j]				
Physical examination[k]	X	X[j]	X	X	X[j]	X	X	X	X
Electrocardiogram[l]	X								
Vital signs[m]	X	X[j]			X[j]				
Distribution of subject diary[n]		X			X				
Review of subject diary by site staff			X			X			
Adverse events[o]	Continuous								
Serious adverse events	Continuous								
Concomitant therapies	X	X	X	X	X	X	X	X	X[p]
Clinical Laboratory Assessments[q]									
Hematology, chemistry	X								
Urinalysis	X								
Immunogenicity Assessments[r]									
Blood sampling for humoral assays (# mL)		X[j] (40 mL)		X (20 mL)	X[j] (20 mL)		X (100 mL)	X (50 mL)	X (50 mL)
Blood sampling for cellular assays (# mL)		X[j] (60 mL)		X (40 mL)	X[j] (40 mL)		X (60 mL)	X (60 mL)	X (60 mL)

d pp: days post-prime; d pb: days post-boost; m pb: months post-boost;
▲ *Ad26.ZEBOV* $5x10^{10}$ *viral particles (vp) or placebo* ▼ *MVA-BN-Filo* $1x10^{8}$ *infectious units (Inf.U) or placebo.*

NOTE: *If a subject withdraws early from the study, early withdrawal assessments should be obtained per the assessments for the 42-day post-boost visit, with the exception of the immunogenicity assessments. A subject who wishes to withdraw consent from participation in the study will be offered an optional visit for safety follow-up (before formal withdrawal of consent), but the subject has the right to refuse.*

[a] Screening may be split into multiple days or visits. Retesting of values (eg, safety laboratory) that lead to exclusion is allowed once using an unscheduled visit during the screening phase. The safety laboratory assessments at screening are to be performed within 28 days prior to the prime vaccination and may be repeated if they fall outside this time window.

[b] In addition to the assessments scheduled for the 42-day post-boost visit, subjects will be instructed to contact the investigator before the next visit if they experience any adverse event or intercurrent illness that they perceive as relevant and/or can be possibly related to study vaccine in their opinion. All subjects will complete a 6-month post-boost visit (Day 237) to further assess safety and immunogenicity.

[c] Signing of the informed consent form (ICF) needs to be done before the first study-related activity. Minimum criteria for the availability of documentation supporting the eligibility criteria are described in Section 17.4.

[d] The investigators should ensure that all study enrollment criteria have been met at the end of the screening phase.

[e] Prestudy therapies up to 30 days prior to the start of screening and previous vaccinia/smallpox vaccination at any time prior to study entry must be recorded in the case report form (CRF).

[f] For women of childbearing potential.

[g] For women >45 years of age with amenorrhea for less than 2 years or at any age with amenorrhea for more than 6 months.

[h] If a subject's clinical status changes (including available laboratory results or receipt of additional medical records) after screening so the subject no longer meets eligibility criteria, the subject should be excluded from further participation in the study.

[i] After each vaccination, subjects will remain at the site for a total of 60 (±15) minutes post-vaccination to monitor for the development of any acute reactions, or longer if deemed necessary by the investigator. Solicited and unsolicited adverse events emerging during the observation period at the site will be recorded in the CRF.

[j] Prior to study vaccine administration.

[k] A full physical examination, including height and body weight, will be carried out at screening. At other visits, an abbreviated, symptom-directed examination will be performed as indicated by the investigator.

[l] A single, 12-lead electrocardiogram (ECG) (supine) after at least 5 minutes rest will be performed and interpreted locally. Additional ECG monitoring may be done at other time points during the study if clinically indicated based on signs and symptoms. If blood sampling or vital sign measurement is scheduled for the same time point as ECG recording, the procedures should be performed in the following order: vital signs, ECG(s), blood draw.

[m] Includes blood pressure, pulse/heart rate (at least 5 minutes of rest in supine position) and body temperature to be assessed prior to study vaccine administration.

[n] Subjects will use the subject diary to document solicited local and systemic adverse events (reactogenicity) in the evening after each vaccination and then daily for the next 7 days. If a solicited local or systemic adverse event is not resolved on Day 8, the follow-up will be captured on the diary.

[o] Pregnancies will be reported from signing of the ICF until the end of the study.

[p] After the 42-day post-boost visit, concomitant therapies should only be recorded if given in conjunction with serious adverse events.

[q] Blood samples (~15 mL in total) will be collected for serum chemistry and hematology, serology (HIV-1/2, hepatitis B/C) and pregnancy testing.

[r] Blood samples will be collected for humoral assays from all subjects and for cellular assays from subjects at selected site(s) with the capabilities to process peripheral blood mononuclear cells (PBMC) (targeted at 10% of all subjects).

ABBREVIATIONS

Ad26	adenovirus serotype 26 (vector)
Ad26.ZEBOV	adenovirus serotype 26 expressing the Ebola virus Mayinga glycoprotein
ALT	alanine aminotransferase
AST	aspartate aminotransferase
β-hCG	β-human chorionic gonadotropin
BN	Bavarian Nordic
CI	confidence interval
CRF	case report form(s)
DRC	Data Review Committee
EBOV	Ebola virus
ECG	electrocardiogram
eDC	electronic data capture
EDTA	ethylenediaminetetraacetic acid
ELISA	enzyme-linked immunosorbent assay
ELISpot	enzyme-linked immunospot
EU	ELISA units
FSH	follicle stimulating hormone
GCP	Good Clinical Practice
GMC	geometric mean concentration
GP	glycoprotein
HBsAg	hepatitis B surface antigen
HCV	hepatitis C virus
HIV	human immunodeficiency virus
IC	inhibitory concentration
ICF	informed consent form
ICH	International Conference on Harmonisation
ICS	intracellular cytokine staining
IEC	Independent Ethics Committee
IFN-γ	interferon gamma
IL	interleukin
IM	intramuscular(ly)
Inf.U	infectious units
IRB	Institutional Review Board
IWRS	interactive web response system
MARV	Marburg virus
MedDRA	Medical Dictionary for Regulatory Activities
MVA	Modified Vaccinia Ankara
MVA-BN-Filo	Modified Vaccinia Ankara Bavarian Nordic vector expressing multiple filovirus proteins
MVS	master virus seed
NP	nucleoprotein
PBMC	peripheral blood mononuclear cells
PCR	polymerase chain reaction
PQC	Product Quality Complaint
RBC	red blood cell
SAP	Statistical Analysis Plan
SUDV	Sudan virus
SUSAR	suspected unexpected serious adverse reaction
TAFV	Tai Forest virus
$TCID_{50}$	50% tissue culture infective dose
THAM	tris (hydroxymethyl)-amino methane
TNF	tumor necrosis factor
US	United States
VISP	vaccine-induced seropositivity
vp	viral particles
WBC	white blood cell
WVS	working virus seed

DEFINITIONS OF TERMS

Study vaccine	Ad26.ZEBOV, MVA-BN-Filo or placebo.
Blinded study vaccine administrator	A blinded trained study nurse, medical doctor/investigator, or otherwise qualified health care provider.
Independent study vaccine monitor	An unblinded study vaccine monitor assigned to the study who is responsible for the unblinded interface between the sponsor and the investigational site pharmacy.

1. INTRODUCTION

Crucell Holland B.V. (hereafter referred to as the sponsor), in collaboration with Bavarian Nordic (BN), is investigating the potential of a prophylactic Ebola vaccine regimen comprised of the following 2 candidate Ebola vaccines:

Ad26.ZEBOV is a monovalent vaccine expressing the full length Ebola virus (EBOV, formerly known as *Zaire ebolavirus*) Mayinga glycoprotein (GP), and is produced in the human cell line PER.C6®.

MVA-mBN226B, further referred to as Modified Vaccinia Ankara (MVA)-BN®-Filo, is a multivalent vaccine expressing the Sudan virus (SUDV) GP, the EBOV GP, the Marburg virus (MARV) Musoke GP, and the Tai Forest virus (TAFV, formerly known as *Côte d'Ivoire ebolavirus*) nucleoprotein (NP), and is produced in chicken embryo fibroblast cells. The EBOV GP expressed by MVA-BN-Filo has 100% homology to the one expressed by Ad26.ZEBOV.

For the most up-to-date nonclinical and clinical information regarding Ad26.ZEBOV and MVA-BN-Filo, refer to the latest versions of the Investigator's Brochures and Addenda (if applicable).[14,15] A brief summary of the nonclinical and clinical information is provided below.

The term "sponsor" used throughout this document refers to the entities listed in the Contact Information page(s), which will be provided as a separate document.

1.1. Background

Ebola viruses belong to the Filoviridae family and cause Ebola virus disease, which can induce severe hemorrhagic fever in humans and nonhuman primates. Case fatality rates in Ebola disease range from 25% to 90% (average: 50%), according to the World Health Organization.[23] These viruses are highly prioritized by the United States (US) Government, who has defined them as 'Category A' agents, due to the high mortality rate of infected individuals. Currently, no licensed vaccine, treatment or cure exists for this disease.

Filoviruses are named for their long, filamentous shape. Within this filamentous virus, a single 19-kilobase negative-sense ribonucleic acid (RNA) genome encodes 7 proteins: the GP, the polymerase, the NP, the secondary matrix protein, the transcriptional activator, the polymerase cofactor, and the matrix protein. The virion surface is covered by homotrimers of the viral GP, which is believed to be the sole host attachment factor for filoviruses. Following cell entry, the viruses replicate their genomes and viral proteins in the cytoplasm using an RNA-dependent RNA polymerase, which is carried into the cell together with the virus.[9]

Nonclinical Studies

Immunogenicity and Efficacy

Immunogenicity and efficacy of the vaccine combination Ad26.ZEBOV and MVA-BN-Filo was evaluated in a nonhuman primate model (ie, Cynomolgus macaques, *Macaca fascicularis*). The combination was assessed in a multivalent filovirus setting in a small number (2 per regimen) of animals and the study included heterologous prime-boost regimens of adenovirus serotype 26

(Ad26), Ad35 and MVA-BN-Filo vectors expressing different Ebola and Marburg proteins. Full protection from Ebola virus disease and death after wild-type EBOV Kikwit 1995 challenge was obtained with all heterologous regimens, including the Ad26 and MVA vaccine regimen. All heterologous prime-boost regimens induced comparable immune responses against the EBOV Mayinga GP. Independently of the vaccine regimen, a strong boost effect was seen after heterologous prime-boost immunization. Two additional studies involving more animals are ongoing, to strengthen the robustness of the nonclinical efficacy data, and also to optimize the prime-boost schedule so as to obtain induction of protective immunity as quickly as possible, to specifically respond to the Ebola virus disease outbreak in West Africa.

Toxicology

A repeated-dose toxicity study in rabbits was performed with prime-boost combinations of Ad26.ZEBOV and MVA-BN-Filo. The different dose regimens were well tolerated when administered twice by intramuscular (IM) injection to New Zealand White rabbits with a 14-day interval period. Additionally, the objective was to assess the persistence, reversibility or delayed onset of any effects after a 14-day treatment-free period. In the heterologous prime-boost regimen, either vector or both were used to prime a filovirus-specific immune response and the other/same vector or both were used to boost the immune response 2 weeks later. All vaccine dosing regimens resulted in detectable EBOV GP-specific antibody titers. No significant toxicological effects (no adverse effects) were observed. The immune response was associated with transient increases in fibrinogen, C-reactive protein, globulin, decreases in hematocrit and hemoglobin, and microscopic findings in draining iliac lymph nodes, spleen and at the injection sites. The findings were noted to be recovering over a 2-week treatment-free period and were considered to reflect a physiological response associated with vaccination. There were no effects noted that were considered to be adverse.

Biodistribution

Single-dose biodistribution studies in rabbits were performed using the MVA-BN vector or the Ad26 vector in combination with another insert (Ad26.ENVA.01: an experimental, prophylactic Ad26 vector expressing the human immunodeficiency virus [HIV] type 1, Clade A envelope protein). MVA-BN distributed to the skin, muscle, blood, spleen, lung, liver, and pooled lymph nodes and was rapidly cleared (within 48 hours following vaccination). Ad26.ENVA.01 was primarily localized in the injection site muscle, the regional lymph nodes and the spleen. Three months after the single IM injection of Ad26.ENVA.01, the vaccine was cleared from most of the examined tissues. As biodistribution is dependent on the vector platform (MVA or Ad26) and not on the insert, it can be assumed that recombinant MVA-BN-Filo or Ad26.ZEBOV is distributed in the same way as the MVA-BN vector or Ad26.ENVA.01 vector, respectively.

Clinical Studies

To date, no human clinical studies have been completed with Ad26.ZEBOV or MVA-BN-Filo. The safety/tolerability and immunogenicity of the Ad26.ZEBOV and MVA-BN-Filo vaccines are being assessed in the ongoing Phase 1 studies (VAC52150EBL1001, VAC52150EBL1002, VAC52150EBL1003 and VAC52150EBL1004), where monovalent Ad26.ZEBOV and multivalent MVA-BN-Filo are combined in homologous or heterologous prime-boost regimens

in which each vector is used to prime a filovirus-specific immune response followed by a boost immunization with the same or the other vector 2 to 8 weeks later. Two additional Phase 1 studies investigating MVA-BN-Filo are also ongoing (EBL01 and CVD-Mali Ebola Vaccine #1000). Refer to the latest versions of the Ad26.ZEBOV and MVA-BN-Filo Investigator's Brochures and Addenda (if applicable) for more details.[14,15]

Limited data from the ongoing Phase 1 studies with Ad26.ZEBOV and MVA-BN-Filo are available.

VAC52150EBL1001, a first-in-human study, enrolled 87 subjects. Based on the 7-day post-prime safety data (blinded on a treatment group level) on 72 subjects (36 per treatment group), the following information was obtained. Most of the adverse events reported were grade 1 or grade 2 in severity. Local injection site reactions were reported in 18 (50%) MVA/placebo subjects (all grade 1) and 28 (78%) Ad26/placebo subjects (grade 1 [22], grade 2 [5], grade 3 [1]). The most frequent local reaction was injection site pain, in 17 (47%) MVA/placebo subjects and 28 (78%) Ad26/placebo subjects, with one grade 3 case occurring in the Ad26/placebo group. Solicited systemic reactions were reported in 25 (69%) MVA/placebo subjects (grade 1 [24] and grade 2 [1]) and 31 (86%) Ad26/placebo subjects (grade 1 [22], grade 2 [7], grade 3 [1], unknown [1]). The most frequent systemic reactions were fatigue (50% overall), followed by headache (46%) and myalgia (35%). One Ad26/placebo subject experienced 3 grade 3 solicited systemic reactions (headache, myalgia and nausea). None of the subjects reported fever; however, 2 subjects had one temperature measurement missing. The most frequent unsolicited adverse events were decreased neutrophils, in 3 (8%) MVA/placebo subjects and 6 (17%) Ad26/placebo subjects, followed by activated partial thromboplastin time prolongation and hypokalemia, in 3 (8%) MVA/placebo subjects and 5 (14%) Ad26/placebo subjects each. All of these events were transient in nature and resolved without intervention. No deaths or serious adverse events and no adverse events leading to discontinuation of the study vaccination were reported.[14]

Study VAC52150EBL1002 completed enrollment of 128 subjects; the blinded phase of the study is ongoing. No serious adverse events related to study vaccine have been reported and no safety issues have been identified to date.

Safety data generated with the 2 vaccines containing different inserts are provided below:

Safety Profile of Ad26.ZEBOV

Ad26.ZEBOV is a monovalent recombinant, replication-incompetent Ad26-based vaccine. Only limited clinical data are available for Ad26.ZEBOV. However, adenovirus vaccines containing other gene inserts revealed no significant safety issues. The data described below are based on the evaluation of the prototype vaccine Ad26.ENVA.01, which expresses the HIV envelope gene.[11]

Three randomized, placebo-controlled, Phase 1 studies (IPCAVD-001, IPCAVD-003, IPCAVD-004) have evaluated the safety and immunogenicity of the prototype vaccine

Ad26.ENVA.01. This prototype vaccine has been administered to more than 200 healthy, HIV-negative subjects between the ages of 18 and 50 years in the US and Africa.[11,12,13]

- In the dose-escalation study IPCAVD-001 (n=60), 2 or 3 IM doses of Ad26.ENVA.01 ($1x10^9$, $1x10^{10}$, $5x10^{10}$, $1x10^{11}$ viral particles [vp]) were given to Ad26 seronegative subjects. There were no deaths or vaccine-related serious adverse events. Ad26.ENVA.01 was generally well tolerated at all 4 dose levels with minimal reactogenicity observed in the $1x10^9$ and $1x10^{10}$ vp dose groups. Moderate to severe malaise, myalgia, fatigue and chills occurred in the majority of subjects 12 to 18 hours after the first dose of $1x10^{11}$ vp, but were resolved within 24 to 36 hours and were not seen after the second injection at this dose level. Two subjects in the $1x10^{11}$ vp dose group chose not to have the second injection, however, 1 of them decided to have the 6-month injection. Envelope-specific humoral and cell-mediated immune responses were induced at all 4 dose levels of vaccine.[2,4]

- In the single-dose study IPCAVD-003 (n=24), an IM dose of Ad26.ENVA.01 ($5x10^{10}$ vp) or placebo was given to subjects, who were stratified according to baseline Ad26 immune status, to evaluate the safety, mucosal immunogenicity and innate immune responses. Local reactogenicity comprised moderate injection site pain/tenderness and/or moderate to severe erythema which resolved within 3 days of vaccination. Transient systemic reactogenicity comprised headache, chills, joint pain, myalgia, malaise/fatigue, and fever. No deaths or vaccine-related serious adverse events were observed. Vaccination elicited both systemic and mucosal envelope-specific humoral and cellular immune responses. No increased activated total or vector-specific mucosal CD4+ T-lymphocytes following vaccination were detected in the colorectal mucosa, indicating that vaccination with Ad26 did not increase mucosal inflammation.[1]

- In study IPCAVD-004 (n=217), the safety and immunogenicity of IM doses of Ad26.ENVA.01 and Ad35.ENV (an Ad35 vector expressing an HIV envelope GP used in that study at a dose of $5x10^{10}$ vp), given in heterologous and homologous prime-boost regimens at 3- versus 6-month intervals, was evaluated. There were 452 adverse events reported by 84 of 176 Ad26-vaccine recipients (47.7%), the majority being mild (75.5%) in severity. The proportion of subjects with moderate or severe symptoms was not statistically significantly different between vaccine and placebo. There were 3 serious adverse events: 2 serious adverse events in placebo recipients (grade 3 peritonsillar abscess and grade 4 migraine headache, both resolved with no residual effects) and 1 serious adverse event in an Ad35/Ad26 vaccine recipient (grade 4 acute myelogenous leukemia, resolved with sequelae). No deaths or vaccine-related serious adverse events were reported. Overall, 97% to 100% of subjects developed anti-envelope binding antibodies (enzyme-linked immunosorbent assay [ELISA]) after a second dose, with heterologous and homologous regimens being comparable. Immune responses in groups who received 3- and 6-month schedules were comparable. Four weeks post-vaccination, interferon (IFN)-γ enzyme-linked immunospot (ELISpot) assay showed response rates between 44% and 100%. The heterologous and homologous regimens were comparable. There was induction of Ad26-neutralizing antibodies in the majority of vaccine recipients after 2 immunizations with Ad26.ENVA.01.[16]

In addition, the sponsor performed a Phase 1/2a double-blind, randomized, placebo-controlled, dose-escalation study (MAL-V-A001) to evaluate the safety, tolerability and immunogenicity of 2 dose levels ($1x10^{10}$ and $5x10^{10}$ vp) of Ad35.CS.01/Ad26.CS.01 (both expressing the malaria *Plasmodium falciparum* circumsporozoite antigen) prime-boost regimens in healthy subjects. The dose-escalation phase was followed by an evaluation of efficacy of the higher dose level in an experimental malaria challenge. A total of 42 subjects were enrolled and were vaccinated. The analysis of adverse events did not show any consistent pattern suggestive of an association of Ad35.CS.01 or Ad26.CS.01 with specific adverse events. There were no serious adverse events reported during the study. No subject discontinued during a study phase (vaccination or challenge) due to adverse events. One subject in the high-dose group completed the vaccination phase and the final safety follow-up visit but did not take part in any challenge phase activities because of ongoing dyspnea. The most common related adverse events after each vaccination were injection site pain, malaise, headache, myalgia and chills. The incidence of vaccine-related adverse events was generally higher in the high-dose group than in the low-dose group. In general, incidence of malaise, headache, and myalgia were higher after the third dose (Ad26) than after the first or second doses (Ad35). Injection site pain was more commonly reported in the low and high-dose groups than by placebo subjects. There were no clinically significant changes in laboratory test parameters or vital signs data.[5]

Recent data indicate that administration of a deoxyribonucleic acid (DNA) vaccine expressing EBOV Mayinga GP, the same GP as in the Ad26.ZEBOV component, was safe, well tolerated and immunogenic in a Phase 1 clinical study. During this study, 9 subjects received three 4-weekly IM doses of vaccine (4 mg/dose), followed by a boost at ≥32 weeks in 8 subjects.[19]

Safety Profile of MVA-BN

MVA-BN is a further attenuated version of the MVA virus, which in itself is a highly attenuated strain of the poxvirus Chorioallantois Vaccinia Virus Ankara. MVA-BN induces strong cellular activity as well as a humoral (antibody) immune response and has demonstrated an ability to stimulate a response even in individuals with pre-existing immunity against Vaccinia. One of the advantages of MVA-BN is the virus' inability to replicate in a vaccinated individual. The replication cycle is blocked at a very late stage, which ensures that new viruses are not generated and released. This means that the virus cannot spread in the vaccinated person and none of the serious side effects normally associated with replicating Vaccinia viruses have been seen with MVA-BN.

MVA-BN (MVA-BN®, trade name IMVAMUNE® outside the European Union, invented name IMVANEX® in the European Union) has received marketing authorization in the European Union for active immunization against smallpox in adults, and in Canada for persons 18 years of age and older who have a contraindication to the first or second generation smallpox vaccines including individuals with immune deficiencies and skin disorders.[10] A Phase 3 clinical study (POX-MVA-013) has been completed (ClinicalTrials.gov Identifier: NCT01144637).[6] Results of completed and ongoing clinical studies of MVA-BN-based vaccines in more than 8,000 individuals, including elderly, children and immunocompromised subjects in whom replicating vaccines are contraindicated, have shown that the platform displays high

immunogenicity and a favorable safety profile.[17] Across all clinical studies, no trends for unexpected or serious adverse reactions due to the product were detected.

Extensive nonclinical studies support the safety profile of the MVA-BN strain.[20,21]

1.2. Benefit/Risk Section

1.2.1. Known Benefits

The clinical benefit of prime-boost combinations of Ad26.ZEBOV and MVA-BN-Filo is to be established.

1.2.2. Potential Benefits

Subjects may benefit from clinical testing and physical examination; others may benefit from the knowledge that they may aid in the development of an Ebola vaccine. There is no direct individual benefit from vaccination for the subjects at the current development stage.

1.2.3. Known Risks

To date, there are only limited data from the Phase 1 studies with Ad26.ZEBOV and MVA-BN-Filo available. However, Ad26- and Ad35-based vaccines with other gene inserts have been administered to a limited number of human volunteers in clinical studies. These other vaccines mainly elicited some solicited local and systemic reactions, as expected with injectable vaccines, and no serious safety concerns in study participants. MVA-BN-based vaccines have been administered to more than 8,000 individuals without unexpected or serious adverse reactions reported. For details, see the safety data presented in Section 1.1.

1.2.4. Potential Risks

The following potential risks for Ad26.ZEBOV and MVA-BN-Filo will be monitored during the study and are specified in the protocol:

Risks Related to Vaccines

Subjects may exhibit general signs and symptoms associated with administration of a vaccine, or a placebo vaccination, including nausea/vomiting, headache, myalgia, arthralgia, fever, fatigue/malaise and chills. In addition, subjects may experience local (injection site) reactions such as pain/tenderness, erythema, induration/swelling and itching at the injection site. These events will be monitored, but are generally short-term and do not require treatment.

Subjects may have an allergic reaction to the vaccination. An allergic reaction may cause a rash, hives or even difficulty breathing (anaphylaxis). Severe reactions are rare. Medications must be available in the clinic to treat serious allergic reactions.

The risks related to vaccine-induced seropositivity (VISP) are discussed in Section 9.4.

Risk of Myo/Pericarditis

While replicating smallpox vaccines have been associated with an increased risk to develop myo/pericarditis,[18] this has not been observed with MVA-BN and is not expected with this highly attenuated, non-replicating vaccine. Based on observations with first- and second-generation replication-competent smallpox vaccines, particular attention has been placed on the monitoring for cardiac signs and symptoms in all clinical studies using MVA-BN. Despite the close cardiac monitoring, no event indicating a case of myo/pericarditis has been observed in any completed MVA-BN study. In a review of prospective surveillances for cardiac adverse events in 6 different clinical studies in 382 subjects receiving MVA vaccines, only 1 subject (0.3%) met the criteria for vaccine-induced myocarditis and eventually the subject was found to suffer from exercise-induced palpitations. Self-limited mild elevations in troponin I were recorded in 3 subjects (0.8%) without evidence of myo/pericarditis.[7] Based on the current exposure data in more than 8,000 subjects vaccinated with MVA-BN and other MVA-BN recombinant products, the safety profile of MVA-BN has shown to be comparable with other licensed, live attenuated vaccines.

Pregnancy and Birth Control

The effect of the study vaccines on a fetus or nursing baby is unknown, as well as the effect on semen, so women of childbearing potential, and men having sexual intercourse with women, are required to agree to practice adequate birth control measures for sexual intercourse from at least 28 days before the prime vaccination (or prior to enrollment for men) until at least 3 months after the prime vaccination or up to 28 days after the boost vaccination (whichever takes longer) (see Section 4.3). Women who are pregnant or breast-feeding, or are planning to become pregnant while enrolled in the study until 3 months after the prime vaccination or up to 28 days after the boost vaccination (whichever takes longer), will be excluded from enrollment into the study.

Risks from Blood Draws

Blood draws may cause pain/tenderness, bruising, bleeding, and, rarely, infection at the site where the blood is taken.

Unknown Risks

There are no clinical data on the use of either vaccine (Ad26.ZEBOV or MVA-BN-Filo) in:

- Children (<18 years);
- Pregnant or nursing women;
- Adults >50 years;
- Immunocompromised individuals (including those with HIV infection).

There may be other serious risks that are not known.

1.2.5. Overall Benefit/Risk Assessment

Based on the available data and proposed safety measures, the overall benefit/risk assessment for this clinical study is considered acceptable for the following reasons:

Preliminary safety data from the ongoing Phase 1 studies and safety data generated with the 2 vaccines with different inserts revealed no significant safety issues (see Sections 1.1 and 1.2.3). Further experience from Ad26.ZEBOV or MVA-BN-Filo will be gained from currently ongoing clinical studies.

- Only subjects who meet all inclusion criteria and none of the exclusion criteria (specified in Section 4) will be allowed to participate in this study. The selection criteria include adequate provisions to minimize the risk and protect the well-being of subjects in the study.
- Safety will be closely monitored throughout the study:
  - After each vaccination, subjects will remain at the site for a total of 60 (±15) minutes post-vaccination to monitor the development of any acute reactions, or longer if deemed necessary by the investigator. Refer to Section 6.1 for more information on emergency care. The subjects will be closely observed by study-site personnel for the first 30 (±10) minutes after each vaccination and again at 60 (±15) minutes post-vaccination, and any unsolicited, solicited local or systemic adverse events will be documented during this period. Subjects will use a diary to document solicited local and systemic adverse events in the evening after each vaccination and then daily for the next 7 days at approximately the same time each day. Diaries should be completed at home by the subject.
  - The investigator or the designee will document unsolicited adverse events from signing of the informed consent form (ICF) onwards until 42 days post-boost, and serious adverse events from signing of the ICF onwards until the end of the study.
  - Safety evaluations, including an electrocardiogram (ECG; performed at screening and at other time points during the study if clinically indicated based on signs and symptoms), physical examinations, vital sign measurements, clinical laboratory testing (performed at screening) and pregnancy testing, will be performed at scheduled visits during the study, which lasts up to 6 months after the boost vaccination.
  - Any clinically significant abnormalities (including those persisting at the end of the study/early withdrawal) will be followed by the investigator until resolution or until a clinically stable endpoint is reached.
- Several safety measures are included in this protocol to minimize the potential risk to subjects, including the following:
  - The safety evaluations described in Section 9.3 take into account adverse events of special interest based on clinical safety data and available nonclinical data.
  - There are pre-specified pausing rules that would result in pausing of further vaccination if predefined conditions occur, preventing exposure of new subjects to study vaccine until a Data Review Committee (DRC) reviews all safety data (see Section 9.3.3).

- Subjects will discontinue study vaccine for the reasons included in Section 10.2.
- If acute illness (excluding minor illnesses such as diarrhea or mild upper respiratory tract infection) or fever (body temperature ≥38.0°C) occur at the scheduled time for vaccination, the subject may be vaccinated up to 10 days beyond the window allowed for the scheduled vaccination, or be withdrawn from that vaccination at the discretion of the investigator and after consultation with the sponsor (see Section 6.2).
- Contraindications to boost vaccinations are included in Section 6.3.
- If a subject withdraws from the study (withdrawal of consent), he/she maintains the option to participate in the safety follow-up (see Section 10.3).

1.3. Overall Rationale for the Study

In nonclinical studies in the Cynomolgus macaque model, heterologous prime-boost regimens of a multivalent mixture of Ad26 vectors (each expressing EBOV Mayinga, SUDV or MARV GP) and MVA-BN-Filo provided complete protection against the highly pathogenic wild-type EBOV Kikwit 1995 variant (report pending). Further nonclinical studies are ongoing to evaluate the protection of the multivalent vaccine regimen in additional animals and to assess the protective efficacy of a combination regimen of Ad26.ZEBOV and MVA-BN-Filo (either a simultaneous administration or as prime-boost regimen).

In humans, both Ad26- and MVA-based vaccines containing various antigenic inserts have been shown to be safe and immunogenic (see Section 1.1). To date, more than 230 subjects have received the sponsor's Ad26-based vaccines in completed clinical studies (based on the adenoviral vaccine safety database report [dated 20 March 2015]). Up to 28 October 2015, 227 subjects received Ad26.ZEBOV in ongoing studies. The MVA-BN platform is the basis of the non-replicating smallpox vaccine registered in Canada and Europe, and has been safely used in more than 8,000 humans.[17] Although routinely used by the subcutaneous route, MVA-BN at a dose of $1x10^8$ 50% Tissue Culture Infective Dose ($TCID_{50}$) has been demonstrated to be as safe and immunogenic when used by the IM route.[8,22] The IM route has been chosen for the present study.

This study is one of a series of studies to evaluate the heterologous combination of Ad26.ZEBOV and MVA-BN-Filo as a possible vaccine regimen to prevent Ebola virus disease. The concept of a prime-boost regimen that will be evaluated with the candidate prophylactic Ebola vaccines Ad26.ZEBOV and MVA-BN-Filo is supported by the results of clinical studies with candidate malaria vaccines which have demonstrated that Ad-based prime immunization followed by MVA-vector boost induced high levels of immunity. The sponsor's Ad26 vaccine expressing the EBOV Mayinga GP (Ad26.ZEBOV) and the MVA-BN vaccine with EBOV, SUDV and MARV GP inserts and TAFV NP insert (MVA-BN-Filo) will be evaluated as a heterologous prime-boost regimen using Ad26.ZEBOV to prime a filovirus-specific immune response and MVA-BN-Filo to boost the immune response 56 days later. The EBOV GP that is currently circulating in West Africa has 97% homology to the EBOV GP used in this vaccine regimen.[3]

A number of changes have been made to the manufacture of Ad26.ZEBOV to increase capacity. Most notably, the virus seed strategy has changed from a 1 tiered virus seed, based on master virus seed (MVS) to a 2 tiered seed approach, using working virus seed (WVS) and the manufacturing process has been implemented in Bern, Switzerland (in addition to in Leiden, The Netherlands). An initial detailed assessment of the changes, as well as the limited analytical comparability data available to date, indicate that the product from the 3 manufacturing processes (manufacture based on MVS in Leiden, The Netherlands, manufacture based on WVS in Leiden, and manufacture based on WVS in Bern) is very comparable. This clinical bridging study will be performed to support manufacturing variation and specification setting, will help build the safety and immunology database and finally will, if successful, confirm the consistency of the product and process.

This Phase 3 study will be conducted to demonstrate immunogenic equivalence of 3 different batches (WVS batch Leiden, WVS batch Bern and MVS batch Leiden) of Ad26.ZEBOV in a prime-boost regimen of Ad26.ZEBOV as prime and MVA-BN-Filo (single batch) as boost, through the measurement of geometric mean concentrations (GMC) by ELISA. This study will also allow for expansion of the safety experience of the Ad26.ZEBOV/MVA-BN-Filo prime-boost regimen.

2. OBJECTIVES AND HYPOTHESIS

2.1. Objectives

Primary Objective

The primary objective is to demonstrate that 2 different batches of Ad26.ZEBOV as prime (WVS batch Bern and MVS batch Leiden) induce an equivalent humoral immune response, in terms of GMC against the EBOV GP as measured by ELISA (ELISA U/mL [EU/mL]) 56 days post-prime.

Secondary Objectives

The secondary objectives are:

- To compare the humoral immune response of 2 different batches of Ad26.ZEBOV as prime (WVS batch Leiden and WVS batch Bern; WVS batch Leiden and MVS batch Leiden), in terms of GMC against the EBOV GP as measured by ELISA (EU/mL) 56 days post-prime.
- To compare the humoral immune response of 3 different batches of Ad26.ZEBOV as prime and a single batch of MVA-BN-Filo as boost at a 56-day interval, in terms of GMC against the EBOV GP as measured by ELISA (EU/mL) 21 days post-boost.
- To assess humoral immune responses to the EBOV GP of a heterologous prime-boost regimen of Ad26.ZEBOV and MVA-BN-Filo administered intramuscularly (IM) at a 56-day interval using 3 different batches of Ad26.ZEBOV and a single batch of MVA-BN-Filo as measured by ELISA (EU/mL) at all other time points.

- To assess the safety and tolerability of a heterologous prime-boost regimen of Ad26.ZEBOV and MVA-BN-Filo administered IM at a 56-day interval using 3 different batches of Ad26.ZEBOV and a single batch of MVA-BN-Filo.

Exploratory Objectives

The exploratory objectives are:

- To further explore humoral immune responses to different EBOV GPs and the adenovirus and MVA backbones.
- To explore cellular immune responses to different EBOV GPs using ELISpot and intracellular cytokine staining (ICS).

2.2. Hypothesis

Null Hypothesis:

The GMC of binding antibodies against the EBOV GP 56 days after Ad26.ZEBOV administration from the WVS Ad26.ZEBOV batch produced in Bern is not equivalent to the GMC 56 days after Ad26.ZEBOV administration from the MVS Ad26.ZEBOV batch produced in Leiden.

Alternative Hypothesis:

The GMC of binding antibodies against the EBOV GP 56 days after Ad26.ZEBOV administration from the WVS Ad26.ZEBOV batch produced in Bern is equivalent to the GMC 56 days after Ad26.ZEBOV administration from the MVS Ad26.ZEBOV batch produced in Leiden.

Immunogenic equivalence of the 2 Ad26.ZEBOV batches is shown if the 95% confidence interval (CI) of the ratio of GMC of binding antibodies against the EBOV GP (56 days after prime vaccination) of both batches is entirely within the range 2/3 to 3/2.

3. STUDY DESIGN AND RATIONALE

3.1. Overview of Study Design

This is a randomized, double-blind, placebo-controlled, parallel-group, multicenter, Phase 3 study to evaluate immunogenic equivalence of a heterologous prime-boost regimen using 3 different batches of Ad26.ZEBOV at a dose of $5x10^{10}$ vp as prime and a single batch of MVA-BN-Filo at a dose of $1x10^{8}$ infectious units (Inf.U) as boost at a 56-day interval in healthy adult subjects in the US. The 3 drug substance batches used in Ad26.ZEBOV drug product have been manufactured, according to the 2x10 L scale process, from WVS in Leiden, The Netherlands manufacturing facility (Group 1), from WVS in Bern, Switzerland manufacturing facility (Group 2) and from MVS in Leiden, The Netherlands manufacturing facility (Group 3, identical to batch #32645 currently used for Phase 2 clinical studies).

Approximately 329 subjects will be enrolled and randomly assigned to one of the 4 groups: to one of 3 groups receiving Ad26.ZEBOV and MVA-BN-Filo (Groups 1 to 3) with approximately 94 subjects per group, or to a placebo group (Group 4) with approximately 47 subjects.

The subject population will consist of healthy men and women aged between 18 and 50 years (inclusive), who have not had prior exposure to Ebola virus (including travel to West Africa less than 1 month prior to screening) or a diagnosis of Ebola virus disease. Subjects who have received a candidate Ebola vaccine or an experimental candidate Ad26- or MVA-based vaccine in the past or with known allergy or history of anaphylaxis or other serious adverse reactions to vaccines or vaccine products, including known allergy to egg, egg products and aminoglycosides will be excluded.

The subjects will be randomized at baseline (on Day 1) in a 2:2:2:1 ratio to Groups 1, 2, 3 and 4. Subjects will be randomized to receive the prime-boost regimen with either Ad26.ZEBOV and MVA-BN-Filo or placebo. Randomization will be stratified by site. A schematic overview of the study design and groups is provided in Table 1.

Table 1: Schematic Overview of Study Design and Groups

Group	N	Prime	Boost
		Day 1	Day 57
1	94	Ad26.ZEBOV – Batch #33831 (V)	MVA-BN-Filo – Batch #32791 (A)
2	94	Ad26.ZEBOV – Batch #33488 (B)	MVA-BN-Filo – Batch #32791 (A)
3	94	Ad26.ZEBOV – Batch #32642 (C)	MVA-BN-Filo – Batch #32791 (A)
4	47	Placebo (0.9% saline)	Placebo (0.9% saline)

A: batch Kvistgård; B: WVS batch Bern; C: MVS batch Leiden (identical to batch #32645 used for Phase 2 studies); N: planned number of subjects to receive study vaccine; V: WVS batch Leiden
Ad26.ZEBOV dose level is $5x10^{10}$ vp and MVA-BN-Filo dose level is $1x10^{8}$ Inf.U

Study-site personnel (except for those with primary responsibility for study vaccine preparation and dispensing), sponsor personnel and subjects will be blinded to the study vaccine allocation until the time of database lock for the primary analysis, when all subjects have completed the 6-month post-boost visit or discontinued earlier. Refer to Section 5 for details on blinding in case of interim analyses.

The study consists of a screening phase of up to 6 weeks (starting from the moment the subject signs the ICF). Subjects will be vaccinated at baseline (Day 1) and at Day 57, and will have follow-up visits at 7 and 28 days post-prime (Days 8 and 29), at 7, 21 and 42 days post-boost (Days 64, 78 and 99) and at 6 months post-boost (Day 237). All subjects will complete a 6-month post-boost visit (Day 237) to further assess safety and immunogenicity.

The baseline visit may be scheduled as soon as the results of all screening assessments are known (but should occur within 6 weeks from screening, see Section 9.1.2) and show that the subject is eligible for inclusion. The prime vaccination will occur on Day 1 (baseline), after the completion of all baseline assessments.

All subjects will receive the study vaccine (Ad26.ZEBOV and MVA-BN-Filo, or placebo) IM in the deltoid muscle:

- Ad26.ZEBOV ($5x10^{10}$ vp, Batch #33831 [V]) on Day 1, followed by a boost vaccination of MVA-BN-Filo ($1x10^8$ Inf.U, Batch #32791 [A]) on Day 57; *OR*
- Ad26.ZEBOV ($5x10^{10}$ vp, Batch #33488 [B]) on Day 1, followed by a boost vaccination of MVA-BN-Filo ($1x10^8$ Inf.U, Batch #32791 [A]) on Day 57; *OR*
- Ad26.ZEBOV ($5x10^{10}$ vp, Batch #32642 [C]) on Day 1, followed by a boost vaccination of MVA-BN-Filo ($1x10^8$ Inf.U, Batch #32791 [A]) on Day 57; *OR*
- Placebo (0.9% saline) on Day 1, followed by a boost vaccination of placebo (0.9% saline) on Day 57.

Refer to Section 6 for further details on dosage and administration. After each vaccination, subjects will remain at the site for a total of 60 (±15) minutes post-vaccination to monitor for the development of any acute reactions, or longer if deemed necessary by the investigator.

The investigators, together with the sponsor's medical monitor, will be responsible for the safety monitoring of the study, and will halt vaccination of further subjects in case any of the pre-specified pausing rules described in Section 9.3.3 have been met. In addition, discontinuation of study vaccine should occur in any subject meeting the criteria outlined in Section 10.2. Criteria for postponement of vaccination at the scheduled time for vaccine administration and contraindications to boost vaccination have been defined and will be applied by the investigator (see Sections 6.2 and 6.3, respectively).

The investigator will collect samples for immunogenicity assessments (humoral and cellular assays) for the evaluation of primary, secondary and exploratory endpoints as planned by the sponsor (see Table 3 in Section 9.2.2) at the time points indicated in the Time and Events Schedule. Samples to assess humoral immune responses will be taken from all subjects; samples to assess cellular immune responses will be taken from subjects at selected site(s) with the capabilities to process peripheral blood mononuclear cells (PBMC) (targeted at 10% of all subjects). Subjects giving informed consent for the study will be informed that their leftover blood samples will be stored for potential future research. Subjects participating at selected site(s) where PBMC samples are collected will be asked explicitly to consent for potential future genetic research to be performed on PBMC samples. Subjects can withdraw consent for their samples to be used for future research at any time (see Section 16.2.5).

Safety will be assessed by collection of solicited local and systemic adverse events (reactogenicity), unsolicited adverse events and serious adverse events. The subjects will be closely observed by study-site personnel for the first 30 (±10) minutes after each vaccination and again at 60 (±15) minutes post-vaccination, and any unsolicited, solicited local or systemic adverse events will be documented during this period. Upon discharge from the site, subjects will receive a diary, a thermometer and a ruler to measure and record body temperature and solicited

local reactions. Subjects will be instructed to record solicited local and systemic adverse events in the diary in the evening after each vaccination and then daily for the next 7 days at approximately the same time each day. Diaries should be completed at home by the subject. The investigator will document unsolicited adverse events from signing of the ICF onwards until 42 days post-boost, and serious adverse events from signing of the ICF onwards until the end of the study. The secondary safety endpoints are adverse events, serious adverse events, and solicited local and systemic adverse events, see Section 9.3.1.

Other safety assessments include vital signs (blood pressure, pulse/heart rate, and body temperature), physical examination and pregnancy testing at the time points indicated in the Time and Events Schedule.

The primary analysis will be performed when all subjects have completed the last study-related visit (ie, Day 237) or discontinued earlier.

Interim analyses may be performed (as detailed in Section 11.6) during the study for the purpose of informing future vaccine-related decisions in a timely manner. The results will not influence the conduct of the study in terms of early termination or later safety or immunogenicity endpoint assessments.

A DRC will be commissioned for this study. Refer to Section 11.7, Data Review Committee, for details.

After completing the present study, subjects who received Ad26.ZEBOV or MVA-BN-Filo will be approached to consent for enrollment into the VAC52150 Vaccine Development Roll-over study (VAC52150EBL4001) for long-term safety surveillance (at least 4 years after the prime vaccination).

3.2. Study Design Rationale

Control and Blinding

Randomization will be used to minimize bias in the assignment of subjects to groups, to increase the likelihood that known and unknown subject attributes (eg, demographic and baseline characteristics) are evenly balanced across groups, and to enhance the validity of possible statistical comparisons across groups.

Placebo recipients are included for blinding purposes and the assessment of safety, and will provide control specimens for immunogenicity assays. A placebo control will be used to establish the frequency and magnitude of changes in clinical safety endpoints that may occur in the absence of Ad26.ZEBOV and MVA-BN-Filo. The study vaccines (Ad26.ZEBOV and MVA-BN-Filo versus placebo) will be blinded to reduce potential bias during data collection and evaluation of clinical safety endpoints. Blinding will be guaranteed by preparation of study vaccine by unblinded qualified study-site personnel not involved in any other study-related procedure, and by the administration of vaccine in a masked syringe by a blinded study vaccine administrator (see Definitions of Terms).

Study Groups

The prime-boost regimen (Groups 1, 2, and 3) will only differ in the batch of Ad26.ZEBOV, while the timing of the boost vaccination (ie, 56 days after prime) and the sequence of vaccination will be identical. The prime vaccine consists of Ad26.ZEBOV (ie, Batch #33831 [V], Batch #33488 [B], Batch #32642 [C], respectively referred to as Groups 1, 2 and 3) at a similar dose of $5x10^{10}$ vp and a single batch of MVA-BN-Filo (ie, Batch #32791 [A]) at a dose of $1x10^8$ Inf.U as boost. The $1x10^8$ Inf.U dose corresponds to the dose of $1x10^8$ $TCID_{50}$ that is used in the current Phase 1 studies. A control group (Group 4) will be receiving placebo (prime and boost). The safety, tolerability and immunogenicity of the regimen and different batches will be evaluated in the study.

Future Research

Subjects giving informed consent for the study will be informed that their leftover blood samples (serum and/or PBMC) will be stored for potential future research (see Section 16.2.5). Future scientific research may be conducted to further investigate Ebola vaccine- and disease-related questions. This may include the development of new or the improvement of existing techniques to characterize EBOV-directed immune responses or diagnostic tests. Subjects participating at selected site(s) where PBMC samples are collected will be asked explicitly to consent for potential future genetic research to be performed on PBMC samples. Subjects can withdraw consent for their samples to be used for future research at any time.

4. SUBJECT POPULATION

Screening for eligible subjects will be performed within 6 weeks before administration of the study vaccine on Day 1. Signing of the ICF needs to be done before the first study-related activity.

The inclusion and exclusion criteria for enrolling subjects in this study are described in the following 2 subsections. If there is a question about the inclusion or exclusion criteria below, the investigator must consult with the appropriate sponsor representative and resolve any issues before enrolling a subject in the study. Waivers are not allowed.

For a discussion of the statistical considerations of subject selection, refer to Section 11.2, Sample Size Determination.

4.1. Inclusion Criteria

Each potential subject must satisfy all of the following criteria to be enrolled in the study.

1. Signed an ICF indicating that he/she understands the purpose of, and procedures required for, the study and is willing to participate in the study.
2. Man or woman, between 18 and 50 years of age, inclusive, at randomization.
3. Healthy in the investigator's clinical judgment on the basis of medical history, physical examination, ECG and vital signs performed at screening. If the results of these screening tests are outside the normal reference ranges, the subject may be

included only if the investigator judges the abnormalities or deviations from normal to be not clinically significant. This determination must be recorded in the subject's source documents and initialed by the investigator.

4. Healthy on the basis of clinical laboratory tests performed at screening. If the results of the laboratory screening tests are outside the normal reference ranges, the subject may be included only if the investigator judges the abnormalities or deviations from normal to be not clinically significant. This determination must be recorded in the subject's source documents and initialed by the investigator.

   Note: The safety laboratory assessments at screening are to be performed within 28 days prior to the prime vaccination on Day 1 and may be repeated if they fall outside this time window.

   Note: In case of menstruation, urinalysis must be postponed but a result should be available before the prime vaccination.

   Note: If laboratory screening tests are out of range and deemed clinically significant, repeat of screening tests is permitted once using an unscheduled visit during the screening period to assess eligibility.

5. Before randomization, a woman must be either:

   Of childbearing potential and practicing (or intending to practice) a highly effective method of birth control consistent with local regulations regarding the use of birth control methods for subjects participating in clinical studies, beginning at least 28 days prior to vaccination. The sponsor considers the following methods of birth control to be highly effective: established use of oral, injected or implanted hormonal methods of contraception; placement of an intrauterine device or intrauterine system; barrier methods: condom or occlusive cap (diaphragm or cervical/vault caps) with spermicidal foam/gel/film/cream/suppository; male partner sterilization (the vasectomized partner should be the sole partner for that subject); true abstinence (when this is in line with the preferred and usual lifestyle of the subject); *OR*

   - Not of childbearing potential: postmenopausal (>45 years of age with amenorrhea for at least 2 years or any age with amenorrhea for at least 6 months and a serum follicle stimulating hormone (FSH) level >40 IU/L or mIU/mL); permanently sterilized (eg, bilateral tubal occlusion [which includes tubal ligation procedures as consistent with local regulations], hysterectomy, bilateral salpingectomy, bilateral oophorectomy); or otherwise be incapable of pregnancy.

   Note: If the social situation of a woman of childbearing potential changes (eg, woman who is not heterosexually active becomes active), she must begin a highly effective method of birth control, as described above.

6. Woman of childbearing potential must have a negative serum (β-human chorionic gonadotropin [β-hCG]) at screening and a negative urine β-hCG pregnancy test immediately prior to each study vaccine administration.

7. Man who is sexually active with a woman of childbearing potential and has not had a vasectomy performed more than 1 year prior to screening must be willing to use condoms for sexual intercourse beginning prior to enrollment, in addition to the birth control method used by the female partner.

8. Available and willing to participate for the duration of the study and follow-up visits.

9. Willing and able to comply with the protocol requirements, including the prohibitions and restrictions specified in Section 4.3.

10. Willing to provide verifiable identification.

11. Having a means to be contacted.

4.2. Exclusion Criteria

Any potential subject who meets any of the following criteria will be excluded from participating in the study.

1. Having received a candidate Ebola vaccine.

2. Diagnosed with Ebola virus disease, or prior exposure to Ebola virus, including travel to West Africa less than 1 month prior to screening. West Africa includes but is not limited to the countries of Guinea, Liberia, Mali, and Sierra Leone.

3. Having received an experimental candidate Ad26- or MVA-based vaccine in the past.

   Note: Receipt of any approved vaccinia/smallpox vaccine or experimental Ad-vector vaccine other than Ad26 at any time prior to study entry is allowed.

4. Known allergy or history of anaphylaxis or other serious adverse reactions to vaccines or vaccine products (including any of the constituents of the study vaccines [eg, polysorbate 80, ethylenediaminetetraacetic acid (EDTA) or L-histidine for Ad26.ZEBOV vaccine; and tris (hydroxymethyl)-amino methane (THAM) for MVA-BN-Filo vaccine]), including known allergy to egg, egg products and aminoglycosides.

5. Presence of acute illness (this does not include minor illnesses such as diarrhea or mild upper respiratory tract infection) or body temperature ≥38.0ºC on Day 1. Subjects with such symptoms will be excluded from enrollment at that time, but may be rescheduled for enrollment at a later date.

6. Positive hepatitis B surface antigen (HBsAg) or hepatitis C virus (HCV) antibody at screening.

7. HIV type 1 or type 2 infection.

8. Pregnant, breast-feeding, or planning to become pregnant while enrolled in this study until at least 3 months after the prime vaccination or up to 28 days after the boost vaccination (whichever takes longer).

9. Presence of significant conditions or clinically significant findings during screening of

medical history, physical examination, ECG, vital signs or laboratory testing for which, in the opinion of the investigator, participation would not be in the best interest of the subject (eg, compromise the safety or well-being) or that could prevent, limit, or confound the protocol-specified assessments.

10. History of or underlying liver or renal insufficiency or significant cardiac, vascular, pulmonary (eg, persistent asthma), gastrointestinal, endocrine, neurologic, hematologic, rheumatologic, psychiatric, or metabolic disturbances.

11. History of malignancy other than squamous cell or basal cell skin cancer. However, subjects who underwent surgical excision, that is considered cured, can be enrolled.

12. Major surgery (per the investigator's judgment) within the 6 weeks prior to screening, or planned major surgery during the study (from the start of screening onwards).

13. Post-organ and/or stem cell transplant whether or not with chronic immunosuppressive therapy.

14. Received any disallowed therapies as noted in Section 8 before the planned first administration of the prime vaccine on Day 1.

15. Received an investigational drug or investigational vaccines or used an invasive investigational medical device within 3 months prior to screening, or current or planned participation in another clinical study during the study.

    Note: Participation in an observational clinical study is allowed.

16. Donation of a unit of blood within 12 weeks before Day 1 or plans to donate blood during participation in the study (from the start of screening onwards).

17. Receipt of blood products or immunoglobulin within 3 months prior to screening and during participation in the study.

18. Current or past abuse of alcohol, recreational or narcotic drugs, which in the investigator's opinion would compromise the subject's safety and/or compliance with the study procedures.

19. History of chronic urticaria (recurrent hives).

20. Unable to communicate reliably with the investigator.

21. Unlikely to adhere to the requirements of the study in the opinion of the investigator.

22. Employee of the investigator or study site, with direct involvement in the proposed study or other studies under the direction of that investigator or study site, as well as family members of the employees or the investigator.

23. Under legal guardianship or incapacitation.

NOTE: Investigators should ensure that all study enrollment criteria have been met at screening. If a subject's clinical status changes (including available laboratory results or receipt of additional medical records) after screening but before the prime vaccination on Day 1 such that he/she no longer meets all eligibility criteria, then the subject should be excluded from further

participation in the study. Section 17.4, Source Documentation, describes the required documentation to support meeting the enrollment criteria.

4.3. Prohibitions and Restrictions

Potential subjects must be willing and able to adhere to the following prohibitions and restrictions during the course of the study to be eligible for participation:

1. Woman of childbearing potential must remain on a highly effective method of birth control consistent with local regulations regarding the use of birth control methods for subjects participating in clinical studies (see inclusion criteria) until at least 3 months after the prime vaccination or up to 28 days after the boost vaccination (whichever takes longer). If the social situation of a woman of childbearing potential changes (eg, woman who is not heterosexually active becomes active), she must begin a highly effective method of birth control, as described above in Section 4.1, until at least 3 months after the prime vaccination or up to 28 days after the boost vaccination (whichever takes longer).

   Note: A period of 3 months after vaccination with Ad26.ZEBOV and 28 days after vaccination with MVA-BN-Filo should be respected.

   Note: Prior to each study vaccine administration, a urine pregnancy test should be performed for women of childbearing potential.

2. Woman must agree not to donate eggs (ova, oocytes) for the purposes of assisted reproduction from the start of screening onwards until at least 3 months after the prime vaccination or up to 28 days after the boost vaccination (whichever takes longer).

3. Man who has not had a vasectomy performed more than 1 year prior to screening and is sexually active with a woman of childbearing potential must use condoms for sexual intercourse until at least 3 months after the prime vaccination or up to 28 days after the boost vaccination (whichever takes longer), in addition to the birth control method used by the female partner, and must also not donate sperm from the start of the study onwards until at least 3 months after the prime vaccination or up to 28 days after the boost vaccination (whichever takes longer).

4. Woman should not breast-feed while enrolled in the study until at least 3 months after the prime vaccination or up to 28 days after the boost vaccination (whichever takes longer).

5. Not travel to epidemic Ebola areas while enrolled in the study from the start of screening onwards until the 42-day post-boost visit. Subjects who traveled after the 42-day post-boost visit to these areas should have returned at least 1 month before the Day 237 visit. Any traveling to epidemic Ebola areas should be documented in the case report form (CRF).

   Note: Subjects travelling to epidemic Ebola areas will be excluded from follow-up collection of blood for immunogenicity assessments if they contract Ebola virus disease (see also exclusion criterion #2 in Section 4.2.)

6. Not use any disallowed concomitant therapies as described in Section 8.

5. STUDY VACCINE ALLOCATION AND BLINDING

Study Vaccine Allocation

Central randomization will be implemented in this study. Subjects will be randomly assigned in a 2:2:2:1 ratio to Groups 1, 2, 3 and 4 (placebo), respectively, based on a computer-generated randomization schedule prepared before the study by or under the supervision of the sponsor. The randomization will be balanced by using randomly permuted blocks and will be stratified by site. The interactive web response system (IWRS) will assign a unique code, which will dictate the group assignment for the subject. The requestor must use his or her own user identification and personal identification number when contacting the IWRS, and will then give the relevant subject details to uniquely identify the subject.

Blinding

Study-site personnel, sponsor personnel and subjects will be blinded to the study vaccine allocation until the time of database lock for the primary analysis, when all subjects have completed the 6-month post-boost visit or discontinued earlier, except for unblinded qualified study-site personnel with primary responsibility for study vaccine preparation and dispensing, and not involved in any other study-related procedure. The study vaccines will be administered by a blinded study vaccine administrator (see Definitions of Terms).

In case interim analyses will be performed before the primary analysis, study-site personnel (except for those with primary responsibility for study vaccine preparation and dispensing), the sponsor (except programming, statistics, clinical and clinical immunology personnel and the sponsor committee involved in making future decisions for the program) and subjects will remain blinded to study vaccine allocation (see below).

The investigator will not be provided with randomization codes until the time of database lock for the primary analysis. The codes will be maintained within the IWRS, which has the functionality to allow the investigator to break the blind for an individual subject.

The blind should only be broken if specific emergency treatment/course of action would be dictated by knowing the group assignment of the subject. In such cases, the investigator may in an emergency determine the identity of the study vaccine by contacting the IWRS. It is recommended that the investigator contact the sponsor or its designee if possible to discuss the particular situation, before breaking the blind. Telephone contact with the sponsor or its designee will be available 24 hours per day, 7 days per week. In the event the blind is broken, the sponsor must be informed as soon as possible. The date and reason for the unblinding must be documented by the IWRS, in the appropriate section of the CRF, and in the source document. The documentation received from the IWRS indicating the code break must be retained with the subject's source documents in a secure manner.

If the randomization code is broken by the investigator or the study-site personnel, the subject must discontinue further study vaccine administration and must be followed as appropriate (see Section 10.2 for details). If the randomization code is broken by the sponsor for safety reporting purposes, the subject should not discontinue further study vaccine administration and may remain in the study (if the randomization code is still blinded to the study-site personnel and the subject).

Data that may potentially unblind the study vaccine assignment (ie, study vaccine preparation/accountability data, or other specific laboratory data) will be handled with special care to ensure that the integrity of the blind is maintained and the potential for bias is minimized. This can include making special provisions, such as segregating the data in question from view by the investigators, clinical team, or others as appropriate until the time of database lock for the primary analysis and unblinding. The pharmacy and preparation of study vaccines will be monitored by an independent study vaccine monitor (see Section 17.8).

6. DOSAGE AND ADMINISTRATION

An overview of the study vaccines and batches in each group is provided in Table 1.

6.1. General Instructions and Procedures

All subjects will receive a vaccination, according to randomization, on Day 1 and on Day 57 at the following dose levels:

- Ad26.ZEBOV: $5x10^{10}$ vp, supplied in a single use vial (0.5 mL extractable) (Groups 1, 2, and 3);
- MVA-BN-Filo: $1x10^{8}$ Inf.U (nominal titer), supplied in a single use vial (0.5 mL extractable) (Groups 1, 2, and 3);
- Placebo: 0.9% saline, 0.5 mL (Group 4).

Ad26.ZEBOV and MVA-BN-Filo, or placebo will be administered as 0.5-mL IM injections by a blinded study vaccine administrator. The injection site should be free from any injury, local skin conditions, or other issue that might interfere with the evaluation of local reactions (eg, significant tattoo). In each subject, the boost vaccination should be administered in the opposite deltoid from the prime vaccination (unless the opposite arm has a condition that prevents evaluating the arm after injection) and it should be recorded in the CRF in which arm the vaccination has been administered. No local or topical anesthetic will be used prior to the injection.

Discontinuation of study vaccine administration should occur in any subject meeting the criteria outlined in Section 10.2. Criteria for postponement of vaccination at the scheduled time for vaccine administration and contraindications to boost vaccination have been defined in Sections 6.2 and 6.3, respectively. Refer to Section 9.3.3 for details on the pre-specified pausing rules to halt vaccination of further subjects.

After each vaccination, subjects will remain at the site for a total of 60 (±15) minutes post-vaccination to monitor for the development of any acute reactions, or longer if deemed necessary by the investigator. As with any vaccine, allergic reactions following vaccination with the study vaccine are possible. Therefore, appropriate drugs and medical equipment to treat acute anaphylactic reactions must be immediately available and a medically qualified member of study-site personnel trained to recognize and treat anaphylaxis must be present in the clinic during the entire vaccination procedure and post-vaccination monitoring period.

The investigator must provide emergency care as needed for any subject who experiences a life-threatening event. All sites will have facilities, equipment and the ability to manage an anaphylactic reaction. If additional therapy is required, the investigator will arrange for transport to the closest appropriate facility for continuing care.

The Site Investigational Product Procedures Manual specifies the maximum time that will be allowed between preparation and administration of the study vaccine. For storage conditions, please refer to Section 14.3.

6.2. Criteria for Postponement of Vaccination

A subject will not be vaccinated if he/she experiences any of the following events at the scheduled time of vaccination:

- Acute illness (this does not include minor illnesses such as diarrhea or mild upper respiratory tract infection);
- Fever (body temperature ≥38.0°C).

Subjects experiencing any of the events described above may be vaccinated up to 10 days beyond the window allowed for the scheduled vaccination, or be withdrawn from that vaccination at the discretion of the investigator and after consultation with the sponsor (see Section 10.2).

Note: In case the boost vaccination is postponed, the timing of the post-boost visits will be planned relative to the actual vaccination day (see Section 9.1.1).

6.3. Contraindications to Boost Vaccination

A subject will not be given the boost vaccination if he/she experiences any of the following events at any time after the prime vaccination and the sponsor's medical monitor will be notified immediately:

1. Anaphylaxis clearly attributable to vaccination with study vaccine; *OR*
2. Generalized urticaria within 72 hours of vaccination considered to be at least possibly related to study vaccine; *OR*
3. A serious adverse event considered to be at least possibly related to study vaccine; *OR*

4. Injection site ulceration, abscess or necrosis considered to be at least possibly related to study vaccine; *OR*
5. Any other safety concern threatening the subject's safety.

Subjects experiencing any of the events described above must not receive any further study vaccine, but should be monitored for safety and for immunogenicity according to the protocol as described in Section 10.2.

An ad hoc DRC meeting may be requested via the sponsor for any single event or combination of multiple events which are considered to jeopardize the safety of the subjects.

7. STUDY VACCINE COMPLIANCE

All study vaccines will be administered on site by a blinded study vaccine administrator (see Definitions of Terms). The date and time of each study vaccine administration will be recorded in the CRF.

8. PRESTUDY AND CONCOMITANT THERAPY

Prestudy therapies administered up to 30 days prior to the start of screening and previous vaccinia/smallpox vaccination at any time prior to study entry must be recorded in the CRF.

Concomitant therapies must be recorded from screening onwards until the 42-day post-boost visit. Concomitant therapies should also be recorded after the 42-day post-boost visit but only if given in conjunction with serious adverse events that meet the criteria outlined in Section 12.3.2.

All therapies (prescription or over-the-counter medications, including vaccines, vitamins, herbal supplements; non-pharmacologic therapies such as electrical stimulation, acupuncture, special diets, exercise regimens) must be recorded in the CRF. Recorded information will include a description of the type of the drug, treatment period, dosing regimen, route of administration, and its indication.

Subjects must use adequate birth control measures prior to randomization as described in Section 4.

Subjects are allowed to receive all routine immunizations according to local schedules, taking into consideration the following restrictions:

- Inactivated vaccines should be administered at least 15 days before or after administration of any study vaccine to avoid any potential interference in efficacy of the routine immunizations or the interpretation of immune responses to study vaccines, as well as to avoid potential confusion with regard to attribution of adverse events.
- Live attenuated vaccines are prohibited in the period from 30 days before baseline (Day 1) to 30 days after the boost vaccination.

However, if a vaccine is indicated in a post-exposure setting (eg, rabies or tetanus), it must take priority over the study vaccine.

Analgesic/antipyretic medications and non-steroidal anti-inflammatory drugs may be used post-vaccination only in case of medical need (eg, body temperature ≥38.5°C or pain) and their use must be documented. Use of these medications as routine prophylaxis prior to study vaccine administration is prohibited.

Chronic or recurrent use of medications that modify the host immune response (eg, cancer chemotherapeutic agents, systemic corticosteroids, immunomodulators) are prohibited.

The sponsor must be notified in advance (or as soon as possible thereafter) of any instances in which prohibited therapies are administered. Prohibited therapies will be captured as protocol deviations.

9. STUDY EVALUATIONS

9.1. Study Procedures

9.1.1. Overview

The Time and Events Schedule summarizes the frequency and timing of safety, tolerability and immunogenicity measurements and evaluations applicable to this study. Details for all study procedures are provided in the following sections. Additional unscheduled study visits may be required if, in the investigator's opinion, further clinical or laboratory evaluation is needed.

Visit Windows

The screening visit has to be performed within 6 weeks prior to the baseline visit (ie, the day of the subject's prime vaccination, Day 1). If a subject did not receive study vaccine on the planned day of vaccination, the timings of the next visits post-vaccination (see Time and Events Schedule) will be determined relative to the actual day of vaccination. Visit windows that will be allowed are summarized in Table 2. The subject should be encouraged to come in the exact day planned and use the visit window only if absolutely necessary.

Table 2: Visit Windows

Visit Description	Day	Window
Seven Days Post-prime Vaccination	Day 8	±2 days
Twenty-eight Days Post-prime Vaccination	Day 29	±3 days
Boost Vaccination	Day 57	±3 days
Seven Days Post-boost Vaccination	Day 64	±2 days
Twenty-one Days Post-boost Vaccination	Day 78	±3 days
Forty-two Days Post-boost Vaccination	Day 99	±3 days
Six Months Post-boost Vaccination	Day 237	±15 days

Blood Sampling Volumes

Approximately 280 mL of blood (or 600 mL including PBMC samples), excluding the blood volume for clinical laboratory testing at screening, will be drawn over a period of around 8 months, and remains well below the limits of standard blood donation.

Additional serum or urine pregnancy tests may be performed, as determined necessary by the investigator or required by local regulation, to establish the absence of pregnancy at any time during the subject's participation in the study.

Repeat or unscheduled samples may be taken for safety reasons or for technical issues with the samples.

9.1.2. Screening Phase

Up to 6 weeks before baseline (the day of the subject's prime vaccination, Day 1) and after signing and dating the ICF (see Section 16.2.3), screening assessments will be performed as indicated in the Time and Events Schedule. Screening may be split into multiple days or visits. In exceptional cases, the screening phase can be extended if discussed with and approved (documented) by the sponsor, eg, if not all the test results become available during the allocated 6 weeks; this will be evaluated on a case-by-case basis. Screening results from study VAC52150EBL3002 (eg, laboratory and ECG data) can be used if obtained within 6 weeks before the prime vaccination in the current study.

For men and women of non-childbearing potential, there will be no minimum duration of the screening period and it will last only for the time required to verify eligibility criteria. For women of childbearing potential, it should be confirmed that adequate birth control measures were used from at least 28 days before the prime vaccination with a negative serum β-hCG pregnancy test at screening and a negative urine test immediately prior to each study vaccination (see Section 4). All men and women, except for women of non-childbearing potential, will be asked to use adequate birth control for sexual intercourse until at least 3 months after the prime vaccination or up to 28 days after the boost vaccination (whichever takes longer) (see Section 4.3).

Only subjects complying with the criteria specified in Section 4 will be included in the study. The investigator will provide detailed information on the study to the subject and will obtain written informed consent prior to each subject's study participation. The procedures indicated in the Time and Events Schedule will only be performed after the subject's written informed consent has been obtained.

The following is performed to determine eligibility requirements as specified in the inclusion and exclusion criteria:

- Review of all inclusion and exclusion criteria;
- Review of medical history (including concomitant diseases) and demographics;

- Review of prestudy therapies (up to 30 days prior to the start of screening), previous vaccinia/smallpox vaccination if known (at any time prior to study entry) and concomitant therapies;
- Serum pregnancy test (for women of childbearing potential);
- Blood sampling for hematology and chemistry (fasting or non-fasting);
- Urine sampling for urinalysis (dipstick);
- Serology testing (HIV type 1 or type 2, hepatitis B, hepatitis C);
- FSH assessment (for women >45 years of age with amenorrhea for less than 2 years or at any age with amenorrhea for more than 6 months);
- ECG recording;
- Full physical examination (including height and body weight; a genitourinary examination is not required);
- Measurement of vital signs (blood pressure, pulse/heart rate, body temperature).

All adverse events, serious adverse events and pregnancies will be collected from the time a signed and dated ICF is obtained.

The overall eligibility of the subject to participate in the study will be assessed once all screening values and results of any other required evaluations are available. Retesting of values (eg, safety laboratory) that lead to exclusion is allowed once using an unscheduled visit during screening to assess eligibility. The safety laboratory assessments are to be performed within 28 days prior to the prime vaccination and may be repeated if they fall outside this time window. Study subjects who qualify for inclusion will be contacted and scheduled for enrollment and prime vaccination within 6 weeks.

9.1.3. Study Visits

Day 1 Visit (Prime Vaccination)

If eligible, the subject will come for the baseline visit (Day 1). The investigator should ensure that all enrollment criteria have been met during screening. If a subject's clinical status changes (including available laboratory results or receipt of additional medical records) after screening but before the prime vaccination (Day 1) such that the subject no longer meets all enrollment criteria, then the subject should be excluded from further participation in the study. If the initial laboratory sampling occurred more than 6 weeks before baseline (Day 1), sampling will need to be repeated.

A urine pregnancy test (for women of childbearing potential), a symptom-directed physical examination (defined in Section 9.3.2) as indicated by the investigator, and measurements of vital signs will be performed. The eligible subjects will be allocated (by central randomization) to a study group as described in Section 5 and will receive the prime vaccination via IM injection as described in Section 6, unless any of the pre-specified criteria not to proceed with vaccination

are met (refer to Sections 6.2 and 10.2 for details) or if a pause for vaccination of further subjects has been installed (see Section 9.3.3).

Study vaccine will be prepared on-site by unblinded qualified study-site personnel not involved in any other study-related procedure who will place a blinding tape on the syringe to mask its content and send the study vaccine to a blinded study vaccine administrator (see Definitions of Terms) for administration to the subject (see Section 14.3 for details). Refer to Section 6 for further details on dosage and administration and post-vaccination monitoring.

Subjects will have blood drawn for immunogenicity assessments as specified in the Time and Events Schedule. Refer to Section 9.2 for further details on the immunogenicity evaluations.

All adverse events, serious adverse events and pregnancies will be collected and documented on the CRF, together with the information on any concomitant therapies.

Upon discharge from the site, subjects will be provided with a diary, a thermometer, and a ruler to measure and record body temperature and local solicited adverse events. Subjects will also record solicited local and systemic adverse events (reactogenicity) in the diary in the evening after vaccination and then daily for the next 7 days at approximately the same time each day. Diaries should be completed at home by the subject. Subjects will be instructed to contact the investigator immediately in case they experience an unsolicited adverse event (not listed on the diary card) or for any severe (grade 3) solicited adverse event (listed on the diary card).

Days 8 and 29 Visits

Subjects will come to the site at 7 and 28 days after the prime vaccination as indicated in the Time and Events Schedule. The investigator will examine the injection site for occurrences of erythema, induration/swelling, pain/tenderness or itching at this visit in order to complete the relevant parts of the CRF. The subject’s diary will be reviewed by study-site personnel at the 7-day post-prime visit (Day 8). A symptom-directed physical examination (defined in Section 9.3.2) will be performed as indicated by the investigator.

Subjects will have blood drawn for immunogenicity assessments as specified in the Time and Events Schedule. Refer to Section 9.2 for further details on the immunogenicity evaluations.

All adverse events, serious adverse events and pregnancies will be collected and documented on the CRF, together with the information on any concomitant therapies.

Day 57 Visit (Boost Vaccination)

The subjects will receive the boost vaccination via IM injection as described in Section 6, unless any of the pre-specified criteria not to proceed with vaccination are met (refer to Sections 6.2, 6.3 and 10.2 for details) or if a pause for vaccination of further subjects has been installed (see Section 9.3.3).

Before the boost vaccination, a urine pregnancy test (for women of childbearing potential), a symptom-directed physical examination (defined in Section 9.3.2) as indicated by the investigator, and measurements of vital signs will be performed

Subjects will have blood drawn for immunogenicity assessments as specified in the Time and Events Schedule. Refer to Section 9.2 for further details on the immunogenicity evaluations.

The same procedures are applicable as for the prime vaccination regarding vaccine preparation; collection of adverse events, serious adverse events and pregnancies, together with the information on concomitant therapies; and procedures for subjects after discharging.

Days 64, 78 and 99 Visits

Subjects will come to the site at 7, 21 and 42 days after the boost vaccination as indicated in the Time and Events Schedule. The investigator will examine the injection site for occurrences of erythema, induration/swelling, pain/tenderness or itching at these visits in order to complete the relevant parts of the CRF. The subject's diary will be reviewed by study-site personnel at the 7-day post-boost visit (Day 64). A symptom-directed physical examination (defined in Section 9.3.2) will be performed as indicated by the investigator.

Subjects will have blood drawn for immunogenicity assessments at the specified visits in the Time and Events Schedule. Refer to Section 9.2 for further details on the immunogenicity evaluations.

All adverse events, serious adverse events and pregnancies will be collected and documented on the CRF, together with the information on any concomitant therapies.

Subjects will be instructed to contact the investigator before the next visit (ie, on Day 237) if they experience any adverse event or intercurrent illness that they perceive as relevant and/or can be possibly related to study vaccine in their opinion.

Day 237 Visit

All subjects will complete a 6-month post-boost visit (Day 237). A symptom-directed physical examination (defined in Section 9.3.2) will be performed as indicated by the investigator. When all subjects have had their 6-month post-boost visit (Day 237) or discontinued earlier and the database has been locked for the primary analysis, the study will be unblinded.

Subjects will have blood drawn for immunogenicity assessments on Day 237. Refer to Section 9.2 for further details on the immunogenicity evaluations.

Serious adverse event information will be collected until the end of the study, and concomitant therapies should only be recorded if given in conjunction with serious adverse events. Pregnancies will be reported until the end of the study.

9.1.4. VAC52150 Vaccine Development Roll-over Study

After completing the present study, subjects enrolled in this study who received Ad26.ZEBOV or MVA-BN-Filo will be approached to consent for enrollment into the VAC52150 Vaccine Development Roll-over study (under development) for long-term safety surveillance (at least 4 years after the prime vaccination). The parent(s)/legal guardian of children born to female subjects on Ad26.ZEBOV or MVA-BN-Filo who became pregnant during the study up to 3 months after the prime vaccination will also be approached to consent for enrollment of their children into the Roll-over study.

9.2. Immunogenicity Evaluations

9.2.1. Immunogenicity Endpoints

Primary Endpoint:

The primary endpoint is the immunogenicity of Ad26.ZEBOV expressed as:

- Binding antibody levels against the EBOV GP using ELISA (EU/mL) at 56 days after the prime vaccination.

Secondary Endpoints:

- Binding antibody levels against the EBOV GP using ELISA (EU/mL) at all other time points.

Exploratory Endpoints:

Exploratory endpoints may include, but might not be limited to, the following assays:

- Neutralizing antibody responses against the EBOV GP defined as the serum titer that is able to inhibit viral infection by a certain percentage (50%/80% and/or 90% inhibitory concentration [IC_{50}/IC_{80} and/or IC_{90}]) in the virus neutralization assay.
- Binding and/or neutralizing antibody responses against Ad26 and/or MVA vector in ELISA or neutralization assays.
- Humoral immune responses to different EBOV and/or Filovirus GPs, if assays are available.
- Molecular and functional characterization of study vaccine-elicited antibodies, which may include, but will not be limited to, repertoire analysis, Fc characterization, isotype analysis and epitope mapping.
- The presence and functional capacity of T cells will be determined after pathogen-specific stimulation of PBMC with EBOV GP-specific peptides in the IFN-γ ELISpot assay. Cytokine-producing T cells can be quantified using ELISpot technology.
- Activation of CD4+ and CD8+ T cell subsets and their cytokine expression patterns may be determined by flow cytometry (ICS) after EBOV GP-specific stimulation of PBMC (including, but not limited to, IFN-γ, interleukin [IL]-2, and tumor necrosis factor [TNF]-α).

9.2.2. Immunogenicity Assessments

Blood samples for immunogenicity assessments will be collected at the time points and in the volumes indicated in the Time and Events Schedule. Samples for assessment of humoral immune responses will be obtained from all subjects. Samples for assessment of cellular immune responses will be obtained from subjects at selected site(s) with the capabilities to process PBMC (targeted at 10% of all subjects). Table 3 provides an overview of the immunogenicity assessments (humoral and cellular assays).

Table 3: Overview of Immunogenicity Assessments

Sample	Immunogenicity Assessments (non-exhaustive)	Assays (non-exhaustive)
Serum, all subjects	Analysis of antibodies binding to EBOV GP<br>Analysis of neutralizing antibodies to EBOV GP<br>Analysis of binding and/or neutralizing antibodies to adenovirus and/or MVA<br>Analysis of anti-EBOV GP antibody characteristics, including IgG subtyping	ELISA (EU/mL)<br>Virus neutralization assay<br>Adenovirus and/or MVA ELISA and/or neutralization assay<br>Molecular antibody characterization
PBMC, subjects at selected site(s)[a]	T-cell IFN-γ responses to EBOV GP<br>Analysis of T-cell responses to EBOV GP (including CD4/8, IL-2, IFN-γ, TNF-α and/or activation markers)	ELISpot<br>ICS

ELISA: enzyme-linked immunosorbent assay; ELISpot: enzyme-linked immunospot; ICS: intracellular cytokine staining; IFN: interferon; IgG: immunoglobulin G; IL: interleukin; TNF: tumor necrosis factor

[a] with the capabilities to process PBMC (targeted at 10% of all subjects).

9.3. Safety Evaluations

9.3.1. Safety Endpoints

Secondary Endpoints:

- Solicited local and systemic adverse events (reactogenicity) until 7 days post vaccination.
- Adverse events until the 42-day post-boost visit (Day 99).
- Serious adverse events until the end of the study.

9.3.2. Safety Assessments

The study will include the following evaluations of safety and tolerability as described below and according to the time points provided in the Time and Events Schedule. Any clinically significant abnormalities in any of the safety assessments occurring from signing of the ICF onwards until 42 days after the boost vaccination must be recorded on the Adverse Event section of the CRF. Thereafter, reporting will be limited to all serious adverse events. Any clinically significant abnormalities (including those persisting at the end of the study/early withdrawal) will be followed by the investigator until resolution or until a clinically stable endpoint is reached (see Section 12).

The investigators, together with the sponsor's medical monitor, will be responsible for the safety monitoring of the study, and will halt vaccination of further subjects in case any of the

pre-specified pausing rules described in Section 9.3.3 have been met. Further safety measures with regards to vaccination are described in Sections 6.2 and 6.3.

A DRC will be established by the sponsor before the start of the study and will convene to review the available safety data in case a pausing rule is met (as described in Section 9.3.3). Details regarding the DRC are provided in Section 11.7.

Adverse Events

All adverse events, whether serious or non-serious, will be collected at all visits from signing of the ICF onwards until 42 days post-boost. Thereafter, reporting will be limited to all serious adverse events up to the subject's last study-related procedure. Solicited local and systemic adverse events (reactogenicity, see below) will be reported by the subject until 7 days after each administration of study vaccine. Adverse events will be followed by the investigator as specified in Section 12, Adverse Event Reporting.

Solicited Adverse Events

Solicited adverse events are precisely defined events that subjects are specifically asked about and which are noted by subjects in the diary. The subjects will be closely observed by study-site personnel for the first 30 (±10) minutes after each administration of study vaccine and again at 60 (±15) minutes post-vaccination, and any unsolicited, solicited local or systemic adverse events will be documented during this period. Upon discharge from the site, subjects will receive a diary, a thermometer and a ruler to measure and record body temperature and solicited local reactions. Subjects will be instructed to record solicited local and systemic adverse events in the diary in the evening after each administration of study vaccine and then daily for the next 7 days (until Day 8) at approximately the same time each day to serve as a reminder to the subject for the next visit. On Day 8, the diary needs to be completed on site before the subject leaves the site. The investigator should discuss the information from the diary with the subject, document the relevant information in the clinic chart, and complete the relevant parts of the CRF as described in the CRF Completion Guidelines.

On-site and diary reported solicited adverse events will be captured on a separate CRF page as described in the CRF Completion Guidelines, in contrast to the unsolicited adverse events which will be reported on the Adverse Event page of the CRF. The investigator must record in the CRF his/her opinion concerning the relationship of the adverse event to study vaccine.

Solicited Local (Injection Site) Adverse Events

Subjects will also be instructed on how to note occurrences of erythema, induration/swelling (measured using the ruler supplied), pain/tenderness and itching at the injection site in the evening after each administration of study vaccine and then daily for the next 7 days in the diary at approximately the same time each day.

Solicited Systemic Adverse Events

Subjects will be instructed on how to record daily oral body temperature using a thermometer provided for home use. Subjects should record the body temperature in the evening after each

vaccination and then daily for the next 7 days in the diary. Body temperature should be measured at approximately the same time each day. If more than one measurement is made on any given day, the highest value will be recorded in the CRF.

Subjects will also be instructed on how to note the following symptoms in the evening after each administration of study vaccine and then daily for the next 7 days in the diary at approximately the same time each day:

- Nausea/vomiting
- Headache
- Myalgia
- Arthralgia
- Fatigue/malaise
- Chills

If a ***solicited local or systemic adverse event*** is not resolved on Day 8, the follow-up will be captured on the diary. The subject will be instructed to record the date of last symptoms and maximum severity in the diary after resolution.

Adverse Events of Special Interest

Although not a single case of confirmed myocarditis and/or pericarditis has been reported to date following MVA-BN vaccination, due to particular concerns associated with traditional smallpox vaccines, the following adverse events of special interest are defined and must be reported to the appropriate sponsor contact person by study-site personnel within 24 hours of their knowledge of the event (see Section 12.2):

- Any cardiac sign or symptom developed since the boost vaccination;
- ECG changes determined to be clinically significant.

In case of any cardiac sign or symptom, additional ECG might be performed, as deemed necessary by the investigator.

Adverse events of special interest will be reported on the Adverse Event page of the CRF.

Clinical Laboratory Tests

Blood samples (~15 mL in total) for serum chemistry and hematology, serology (HIV-1/2, hepatitis B/C) and pregnancy testing and a urine sample for urinalysis (dipstick) will be collected at screening. Samples can be taken in fasting or non-fasting conditions but should be documented in the CRF. The investigator must review the laboratory results, document this review and record any clinically relevant changes occurring during the study in the Adverse Event section of the CRF. The laboratory reports must be filed with the source documents.

The following tests will be performed by the central laboratory and will only be measured at screening:

- Hematology Panel

  - hemoglobin
  - white blood cell (WBC) count with differential
  - platelet count

  Blood smear: A WBC evaluation may include any abnormal cells, which will then be reported by the laboratory. A hematology evaluation may include abnormalities in the red blood cell (RBC) count and/or RBC parameters and/or RBC morphology, which will then be reported by the laboratory. In addition, any other abnormal cells in a blood smear will also be reported. Only clinically significant abnormal WBC, abnormal RBC, or any other abnormal cells in a blood smear will be reported as adverse events.

- Serum Chemistry Panel

  - sodium
  - potassium
  - blood urea nitrogen
  - aspartate aminotransferase (AST)
  - alanine aminotransferase (ALT)
  - total bilirubin
  - creatinine
  - FSH (only in women >45 years of age with amenorrhea <2 years or at any age with amenorrhea >6 months)

- Urinalysis – Dipstick for:

  - glucose
  - ketones
  - protein
  - blood

  In case of positive urinalysis dipstick results, the sediment will be examined microscopically (only RBC will be documented).

Additional clinical laboratory assessments to be performed are as follows:

- Serum pregnancy test for women of childbearing potential at screening;
- Urine pregnancy test for women of childbearing potential before each study vaccination;
- Serology (HIV type 1 and type 2 antibody, HBsAg, and HCV antibody) at screening.

Any abnormal laboratory values will be graded according to the toxicity grading tables in Attachment 1 if applicable for laboratory tests.

Electrocardiogram (ECG)

A single, 12-lead ECG will be performed at screening and interpreted locally. Additional ECG monitoring may be done at other time points during the study if clinically indicated based on signs and symptoms.

During the collection of ECGs, subjects should be in a quiet setting without distractions (eg, television, cell phones). Subjects should rest in a supine position for at least 5 minutes before ECG collection and should refrain from talking or moving arms or legs. If blood sampling or vital sign measurement is scheduled for the same time point as ECG recording, the procedures should be performed in the following order: vital signs, ECG(s), blood draw.

Vital Signs (blood pressure, pulse/heart rate, body temperature)

Vital sign measurements will be performed at the time points indicated in the Time and Events Schedule. Blood pressure and pulse/heart rate measurements will be assessed (at rest in supine position) with a completely automated device. Manual techniques will be used only if an automated device is not available. Confirmatory measurements can be performed if inconsistent with a prior measurement. Body temperature is preferably measured orally. If the body temperature was measured at another site this needs to be captured in the CRF.

Physical Examination

A full physical examination, including height and body weight, will be performed by the investigator at screening. A full physical examination includes the following: general appearance, eyes, ears, nose, throat, cardiovascular system, respiratory system, gastrointestinal system, and skin and mucous membranes. A neurological and musculoskeletal examination as well as an examination of the lymph nodes will also be performed. The height should be measured barefooted at the screening visit. To obtain the actual body weight, subjects must be weighed lightly clothed.

After screening, an abbreviated, symptom-directed physical examination will be performed as indicated by the investigator based on any clinically relevant issues, clinically relevant symptoms and medical history. The symptom-directed physical examination may be repeated at other visits if deemed necessary by the investigator. An abbreviated, symptom-directed physical examination may include inspection of the vaccination site(s).

9.3.3. Pausing Rules

The investigators and the sponsor's medical monitor will review the blinded safety data of enrolled subjects on an ongoing basis. The sponsor's medical monitor will be involved in all discussions and decisions.

If any of the following events occur in any subject who received at least one dose of study vaccine in the study (at any site), that site investigator will halt the vaccination of further subjects and the sponsor's medical monitor will be notified immediately. The sponsor's medical monitor will then also inform all the other investigators to halt further vaccination as well.

1. Death in any subject considered at least possibly related to study vaccine; *OR*

2. An anaphylactic reaction within 24 hours of vaccination or the presence of generalized urticaria within 72 hours of vaccination in any subject considered at least possibly related to study vaccine; *OR*

3. A life-threatening or other serious adverse event in any subject considered at least possibly related to study vaccine.

For the events described above, the sponsor's medical monitor notifies the DRC immediately and dosing will be halted. Within 3 business days, the DRC will convene to review the available safety data as outlined in the charter and to advise whether vaccination may resume, or additional safety data are needed, eventually ask for a protocol amendment, or discontinue further vaccination or suspend the study. The sites will be allowed to resume activities upon receipt of a written notification from the sponsor. The communications from the DRC will be forwarded by the investigator to the Independent Ethics Committee (IEC)/Institutional Review Board (IRB) according to local standards and regulations and health authorities will be notified of any pause and the DRC recommendation.

Any event that meets the criteria of a serious adverse event should be recorded on the Serious Adverse Event page of the CRF (see Section 12.3.2).

9.4. Vaccine-induced Seropositivity

In general, uninfected subjects who participate in Ebola vaccine studies may develop Ebola-specific antibodies as a result of an immune response to the candidate Ebola vaccine, referred to as VISP. These antibodies may be detected in Ebola serologic tests, causing the test to appear positive even in the absence of actual Ebola infection. VISP may become evident during the study, or after the study has been completed. The potential of a study participant becoming polymerase chain reaction (PCR)-positive after vaccination is being assessed in a Phase 1 study (VAC52150EBL1002).

Subjects should not donate blood during participation in the study (from the start of screening onwards; see Section 4.2).

Consent will be obtained to contact the doctors that the subject sees regularly, to let them know that the subject is taking part in this study. It is important for all of the subject's doctors to know that the subject may be administered experimental vaccines. Subjects participating in the study will be provided with a "wallet (study) card" and instructed to carry this card with them for the duration of the study (see Section 12.3.1).

9.5. Sample Collection and Handling

The actual dates and times of sample collection must be recorded in the CRF or laboratory requisition form. Refer to the Time and Events Schedule for the timing and frequency of all sample collections.

Sample collection and processing will be performed by the study-site personnel according to current versions of approved standard operating procedures.

Instructions for the collection, handling, storage, and shipment of samples will be provided in the Laboratory Manual. Collection, handling, storage, and shipment of samples must be under the specified, and where applicable, controlled temperature conditions as indicated in the Laboratory Manual.

10. SUBJECT COMPLETION/DISCONTINUATION OF STUDY VACCINATION/WITHDRAWAL FROM THE STUDY

10.1. Completion

A subject will be considered to have completed the study if he or she has completed all assessments at the 6-month post-boost visit.

10.2. Discontinuation of Vaccinations

If a subject's study vaccine must be discontinued before the end of the vaccination schedule, this will not result in automatic withdrawal of the subject from the study.

A subject's study vaccine (prime or boost) may be discontinued at the discretion of the investigator and after consultation with the sponsor for any of the events in Section 6.2.

A subject's study vaccine should be **permanently** discontinued if:

- The investigator believes that for safety reasons (eg, adverse event) it is in the best interest of the subject to withhold from further administration of study vaccines;
- The subject or the partner of a male subject becomes pregnant;
- The subject has confirmed Ebola virus disease through natural exposure to the virus (eg, by travel to an affected country);
- The subject experiences any of the events described in Section 6.3;
- The randomization code is broken by the investigator or the study-site personnel.

Subjects meeting any of the reasons listed above must not receive any further study vaccine, but should continue to be monitored for safety and for immunogenicity according to the protocol if this does not result in safety risks for the subject. In case of early discontinuation of study vaccine due to an adverse event, the investigator will collect all information relevant to the adverse event and safety of the subject, and will follow the subject to resolution, or until reaching a clinically stable endpoint.

10.3. Withdrawal From the Study

Each subject has the right to withdraw from the study at any time for whatever reason. The investigator should make an attempt to contact subjects who did not return for scheduled visits or follow-up. Although the subject is not obliged to give reason(s) for withdrawing early, the

investigator should make a reasonable effort to ascertain the reason(s) while fully respecting the subject's rights.

A subject will be withdrawn from the study for any of the following reasons:

- Decision by the investigator to withdraw a subject for repeated failure to comply with protocol requirements;
- Decision by the sponsor to stop or cancel the study;
- Decision by local regulatory authorities and IEC/IRB to stop or cancel the study;
- Lost to follow-up;
- Withdrawal of consent;
- Death.

If a subject withdraws early from the study for any of the reasons listed above (except in case of death), early withdrawal assessments should be obtained per the assessments for the 42-day post-boost visit, with the exception of the immunogenicity assessments. A subject who wishes to withdraw consent from participation in the study will be offered an optional visit for safety follow-up (before formal withdrawal of consent), but the subject has the right to refuse.

If a subject is lost to follow-up, every reasonable effort must be made by the study-site personnel to contact the subject and determine the reason for discontinuation/withdrawal. The measures taken to follow up must be documented.

When a subject withdraws before completing the study, the reason for withdrawal is to be documented in the CRF and in the source document. Study vaccine assigned to the withdrawn subject may not be assigned to another subject. For subjects who withdraw from the study after randomization but before the prime vaccination, an additional subject will be enrolled. Subjects who withdraw from the study after receiving the prime vaccination will not be replaced.

A subject who withdraws from the study will have the following options for storage of samples for potential future use:

- The collected samples will be retained and used in accordance with the subject's original informed consent for storage of samples for future use.
- The subject may withdraw consent for storage of samples for potential future use (see Section 16.2.5), in which case the samples will be destroyed and no further testing will take place. To initiate the sample destruction process, the investigator must notify the sponsor study site contact of withdrawal of consent for the storage of leftover samples for future research and request sample destruction. The sponsor study site contact will, in turn, contact the biomarker representative to execute sample destruction. If requested, the investigator will receive written confirmation from the sponsor that the samples have been destroyed. Details of the sample retention for research are presented in the ICF.

Withdrawal From Storage of Samples for Future Use While Remaining in the Study

The subject may withdraw consent for storage of samples for future use (refer to Section 16.2.5) while remaining in the study. In such a case, the samples will be destroyed after they are no longer needed for the clinical study as described above. Details of the sample retention for research are presented in the ICF.

11. STATISTICAL METHODS

Statistical analysis will be done by the sponsor or under the authority of the sponsor. A general description of the statistical methods to be used to analyze the safety and immunogenicity data is outlined below. Specific details will be provided in the Statistical Analysis Plan (SAP).

The primary analysis will be conducted when all subjects have completed the 6-month post-boost visit or discontinued earlier. Refer to Section 5 for details on blinding.

Interim analyses may be performed as described in Section 11.6.

11.1. Analysis Sets

The Full Analysis set includes all subjects who were randomized and received at least 1 dose of study vaccine, regardless of the occurrence of protocol deviations. Safety data will be analyzed based on the Full Analysis set.

The Immunogenicity Analysis set includes all randomized and vaccinated subjects, who have data from baseline and at least 1 post-vaccination immunogenicity blood draw.

The Per Protocol Analysis set includes all randomized and vaccinated subjects, who received both the prime and boost vaccinations, have data from baseline and at least 1 post-vaccination immunogenicity blood draw, and experienced no major protocol violations that have an influence on the immune response.

For assessment of the primary objective, the analysis population is the Per Protocol Analysis set. As a sensitivity analysis, the analysis of the primary endpoint will also be based on the Immunogenicity Analysis set to evaluate the robustness of the analysis results. For all other analyses of immune response, the analysis population is the Immunogenicity Analysis set.

11.2. Sample Size Determination

Sample size calculations are performed under the following assumptions:

– Immune response is measured by concentrations of binding antibodies against the EBOV GP (using ELISA) 56 days post-prime.
– A standard deviation of 0.323 at the log_{10} scale (56 days post-prime) in Group 2 (WVS batch Bern) and Group 3 (MVS batch Leiden). This standard deviation is obtained from clinical study VAC52150EBL1001.
– A non-zero difference of 10% in GMC of binding antibodies between batches, expressed as $GMC_{Group\ 2} = 0.9 \times GMC_{Group\ 3}$. This difference is based on the batch variability of the manufacturing process (see Attachment 2).

With a sample size of 94 subjects per group, and given assumptions; a power of 83% is achieved to conclude equivalence between the WVS Ad26.ZEBOV batch from Bern and the MVS Ad26.ZEBOV batch from Leiden. Equivalence will be shown if the 95% CI of the ratio of GMC of binding antibodies against the EBOV GP is entirely within 2/3 to 3/2. This range has been used in the development of other vaccines for which no correlate of protection has been established. The sample size calculation takes a 10% overall dropout rate into account. A total of 94 subjects per group receiving Ad26.ZEBOV as prime and MVA-BN-Filo as boost, and a control group of approximately 47 subjects receiving placebo, results in an overall sample size of approximately 329 subjects.

11.3. Subject Information

For all subjects, demographic characteristics (eg, age, height, weight, body mass index, race and sex) and screening/baseline characteristics (eg, physical examination, medical history) will be summarized using descriptive statistics and/or listed.

11.4. Immunogenicity Analyses

Descriptive statistics (actual values and changes from baseline, including 95% CIs, if applicable) will be calculated for continuous immunologic parameters by time point. Graphical representations of changes in immunologic parameters will be prepared, as applicable. Frequency tabulations will be calculated for discrete (qualitative) immunologic parameters by time point.

To assess the primary objective, only Groups 2 (WVS Ad26.ZEBOV batch from Bern) and 3 (MVS Ad26.ZEBOV batch from Leiden) are considered. For pairwise comparisons of the ELISA concentrations (in EU/mL), estimated differences will be expressed as ratios of GMC with corresponding 95% CI. This 95% CI is determined from comparing log_{10}-transformed ELISA concentrations between groups and back-transformation of the estimated difference and corresponding 95% CI. Equivalence of 2 groups will be shown if the 95% CI of the estimated GMC ratio is entirely within the range of 2/3 to 3/2. Bridging is accomplished if equivalence is shown for the pairwise comparison of Groups 2 and 3.

The primary comparison will be repeated adjusted for sex, age and body weight as a sensitivity analysis.

As an exploratory analysis, response patterns over time for the immunologic parameters will be analyzed, taking into account within-subject correlations.

11.5. Safety Analyses

No formal statistical testing of safety data is planned. Adverse events and categorical safety parameters will be tabulated, and continuous safety parameters will be descriptively analyzed.

Baseline for all safety parameters will be defined as the last value before the prime vaccination.

Adverse Events (Including Reactogenicity)

The verbatim terms used in the CRF by investigators to report adverse events will be coded using the Medical Dictionary for Regulatory Activities (MedDRA). All reported adverse events (solicited local, solicited systemic, and unsolicited) will be included in the analysis. For each adverse event, the number and percentage of subjects who experience at least 1 occurrence of the given event will be summarized by group. Summaries, listings, datasets, or subject narratives may be provided, as appropriate, for those subjects who die, who discontinue study vaccine due to an adverse event, or who experience a severe or a serious adverse event.

The analysis for solicited adverse events will be done on those subjects in the Full Analysis set for whom reactogenicity assessments are available in the database. The analysis of unsolicited adverse events, including (but not limited to) adverse events of special interest, serious adverse events, adverse events leading to discontinuation and fatal adverse events, will be done on the Full Analysis set.

Clinical Laboratory Tests (Pregnancy Tests)

The results of the serum and urine pregnancy tests will be summarized.

Vital Signs

Descriptive statistics of blood pressure (systolic and diastolic), pulse/heart rate and body temperature values and changes from baseline will be summarized at each scheduled time point. The percentage of subjects with values beyond clinically important limits will be summarized.

Physical Examination

Physical examination findings will be listed.

11.6. Interim Analysis

Interim analyses may be performed during the study for the purpose of informing future vaccine-related decisions in a timely manner. The results will not influence the conduct of the study in terms of early termination or later safety or immunogenicity endpoint assessments. Study-site personnel (except for those with primary responsibility for study vaccine preparation and dispensing), the sponsor (except for programming, statistics, clinical and clinical immunology

personnel involved in the analysis, and the sponsor committee involved in making future decisions for the program) and subjects will remain blinded to study vaccine allocation until the time of database lock for the primary analysis. Refer to Section 5 for further details on blinding.

A separate interim SAP will be prepared before the conduct of an interim analysis.

11.7. Data Review Committee

A DRC will be established by the sponsor before the start of the study and will convene to review the available safety data in case a pausing rule is met (as described Section 9.3.3). Ad hoc DRC meetings may be requested via the sponsor for any single event or combination of multiple events which are considered to jeopardize the safety of the subjects. After the review, the DRC will make recommendations regarding the continuation of the study. The details will be provided in a separate DRC charter.

12. ADVERSE EVENT REPORTING

Timely, accurate, and complete reporting and analysis of safety information from clinical studies are crucial for the protection of subjects, investigators, and the sponsor, and are mandated by regulatory agencies worldwide. The sponsor has established Standard Operating Procedures in conformity with regulatory requirements worldwide to ensure appropriate reporting of safety information; all clinical studies conducted by the sponsor or its affiliates will be conducted in accordance with those procedures.

12.1. Definitions

12.1.1. Adverse Event Definitions and Classifications

Adverse Event

An adverse event is any untoward medical occurrence in a clinical study subject administered a medicinal (investigational or non-investigational) product. An adverse event does not necessarily have a causal relationship with the treatment. An adverse event can therefore be any unfavorable and unintended sign (including an abnormal finding), symptom, or disease temporally associated with the use of a medicinal (investigational or non-investigational) product, whether or not related to that medicinal (investigational or non-investigational) product. (Definition per International Conference on Harmonisation [ICH])

This includes any occurrence that is new in onset or aggravated in severity or frequency from the baseline condition, or abnormal results of diagnostic procedures, including laboratory test abnormalities.

Note: The sponsor collects adverse events starting with the signing of the ICF (refer to Section 12.3.1 for time of last adverse event recording).

Serious Adverse Event

A serious adverse event based on ICH and European Union Guidelines on Pharmacovigilance for Medicinal Products for Human Use is any untoward medical occurrence that at any dose:

- Results in death
- Is life-threatening
  (The subject was at risk of death at the time of the event. It does not refer to an event that hypothetically might have caused death if it were more severe.)
- Requires inpatient hospitalization or prolongation of existing hospitalization
- Results in persistent or significant disability/incapacity
- Is a congenital anomaly/birth defect
- Is a suspected transmission of any infectious agent via a medicinal product
- Is Medically Important*

*Medical and scientific judgment should be exercised in deciding whether expedited reporting is also appropriate in other situations, such as important medical events that may not be immediately life-threatening or result in death or hospitalization but may jeopardize the subject or may require intervention to prevent one of the other outcomes listed in the definition above. These should usually be considered serious.

If a serious and unexpected adverse event occurs for which there is evidence suggesting a causal relationship between the study vaccine and the event (eg, death from anaphylaxis), the event must be reported as a suspected unexpected serious adverse reaction (SUSAR) (even after the study is over, if the sponsor, DRC or investigator becomes aware of them) by the sponsor to the Health Authorities and by the investigator to the IEC/IRB according to regulatory and local requirements.

Unlisted (Unexpected) Adverse Event/Reference Safety Information

An adverse event is considered unlisted if the nature or severity is not consistent with the applicable product reference safety information. For Ad26.ZEBOV and MVA-BN-Filo, the expectedness of an adverse event will be determined by whether or not it is listed in the Investigator's Brochures and Addenda, if applicable.

Adverse Event Associated With the Use of the Study Vaccine

An adverse event is considered associated with the use of the study vaccine if the attribution is **possibly**, **probably**, or **very likely** by the definitions listed in Section 12.1.2.

12.1.2. Attribution Definitions

Every effort should be made by the investigator to explain any adverse event and to assess its potential causal relationship, ie, to administration of the study vaccine or to alternative causes, eg, natural history of underlying disease(s), concomitant drug(s). This applies to all adverse events, whether serious or non-serious. Assessment of causality must be done by a licensed study physician (the investigator or designee).

The investigator will use the following guidelines to assess the causal relationship of an adverse event to study vaccine:

Not Related

An adverse event that is not related to the use of the vaccine.

Doubtful

An adverse event for which an alternative explanation is more likely, eg, concomitant drug(s), concomitant disease(s), or the relationship in time suggests that a causal relationship is unlikely.

Possible

An adverse event that might be due to the use of the vaccine. An alternative explanation, eg, concomitant drug(s), concomitant disease(s), is inconclusive. The relationship in time is reasonable; therefore, the causal relationship cannot be excluded.

Probable

An adverse event that might be due to the use of the vaccine. The relationship in time is suggestive. An alternative explanation is less likely, eg, concomitant drug(s), concomitant disease(s).

Very Likely

An adverse event that is listed as a possible adverse reaction and cannot be reasonably explained by an alternative explanation, eg, concomitant drug(s), concomitant disease(s). The relationship in time is very suggestive.

12.1.3. Severity Criteria

Adverse events will be coded for severity using the toxicity grading tables in Attachment 1. For adverse events not identified in the table, the following guidelines will apply:

**Mild**	Grade 1	Symptoms causing no or minimal interference with usual social and functional activities
**Moderate**	Grade 2	Symptoms causing greater than minimal interference with usual social and functional activities
**Severe**	Grade 3	Symptoms causing inability to perform usual social and functional activities

12.2. Special Reporting Situations

Safety events of interest on a sponsor study vaccine that may require expedited reporting or safety evaluation include, but are not limited to:

- Administration of an overdose of study vaccine
- Accidental or occupational exposure to a study vaccine
- Administration error involving a study vaccine (with or without subject/patient exposure to the study vaccine, eg, name confusion)
- Adverse event of special interest (see Section 9.3.2)

Special reporting situations should be recorded in the CRF. Any special reporting situation that meets the criteria of a serious adverse event should be recorded on the Serious Adverse Event page of the CRF.

12.3. Procedures

12.3.1. All Adverse Events

All adverse events and special reporting situations, whether serious or non-serious, will be reported from the time a signed and dated ICF is obtained until the 42-day post-boost visit. Serious adverse events will be collected from signing of the ICF onwards until the end of the study. Subjects will record symptoms of solicited local or systemic adverse events (reactogenicity) in the diary in the evening after each vaccination and then daily for the next 7 days. Serious adverse events must be reported by the investigator using the Serious Adverse Event Form. SUSARs will be reported even after the study is over, if the sponsor, the DRC or the investigator becomes aware of them. The sponsor will evaluate any safety information that is spontaneously reported by an investigator beyond the time frame specified in the protocol.

All adverse events, regardless of seriousness, severity, or presumed relationship to study vaccine, must be recorded using medical terminology in the source document and the CRF. Whenever possible, diagnoses should be given when signs and symptoms are due to a common etiology (eg, cough, runny nose, sneezing, sore throat, and head congestion should be reported as "upper respiratory infection"). Investigators must record in the CRF their opinion concerning the relationship of the adverse event to study vaccine. All measures required for adverse event management must be recorded in the source document and reported according to sponsor instructions. Solicited adverse events will be captured on a separate CRF page as described in the CRF Completion Guidelines. Unsolicited adverse events which will be reported on the Adverse Event page of the CRF.

The sponsor assumes responsibility for appropriate reporting of adverse events to the regulatory authorities. The sponsor will also report to the investigator (and the head of the investigational institute where required) all SUSARs. The investigator (or sponsor where required) must report SUSARs to the appropriate IEC/IRB that approved the protocol unless otherwise required and documented by the IEC/IRB. A SUSAR will be reported to regulatory authorities unblinded.

Participating investigators and IEC/IRB will receive a blinded SUSAR summary, unless otherwise specified.

For all studies with an outpatient phase, including open-label studies, the subject must be provided with a "wallet (study) card" and instructed to carry this card with them for the duration of the study indicating the following:

- Study number
- Statement, in the local language(s), that the subject is participating in a clinical study
- Investigator's name and 24-hour contact telephone number
- Local sponsor's name and 24-hour contact telephone number (for medical staff only)
- Site number
- Subject number
- Any other information that is required to do an emergency breaking of the blind

12.3.2. Serious Adverse Events

All serious adverse events occurring during the study must be reported to the appropriate sponsor contact person by study-site personnel within 24 hours of their knowledge of the event.

Information regarding serious adverse events will be transmitted to the sponsor using the Serious Adverse Event Form, which must be completed and signed by a physician from the study site, and transmitted to the sponsor within 24 hours. The initial and follow-up reports of a serious adverse event should be made by facsimile (fax).

All serious adverse events that have not resolved by the end of the study, or that have not resolved upon discontinuation of the subject's participation in the study, must be followed until any of the following occurs:

- The event resolves
- The event stabilizes
- The event returns to baseline, if a baseline value/status is available
- The event can be attributed to agents other than the study vaccine or to factors unrelated to study conduct
- It becomes unlikely that any additional information can be obtained (subject or health care practitioner refusal to provide additional information, lost to follow-up after demonstration of due diligence with follow-up efforts)

Suspected transmission of an infectious agent by a medicinal product will be reported as a serious adverse event. Any event requiring hospitalization (or prolongation of hospitalization) that occurs during the course of a subject's participation in a study must be reported as a serious adverse event, except hospitalizations for the following:

- Hospitalizations not intended to treat an acute illness or adverse event (eg, social reasons such as pending placement in long-term care facility)
- Surgery or procedure planned before entry into the study (must be documented in the CRF). Note: Hospitalizations that were planned before the signing of the ICF, and where the underlying condition for which the hospitalization was planned has not worsened, will not be considered serious adverse events. Any adverse event that results in a prolongation of the originally planned hospitalization is to be reported as a new serious adverse event.

The cause of death of a subject in a study, whether or not the event is expected or associated with the study vaccine, is considered a serious adverse event.

12.3.3. Pregnancy

Pregnancies will be reported from signing of the ICF until the end of the study.

All initial reports of pregnancy in female subjects or partners of male subjects must be reported to the sponsor by the study-site personnel within 24 hours of their knowledge of the event using the appropriate pregnancy notification form. Abnormal pregnancy outcomes (eg, spontaneous abortion, fetal death, stillbirth, congenital anomalies, ectopic pregnancy) are considered serious adverse events and must be reported using the Serious Adverse Event Form. Any subject or partner of a male subject who becomes pregnant during the study must be promptly withdrawn from further study vaccination but should continue participation in the study for safety follow-up.

Because the effect of the study vaccine on sperm is unknown, pregnancies in partners of male subjects included in the study will be reported by the study-site personnel within 24 hours of their knowledge of the event using the appropriate pregnancy notification form.

Follow-up information regarding the outcome of the pregnancy and any postnatal sequelae in the infant will be required.

All female subjects who received Ad26.ZEBOV or MVA-BN-Filo on Ad26.ZEBOV or MVA-BN-Filo who become pregnant during the study up to 3 months after the prime vaccination will be approached to consent for enrollment of their children into the VAC52150 Vaccine Development Roll-over study (see Section 9.1.4).

12.4. Contacting Sponsor Regarding Safety

The names (and corresponding telephone numbers) of the individuals who should be contacted regarding safety issues or questions regarding the study are listed in the Contact Information page(s), which will be provided as a separate document.

13. PRODUCT QUALITY COMPLAINT HANDLING

A product quality complaint (PQC) is defined as any suspicion of a product defect related to manufacturing, labeling, or packaging, ie, any dissatisfaction relative to the identity, quality, durability, or reliability of a product, including its labeling or package integrity. A PQC may have an impact on the safety and efficacy of the product. Timely, accurate, and complete reporting and

analysis of PQC information from studies are crucial for the protection of subjects, investigators, and the sponsor, and are mandated by regulatory agencies worldwide. The sponsor has established procedures in conformity with regulatory requirements worldwide to ensure appropriate reporting of PQC information; all studies conducted by the sponsor or its affiliates will be conducted in accordance with those procedures.

13.1. Procedures

All initial PQC reports must be reported to the sponsor by the study-site personnel within 24 hours after being made aware of the event.

If the defect is combined with a serious adverse event, the study-site personnel must report the PQC to the sponsor according to the serious adverse event reporting timelines (refer to Section 12.3.2). A sample of the suspected product should be maintained for further investigation if requested by the sponsor.

13.2. Contacting Sponsor Regarding Product Quality

The names (and corresponding telephone numbers) of the individuals who should be contacted regarding product quality issues are listed in the Contact Information page(s), which will be provided as a separate document.

14. STUDY VACCINE INFORMATION

14.1. Description of Study Vaccines

Ad26.ZEBOV

Ad26.ZEBOV is a monovalent recombinant, replication-incompetent Ad26-based vector that expresses the full length EBOV Mayinga GP and is produced in the human cell line PER.C6®.

The 3 substance batches used in Ad26.ZEBOV drug product are manufactured, according to the 2x10 L scale process, from WVS in Leiden, The Netherlands manufacturing facility (Group 1), from WVS in Bern, Switzerland manufacturing facility (Group 2) and from MVS in Leiden, The Netherlands manufacturing facility (Group 3, identical to batch #32645 currently used for Phase 2 clinical studies).

The Ad26.ZEBOV vaccine will be supplied at a concentration of 1×10^{11} vp/mL in 2-mL single-use glass vials as a frozen liquid suspension to be thawed before use. Each vial contains an extractable volume of 0.5 mL. Refer to the Investigator's Brochure for a list of excipients.[14]

MVA-BN-Filo

MVA-BN-Filo is a recombinant multivalent vaccine intended for active immunization against Ebola and Marburg virus infection. MVA-BN-Filo is strongly attenuated; the vaccine is propagated in primary chicken embryo fibroblast cells and does not replicate in human cells.

The MVA-BN-Filo drug substance, starting from WVS is manufactured in Kvistgård, Denmark manufacturing facility (Groups 1 to 3).

The MVA-BN-Filo vaccine will be supplied at a concentration of $2x10^8$ Inf.U/mL (nominal titer) in 2 mL glass vials as a frozen liquid suspension to be thawed before use. Each vial contains an extractable volume of 0.5 mL. Refer to the Investigator’s Brochure for a list of excipients.[15]

Placebo

The placebo supplied for this study will be formulated as a sterile 0.9% saline for injection (as commercially available).

14.2. Packaging and Labeling

All study vaccines will be manufactured and packaged in accordance with Good Manufacturing Practice. All study vaccines will be packaged and labeled under the responsibility of the sponsor. No study vaccine can be repacked or relabeled on site without prior approval from the sponsor.

Further details for study vaccine packaging and labeling can be found in the Site Investigational Product Procedures Manual.

14.3. Preparation, Handling, and Storage

Study vaccine must be stored at controlled temperatures: Ad26.ZEBOV vials must be stored at ≤ -60°C and MVA-BN-Filo vials must be stored at ≤ -20°C.

Vials must be stored in a secured location with no access for unauthorized personnel. All study product storage equipment (including refrigerators, freezers) must be equipped with a continuous temperature monitor and alarm, and with back-up power systems. In the event that study vaccine is exposed to temperatures outside the specified temperature ranges, all relevant data will be sent to the sponsor to determine if the affected study vaccine can be used or will be replaced. The affected study vaccine must be quarantined and not used until further instruction from the sponsor is received.

Blinding will be achieved by preparation of study vaccine by unblinded qualified study-site personnel not involved in any other study-related procedure, and by the administration of vaccine in a masked syringe by a blinded study vaccine administrator (see Definitions of Terms).

Details on the preparation, the holding time and storage conditions from the time of preparation to administration of Ad26.ZEBOV and MVA-BN-Filo are provided in the Site Investigational Product Procedures Manual.

14.4. Study Vaccine Accountability

The investigator is responsible for ensuring that all study vaccine received at the site is inventoried and accounted for throughout the study. The study vaccine administered to the subject must be documented on the study vaccine accountability form. All study vaccine will be stored and disposed of according to the sponsor's instructions. Study-site personnel must not combine contents of the study vaccine containers.

Study vaccine must be handled in strict accordance with the protocol and the container label, and must be stored at the study site in a limited-access area or in a locked cabinet under appropriate environmental conditions. Unused study vaccine must be available for verification by the sponsor's study-site monitor during on-site monitoring visits. The return to the sponsor of unused study vaccine will be documented on the study vaccine return form. When the study site is an authorized destruction unit and study vaccine supplies are destroyed on-site, this must also be documented on the study vaccine return form.

Potentially hazardous materials such as used ampules, needles, syringes and vials containing hazardous liquids should be disposed of immediately in a safe manner and therefore will not be retained for study vaccine accountability purposes.

Study vaccine should be dispensed under the supervision of the investigator or a qualified member of the study-site personnel, or by a qualified staff member. Study vaccine will be supplied only to subjects participating in the study. Returned study vaccine must not be dispensed again, even to the same subject. Study vaccine may not be relabeled or reassigned for use by other subjects. The investigator agrees neither to dispense the study vaccine from, nor store it at, any site other than the study sites agreed upon with the sponsor.

15. STUDY-SPECIFIC MATERIALS

The investigator will be provided with the following supplies:

- Investigator’s Brochures and Addenda (if applicable) for Ad26.ZEBOV and MVA-BN-Filo
- Site Investigational Product Procedures Manual
- Laboratory Manual
- IWRS Manual
- Electronic Data Capture (eDC) Manual/electronic CRF Completion Guidelines and Randomization Instructions
- Sample ICF
- Subject diaries
- Rulers, thermometers
- Subject wallet cards
- Recruitment tools, as applicable

16. ETHICAL ASPECTS

16.1. Study-specific Design Considerations

Potential subjects will be fully informed of the risks and requirements of the study and, during the study, subjects will be given any new information that may affect their decision to continue participation. They will be told that their consent to participate in the study is voluntary and may be withdrawn at any time with no reason given and without penalty or loss of benefits to which they

would otherwise be entitled. Only subjects who are fully able to understand the risks, benefits, and potential adverse events of the study, and provide their consent voluntarily will be enrolled.

The total blood volume to be collected is considered to be within the limits of standard blood donation.

16.2. Regulatory Ethics Compliance

16.2.1. Investigator Responsibilities

The investigator is responsible for ensuring that the study is performed in accordance with the protocol, current ICH guidelines on Good Clinical Practice (GCP), and applicable regulatory and country-specific requirements.

Good Clinical Practice is an international ethical and scientific quality standard for designing, conducting, recording, and reporting studies that involve the participation of human subjects. Compliance with this standard provides public assurance that the rights, safety, and well-being of study subjects are protected, consistent with the principles that originated in the Declaration of Helsinki, and that the study data are credible.

16.2.2. Independent Ethics Committee or Institutional Review Board

Before the start of the study, the investigator (or sponsor where required) will provide the IEC/IRB with current and complete copies of the following documents (as required by local regulations):

- Final protocol and, if applicable, amendments
- Sponsor-approved ICF (and any other written materials to be provided to the subjects)
- Investigator's Brochure (or equivalent information) and amendments/addenda
- Sponsor-approved subject recruiting materials
- Information on compensation for study-related injuries or payment to subjects for participation in the study, if applicable
- Investigator's curriculum vitae or equivalent information (unless not required, as documented by the IEC/IRB)
- Information regarding funding, name of the sponsor, institutional affiliations, other potential conflicts of interest, and incentives for subjects
- Any other documents that the IEC/IRB requests to fulfill its obligation

This study will be undertaken only after the IEC/IRB has given full approval of the final protocol, amendments (if any, excluding the ones that are purely administrative, with no consequences for subjects, data or study conduct, unless required locally), the ICF, applicable recruiting materials, and subject compensation programs, and the sponsor has received a copy of this approval. This approval letter must be dated and must clearly identify the IEC/IRB and the documents being approved.

During the study the investigator (or sponsor where required) will send the following documents and updates to the IEC/IRB for their review and approval, where appropriate:

- Protocol amendments (excluding the ones that are purely administrative, with no consequences for subjects, data or study conduct)
- Revision(s) to ICF and any other written materials to be provided to subjects
- If applicable, new or revised subject recruiting materials approved by the sponsor
- Revisions to compensation for study-related injuries or payment to subjects for participation in the study, if applicable
- New edition(s) of the Investigator's Brochure and amendments/addenda
- Summaries of the status of the study at intervals stipulated in guidelines of the IEC/IRB (at least annually)
- Reports of adverse events that are serious, unlisted/unexpected, and associated with the study vaccine
- New information that may adversely affect the safety of the subjects or the conduct of the study
- Deviations from or changes to the protocol to eliminate immediate hazards to the subjects
- Report of deaths of subjects under the investigator's care
- Notification if a new investigator is responsible for the study at the site
- Development Safety Update Report and Line Listings, where applicable
- Any other requirements of the IEC/IRB

For all protocol amendments (excluding the ones that are purely administrative, with no consequences for subjects, data or study conduct), the amendment and applicable ICF revisions must be submitted promptly to the IEC/IRB for review and approval before implementation of the change(s).

At least once a year, the IEC/IRB will be asked to review and reapprove this study, where required.

At the end of the study, the investigator (or sponsor where required) will notify the IEC/IRB about the study completion (if applicable, the notification will be submitted through the head of investigational institution).

16.2.3. Informed Consent

Each subject must give written consent according to local requirements after the nature of the study has been fully explained. The ICF(s) must be signed before performance of any study-related activity. The ICF(s) that is/are used must be approved by both the sponsor and by the reviewing IEC/IRB and be in a language that the subject can read and understand. The informed consent should be in accordance with principles that originated in the Declaration of Helsinki, current ICH and GCP guidelines, applicable regulatory requirements, and sponsor policy.

Before enrollment in the study, the investigator or an authorized member of the study-site personnel must explain to potential subjects the aims, methods, reasonably anticipated benefits, and potential hazards of the study, and any discomfort participation in the study may entail. Subjects will be informed that their participation is voluntary and that they may withdraw consent to participate at any time. They will be informed that choosing not to participate will not affect the care the subject will receive. Finally, they will be told that the investigator will maintain a subject identification register for the purposes of long-term follow up if needed and that their records may be accessed by health authorities and authorized sponsor personnel without violating the confidentiality of the subject, to the extent permitted by the applicable law(s) or regulations. By signing the ICF the subject is authorizing such access, and agrees to allow his or her study physician to recontact the subject for the purpose of obtaining consent for additional safety evaluations, if needed.

The subject will be given sufficient time to read the ICF and the opportunity to ask questions. After this explanation and before entry into the study, consent should be appropriately recorded by means of the subject's personally dated signature. After having obtained the consent, a copy of the ICF must be given to the subject.

If the subject is unable to read or write, an impartial witness should be present for the entire informed consent process (which includes reading and explaining all written information) and should personally date and sign the ICF after the oral consent of the subject is obtained.

16.2.4. Privacy of Personal Data

The collection and processing of personal data from subjects enrolled in this study will be limited to those data that are necessary to fulfill the objectives of the study.

These data must be collected and processed with adequate precautions to ensure confidentiality and compliance with applicable data privacy protection laws and regulations. Appropriate technical and organizational measures to protect the personal data against unauthorized disclosures or access, accidental or unlawful destruction, or accidental loss or alteration must be put in place. Sponsor personnel whose responsibilities require access to personal data agree to keep the identity of subjects confidential.

The informed consent obtained from the subject includes explicit consent for the processing of personal data and for the investigator/institution to allow direct access to his or her original medical records (source data/documents) for study-related monitoring, audit, IEC/IRB review, and regulatory inspection. This consent also addresses the transfer of the data to other entities and to other countries.

The subject has the right to request through the investigator access to his or her personal data and the right to request rectification of any data that are not correct or complete. Reasonable steps will be taken to respond to such a request, taking into consideration the nature of the request, the conditions of the study, and the applicable laws and regulations.

Exploratory immunogenicity research is not conducted under standards appropriate for the return of data to subjects. In addition, the sponsor cannot make decisions as to the significance of any findings resulting from exploratory research. Therefore, exploratory research data will not be returned to subjects or investigators, unless required by law or local regulations. Privacy and confidentiality of data generated in the future on stored samples will be protected by the same standards applicable to all other clinical data.

16.2.5. Long-term Retention of Samples for Additional Future Research

Samples collected in this study for which consent has been obtained and for which additional material is available after study-specified testing is complete, may be stored for up to 15 years (or according to local regulations) for possible additional future scientific/genetic research. Samples will only be used to understand Ebola vaccine- and disease-related questions, and to develop tests/assays related to the characterization of EBOV-directed immune responses or diagnostic tests. The research may begin at any time during the study or the post-study storage period. Applicable approvals will be sought before any such samples are used for analysis not specified in the protocol (amendment) approved by the IEC/IRB.

Stored samples will be coded throughout the sample storage and analysis process and will not be labeled with personal identifiers. Subjects may withdraw their consent for their samples to be stored for future research at any time (refer to Section 10.3). In such case, their blood samples will be destroyed after all the tests specified for this study have been concluded.

The sponsor will be responsible for the overall management of the sample inventory, shipping plan, allocation and storage of samples.

16.2.6. Country Selection

This study will only be conducted in those countries where the intent is to launch or otherwise help ensure access to the developed product if the need for the product persists, unless explicitly addressed as a specific ethical consideration in Section 16.1.

17. ADMINISTRATIVE REQUIREMENTS

17.1. Protocol Amendments

Neither the investigator nor the sponsor will modify this protocol without a formal amendment by the sponsor. All protocol amendments must be issued by the sponsor, and signed and dated by the investigator. Protocol amendments must not be implemented without prior IEC/IRB approval, or when the relevant competent authority has raised any grounds for non-acceptance, except when necessary to eliminate immediate hazards to the subjects, in which case the amendment must be promptly submitted to the IEC/IRB and relevant competent authority. Documentation of amendment approval by the investigator and IEC/IRB must be provided to the sponsor. When the change(s) involve(s) only logistic or administrative aspects of the study, the IRB/IEC (where required) only needs to be notified.

During the course of the study, in situations where a departure from the protocol is unavoidable, the investigator or other physician in attendance will contact the appropriate sponsor representative (listed in the Contact Information page(s), which will be provided as a separate document). Except in emergency situations, this contact should be made before implementing any departure from the protocol. In all cases, contact with the sponsor must be made as soon as possible to discuss the situation and agree on an appropriate course of action. The data recorded in the CRF and source documents will reflect any departure from the protocol, and the source documents will describe this departure and the circumstances requiring it.

17.2. Regulatory Documentation

17.2.1. Regulatory Approval/Notification

This protocol and any amendment(s) must be submitted to the appropriate regulatory authorities in each respective country, if applicable. A study may not be initiated until all local regulatory requirements are met.

17.2.2. Required Prestudy Documentation

The following documents must be provided to the sponsor before shipment of study vaccine to the study site:

- Protocol and amendment(s), if any, signed and dated by the principal investigator
- A copy of the dated and signed (or sealed, where appropriate per local regulations), written IEC/IRB approval of the protocol, amendments, ICF, any recruiting materials, and if applicable, subject compensation programs. This approval must clearly identify the specific protocol by title and number and must be signed (or sealed, where appropriate per local regulations) by the chairman or authorized designee.
- Name and address of the IEC/IRB, including a current list of the IEC/IRB members and their function, with a statement that it is organized and operates according to GCP and the applicable laws and regulations. If accompanied by a letter of explanation, or equivalent, from the IEC/IRB, a general statement may be substituted for this list. If an investigator or a member of the study-site personnel is a member of the IEC/IRB, documentation must be obtained to state that this person did not participate in the deliberations or in the vote/opinion of the study.
- Regulatory authority approval or notification, if applicable
- Signed and dated statement of investigator (eg, Form FDA 1572), if applicable
- Documentation of investigator qualifications (eg, curriculum vitae)
- Completed investigator financial disclosure form from the principal investigator, where required
- Signed and dated clinical trial agreement, which includes the financial agreement
- Any other documentation required by local regulations

The following documents must be provided to the sponsor before enrollment of the first subject:

- Completed investigator financial disclosure forms from all subinvestigators
- Documentation of subinvestigator qualifications (eg, curriculum vitae)
- Name and address of any local laboratory conducting tests for the study, and a dated copy of current laboratory normal ranges for these tests, if applicable
- Local laboratory documentation demonstrating competence and test reliability (eg, accreditation/license), if applicable

17.3. Subject Identification, Enrollment, and Screening Logs

The investigator agrees to complete a subject identification and enrollment log to permit easy identification of each subject during and after the study. This document will be reviewed by the sponsor study-site contact for completeness.

The subject identification and enrollment log will be treated as confidential and will be filed by the investigator in the study file. To ensure subject confidentiality, no copy will be made. All reports and communications relating to the study will identify subjects by subject identification and date of birth. In cases where the subject is not randomized into the study, the date seen and date of birth will be used.

The investigator must also complete a subject screening log, which reports on all subjects who were seen to determine eligibility for inclusion in the study.

17.4. Source Documentation

At a minimum, source documents consistent in the type and level of detail with that commonly recorded at the study site as a basis for standard medical care, must be available for the following: subject identification, eligibility, and study identification; study discussion and date of signed informed consent; dates of visits; results of safety and immunogenicity parameters as required by the protocol; record of all adverse events and follow-up of adverse events; concomitant medication; study vaccine receipt/dispensing/return records; study vaccine administration information; and date of study completion and reason for early discontinuation of study vaccine or withdrawal from the study, if applicable.

The author of an entry in the source documents should be identifiable.

Specific details required as source data for the study and source data collection methods will be reviewed with the investigator before the study and will be described in the monitoring guidelines (or other equivalent document).

The subject diary will be considered a source document. Information from the diary provided to subjects to record symptoms of solicited local and systemic adverse events until 7 days after each vaccination will be reviewed by the investigator to transcribe into the relevant parts of the CRF as described in the CRF Completion Guidelines.

17.5. Case Report Form Completion

Case report forms are prepared and provided by the sponsor for each subject in electronic format. All data relating to the study must be recorded in CRF. All CRF entries, corrections, and alterations must be made by the investigator or authorized study-site personnel. The investigator must verify that all data entries in the CRF are accurate and correct.

The study data will be transcribed by study-site personnel from the source documents onto an electronic CRF, if applicable. Study-specific data will be transmitted in a secure manner to the sponsor.

Data must be entered into CRF in English. The CRF must be completed as soon as possible after a subject visit and the forms should be available for review at the next scheduled monitoring visit.

If necessary, queries will be generated in the electronic Data Capture (eDC) tool. If corrections to a CRF are needed after the initial entry into the CRF, this can be done in either of the following ways:

- Investigator and study-site personnel can make corrections in the eDC tool at their own initiative or as a response to an auto query (generated by the eDC tool).
- Sponsor or sponsor delegate can generate a query for resolution by the investigator and study-site personnel.

17.6. Data Quality Assurance/Quality Control

Steps to be taken to ensure the accuracy and reliability of data include the selection of qualified investigators and appropriate study sites, review of protocol procedures with the investigator and study-site personnel before the study, periodic monitoring visits by the sponsor and direct transmission of clinical laboratory data from a central laboratory into the sponsor's database. Written instructions will be provided for collection, handling, storage, and shipment of samples.

Guidelines for CRF completion will be provided and reviewed with study-site personnel before the start of the study. The sponsor will review CRF for accuracy and completeness during on-site monitoring visits and after transmission to the sponsor; any discrepancies will be resolved with the investigator or designee, as appropriate. After upload of the data into the study database they will be verified for accuracy and consistency with the data sources.

17.7. Record Retention

In compliance with the ICH/GCP guidelines, the investigator/institution will maintain all CRF and all source documents that support the data collected from each subject, as well as all study documents as specified in ICH/GCP Section 8, Essential Documents for the Conduct of a Clinical Trial, and all study documents as specified by the applicable regulatory requirement(s). The investigator/institution will take measures to prevent accidental or premature destruction of these documents.

Essential documents must be retained until at least 2 years after the last approval of a marketing application in an ICH region and until there are no pending or contemplated marketing applications in an ICH region or until at least 2 years have elapsed since the formal discontinuation of clinical development of the investigational product. These documents will be retained for a longer period if required by the applicable regulatory requirements or by an agreement with the sponsor. It is the responsibility of the sponsor to inform the investigator/institution as to when these documents no longer need to be retained.

If the responsible investigator retires, relocates, or for other reasons withdraws from the responsibility of keeping the study records, custody must be transferred to a person who will accept the responsibility. The sponsor must be notified in writing of the name and address of the new custodian. Under no circumstance shall the investigator relocate or dispose of any study documents before having obtained written approval from the sponsor.

If it becomes necessary for the sponsor or the appropriate regulatory authority to review any documentation relating to this study, the investigator/institution must permit access to such reports.

17.8. Monitoring

The sponsor will perform on-site monitoring visits as frequently as necessary. The monitor will record dates of the visits in a study site visit log that will be kept at the study site. The first post-initiation visit will be made as soon as possible after enrollment has begun. At these visits, the monitor will compare the data entered into the CRF with the source documents (eg, hospital/clinic/physician's office medical records). The nature and location of all source documents will be identified to ensure that all sources of original data required to complete the CRF are known to the sponsor and study-site personnel and are accessible for verification by the sponsor study-site contact. If electronic records are maintained at the study site, the method of verification must be discussed with the study-site personnel.

Direct access to source documents (medical records) must be allowed for the purpose of verifying that the recorded data are consistent with the original source data. Findings from this review will be discussed with the study-site personnel. The sponsor expects that, during monitoring visits, the relevant study-site personnel will be available, the source documents will be accessible, and a suitable environment will be provided for review of study-related documents. The monitor will meet with the investigator on a regular basis during the study to provide feedback on the study conduct.

There will be independent monitoring of the pharmacy and preparation of study vaccines by an unblinded monitor (independent study vaccine monitor, see Definitions of Terms); regular monitors will be blinded.

In addition to on-site monitoring visits, remote contacts can occur. It is expected that during these remote contacts, study-site personnel will be available to provide an update on the progress of the study at the site.

17.9. Study Completion/Termination

17.9.1. Study Completion/End of Study

The study is considered completed with the last visit for the last subject participating in the study. The final data from the study site will be sent to the sponsor (or designee) after completion of the final subject visit at that study site, in the time frame specified in the Clinical Trial Agreement.

17.9.2. Study Termination

The sponsor reserves the right to close the study site or terminate the study at any time for any reason at the sole discretion of the sponsor. Study sites will be closed upon study completion. A study site is considered closed when all required documents and study supplies have been collected and a study-site closure visit has been performed.

The investigator may initiate study-site closure at any time, provided there is reasonable cause and sufficient notice is given in advance of the intended termination.

Reasons for the early closure of a study site by the sponsor or investigator may include but are not limited to:

- Failure of the investigator to comply with the protocol, the requirements of the IEC/IRB or local health authorities, the sponsor's procedures, or GCP guidelines
- Inadequate recruitment of subjects by the investigator
- Discontinuation of further study vaccine development

17.10. On-site Audits

Representatives of the sponsor's clinical quality assurance department may visit the study site at any time during or after completion of the study to conduct an audit of the study in compliance with regulatory guidelines and company policy. These audits will require access to all study records, including source documents, for inspection. Subject privacy must, however, be respected. The investigator and study-site personnel are responsible for being present and available for consultation during routinely scheduled study-site audit visits conducted by the sponsor or its designees.

Similar auditing procedures may also be conducted by agents of any regulatory body, either as part of a national GCP compliance program or to review the results of this study in support of a regulatory submission. The investigator should immediately notify the sponsor if he/she has been contacted by a regulatory agency concerning an upcoming inspection.

17.11. Use of Information and Publication

All information, including but not limited to information regarding Ad26.ZEBOV and MVA-BN-Filo or the sponsor's operations (eg, patent application, formulas, manufacturing processes, basic scientific data, prior clinical data, formulation information) supplied by the sponsor to the investigator and not previously published, and any data, including exploratory research data,

generated as a result of this study, are considered confidential and remain the sole property of the sponsor. The investigator agrees to maintain this information in confidence and use this information only to accomplish this study, and will not use it for other purposes without the sponsor's prior written consent.

The investigator understands that the information developed in the study will be used by the sponsor in connection with the continued development of Ad26.ZEBOV and MVA-BN-Filo, and thus may be disclosed as required to other clinical investigators or regulatory agencies. To permit the information derived from the clinical studies to be used, the investigator is obligated to provide the sponsor with all data obtained in the study.

The results of the study will be reported in a Clinical Study Report generated by the sponsor and will contain data from all study sites that participated in the study as per protocol. Recruitment performance or specific expertise related to the nature and the key assessment parameters of the study will be used to determine a coordinating investigator. Results of exploratory analyses performed after the Clinical Study Report has been issued will be reported in a separate report and will not require a revision of the Clinical Study Report. Study subject identifiers will not be used in publication of results. Any work created in connection with performance of the study and contained in the data that can benefit from copyright protection (except any publication by the investigator as provided for below) shall be the property of the sponsor as author and owner of copyright in such work.

Consistent with Good Publication Practices and International Committee of Medical Journal Editors guidelines, the sponsor shall have the right to publish such primary (multicenter) data and information without approval from the investigator. The investigator has the right to publish study site-specific data after the primary data are published. If an investigator wishes to publish information from the study, a copy of the manuscript must be provided to the sponsor for review at least 60 days before submission for publication or presentation. Expedited reviews will be arranged for abstracts, poster presentations, or other materials. If requested by the sponsor in writing, the investigator will withhold such publication for up to an additional 60 days to allow for filing of a patent application. In the event that issues arise regarding scientific integrity or regulatory compliance, the sponsor will review these issues with the investigator. The sponsor will not mandate modifications to scientific content and does not have the right to suppress information. For multicenter study designs and substudy approaches, secondary results generally should not be published before the primary endpoints of a study have been published. Similarly, investigators will recognize the integrity of a multicenter study by not submitting for publication data derived from the individual study site until the combined results from the completed study have been submitted for publication, within 12 months of the availability of the final data (tables, listings, graphs), or the sponsor confirms there will be no multicenter study publication. Authorship of publications resulting from this study will be based on the guidelines on authorship, such as those described in the Uniform Requirements for Manuscripts Submitted to Biomedical Journals, which state that the named authors must have made a significant contribution to the design of the study or analysis and interpretation of the data, provided critical review of the paper, and given final approval of the final version.

Registration of Clinical Studies and Disclosure of Results

The sponsor will register and disclose the existence of and the results of clinical studies as required by law.

REFERENCES

1. Baden LR, Liu J, Li H, et al. Induction of HIV-1-specific mucosal immune responses following intramuscular recombinant adenovirus serotype 26 HIV-1 vaccination of humans. J Infect Dis. 2015;211(4):518-528.
2. Baden LR, Walsh SR, Seaman MS, et al. First-in-human evaluation of the safety and immunogenicity of a recombinant adenovirus serotype 26 HIV-1 Env vaccine (IPCAVD 001). J Infect Dis. 2013;207:240-247.
3. Baize S, Pannetier D, Oestereich L, et al. Emergence of Zaire Ebola virus disease in Guinea. N Engl J Med. 2014;371(15):1418-1425.
4. Barouch DH, Liu J, Peter L, et al. Characterization of humoral and cellular immune responses elicited by a recombinant adenovirus serotype 26 HIV-1 Env vaccine in healthy adults (IPCAVD 001). J Infect Dis. 2013;207:248-256.
5. Clinical Study Report MAL-V-A001. A Phase I/IIa, double-blind, randomized, placebo-controlled, dose-escalation clinical study evaluating safety, tolerability and immunogenicity of two dose levels of recombinant adenoviral serotype Ad35 and serotype Ad26 vectors expressing the malaria *Plasmodium falciparum* circumsporozoite antigen administered as heterologous prime-boost regimen, and assessing protective efficacy of the higher dose in a malaria challenge model in unblinded conditions. Crucell Holland B.V. (Aug 2014).
6. Clinical Study Report POX-MVA-013. A randomized, double-blind, placebo-controlled Phase III trial to evaluate immunogenicity and safety of three consecutive production lots of IMVAMUNE® (MVA-BN®) smallpox vaccine in healthy, Vaccinia-naïve subjects. Bavarian Nordic (Aug 2015).
7. Elizaga ML, Vasan S, Marovich MA, et al.; MVA Cardiac Safety Working Group. Prospective surveillance for cardiac adverse events in healthy adults receiving modified vaccinia Ankara vaccines: a systematic review. PLoS One. 2013;8(1):e54407.
8. Frey SE, Newman FK, Kennedy JS, et al. Clinical and immunologic responses to multiple doses of IMVAMUNE (Modified Vaccinia Ankara) followed by Dryvax challenge. Vaccine. 2007;25(51):8562-8573.
9. Friedrich B, Trefry J, Biggins J, et al. Potential vaccines and post-exposure treatments for filovirus infections. Viruses. 2012;4(9):1619-1650.
10. IMVANEX suspension for injection. UK Summary of Product Characteristics. Available at: http://www.ema.europa.eu/docs/en_GB/document_library/EPAR_-_Product_Information/human/002596/WC500147896.pdf. Accessed 26 March 2015.
11. Investigator Brochure: Ad26.ENVA.01 HIV-1 Vaccine. Edition 3. Crucell Holland B.V. (Jun 2013).
12. Investigator Brochure: JNJ-55471468-AAA, JNJ-55471494-AAA, JNJ-55471520-AAA (Ad26.Mos.HIV) HIV 1 vaccine. Edition 2. Crucell Holland B.V. (Nov 2014).
13. Investigator Brochure: JNJ-55471468-AAA, JNJ-55471494-AAA, JNJ-55471520-AAA (Ad26.Mos.HIV) HIV 1 vaccine. Addendum to Edition 2. Crucell Holland B.V. (Dec 2014).
14. Investigator's Brochure: JNJ-61210474 (Ad26.ZEBOV) Edition 2.1. Crucell Holland B.V. (5 June 2015).
15. Investigator's Brochure: MVA-BN-Filo (MVA-mBN226B) Edition 5. Bavarian Nordic A/S (18 March 2015).
16. Karita E, Mutua G, Bekker LG, et al. Safety in a Phase 1 randomized, double-blind, placebo-controlled trial evaluating two adenovirus hiv vaccines in three different geographic regions (IAVI-B003/IPCAVD-004/HVTN091 trial). Poster presented at AIDS Vaccine, 2013, Barcelona, Spain.
17. MVA-BN® (Modified Vaccinia Ankara - Bavarian Nordic). Available at: http://id.bavarian-nordic.com/pipeline/technology-platform/mva-bn.aspx Accessed 26 March 2015.
18. Neff J, Modlin J, Birkhead GS, et al. Monitoring the safety of a smallpox vaccination program in the United States: report of the joint Smallpox Vaccine Safety Working Group of the advisory committee on immunization practices and the Armed Forces Epidemiological Board. Clin Infect Dis. 2008;46 Suppl.3:S258-S270.

19. Sarwar UN, Costner P, Enama ME, et al. Safety and immunogenicity of DNA vaccines encoding ebolavirus and marburgvirus wild-type glycoproteins in a phase I clinical trial. J Infect Dis. 2015;211(4):549-557.

20. Stittelaar KJ, Kuiken T, de Swart RL et al. Safety of Modified Vaccinia Virus Ankara (MVA) in immune-suppressed macaques. Vaccine. 2001;19(27):3700-3709.

21. Verheust C, Goossens M, Pauwels K, Breyer D. Biosafety aspects of Modified Vaccinia Virus Ankara (MVA)-based vectors used for gene therapy or vaccination. Vaccine. 2012;30(16):2623-2632.

22. Vollmar J, Arndtz N, Eckl KM, et al. Safety and immunogenicity of IMVAMUNE, a promising candidate as a third generation smallpox vaccine. Vaccine. 2006;24(12):2065-2070.

23. WHO Fact Sheet N°103 Ebola Virus Disease 2014. Available at: http://www.who.int/mediacentre/factsheets/fs103/en/. Accessed 26 March 2015.

ATTACHMENTS

Attachment 1: Toxicity Tables for Use in Trials Enrolling Healthy Adults

The abbreviations used in the following tables are: ALT: alanine aminotransferase; aPTT: activated partial thromboplastin time; AST: aspartate aminotransferase; AV block: atrioventricular block; bpm: beats per minute; CK: creatine kinase; FEV_1: forced expiratory volume in 1 second; g: gram; HI: high; HPF: high power field; INR: international normalized ratio; IV: intravenous; LO: low; mEq: milliequivalent; mm Hg: millimeter of mercury; ms: millisecond; N: not graded; PT: prothrombin time; PTT: partial thromboplastin time; QTc: QT-interval corrected for heart rate; QTcB: Bazett's corrected QT interval; QTcF: Fridericia's corrected QT interval; RBC: red blood cell; Rx: therapy; s: second; U: unit; ULN: upper limit of normal

CLINICAL ADVERSE EVENTS

Grading scale used for clinical adverse events is adapted from the DMID Toxicity Tables (2014). For adverse events not included in the tables below, refer to the severity criteria guidelines in Section 12.1.3.

**Cardiovascular**	**Grade 1**	**Grade 2**	**Grade 3**
Arrhythmia		Asymptomatic, transient signs, no Rx required	Recurrent/persistent; symptomatic Rx required
Hemorrhage, blood loss	Estimated blood loss ≤100 mL	Estimated blood loss >100 mL, no transfusion required	Transfusion required
QTcF (Fridericia's correction) [a] or QTcB (Bazett's correction)	Asymptomatic, QTc interval 450-479 ms, *OR* Increase in interval<30 ms above baseline	Asymptomatic, QTc interval 480-499 ms, *OR* Increase in interval 30-50 ms above baseline	Asymptomatic, QTc interval ≥500 ms, *OR* Increase in interval ≥60 ms above baseline
PR interval (prolonged)	PR interval 0.21-0.25 s	PR interval >0.25 s	Type II 2nd degree AV block *OR* Ventricular pause >3.0 s
**Respiratory**	**Grade 1**	**Grade 2**	**Grade 3**
Cough	Transient-no treatment	Persistent cough	Interferes with daily activities
Bronchospasm, acute	Transient; no treatment; FEV_1 71%-80% of peak flow	Requires treatment; normalizes with bronchodilator; FEV_1 60%-70% (of peak flow)	No normalization with bronchodilator; FEV_1 <60% of peak flow
Dyspnea	Does not interfere with usual and social activities	Interferes with usual and social activities, no treatment	Prevents daily and usual social activity or requires treatment

[a] Inclusion dependent upon protocol requirements.

Gastrointestinal	Grade 1	Grade 2	Grade 3
Nausea/vomiting	Minimal symptoms; caused minimal or no interference with work, school or self-care activities	Notable symptoms; required modification in activity or use of medications; did not result in loss of work or cancellation of social activities	Incapacitating symptoms; required bed rest and/or resulted in loss of work or cancellation of social activities
Diarrhea	2-3 loose or watery stools or <400 g/24 hours	4-5 loose or watery stools or 400-800 g/24 hours	6 or more loose or watery stools or >800 g/24 hours or requires IV hydration
**Reactogenicity**	**Grade 1**	**Grade 2**	**Grade 3**
Local reactions			
Pain/tenderness at injection site	Aware of symptoms but easily tolerated; does not interfere with activity; discomfort only to touch	Notable symptoms; required modification in activity or use of medications; discomfort with movement	Incapacitating symptoms; inability to do work or usual activities; significant discomfort at rest
Erythema/redness[a]	2.5-5 cm	5.1-10 cm	>10 cm
Induration/swelling[b]	2.5-5 cm and does not interfere with activity	5.1-10 cm or interferes with activity	>10 cm or prevents daily activity
Itching at the injection site	Minimal symptoms; caused minimal or no interference with work, school or self-care activities	Notable symptoms; required modification in activity or use of medications; did not result in loss of work or cancellation of social activities	Incapacitating symptoms; required bed rest and/or resulted in loss of work or cancellation of social activities
Systemic reactions			
Allergic reaction	Pruritus without rash	Localized urticaria	Generalized urticaria; angioedema or anaphylaxis
Headache	Minimal symptoms; caused minimal or no interference with work, school or self-care activities	Notable symptoms; required modification in activity or use of medications; did not result in loss of work or cancellation of social activities	Incapacitating symptoms; required bed rest and/or resulted in loss of work or cancellation of social activities
Fatigue/malaise	Minimal symptoms; caused minimal or no interference with work, school or self-care activities	Notable symptoms; required modification in activity or use of medications; did not result in loss of work or cancellation of social activities	Incapacitating symptoms; required bed rest and/or resulted in loss of work or cancellation of social activities
Myalgia	Minimal symptoms; caused minimal or no interference with work, school or self-care activities	Notable symptoms; required modification in activity or use of medications; did not result in loss of work or cancellation of	Incapacitating symptoms; required bed rest and/or resulted in loss of work or cancellation of social activities

[a] In addition to grading the measured local reaction at the greatest single diameter, the measurement should be recorded as a continuous variable.
[b] Induration/swelling should be evaluated and graded using the functional scale as well as the actual measurement.

		social activities	
Arthralgia	Minimal symptoms; caused minimal or no interference with work, school or self-care activities	Notable symptoms; required modification in activity or use of medications; did not result in loss of work or cancellation of social activities	Incapacitating symptoms; required bed rest and/or resulted in loss of work or cancellation of social activities
Chills	Minimal symptoms; caused minimal or no interference with work, school or self-care activities	Notable symptoms; required modification in activity or use of medications; did not result in loss of work or cancellation of social activities	Incapacitating symptoms; required bed rest and/or resulted in loss of work or cancellation of social activities

LABORATORY TOXICITY GRADING

Grading scale used for lab assessments is based on 'FDA's Toxicity Grading Scale for Healthy Adult and Adolescent Volunteers Enrolled in Preventive Vaccine Clinical Trials', but grade 3 and 4 are pooled below, consistent with the 3 scale toxicity grading used throughout the protocol. **If a laboratory value falls within the grading as specified below but also within the laboratory normal limits, the value is considered as normal.** For hemoglobin only the change from reference is used for the grading. The FDA table does not include toxicity grading for hematocrit, red blood cell counts or INR.

Blood, Serum, or Plasma Chemistries [a,b]	LO/HI/N [c]	Mild (Grade 1)	Moderate (Grade 2)	Severe (Grade 3)
Sodium (mEq/L or mmol/L)	LO	132-134	130-131	≤129
	HI	144-145	146-147	≥148
Potassium (mEq/L or mmol/L)	LO	3.5-3.6	3.3-3.4	≤3.2
	HI	5.1-5.2	5.3-5.4	≥5.5
Glucose (mg/dL)	LO	65-69	55-64	≤54
	HI [d]	100-110	111-125	>125
	HI [e]	110-125	126-200	>200
Blood urea nitrogen	HI	23-26 (mg/dL) or 8.3-9.4 (mmol/L)	27-31 (mg/dL) or 9.5- 11.2 (mmol/L)	>31 (mg/dL) or >11.2 (mmol/L)
Creatinine	N	1.5-1.7 (mg/dL) or 133-151 (μmol/L)	1.8-2.0 (mg/dL) or 152-177 (μmol/L)	>2.0 (mg/dL) or >177 (μmol/L)
Calcium (mg/dL)	LO	8.0-8.4	7.5-7.9	<7.5
	HI	10.5-11.0	11.1-11.5	>11.5
Magnesium (mg/dL)	LO	1.3-1.5	1.1-1.2	<1.1
Phosphorus (mg/dL)	LO	2.3-2.5	2.0-2.2	<2.0
Creatine kinase (CK) (mg/dL)	N	1.25-1.5xULN	1.6-3.0xULN	≥3.1xULN
Albumin (g/dL)	LO	2.8-3.1	2.5-2.7	<2.5
Total protein (g/dL)	LO	5.5-6.0	5.0-5.4	<5.0
Alkaline phosphatase (U/L)	N	1.1-2xULN	2.1-3xULN	>3xULN
AST (U/L)	HI	1.1-2.5xULN	2.6-5xULN	>5xULN
ALT (U/L)	HI	1.1-2.5xULN	2.6-5xULN	>5xULN
Bilirubin, serum total (mg/dL) – when accompanied by any increase in Liver Function Test		1.1-1.25xULN	1.26-1.5xULN	>1.5xULN
Bilirubin, serum total (mg/dL) – when Liver Function Test is normal		1.1-1.5xULN	1.6-2.0xULN	>2.0xULN
Amylase (U/L)	N	1.1x1.5ULN	1.6-2.0xULN	>2.0xULN
Lipase (U/L)	N	1.1x1.5ULN	1.6-2.0xULN	>2.0xULN

[a] Depending upon the laboratory used, references ranges, eligibility ranges and grading may be split out by sex and/or age.
[b] Cardiac troponin I increase by factor: >ULN-<2.0xULN; ≥2.0-<5.0xULN; ≥5.0xULN. (This footnote is added by the sponsor).
[c] Low, High, Not Graded.
[d] Fasting.
[e] Non-fasting.

**Hematology**	**LO/HI/N** [a]	**Mild (Grade 1)**	**Moderate (Grade 2)**	**Severe (Grade 3)**
Hemoglobin (women) change from baseline (g/dL)	LO	Any decrease-1.5	1.6-2.0	>2.0
Hemoglobin (men) change from baseline (g/dL)	LO	Any decrease-1.5	1.6-2.0	>2.0
White blood cell count (cell/mm^3)	HI	10,800-15,000	15,001-20,000	>20,000
	LO	2,500-3,500	1,500-2,499	<1,500
Lymphocytes (cell/mm^3)	LO	750-1,000	500-749	< 500
Neutrophils (cell/mm^3)	LO	1,500-2,000	1,000-1,499	< 1000
Eosinophils (cell/mm^3)	HI	650-1500	1501-5000	> 5000
Platelets (cell/mm^3)	LO	125,000-140,000	100,000-124,000	<100,000
**Coagulation**				
Prothrombin time (PT, seconds)	HI	1.0-1.10xULN	1.11-1.20xULN	>1.20xULN
International normalized ratio (INR)[b]	HI	1.1-1.5xULN	1.6-2.0xULN	>2.0xULN
Partial thromboplastin time (PTT or aPTT, seconds)	HI	1.0-1.2xULN	1.21-1.4xULN	>1.4xULN
Fibrinogen (mg/dL)	HI	400-500	501-600	>600
	LO	150-200	125-149	<125
**Urine**				
Protein (dipstick)	HI	Trace	1+	2+
Glucose (dipstick)	HI	Trace	1+	2+
Blood (microscopic) - red blood cells per high power field (RBC/HPF)	HI	1-10	11-50	>50 and/or gross blood

[a] Low, High, Not Graded.
[b] For INR, the values in the table are based on the Division of AIDS Table for Grading the Severity of Adult and Pediatric Adverse Events, 2009.

VITAL SIGNS TOXICITY GRADING

Grading scale used for vital signs is according to DMID Toxicity Tables (2014)

Vital Signs	LO/HI/N [a]	Mild (Grade 1) [b]	Moderate (Grade 2)	Severe (Grade 3)
Fever (°C)[c]	HI	38.0-38.4	38.5-38.9	>38.9
Fever (°F)	HI	100.4-101.1	101.2-102.0	>102.0
Tachycardia - beats per minute	HI	101-115	116-130	>130 or ventricular dysrhythmias
Bradycardia - beats per minute	LO	50-54 or 45-50 bpm if baseline <60 bpm	45-49 or 40-44 bpm if baseline <60 bpm	<45 or <40 bpm if baseline <60 bpm
Hypertension (systolic) - mmHg[d]	HI	141-150	151-160	>160
Hypertension (diastolic) - mmHg	HI	91-95	96-100	>100
Hypotension (systolic) - mmHg	LO	85-89	80-84	<80
Tachypnea - breaths per minute	HI	23-25	26-30	>30

[a] Low, High, Not Graded.
[b] If initial bound of grade 1 has gap from reference range or eligibility range, calculations based on the New England Journal of Medicine (NEJM) reference ranges.
[c] Oral temperature; no recent hot or cold beverages or smoking. A protocol should select either °C or °F for inclusion.
[d] Assuming subject is awake, resting, and supine; for adverse events, 3 measurements on the same arm with concordant results.

Attachment 2: Batch variability of the Manufacturing Process in Terms of Binding Concentrations Against the EBOV GP

In the sample size calculations, a 10% difference between Ad26.ZEBOV batches in GMC of binding antibodies against the EBOV GP (EBOV GP protein ELISA) is used. This difference is based on the process variability in viral particle (vp) content of the batches, assessed via VP-qPCR assay, and assumed linear relationship between $\log_{10}$–transformed vaccine content (in vp) and $\log_{10}$–transformed GMC of binding antibodies (ELISA GMC).

Qualitatively, the Ad26.ZEBOV batches were found comparable.[a] The content of drug product (DP) for release and the content of drug substance (DS) determining the factor of dilution from DS to DP are both measured with the same VP-qPCR assay. The measured $\log_{10}$ content of 10 representative DP batches has a standard deviation of 0.049. The contents of these batches were taken from the Certificates of Analysis.[b]

No additional process variability due to dilution (and filtering) from DS to DP is assumed. The standard deviation of the true $\log_{10}$ DP content is then estimated as 0.035, according to the method described below. Subsequently, the difference in true $\log_{10}$ vp content between 2 DP batches has an estimated standard deviation of 0.049.

A linear relationship between $\log_{10}$ (vp content) and $\log_{10}$ (ELISA GMC) is estimated from ELISA GMC 4 weeks after Ad26.ZEBOV-vaccination from clinical studies VAC52150EBL1001 and VAC52150EBL1002 by linear regression and has a slope of 0.75 (see table below). The slope of 0.75 is similar to the slopes seen in mice studies.[c] Multiplying the slope with the $\log_{10}$ difference in content gives a 0.037 $\log_{10}$ GMC difference between batches or an almost 10% difference in ELISA GMC. This difference is used in the sample size calculations of the current study VAC52150EBL3003.

Geometric Mean Concentrations of Binding Antibodies Against the EBOV GP (Using ELISA) 4 Weeks After Ad26.ZEBOV Vaccination

Actual Ad26.ZEBOV vp content	Actual Ad26.ZEBOV vp content ($\log_{10}$ transformed)	n	ELISA GMC	(95% CI)
4.00E+10	10.60	15	477	(294 ; 776)
4.50E+10	10.65	29	556	(359 ; 860)
8.00E+10	10.90	15	802	(488 ; 1317)

Data from clinical studies VAC52150EBL1001 and VAC52150EBL1002

[a] Study Report DS-TEC-71991: Comparability Report of pre-change and post-change DS and DP manufacturing of Ad26.ZEBOV CTM for phase 3. Crucell Holland B.V. (Jan 2015).

[b] Certificate of Analysis, Ad26.ZEBOV, DP, 0.5mL, 1E11 VP/mL with document numbers NL-CTR-12777, NL-CTR-13413, NL-CTR-12774, NL-CTR-13553, NL-CTR-14031, NL-CTR-14033, NL-CTR-14095, C15026735/1, C15026737/1, C15036782/1.

[c] Zahn R, Gillisen G, Roos A, Koning M, van der Helm E, et al. Ad35 and Ad26 vaccine vectors induce potent and cross-reactive antibody and T cell responses to multiple filovirus species. PLoS ONE. 2012;7(12): e44115. doi:10.1371/journal.pone.0044115.

The linear relationship between $\log_{10}$ (vp content) and $\log_{10}$ (ELISA GMC) 8 weeks after Ad26.ZEBOV vaccination is assumed to be similar to the linear relationship between $\log_{10}$ (vp content) and $\log_{10}$ (ELISA GMC) 4 weeks after Ad26.ZEBOV vaccination.

Method to Calculate the Expected Difference in Content Between Batches

All values are assumed to be on log_{10} scale.

Assume true content of DS batch i is μ_i with $i = 1,2,3,\cdots$.

Assume measurement $Y_{1i} = \mu_i + \varepsilon_{1i}$ of DS batch i has measurement error $\varepsilon_{1i} \sim \mathrm{N}(0, \sigma^2)$.

The target for DP is τ. DS batch i is diluted to DP with a factor on log_{10} scale of $d_i = Y_{1i} - \tau$.

Assuming no additional process variability due to dilution (and filtering) the true content of DP batch i is $\tau_i = \mu_i - d_i = \tau - \epsilon_{1i}$. This means that $\tau_i \sim \mathrm{N}(\tau, \sigma^2)$ as distribution.

Assume measurement $Y_{2i} = \tau_i + \varepsilon_{2i}$ of DP batch i has also measurement error $\varepsilon_{2i} \sim \mathrm{N}(0, \sigma^2)$ since the same measurement format is used for both DS and DP.

The distribution for the difference in true content between 2 DP batches is $\tau_i - \tau_j \sim \mathrm{N}(0, 2\sigma^2)$. Now $Y_{2i} = \tau + \varepsilon_{1i} + \varepsilon_{2i} \sim \mathrm{N}(\tau, 2\sigma^2)$ gives an easy way to estimate $2\sigma^2$ as the variance of the DP measurements. Also $\left(\tau_i - \tau_j\right)^2 \sim 2\sigma^2\chi_1^2$ with expectation $2\sigma^2$.

INVESTIGATOR AGREEMENT

I have read this protocol and agree that it contains all necessary details for carrying out this study. I will conduct the study as outlined herein and will complete the study within the time designated.

I will provide copies of the protocol and all pertinent information to all individuals responsible to me who assist in the conduct of this study. I will discuss this material with them to ensure that they are fully informed regarding the study drug, the conduct of the study, and the obligations of confidentiality.

Coordinating Investigator (where required):

Name (typed or printed): ____________________

Institution and Address: ____________________

____

____

____

Signature: ____________________ Date: ____________________

(Day Month Year)

Principal (Site) Investigator:

Name (typed or printed): ____________________

Institution and Address: ____________________

____

____

____

Telephone Number: ____________________

Signature: ____________________ Date: ____________________

(Day Month Year)

Sponsor's Responsible Medical Officer:

Name (typed or printed): [redacted]

Institution: [redacted]

Signature: [electronic signature appended at the end of the protocol] Date: ____________________

(Day Month Year)

Note: If the address or telephone number of the investigator changes during the course of the study, written notification will be provided by the investigator to the sponsor, and a protocol amendment will not be required.

LAST PAGE

SIGNATURES

Signed by	Date	Justification
[redacted]	18Nov2015, 14:15:14 PM, UTC	Document Approval
