# Supplementary material for: Assessments of different batches and dose levels of a two-dose Ad26.ZEBOV and MVA-BN-Filo vaccine regimen
Source: NPJ Vaccines. 2021 Dec 20;6:157. doi: 10.1038/s41541-021-00402-8 (PMC8688528; doi:10.1038/s41541-021-00402-8)
Supplement: Supplementary file 2 — Reporting Summary [file 41541_2021_402_MOESM2_ESM.pdf]

nature portfolio

Corresponding author(s): Kerstin Luhn

Last updated by author(s): 2021 07 26

Reporting Summary

Nature Portfolio wishes to improve the reproducibility of the work that we publish. This form provides structure for consistency and transparency in reporting. For further information on Nature Portfolio policies, see our Editorial Policies and the Editorial Policy Checklist.

Statistics

For all statistical analyses, confirm that the following items are present in the figure legend, table legend, main text, or Methods section.

n/a	Confirmed	
☐	☒	The exact sample size (n) for each experimental group/condition, given as a discrete number and unit of measurement
☐	☒	A statement on whether measurements were taken from distinct samples or whether the same sample was measured repeatedly
☒	☐	The statistical test(s) used AND whether they are one- or two-sided<br>*Only common tests should be described solely by name; describe more complex techniques in the Methods section.*
☒	☐	A description of all covariates tested
☒	☐	A description of any assumptions or corrections, such as tests of normality and adjustment for multiple comparisons
☐	☒	A full description of the statistical parameters including central tendency (e.g. means) or other basic estimates (e.g. regression coefficient) AND variation (e.g. standard deviation) or associated estimates of uncertainty (e.g. confidence intervals)
☒	☐	For null hypothesis testing, the test statistic (e.g. F, t, r) with confidence intervals, effect sizes, degrees of freedom and P value noted<br>*Give P values as exact values whenever suitable.*
☒	☐	For Bayesian analysis, information on the choice of priors and Markov chain Monte Carlo settings
☒	☐	For hierarchical and complex designs, identification of the appropriate level for tests and full reporting of outcomes
☒	☐	Estimates of effect sizes (e.g. Cohen's d, Pearson's r), indicating how they were calculated

Our web collection on statistics for biologists contains articles on many of the points above.

Software and code

Policy information about availability of computer code

Data collection	No software was used.
Data analysis	SAS Version 9.2

For manuscripts utilizing custom algorithms or software that are central to the research but not yet described in published literature, software must be made available to editors and reviewers. We strongly encourage code deposition in a community repository (e.g. GitHub). See the Nature Portfolio guidelines for submitting code & software for further information.

Data

Policy information about availability of data

All manuscripts must include a data availability statement. This statement should provide the following information, where applicable:

- Accession codes, unique identifiers, or web links for publicly available datasets
- A description of any restrictions on data availability
- For clinical datasets or third party data, please ensure that the statement adheres to our policy

Janssen has an agreement with the Yale Open Data Access (YODA) Project to serve as the independent review panel for evaluation of requests for CSRs and participant level data from investigators and physicians for scientific research that will advance medical knowledge and public health. Data will be made available following publication and approval by YODA of any formal requests with a defined analysis plan. For more information on this process or to make a request, please visit The Yoda Project site at http://yoda.yale.edu. The data sharing policy of Janssen Pharmaceutical Companies of Johnson & Johnson is available at https://www.janssen.com/clinical-trials/transparency.

Field-specific reporting

Please select the one below that is the best fit for your research. If you are not sure, read the appropriate sections before making your selection.

☒ Life sciences ☐ Behavioural & social sciences ☐ Ecological, evolutionary & environmental sciences

For a reference copy of the document with all sections, see nature.com/documents/nr-reporting-summary-flat.pdf

Life sciences study design

All studies must disclose on these points even when the disclosure is negative.

Sample size	In Study 1, the sample size was calculated assuming a 5% type I error rate, a standard deviation of 0.323 for log10-transformed binding antibodies 56 days after Ad26.ZEBOV and a 10% difference in GMCs between batches. With 94 participants, the power was 83% to conclude equivalence between batches with margins of ⅔ (0.67) and 1½ (1.5).<br>The sample size for Study 2 was based on the assumption that the standard deviation for log10-transformed binding antibodies was 0.303 at 21 days post-MVA-BN-Filo, based on data from a phase 1 study16, and GMCs for intermediate and low dose levels would be at least 90% of the full clinical dose. For 90% power, including a 10% drop-out rate, a total of 150 participants were needed per group.
Data exclusions	The safety analyses were based on the full Analysis Set (i.e. all participants who were randomized and received at least one dose of vaccine or placebo). All immunogenicity analyses were based on the per protocol analysis set, which included all randomized and vaccinated participants who received both Ad26.ZEBOV (dose 1) and MVA-BN-Filo (dose 2) vaccinations within the protocol-defined window, who had at least one post-vaccination evaluable immunogenicity blood sample, and who had no major protocol deviations that could influence the immune response. These analyses sets were defined prior to database lock and unblinding.
Replication	All analyses of study data were initially performed by a statistical programmer, and subsequently validated by another independent statistical programmer.
Randomization	In both studies, participants were randomized (2:2:2:1) at enrollment to one of four groups using a computer-generated schedule (via an Interactive Web Response System) provided by the sponsor, balanced using randomly permuted blocks, and stratified by site.
Blinding	All vaccinations were administered by study personnel blinded to vaccine or placebo, or batch being used; masking tape was used to cover the dispensing syringes containing the treatment allocated.

Reporting for specific materials, systems and methods

We require information from authors about some types of materials, experimental systems and methods used in many studies. Here, indicate whether each material, system or method listed is relevant to your study. If you are not sure if a list item applies to your research, read the appropriate section before selecting a response.

Materials & experimental systems

n/a	Involved in the study	
☐	☒	Antibodies
☒	☐	Eukaryotic cell lines
☒	☐	Palaeontology and archaeology
☒	☐	Animals and other organisms
☐	☒	Human research participants
☐	☒	Clinical data
☒	☐	Dual use research of concern

Methods

n/a	Involved in the study	
☒	☐	ChIP-seq
☒	☐	Flow cytometry
☒	☐	MRI-based neuroimaging

Antibodies

Antibodies used	Horseradish peroxidase (HRP)-conjugated goat anti-human IgG (catalogue number 109-035-098, Fcγ fragment-specific; Jackson ImmunoResearch Laboratories, Inc.; West Grove, PA)
Validation	Validated for use in human and non-human primate sera

Human research participants

Policy information about studies involving human research participants

Population characteristics	A total of 329 participants were enrolled and randomized in Study 1, and 525 in Study 2, according to the study designs detailed in Fig. 1. In Study 1 and Study 2, respectively, the first participant was enrolled on 21 September 2015 and 30 July 2015, and the date of the last participant last visit was 20 July 2016 and 29 November 2016. In general, the demographics

were similar across groups within each study (Table 1). While there was a higher proportion Hispanic or Latino participants (19.5%) in Study 1 than Study 2 (6.7%), Study 2 had a higher proportion of White participants (79.6%) than Study 1 (57.4%).

Recruitment

Health volunteers recruited from the local population

Ethics oversight

The protocol for each study was approved by a central IRB (MaGil IRB, Rockville).

Note that full information on the approval of the study protocol must also be provided in the manuscript.

Clinical data

Policy information about clinical studies

All manuscripts should comply with the ICMJE guidelines for publication of clinical research and a completed CONSORT checklist must be included with all submissions.

Clinical trial registration

NCT02543268; NCT02543567

Study protocol

The protocols for each study have been uploaded to the Nature Research Protocol Exchange.

Data collection

In Study 1 and Study 2, respectively, the first participant was enrolled on 21 September 2015 and 30 July 2015, and the date of the last participant last visit was 20 July 2016 and 29 November 2016.
Both studies were performed under the supervision of the same coordinating investigator in multiple sites in the USA; Study 1 (Mishawaka, Indiana; Rockville, Maryland; San Diego, California) and Study 2 (Huntsville, Alabama; Melbourne, Florida; Peoria, Illinois; Rockville, Maryland).

Outcomes

In Study 1 the primary objective was to demonstrate equivalence of EBOV GP binding antibody responses measured by FANG ELISA at 56 days post-Ad26.ZEBOV in groups whose participants were administered with vaccine batch produced with the WVS in the commercial process (Group 2) and vaccine batch from the MVS used in phase 2 studies (Group 3). Equivalence was considered to have been met if the 95% CI of the estimated GMC ratio was entirely within the predefined range of ⅔ (0.67) to 1½ (1.5). The GMC ratio and its 95% CI was determined by computing the difference between the log10-transformed ELISA concentrations (EU/mL) between groups, and back-transforming the estimated difference and its 95% CI. Secondary objectives were to demonstrate: 1) equivalence of Ad26.ZEBOV batches manufactured in Leiden from WVS (Group 1) and the MVS (Group 3) at 56 days post-Ad26.ZEBOV; 2) equivalence of Ad26.ZEBOV batch manufactured in Leiden from WVS (Group 1) and the batch manufactured in Bern from WVS (Group 2) at 56 days post-Ad26.ZEBOV; 3) equivalence of 3 different Ad26.ZEBOV batches administered as dose 1 followed by a single dose of MVA-BN-Filo 56 days later, at the 21 days post-dose 2 time point, using the same equivalence margin.

The primary objective of Study 2 was to demonstrate non-inferiority of the intermediate-dose level to the full clinical dose, based on the GMCs of EBOV GP binding antibodies measured by FANG ELISA at 21 days post- MVA-BN-Filo (day 78), using a predefined non-inferiority margin of ⅔ (0.67). If the primary objective would be met, non-inferiority of the low-dose level to the full clinical dose would be evaluated in the same way (hierarchical testing). Additionally, a pre-planned exploratory non-inferiority analysis was performed at 21 days post-MVA-BN-Filo, using a margin of ½ (0.5). This non-inferiority criterion was used in an ongoing phase 3 lot-to-lot study assessing consistency of Ad26.ZEBOV and MVA-BN-Filo manufacturing and was applied here for consistency31. A post-hoc exploratory analysis was performed at 56 days post-Ad26.ZEBOV, using a non-inferiority margin of ⅔ (0.67), as per regulatory authority request.
For each pair-wise comparison, estimated differences were expressed as ratios of GMCs with 95% CI, determined from comparing the log10-transformed ELISA concentrations (EU/mL) between groups and back-transformation of the estimated difference and corresponding 95% CI. Non-inferiority was to be demonstrated if the 95% CI of the estimated GMC ratio was entirely above the non-inferiority margin.
